# Supplementary material for: The effects of high versus low talker variability and individual aptitude on phonetic training of Mandarin lexical tones
Source: PeerJ. 2019 Aug 9;7:e7191. doi: 10.7717/peerj.7191 (PMC6690337; doi:10.7717/peerj.7191)
Supplement: Supplemental Information 1 — Thirty-six pairs of Mandarin words and their corresponding pictures. [file peerj-07-7191-s001.docx]

**Appendix A** Thirty-six pairs of Mandarin words and their corresponding pictures.

| **List** | **Tone Contrast** | **Item 1** | **Item 2** |
| --- | --- | --- | --- |
| Trained | 1-2 | Chuāng (window, Tone 1) | Chuáng (bed, Tone 2) |
|  |  | Māo (cat) | Máo (anchor) |
|  |  | Qiān (swing) | Qián (money) |
|  | 1-3 | Jiāo (glue) | Jiǎo (foot) |
|  |  | Shū (comb) | Shǔ (mouse) |
|  |  | Xuē (boot) | Xuě (snow) |
|  | 1-4 | Bā (eight) | Bà (father) |
|  |  | Huā (flower) | Huà (paint) |
|  |  | Zhū (pig) | Zhù (pour) |
|  | 2-3 | Bí (nose) | Bǐ (pen) |
|  |  | Wán (to play) | Wǎn (bowl) |
|  |  | Niú (cow) | Niǔ (button) |
|  | 2-4 | Dí (siren) | Dì (earth) |
|  |  | Xié (shoe) | Xiè (crab) |
|  |  | Yún (cloud) | Yùn (iron) |
|  | 3-4 | Dǎn (brush, verb) | Dàn (egg) |
|  |  | Mǐ (rǐce) | Mì (honey) |
|  |  | Yǎn (eye) | Yàn (flame) |
| Novel | 1-2 | Shī (teacher) | Shí (ten) |
|  |  | Tuō (mop) | Tuó (to carry) |
|  |  | Tī (ladder) | Tí (hoof) |
|  | 1-3 | Mā (mother) | Mǎ (horse) |
|  |  | Guī (tortoǐse) | Guǐ (ghost) |
|  |  | Zhēn (needle) | Zhěn (pǐllow) |
|  | 1-4 | Dēng (lamp) | Dèng (bench) |
|  |  | Kū (cry) | Kù (trousers) |
|  |  | Xǐāng (box) | Xiàng (elephant) |
|  | 2-3 | Chí (spoon) | Chǐ (ruler) |
|  |  | Hú (moustache) | Hǔ (tǐger) |
|  |  | Yú (fǐsh) | Yǔ (feather) |
|  | 2-4 | Mó (mushroom) | Mò (mill) |
|  |  | Shé (snake) | Shè (house) |
|  |  | Wá (baby) | Wà (sock) |
|  | 3-4 | Bǎo (treasure) | Bào (newspaper) |
|  |  | Dǎo (island) | Dào (road) |
|  |  | Jǐǎn (scissors) | Jiàn (arrow) |
