# Supplementary material for: The effects of high versus low talker variability and individual aptitude on phonetic training of Mandarin lexical tones
Source: PeerJ. 2019 Aug 9;7:e7191. doi: 10.7717/peerj.7191 (PMC6690337; doi:10.7717/peerj.7191)
Supplement: Supplemental Information 3 [file peerj-07-7191-s003.html]

Mandarin Training Study: High versus Low Speaker Variability


Code 

- Show All Code
- Hide All Code
- Download Rmd

# Mandarin Training Study: High versus Low Speaker Variability

#### *July, 2018*

- Load Packages and Helper Functions
  - Packages
  - Helper Functions
    - logodds
    - SummarySE
    - SummarySEwithin
    - normDataWithin
    - myCenter
    - lizCenter
    - lizContrasts
    - filter2
    - get\_coeffs
- Load Datasets
- Individual Aptitude Task 1: Pitch Contour Perception Test (Section 3.2.1)
  - Figure (Figure 3)
  - Statistical Analysis (Section 3.2.1)
- Individual Aptitude Task 2: Categorisation of Synthesized Tonal Continua (Section 3.2.2)
  - Extract slope coffcient for each participant and descriptives
  - Graph
  - Statistical Analysis
- Look at the relationship between PCPT and CSTC and set up ID\_pre table
  - Correlation Figure
  - Create a median split (this is used later in plotting, but not in analyses)
- Production Reliability Analysis (Section 3.5.1.1)
  - Checking the accuracy of ratings of production data by native speakers
  - Checking the correlation between Rater1 and Rater2
    - Rater accuracy of tone identification in Word Repetition
    - Rater accuracy of pinyin identification in Word Repetition
    - Rater accuracy of tone rating in Word Repetition
    - Rater accuracy of tone identification in Picture Naming
    - Rater accuracy of pinyin identification in Picture Naming
    - Rater accuracy of tone rating in Picture Naming
- Word Repetition (Section 3.5.1)
  - Word Repetition - Tone accuracy (Section 3.5.1.2)
    - Label Parameters and remove unidentifiable trials
    - Figures
      - Main Graph (Figure 7)
      - Graph (Figure 12, Left panel)
      - CSTC Graph (Not used)
    - Statistical Analysis
      - Main Model (without aptitude predictors; Section 3.5.1.2)
      - Analysis with PCPT accuracy (Section 3.7)
      - Analysis with CSTC slope (Section 3.7)
  - Word Repetition - Pinyin Accuracy (Section 3.5.1.3)
    - Figures
      - Main Graph (Figure 8)
      - PCPT Graph (Figure 12, Right panel)
      - CSTC Graph (Not used)
    - Statistical Analysis
      - Main Model (without aptitude predictors; Section 3.5.1.3)
      - Analysis with PCPT accuracy (Section 3.7)
      - Analysis with CSTC slope
- Three-interval Oddity Task (Section 3.4.1)
  - Label Parameters
  - Figures
    - Main Graph (Figure 5)
    - PCPT graph (Figure 10, top panel)
    - CSTC graph (Not included)
    - PCPT Graph (Dot plot, not currently used)
    - CSTC Graph (Dot plot, not currently used)
  - Statistical Analysis
    - Main model (without aptitude predictors; Section 3.4.1)
      - Print out key effects at pre-test session
      - Print out key effects relevant descriptive statistics of test-session and interactions with test-session (i.e. looking effects of training)
      - Examine if learning is different for the easier/harder trial types and print out relevant descriptive statistics
    - Analyses with PCPT accuracy (Section 3.7)
      - Print out key effects of test-session and interactions with test-session (i.e. looking effects of training)
      - Check that we get the same pattern of results for the experimentally manipulated variables as in the previous model
    - Analyses-with CSTC slope
      - Print out key effects of test-session and interactions with test-session (i.e. looking effects of training)
      - Check that we get the same pattern of results for the experimentally manipulated variables as in the previous model
- Training (Section 3.3)
  - Parameters with labels
  - Figures
    - Main Graph (Figure 4)
    - PCPT Graph (Figure 10, Bottom panel)
    - CSTC graph (Not used)
  - Statistical Analysis
    - Main Model (without aptitude predictors; Section 3.3)
      - Print key cofficients
    - Analysis with PCPT accuracy (Section 3.7)
      - Print key cofficients
    - Extra analysis requested by reviewers, with categorical aptitude measure from PCPT accuracy
      - Print key cofficients
    - Analysis with CSTC slope
- Picture Identification (Section 3.4.2)
  - Label Parameters
  - Figures
    - Main Graph (Figure 6)
    - Main Graph 2 (Scatter Plot, Figure 11, Bottom panel, right)
    - PCPT Graph (Figure 11 Bottom panel, left)
    - CSTC graph (Not used)
  - Statistical Analysis
    - Main Model (without individual difference measures; Section 3.4.2)
      - Print out key effects
      - Re-fit the model with a separate slope for voicetyp.ct for each condition"
      - To check it is the same model
      - Print out effect of voice-type for each condition
      - Re-fit the model with a separate slope for the contrast between conditions for trained and untrained nouns
      - To check it is the same model
      - Print out the contrast between conditions for trained and untrained nouns
    - Analysis with PCPT accuracy (Section 3.7)
      - Check that we get the same pattern of results for the experimentally manipulated variables as in the previous model
      - Print out the effect of individual aptitude and key interactions with aptitude
    - Analysis with CSTC slope
      - Check that we get the same pattern of results for the experimentally manipulated variables as in the previous model
      - Print out the effect of individual aptitude and key interactions with aptitude
- Picture Naming (Section 3.5.2)
  - Picture Naming - Tone accuracy
    - Label parameters and remove unidentifiable trials
    - Figures
      - Main Figure (Figure 9, left)
      - Main Figure (Scatter plot, Figure 11, Top panel, right)
      - PCPT Graph (Figure 11 Top panel,left)
      - CSTC graph (Not used)
    - Statistical Analysis
      - Main model (without aptitude predictors; Section 3.5.2)
      - Analysis with PCPT accuracy (Section 3.7)
      - Analysis with CSTC slope
  - Picture Naming - Pinyin Accuracy
    - Figures
      - Main Graph (Figure 9, right)
      - Main Graph (Scatter plot, Figure 11, Middle panel, right)
      - PCPT Graph (Figure 11, Middle panel,left)
      - CSTC Graph (Not used)
    - Statistical Analysis
      - Main Model (without individual aptitude measures; section 3.5.2)
      - Print out key effects
      - Analysis with PCPT accuracy (Section 3.7)
      - Analysis with CSTC slope

# Load Packages and Helper Functions

## Packages


```
rm(list=ls())
suppressPackageStartupMessages(library(ggplot2))
suppressPackageStartupMessages(library(lmerTest))
suppressPackageStartupMessages(library(lme4))
suppressPackageStartupMessages(library(Matrix))
suppressPackageStartupMessages(library(gridExtra))
suppressPackageStartupMessages(library(plyr))
suppressPackageStartupMessages(library(knitr))
suppressPackageStartupMessages(library(fmsb))
suppressPackageStartupMessages(library(irr))
suppressPackageStartupMessages(library(cowplot))
suppressPackageStartupMessages(library(kableExtra))
suppressPackageStartupMessages(theme_set(theme_bw()))
opts_chunk$set(fig.width=8, fig.height=5, echo=TRUE, warning=FALSE, message=FALSE, cache=TRUE)
```

## Helper Functions

### logodds

This function takes a percentage and returns the log odds.


```
logodds <- function(p){log(p/(100-p))}
```

### SummarySE

This function can be found on the website “Cookbook for R”.

http://www.cookbook-r.com/Graphs/Plotting\_means\_and\_error\_bars\_(ggplot2)/#Helper

It summarizes data, giving count, mean, standard deviation, standard error of the mean, and confidence interval (default 95%).

- data: a data frame
- measurevar: the name of a column that contains the variable to be summarized
- groupvars: a vector containing names of columns that contain grouping variables
- na.rm: a boolean that indicates whether to ignore NA’s
- conf.interval: the percent range of the confidence interval (default is 95%)


```
summarySE <- function(data = NULL, measurevar, groupvars = NULL, na.rm = FALSE, conf.interval = .95, .drop = TRUE) {require(plyr)
    # New version of length which can handle NA's: if na.rm==T, don't count them
    length2 <- function (x, na.rm = FALSE) {
        if (na.rm) sum(!is.na(x))
        else       length(x)
    }
    # This does the summary. For each group's data frame, return a vector with
    # N, mean, and sd
    datac <- ddply(data, groupvars, .drop = .drop,
      .fun = function(xx, col) {
        c(N    = length2(xx[[col]], na.rm = na.rm),
          mean = mean   (xx[[col]], na.rm = na.rm),
          sd   = sd     (xx[[col]], na.rm = na.rm)
        )
      },
      measurevar
    )
    # Rename the "mean" column    
    datac <- rename(datac, c("mean" = measurevar))
    datac$se <- datac$sd / sqrt(datac$N)  # Calculate standard error of the mean
    # Confidence interval multiplier for standard error
    # Calculate t-statistic for confidence interval: 
    # e.g., if conf.interval is .95, use .975 (above/below), and use df=N-1
    ciMult <- qt(conf.interval/2 + .5, datac$N-1)
    datac$ci <- datac$se * ciMult
    return(datac)
}
```

### SummarySEwithin

This function can be found on the website “Cookbook for R”.

http://www.cookbook-r.com/Graphs/Plotting\_means\_and\_error\_bars\_(ggplot2)/#Helper

From that website:

Summarizes data, handling within-subject variables by removing inter-subject variability. It will still work if there are no within-subject variables. Gives count, un-normed mean, normed mean (with same between-group mean), standard deviation, standard error of the mean, and confidence interval. If there are within-subject variables, it calculates adjusted values using the method from Morey (2008).

- data: a data frame
- measurevar: the name of a column that contains the variable to be summarized
- betweenvars: a vector containing names of columns that are between-subjects variables
- withinvars: a vector containing names of columns that are within-subjects variables
- idvar: the name of a column that identifies each subject (or matched subjects)
- na.rm: a boolean that indicates whether to ignore NA’s
- conf.interval: the percent range of the confidence interval (default is 95%)


```
summarySEwithin <- function(data = NULL, measurevar, betweenvars = NULL, withinvars = NULL,
                            idvar = NULL, na.rm = FALSE, conf.interval = .95, .drop = TRUE) {
  # Ensure that the betweenvars and withinvars are factors
  factorvars <- vapply(data[, c(betweenvars, withinvars), drop = FALSE],
    FUN = is.factor, FUN.VALUE = logical(1))
  if (!all(factorvars)) {
    nonfactorvars <- names(factorvars)[!factorvars]
    message("Automatically converting the following non-factors to factors: ",
            paste(nonfactorvars, collapse = ", "))
    data[nonfactorvars] <- lapply(data[nonfactorvars], factor)
  }
  # Get the means from the un-normed data
  datac <- summarySE(data, measurevar, groupvars = c(betweenvars, withinvars),
                     na.rm = na.rm, conf.interval = conf.interval, .drop = .drop)
  # Drop all the unused columns (these will be calculated with normed data)
  datac$sd <- NULL
  datac$se <- NULL
  datac$ci <- NULL
  # Norm each subject's data
  ndata <- normDataWithin(data, idvar, measurevar, betweenvars, na.rm, .drop = .drop)
  # This is the name of the new column
  measurevar_n <- paste(measurevar, "_norm", sep ="")
  # Collapse the normed data - now we can treat between and within vars the same
  ndatac <- summarySE(ndata, measurevar_n, groupvars = c(betweenvars, withinvars),
                      na.rm = na.rm, conf.interval = conf.interval, .drop = .drop)
  # Apply correction from Morey (2008) to the standard error and confidence interval
  # Get the product of the number of conditions of within-S variables
  nWithinGroups    <- prod(vapply(ndatac[,withinvars, drop = FALSE], FUN = nlevels,
                           FUN.VALUE = numeric(1)))
  correctionFactor <- sqrt( nWithinGroups / (nWithinGroups-1) )
  # Apply the correction factor
  ndatac$sd <- ndatac$sd * correctionFactor
  ndatac$se <- ndatac$se * correctionFactor
  ndatac$ci <- ndatac$ci * correctionFactor
  # Combine the un-normed means with the normed results
  merge(datac, ndatac)
}
```

### normDataWithin

This function is used by the SummarySEWithin function above. It can be found on the website “Cookbook for R”.

http://www.cookbook-r.com/Graphs/Plotting\_means\_and\_error\_bars\_(ggplot2)/#Helper

From that website:

Norms the data within specified groups in a data frame; it normalizes each subject (identified by idvar) so that they have the same mean, within each group specified by betweenvars.

- data: a data frame
- idvar: the name of a column that identifies each subject (or matched subjects)
- measurevar: the name of a column that contains the variable to be summarized
- betweenvars: a vector containing names of columns that are between-subjects variables
- na.rm: a boolean that indicates whether to ignore NAs


```
normDataWithin <- function(data = NULL, idvar, measurevar, betweenvars = NULL,
                           na.rm = FALSE, .drop = TRUE) {
    #library(plyr)
    # Measure var on left, idvar + between vars on right of formula.
    data.subjMean <- ddply(data, c(idvar, betweenvars), .drop = .drop,
     .fun = function(xx, col, na.rm) {
        c(subjMean = mean(xx[,col], na.rm = na.rm))
      },
      measurevar,
      na.rm
    )
    # Put the subject means with original data
    data <- merge(data, data.subjMean)
    # Get the normalized data in a new column
    measureNormedVar <- paste(measurevar, "_norm", sep = "")
    data[,measureNormedVar] <- data[,measurevar] - data[,"subjMean"] +
                               mean(data[,measurevar], na.rm = na.rm)
    # Remove this subject mean column
    data$subjMean <- NULL
    return(data)
}
```

### myCenter

This function outputs the centered values of a variable, which can be a numeric variable, a factor, or a data frame. It was taken from Florian Jaeger’s blog - https://hlplab.wordpress.com/2009/04/27/centering-several-variables/.

From his blog:

- If the input is a numeric variable, the output is the centered variable
- If the input is a factor, the output is a numeric variable with centered factor level values. That is, the factor’s levels are converted into numerical values in their inherent order (if not specified otherwise, R defaults to alphanumerical order). More specifically, this centers any binary factor so that the value below 0 will be the 1st level of the original factor, and the value above 0 will be the 2nd level.
- If the input is a data frame or matrix, the output is a new matrix of the same dimension and with the centered values and column names that correspond to the colnames() of the input preceded by “c” (e.g. “Variable1” will be “cVariable1”).


```
myCenter = function(x) {
  if (is.numeric(x)) { return(x - mean(x, na.rm = T)) }
    if (is.factor(x)) {
        x= as.numeric(x)
        return(x - mean(x, na.rm = T))
    }
    if (is.data.frame(x) || is.matrix(x)) {
        m= matrix(nrow = nrow(x), ncol = ncol(x))
        colnames(m)= paste("c", colnames(x), sep = "")
    
        for (i in 1:ncol(x)) {
        
            m[,i] = myCenter(x[,i])
        }
        return(as.data.frame(m))
    }
}
```

### lizCenter

This function provides a wrapper around myCenter allowing you to center a specific list of variables from a data frame.

- x: data frame
- listfname: a list of the variables to be centered (e.g. list(variable1, variable2))

The output is a copy of the data frame with a column (always a numeric variable) added for each of the centered variables. These columns are labelled with the each column’s previous name, but with “.ct” appended (e.g., “variable1” will become “variable1.ct”).


```
lizCenter = function(x, listfname) 
{
    for (i in 1:length(listfname)) 
    {
        fname = as.character(listfname[i])
        x[paste(fname,".ct", sep="")] = myCenter(x[fname])
    }
        
    return(x)
}
```

### lizContrasts

This function can be used to create two centered dummy variables which stand in place of a three-way factor (condition). This allows us to inspect each contrast separately, as well as their interactions with other factors. Other fixed effects in the model can be evaluated as the average effects across all levels of the factor.

The function takes a data frame (d), a factor from that database (condition), which must have three levels, and the name of the level of the factor which is to be used as the baseline for the contrasts (base level).

For example, if d is a data frame with a factor “condition” with three levels “(”lex\_skew“,”lex\_noskew“, and”mixed“) then lizContrasts(d, d$condition,”lex\_no\_skew“) returns a data frame with two (numeric) columns added labelled”lex\_noskew\_VERSUS\_lex\_mixed" and “lex\_noskew\_VERSUS\_lex\_skew”.

Wherever you would normally use “condition”in a formula in an LME, it can be replaced by (lex\_noskew\_VERSUS\_lex\_mixed + “lex\_noskew\_VERSUS\_lex\_skew) e.g. ~ (a \* condition) becomes ~ (a \* (lex\_noskew\_VERSUS\_lex\_mixed + lex\_noskew\_VERSUS\_lex\_skew)).


```
lizContrasts = function(d, condition, baselevel) 
{
  
    condition = factor(condition)
  condition = relevel(condition, baselevel)
    a = (contrasts(condition)-apply(contrasts(condition),2,mean))
    d$dummy1[condition == rownames(a)[1]] <- a[1] 
    d$dummy1[condition == rownames(a)[2]] <- a[2] 
    d$dummy1[condition == rownames(a)[3]] <- a[3] 
    d$dummy2[condition == rownames(a)[1]] <- a[4] 
    d$dummy2[condition == rownames(a)[2]] <- a[5] 
    d$dummy2[condition == rownames(a)[3]] <- a[6] 
    name1 = paste(baselevel, rownames(a)[2],sep ="_VERSUS_")
    name2 = paste(baselevel, rownames(a)[3],sep ="_VERSUS_")
    d[name1] = d$dummy1 
    d[name2] = d$dummy2 
    d$dummy1 <-NULL 
    d$dummy2 <-NULL 
    
    return(d)
}
```

### filter2

This is a function which filters a column of data removing values which a certain number of standard deviations above/below the mean for that participant, possibly in some condition/sub-condition.

- im: the input matrix (a data frame)
- svn: a list of the names of factors to be group by (subject name + one or more conditions)
- fn: the name of the column containing the data to be filtered
- lim: how many standard deviations above/below the mean to filter

The function returns an input matrix identical to the original input matrix but with additional columns giving the group means and the “filtered” data


```
filter2 = function(im, svn, fn, lim)
{
  ## work out means for each subject for each word
x = list()
y = ""
for(n in svn) x = append(im[n],x)
for(n in svn) y = paste(y,n,sep = "_")
means = aggregate(im[fn], by = x, mean, na.rm = T)
head(means)
nocols = dim(means)[2]
colnames(means)[nocols] = "means"
sds = aggregate(im[fn], by = x, sd, na.rm = T)
head(sds)
nocols = dim(sds)[2]
colnames(sds)[nocols] = "sds"
gs = merge(means,sds)
## because if there is just one value it does not have a standard deviation and we do not want to just disregard all of these
gs$sds[is.na(gs$sds)] = 0 
gs$max = gs$means + lim*gs$sds
gs$min = gs$means - lim*gs$sds
im2 = merge(im, gs, sort = F)
im2[paste(fn, "filt", sep = "")] = im2[fn]
cn = dim(im2)[2] ## get colnumber (last one added)
im2[,cn][im2[,fn] > im2$max] = ""
im2[,cn][im2[,fn] < im2$min] = ""
im2[,cn] = as.numeric(im2[,cn])
names(im2)[names(im2) == "means"] = paste("mean", y, sep = "_") 
names(im2)[names(im2) =="sds"] = paste("sd", y, sep = "_") 
names(im2)[names(im2) =="max"] = paste("max", y, sep = "_") 
names(im2)[names(im2) =="min"] = paste("min", y, sep = "_") 
return(im2)
}
```

### get\_coeffs

This function allows us to inspect particular coefficients from the output of an LME model by putting them in table.

- x: the output returned when running lmer or glmer (i.e. an object of type lmerMod or glmerMod)
- list: a list of the names of the coefficients to be extracted (e.g. c(“variable1”, “variable1:variable2”))


```
get_coeffs <- function(x,list){(as.data.frame(summary(x)$coefficients)[list,])}
```

# Load Datasets


```
setwd("datafiles")
```


```
The working directory was changed to C:/Users/Liz/Desktop/Hanyu_RAnalyses/datafiles inside a notebook chunk. The working directory will be reset when the chunk is finished running. Use the knitr root.dir option in the setup chunk to change the working directory for notebook chunks.
```


```
pcpt <- read.csv("PCPT.csv")
cstc <-read.csv("CSTC-curve.csv", sep = ",", stringsAsFactors = F) 
dis <- read.csv("Discrimination.csv")
train <- read.csv("Training.csv")
PI <- read.csv("Picture Identification.csv")
PRO <- read.csv("Production_all.csv")
setwd("..")
```

# Individual Aptitude Task 1: Pitch Contour Perception Test (Section 3.2.1)

## Figure (Figure 3)


```
pcpt$condition = factor(pcpt$condition, levels = c("0", "1", "2"), labels = c("LV", "HV", "HVB"))
pcpt$session = factor(pcpt$session, levels = c("1", "2"), labels = c("Pre-test", "Post-test"))
means = with(pcpt, aggregate(accuracy ~ subject + session + condition, FUN = mean))
x <- summarySEwithin(means, measurevar = "accuracy", betweenvars = c("condition"), withinvars = c("session"), idvar = "subject", na.rm = FALSE, conf.interval = .95)
```


```
p1 = ggplot (means, aes(x = condition, y = accuracy, fill = session)) 
p1 = p1 + geom_violin(colour = "black", scale = "count")
p1 = p1 + scale_fill_manual(name = "Test Session", values = c("Pre-test" = "grey", "Post-test" = "white"), labels = c("Pre-test", "Post-test"))
p1 = p1 + scale_x_discrete(labels = c("LV", "HV", "HVB"))
p1 = p1 + geom_errorbar(aes(ymin = accuracy-ci, ymax = accuracy+ci), width = .4, position = position_dodge(.9), data = x)
p1 = p1 + stat_summary(fun.y = mean, geom = "point", shape = 23, size = 2,position = position_dodge(.9))
p1 = p1 +theme_bw()+ labs(x = "Variability Condition", y = "Mean Accuracy")
print(p1)
```

## Statistical Analysis (Section 3.2.1)


```
pcpt = lizContrasts(pcpt, pcpt$condition, "LV")
pcpt_glmer1 = glmer(accuracy ~ 
              session * (LV_VERSUS_HV + LV_VERSUS_HVB)
              + (session|subject),
              family = "binomial", 
              control = glmerControl(optimizer = "bobyqa"), 
              data = pcpt)
```


```
kable (round(summary(pcpt_glmer1)$coefficients, 6), digits = 3)
```


|  | Estimate | Std. Error | z value | Pr(>|z|) |
| --- | --- | --- | --- | --- |
| (Intercept) | -0.324 | 0.104 | -3.098 | 0.002 |
| sessionPost-test | 0.209 | 0.051 | 4.134 | 0.000 |
| LV\_VERSUS\_HV | -0.353 | 0.255 | -1.380 | 0.168 |
| LV\_VERSUS\_HVB | 0.169 | 0.256 | 0.662 | 0.508 |
| sessionPost-test:LV\_VERSUS\_HV | -0.051 | 0.124 | -0.412 | 0.680 |
| sessionPost-test:LV\_VERSUS\_HVB | 0.106 | 0.124 | 0.853 | 0.394 |


```
datapcpt <- summarySEwithin(means, measurevar = "accuracy", withinvars = c("session"), idvar = "subject", na.rm = FALSE, conf.interval = .95)
kable(datapcpt, digits = 3)
```


| session | N | accuracy | accuracy\_norm | sd | se | ci |
| --- | --- | --- | --- | --- | --- | --- |
| Post-test | 60 | 0.466 | 0.466 | 0.056 | 0.007 | 0.014 |
| Pre-test | 60 | 0.424 | 0.424 | 0.056 | 0.007 | 0.014 |

# Individual Aptitude Task 2: Categorisation of Synthesized Tonal Continua (Section 3.2.2)

We analysed data from the CSTC task adapted from Sadakata and McQueen (2014). We first attempted to follow the procedures in their paper. Specifically, to quantify performance in this task, each subjects’ categorization curve was fitted to a logistic function using the Logistic Curve Fit function in SPSS and a slope coefficient was calculated (Joanisse, Manis, Keating, & Seidenberg, 2000) which was taken to indicate the participants ability to categorize the two tones, with smaller slopes indicating better performance (perfect slope = 0.042). Sadakata and McQueen (2014), report that they removed any participant with a slope measure greater than 1.2 from the analysis, suggesting that slopes above this threshold were considered to be poorly fit. However following this process with our participants, the majority were above this threshold (43 out of 60). Given this, we attempted an alternative method of deriving slope coefficients for each participant using a logistic mixed effect model (Schultz, Llanos, & Francis, 2003) with the predicted variable being which of two tones the participants chose on each trial and the predictor being the tone step presented on each trial (varying from 1-6 where 1 is most like Tone 2, and 6 is most like Tone 3). Random intercepts and slopes for tone step were fit by participant and the individual slope coefficients for each participant were extracted from the by-participant random slopes fit in the model. Because the random slopes represent adjustments to the fixed effect slope, more positive slopes represent sharper categorization responses, i.e. more sensitivity to differences in tone step, while more negative slopes represent flatter categorization responses, or in extreme cases reversed responses, and slopes close to 0 reflect responses close to the mean. These slopes could thus be taken to be indicators of individual differences. When we compared these slopes to the ones derived from SPSS we found that they were very similar. The same participants who failed the criteria of Sadakata and McQueen also had very shallow slopes using the logistic regression method. The analyses conducted in this script were done with the individual difference measure extracted from the logistic regression.

This figure shows the individual curves for each group. It also shows the raw data that the slopes are based on.


```
cstc$session = factor(cstc$session, levels = c("1","2"), labels = c("Pre-test", "Post-test"))
cstc$condition = factor(cstc$condition, levels = c("0", "1", "2"), labels = c("LV", "HV", "HVB"))
# Two participants showed the completely reversed pattern. Here we reverse the coding of these 2 participants
cstc$category[cstc$subject == "5"|cstc$subject == "36"] <- abs(cstc$category[cstc$subject == "5"|cstc$subject == "36"] - 1)
ggplot(cstc, aes(x = step, y = category)) + 
  stat_smooth(aes(group = subject), method = "glm", method.args = list(family = "binomial"), size = 0.5, se = FALSE, show.legend = FALSE, color = "#cce2ef") + # individual regression lines
  stat_smooth(method = "glm", method.args = list(family = "binomial"), size = 2, se = FALSE, show.legend = FALSE, color = "#B01326") + # group mean regression line
  ylim(0, 1) + scale_x_continuous(breaks = 1:7) + facet_grid(~condition) +
  xlab("tone step") + ylab("Proportion Tone X responses") + theme_bw() +
  theme(plot.title = element_text(hjust = 0.5, face = "bold"))
```


## Extract slope coffcient for each participant and descriptives


```
cstc$session <- as.numeric(cstc$session)
cstc$session.std <- scale(cstc$session)
cstc$step.std <- scale(cstc$step)
f1 <-  factor(c("HV", "LV"))
df1 <- data.frame(f1, subject = 1:40)
f1 <-  factor("HVB")
df2 <- data.frame(f1, subject = 41:60)
df3 <- rbind.fill(df1, df2)
lmfits_pre <- glmer(category ~ step.std + (step.std|subject), data = cstc[cstc$session == 1,], family = "binomial", control = glmerControl(optimizer = "bobyqa"))
pre = ranef(lmfits_pre)$subject
pre = cbind (pre, df3)
pre$session = "Pre-test"
lmfits_post <- glmer(category ~ step.std + (step.std|subject), data = cstc[cstc$session == 2,], family = "binomial", control = glmerControl(optimizer = "bobyqa"))
post = ranef(lmfits_post)$subject
post = cbind (post, df3)
post$session = "Post-test"
cstc2 <- rbind(pre,post)
names(cstc2)[names(cstc2) == "step.std"] <- "slope"
names(cstc2)[names(cstc2) == "(Intercept)"] <- "intercept"
names(cstc2)[names(cstc2) == "f1"] <- "condition"
# reorder the levels so that they match previous data set
cstc2$condition = factor (cstc2$condition, levels =  c("LV","HV","HVB") )
cstc2$session = factor (cstc2$session, levels =  c("Pre-test","Post-test") )
means = with(cstc2, aggregate(slope ~ subject + session + condition, FUN = mean))
x <- summarySEwithin(cstc2, measurevar = "slope", betweenvars = c("condition"), withinvars = c("session"), idvar = "subject", na.rm = FALSE, conf.interval = .95)
kable(x, digits = 2)
```


| condition | session | N | slope | slope\_norm | sd | se | ci |
| --- | --- | --- | --- | --- | --- | --- | --- |
| HV | Post-test | 20 | -0.17 | -0.12 | 0.60 | 0.13 | 0.28 |
| HV | Pre-test | 20 | 0.02 | 0.07 | 0.60 | 0.13 | 0.28 |
| HVB | Post-test | 20 | -0.77 | -0.15 | 0.58 | 0.13 | 0.27 |
| HVB | Pre-test | 20 | -0.53 | 0.10 | 0.58 | 0.13 | 0.27 |
| LV | Post-test | 20 | 0.86 | 0.19 | 0.46 | 0.10 | 0.21 |
| LV | Pre-test | 20 | 0.44 | -0.24 | 0.46 | 0.10 | 0.21 |

## Graph


```
p2 = ggplot (means, aes(x = condition, y = slope, fill = session)) 
p2 = p2 + geom_violin(colour = "black", scale = "count")
p2 = p2 + scale_fill_manual(name = "Test session", values = c("Pre-test" = "grey", "Post-test" = "white"))
p2 = p2 + scale_x_discrete(labels = c("Low Variability", "High Variability", "High Varaibility Blocking"))
p2 = p2 + geom_errorbar(aes(ymin = slope-ci, ymax = slope+ci), width = .4, position = position_dodge(.9), data = x)
p2 = p2 + stat_summary(fun.y = mean, geom = "point", shape = 23, size = 2, position = position_dodge(.9))
p2 = p2 + theme_bw()
print(p2)
```


```
rm(means)
rm(x)
```

## Statistical Analysis


```
cstc2 = lizContrasts(cstc2, cstc2$condition, "LV")
cstc_lm = lm(slope ~ session * (LV_VERSUS_HV + LV_VERSUS_HVB) , data = cstc2)
```


Look at differences between conditions at pre-test:


```
round (get_coeffs(cstc_lm,c("LV_VERSUS_HV","LV_VERSUS_HVB")),3)
```

# Look at the relationship between PCPT and CSTC and set up ID\_pre table


```
ID_pre = merge(
  
  with(subset(pcpt, session %in% "Pre-test"), aggregate(accuracy ~ subject + session + condition, FUN = mean)),
  
  with(subset(cstc2, session %in% "Pre-test"), aggregate(slope ~ subject + session + condition, FUN = mean)), by = "subject")
ID_pre$aptitude_pcpt = ID_pre$accuracy *10
ID_pre$session = NULL
cor.test(ID_pre$slope, ID_pre$accuracy)
```


```
    Pearson's product-moment correlation

data:  ID_pre$slope and ID_pre$accuracy
t = 2.9688, df = 58, p-value = 0.004341
alternative hypothesis: true correlation is not equal to 0
95 percent confidence interval:
 0.1203742 0.5650138
sample estimates:
      cor 
0.3631974
```


## Correlation Figure


```
ggplot(ID_pre, aes(x = accuracy, y = slope, color = condition.x)) +
geom_point(size = 5)
```

## Create a median split (this is used later in plotting, but not in analyses)


```
ID_pre$aptitude2 = 1
ID_pre$aptitude2[ID_pre$accuracy < median(ID_pre$accuracy)] = 0
ID_pre$aptitude3 = 1
ID_pre$aptitude3[ID_pre$slope < median(ID_pre$slope)] = 0
ID_pre$aptitude2 = factor(ID_pre$aptitude2, levels = c("1", "0"), labels = c ("HA", "LA"))
ID_pre$aptitude3 = factor(ID_pre$aptitude3, levels = c("0", "1"), labels = c ("HA", "LA"))
```

# Production Reliability Analysis (Section 3.5.1.1)

## Checking the accuracy of ratings of production data by native speakers


```
#Remove participant 48 
PRO = PRO[!(PRO$subject=="48"),]
PRO$session = factor(PRO$session, levels = c("pretest", "posttest", "nativespeaker", "picturenaming"), labels = c("Pre-test", "Post-test","Native-Speaker", "Picture-Naming"))
PC = subset(PRO, session %in% "Native-Speaker")
means = mean(PC$tone_score)
means2 = mean(PC$pinyin_score)
means3 = mean(PC$rating)
means4 = mean(PC$tone_score_rater1)
means5 = mean(PC$pinyin_score_rater1)
means6 = mean(PC$rating_rater1)
Rater2 = c(means, means2, means3)
Rater1 = c(means4, means5, means6)
Task = factor (c("Tone accuracy","Pinyin accuracy","Tone rating"))
Table = data.frame ("Task" = Task, "Rater 1" = Rater1, "Rater 2" = Rater2)
Table
```


```
rm(means, means2, means3, means4, means5, means6, Rater2, Rater1, Task)
```

## Checking the correlation between Rater1 and Rater2

These correlations are reported for ratings of the experimental data


```
CWR = subset(PRO, session %in% c("Pre-test", "Post-test"))
CPN = subset(PRO, session %in% "Picture-Naming")
cor1 = Kappa.test(CWR$tone_score, CWR$tone_score_rater1, conf.level = 0.95) 
cor2 = Kappa.test(CWR$pinyin_score, CWR$pinyin_score_rater1, conf.level = 0.95) 
cor3 = icc(cbind(CWR$rating, CWR$rating_rater1), "twoway", "consistency")
cor4 = Kappa.test(CPN$tone_score, CPN$tone_score_rater1, conf.level = 0.95)
cor5 = Kappa.test(CPN$pinyin_score, CPN$pinyin_score_rater1, conf.level = 0.95)
cor6 = icc(cbind(CPN$rating, CPN$rating_rater1), "twoway", "consistency")
```


### Rater accuracy of tone identification in Word Repetition


```
cor1
```


```
$Result

    Estimate Cohen's kappa statistics and test the null hypothesis that the extent of agreement is same as random (kappa=0)

data:  CWR$tone_score and CWR$tone_score_rater1
Z = 25.477, p-value < 2.2e-16
95 percent confidence interval:
 0.3682339 0.4197987
sample estimates:
[1] 0.3940163


$Judgement
[1] "Fair agreement"
```

### Rater accuracy of pinyin identification in Word Repetition


```
cor2
```


```
$Result

    Estimate Cohen's kappa statistics and test the null hypothesis that the extent of agreement is same as random (kappa=0)

data:  CWR$pinyin_score and CWR$pinyin_score_rater1
Z = 28.691, p-value < 2.2e-16
95 percent confidence interval:
 0.3105457 0.3526499
sample estimates:
[1] 0.3315978


$Judgement
[1] "Fair agreement"
```

### Rater accuracy of tone rating in Word Repetition


```
cor3
```


```
 Single Score Intraclass Correlation

   Model: twoway 
   Type : consistency 

   Subjects = 8496 
     Raters = 2 
   ICC(C,1) = 0.22

 F-Test, H0: r0 = 0 ; H1: r0 > 0 
F(8495,8495) = 1.56 , p = 5.07e-94 

 95%-Confidence Interval for ICC Population Values:
  0.2 < ICC < 0.24
```

### Rater accuracy of tone identification in Picture Naming


```
cor4
```


```
$Result

    Estimate Cohen's kappa statistics and test the null hypothesis that the extent of agreement is same as random (kappa=0)

data:  CPN$tone_score and CPN$tone_score_rater1
Z = 31.149, p-value < 2.2e-16
95 percent confidence interval:
 0.6408330 0.7035999
sample estimates:
[1] 0.6722164


$Judgement
[1] "Substantial agreement"
```

### Rater accuracy of pinyin identification in Picture Naming


```
cor5
```


```
$Result

    Estimate Cohen's kappa statistics and test the null hypothesis that the extent of agreement is same as random (kappa=0)

data:  CPN$pinyin_score and CPN$pinyin_score_rater1
Z = 25.434, p-value < 2.2e-16
95 percent confidence interval:
 0.4969453 0.5672871
sample estimates:
[1] 0.5321162


$Judgement
[1] "Moderate agreement"
```

### Rater accuracy of tone rating in Picture Naming


```
cor6
```


```
 Single Score Intraclass Correlation

   Model: twoway 
   Type : consistency 

   Subjects = 2124 
     Raters = 2 
   ICC(C,1) = 0.372

 F-Test, H0: r0 = 0 ; H1: r0 > 0 
F(2123,2123) = 2.18 , p = 5.14e-71 

 95%-Confidence Interval for ICC Population Values:
  0.335 < ICC < 0.408
```

# Word Repetition (Section 3.5.1)

## Word Repetition - Tone accuracy (Section 3.5.1.2)

### Label Parameters and remove unidentifiable trials


```
NWR = CWR[CWR$tone != 0, ]
NWR$condition = factor(NWR$condition, levels = c("0", "1","2"), labels = c("LV", "HV", "HVB"))
NWR$wordtype = factor(NWR$wordtype, levels = c("trained", "untrained"), labels = c("Trained Item", "Untrained Item"))
NWR2 = merge(NWR, ID_pre)
```

### Figures


```
means = with(NWR2, aggregate(tone_score ~ subject + session + condition + wordtype, FUN = mean))
meansb = with(NWR2, aggregate(tone_score ~ subject + session + condition + wordtype + aptitude2, FUN = mean))
means2b = with(NWR2, aggregate(tone_score ~ subject + session + condition + wordtype + aptitude3, FUN = mean))
xb <- summarySEwithin(means, measurevar = "tone_score", betweenvars = c("condition"), withinvars = c("wordtype", "session"), idvar = "subject", na.rm = FALSE, conf.interval = .95)
graphdatab <- summarySEwithin(meansb, measurevar = "tone_score", betweenvars = c("condition", "aptitude2"), withinvars = c("session", "wordtype"), idvar = "subject", na.rm = FALSE, conf.interval = .95)
graphdata2b <- summarySEwithin(means2b, measurevar = "tone_score", betweenvars = c("condition", "aptitude3"), withinvars = c("session", "wordtype"), idvar = "subject", na.rm = FALSE, conf.interval = .95)
```


#### Main Graph (Figure 7)


```
WRp1 = ggplot (means, aes(x = condition, y = tone_score, fill = session)) 
WRp1 = WRp1 + facet_wrap(~wordtype, ncol = 2)
WRp1 = WRp1 + geom_violin(colour = "black", scale = "count")
WRp1 = WRp1 + scale_fill_manual(name = "Test Session", values = c("Pre-test" = "grey", "Post-test" = "white"), labels = c("Pre-test", "Post-test"))
WRp1 = WRp1 + scale_x_discrete(labels = c("LV", "HV", "HVB"))
WRp1 = WRp1 + geom_errorbar(aes(ymin = tone_score-ci, ymax = tone_score+ci), width = .4, position = position_dodge(.9), data = xb)
WRp1 = WRp1 + stat_summary(fun.y = mean, geom = "point", shape = 23, size = 2,position = position_dodge(.9))
WRp1 = WRp1 +theme_bw()+ ggtitle('Word Repetition: Tone accuracy')
WRp1 = WRp1 + labs(x = "Variability Condition", y = "Mean Accuracy")
print(WRp1)
```

#### Graph (Figure 12, Left panel)


```
WRp2 = ggplot (meansb, aes(x = condition, y = tone_score, fill = session)) 
WRp2 = WRp2 + facet_wrap(~wordtype + aptitude2, ncol = 2)
WRp2 = WRp2 + geom_violin(colour = "black", scale = "count")
WRp2 = WRp2 + scale_fill_manual(name = "Test Session", values = c("Pre-test" = "grey", "Post-test" = "white"), labels = c("Pre-test", "Post-test"))
WRp2 = WRp2 + scale_x_discrete(labels = c("LV", "HV", "HVB"))
WRp2 = WRp2 + geom_errorbar(aes(ymin = tone_score-ci, ymax = tone_score+ci), width = .4, position = position_dodge(.9), data = graphdatab)
WRp2 = WRp2 + stat_summary(fun.y = mean, geom = "point", shape = 23, size = 2,position = position_dodge(.9))
WRp2 = WRp2 + theme_bw()+ ggtitle('Word Repetition: Tone accuracy')
WRp2 = WRp2 + labs(x = "Variability Condition", y = "Mean Accuracy")
WRp2.m = WRp2 + theme(legend.position = "NULL")
print(WRp2)
```

#### CSTC Graph (Not used)


```
WRp3 = ggplot (means2b, aes(x = condition, y = tone_score, fill = session)) 
WRp3 = WRp3 + facet_wrap(~wordtype + aptitude3, ncol = 2)
WRp3 = WRp3 + geom_violin(colour = "black", scale = "count")
WRp3 = WRp3 + scale_fill_manual(name = "Test Session", values = c("Pre-test" = "grey", "Post-test" = "white"), labels = c("Pre-test", "Post-test"))
WRp3 = WRp3 + scale_x_discrete(labels = c("LV", "HV", "HVB"))
WRp3 = WRp3 + geom_errorbar(aes(ymin = tone_score-ci, ymax = tone_score+ci), width = .4, position = position_dodge(.9), data = graphdata2b)
WRp3 = WRp3 + stat_summary(fun.y = mean, geom = "point", shape = 23, size = 2,position = position_dodge(.9))
WRp3 = WRp3 + theme_bw()+ ggtitle('Word Repetition: Tone accuracy')
WRp3 = WRp3 + labs(x = "Variability Condition", y = "Mean Accuracy")
print(WRp3)
```

### Statistical Analysis

#### Main Model (without aptitude predictors; Section 3.5.1.2)


```
NWR2 = lizCenter(NWR2, list("wordtype", "condition"))
NWR2 = lizContrasts(NWR2, NWR2$condition, "LV")
nw_glmer = glmer(tone_score ~ wordtype.ct * (LV_VERSUS_HV + LV_VERSUS_HVB) * session
              + (wordtype.ct * session||subject),
              family = "binomial", 
              control = glmerControl(optimizer = "bobyqa"), 
              data = NWR2)
```


```
singular fit
```


##### Print out effects in pre-test


```
kable(get_coeffs(nw_glmer, c("wordtype.ct", "LV_VERSUS_HV", "LV_VERSUS_HVB" )), digits = 3)
```


|  | Estimate | Std. Error | z value | Pr(>|z|) |
| --- | --- | --- | --- | --- |
| wordtype.ct | -0.018 | 0.070 | -0.258 | 0.796 |
| LV\_VERSUS\_HV | 0.011 | 0.175 | 0.063 | 0.950 |
| LV\_VERSUS\_HVB | 0.113 | 0.177 | 0.635 | 0.526 |

##### Print out key effects and relevant descriptive statistics of test-session and interactions with test-session (i.e. looking effects of of training)


```
kable(get_coeffs(nw_glmer, c("sessionPost-test",
                            "wordtype.ct:sessionPost-test",
                            "LV_VERSUS_HV:sessionPost-test",
                            "LV_VERSUS_HVB:sessionPost-test",
                            "wordtype.ct:LV_VERSUS_HV:sessionPost-test",
                            "wordtype.ct:LV_VERSUS_HVB:sessionPost-test")), digits = 3)
```


|  | Estimate | Std. Error | z value | Pr(>|z|) |
| --- | --- | --- | --- | --- |
| sessionPost-test | 0.395 | 0.075 | 5.287 | 0.000 |
| wordtype.ct:sessionPost-test | 0.127 | 0.104 | 1.221 | 0.222 |
| LV\_VERSUS\_HV:sessionPost-test | -0.100 | 0.180 | -0.552 | 0.581 |
| LV\_VERSUS\_HVB:sessionPost-test | -0.113 | 0.182 | -0.620 | 0.535 |
| wordtype.ct:LV\_VERSUS\_HV:sessionPost-test | 0.154 | 0.253 | 0.609 | 0.543 |
| wordtype.ct:LV\_VERSUS\_HVB:sessionPost-test | -0.310 | 0.256 | -1.210 | 0.226 |


```
dataWRtone<- summarySEwithin(means, measurevar = "tone_score", withinvars = c("session"), idvar = "subject", na.rm = FALSE, conf.interval = .95)
kable (dataWRtone, digits = 3)
```


| session | N | tone\_score | tone\_score\_norm | sd | se | ci |
| --- | --- | --- | --- | --- | --- | --- |
| Post-test | 118 | 0.785 | 0.785 | 0.085 | 0.008 | 0.016 |
| Pre-test | 118 | 0.713 | 0.713 | 0.087 | 0.008 | 0.016 |

#### Analysis with PCPT accuracy (Section 3.7)


```
NWR2 = lizCenter(NWR2, list("session", "aptitude_pcpt", "slope", "wordtype"))
nw_glmer_pcpt = glmer(tone_score ~ wordtype.ct * (LV_VERSUS_HV + LV_VERSUS_HVB) * session.ct 
              + aptitude_pcpt.ct
              + aptitude_pcpt.ct : session.ct
              + aptitude_pcpt.ct : (LV_VERSUS_HV + LV_VERSUS_HVB) : session.ct 
              + aptitude_pcpt.ct : (LV_VERSUS_HV + LV_VERSUS_HVB) : session.ct : wordtype.ct
              + (wordtype.ct * session.ct||subject)
              , family="binomial" , 
              control = glmerControl(optimizer = "bobyqa"), 
              data = NWR2)
```


```
singular fit
```


##### Check that we get the same pattern of results for the experimentally manipulated variables as in the previous model


```
kable(get_coeffs(nw_glmer_pcpt, c("session.ct",
                            "wordtype.ct:session.ct",
                            "LV_VERSUS_HV:session.ct",
                            "LV_VERSUS_HVB:session.ct",
                            "wordtype.ct:LV_VERSUS_HV:session.ct",
                            "wordtype.ct:LV_VERSUS_HVB:session.ct" )), digits = 3)
```


|  | Estimate | Std. Error | z value | Pr(>|z|) |
| --- | --- | --- | --- | --- |
| session.ct | 0.401 | 0.077 | 5.186 | 0.000 |
| wordtype.ct:session.ct | 0.089 | 0.109 | 0.816 | 0.415 |
| LV\_VERSUS\_HV:session.ct | -0.089 | 0.189 | -0.470 | 0.638 |
| LV\_VERSUS\_HVB:session.ct | -0.155 | 0.184 | -0.843 | 0.399 |
| wordtype.ct:LV\_VERSUS\_HV:session.ct | 0.076 | 0.261 | 0.289 | 0.772 |
| wordtype.ct:LV\_VERSUS\_HVB:session.ct | -0.340 | 0.258 | -1.319 | 0.187 |

##### Print out the effect of individual aptitude and key interactions with aptitude


```
kable(get_coeffs(nw_glmer_pcpt, c("aptitude_pcpt.ct",
                             "session.ct:aptitude_pcpt.ct",
                             "LV_VERSUS_HV:session.ct:aptitude_pcpt.ct",
                             "LV_VERSUS_HVB:session.ct:aptitude_pcpt.ct",
                             "wordtype.ct:LV_VERSUS_HV:session.ct:aptitude_pcpt.ct",
                             "wordtype.ct:LV_VERSUS_HVB:session.ct:aptitude_pcpt.ct")), digits = 3)
```


|  | Estimate | Std. Error | z value | Pr(>|z|) |
| --- | --- | --- | --- | --- |
| aptitude\_pcpt.ct | 0.071 | 0.030 | 2.350 | 0.019 |
| session.ct:aptitude\_pcpt.ct | 0.032 | 0.044 | 0.718 | 0.473 |
| LV\_VERSUS\_HV:session.ct:aptitude\_pcpt.ct | 0.051 | 0.109 | 0.469 | 0.639 |
| LV\_VERSUS\_HVB:session.ct:aptitude\_pcpt.ct | 0.129 | 0.095 | 1.352 | 0.176 |
| wordtype.ct:LV\_VERSUS\_HV:session.ct:aptitude\_pcpt.ct | -0.143 | 0.148 | -0.966 | 0.334 |
| wordtype.ct:LV\_VERSUS\_HVB:session.ct:aptitude\_pcpt.ct | 0.067 | 0.133 | 0.504 | 0.614 |

#### Analysis with CSTC slope (Section 3.7)


```
#Model did not converge so remove slope.ct : (LV_VERSUS_HV + LV_VERSUS_HVB) : session.ct : wordtype.ct
nw_glmer_cstc = glmer(tone_score ~ wordtype.ct * (LV_VERSUS_HV + LV_VERSUS_HVB) * session.ct 
              + slope.ct
              + slope.ct : session.ct
              + slope.ct : (LV_VERSUS_HV + LV_VERSUS_HVB) : session.ct 
             + (wordtype.ct * session.ct|subject)
              , family = "binomial" , 
              control = glmerControl(optimizer = "bobyqa", optCtrl=list(maxfun=1e6)), 
              data = NWR2)
```


```
singular fit
```


##### Check that get the same pattern of results for experimentally manipulated variables as in previous model


```
kable(get_coeffs(nw_glmer_cstc, c("session.ct", "wordtype.ct:session.ct", "wordtype.ct:LV_VERSUS_HV:session.ct", "wordtype.ct:LV_VERSUS_HVB:session.ct")), digits = 3)
```


|  | Estimate | Std. Error | z value | Pr(>|z|) |
| --- | --- | --- | --- | --- |
| session.ct | 0.336 | 0.078 | 4.327 | 0.000 |
| wordtype.ct:session.ct | 0.119 | 0.106 | 1.123 | 0.262 |
| wordtype.ct:LV\_VERSUS\_HV:session.ct | 0.147 | 0.254 | 0.580 | 0.562 |
| wordtype.ct:LV\_VERSUS\_HVB:session.ct | -0.307 | 0.257 | -1.194 | 0.233 |

##### Print out the effect of individual aptitude and key interactions with aptitude


```
kable(get_coeffs(nw_glmer_cstc, c("slope.ct", "session.ct:slope.ct", "LV_VERSUS_HV:session.ct:slope.ct","LV_VERSUS_HVB:session.ct:slope.ct")), digits = 3)
```


|  | Estimate | Std. Error | z value | Pr(>|z|) |
| --- | --- | --- | --- | --- |
| slope.ct | 0.010 | 0.055 | 0.177 | 0.859 |
| session.ct:slope.ct | -0.110 | 0.075 | -1.474 | 0.140 |
| LV\_VERSUS\_HV:session.ct:slope.ct | -0.232 | 0.164 | -1.420 | 0.156 |
| LV\_VERSUS\_HVB:session.ct:slope.ct | -0.357 | 0.177 | -2.021 | 0.043 |

## Word Repetition - Pinyin Accuracy (Section 3.5.1.3)

### Figures


```
means = with(NWR2, aggregate(pinyin_score ~ subject + session + condition + wordtype, FUN = mean))
meansb = with(NWR2, aggregate(pinyin_score ~ subject + session + condition + wordtype + aptitude2, FUN = mean))
means2b = with(NWR2, aggregate(pinyin_score ~ subject + session + condition + wordtype + aptitude3, FUN = mean))
xb <- summarySEwithin(means, measurevar = "pinyin_score", betweenvars = c("condition"), withinvars = c("wordtype", "session"), idvar = "subject", na.rm = FALSE, conf.interval = .95)
graphdatab <- summarySEwithin(meansb, measurevar = "pinyin_score", betweenvars = c("condition", "aptitude2"), withinvars = c("session", "wordtype"), idvar = "subject", na.rm = FALSE, conf.interval = .95)
graphdata2b <- summarySEwithin(means2b, measurevar = "pinyin_score", betweenvars = c("condition", "aptitude3"), withinvars = c("session", "wordtype"), idvar = "subject", na.rm = FALSE, conf.interval = .95)
```


#### Main Graph (Figure 8)


```
WRp9 = ggplot (means, aes(x = condition, y = pinyin_score, fill = session)) 
WRp9 = WRp9 + facet_wrap(~wordtype, ncol = 2)
WRp9 = WRp9 + geom_violin(colour = "black", scale = "count")
WRp9 = WRp9 + scale_fill_manual(name = "Test Session", values = c("Pre-test" = "grey", "Post-test" = "white"), labels = c("Pre-test", "Post-test"))
WRp9 = WRp9 + scale_x_discrete(labels = c("LV", "HV", "HVB"))
WRp9 = WRp9 + geom_errorbar(aes(ymin = pinyin_score-ci, ymax = pinyin_score+ci), width = .4, position = position_dodge(.9), data = xb)
WRp9 = WRp9 + stat_summary(fun.y = mean, geom = "point", shape = 23, size = 2, position = position_dodge(.9))
WRp9 = WRp9 + theme_bw() + ggtitle('Word Repetition: Pinyin accuracy')
WRp9 = WRp9 + labs(x = "Variability Condition", y = "Mean Accuracy")
print(WRp9)
```

#### PCPT Graph (Figure 12, Right panel)


```
WRp10 = ggplot (meansb, aes(x = condition, y = pinyin_score, fill = session)) 
WRp10 = WRp10 + facet_wrap(~wordtype + aptitude2, ncol = 2)
WRp10 = WRp10 + geom_violin(colour = "black", scale = "count")
WRp10 = WRp10 + scale_fill_manual(name = "Test Session", values = c("Pre-test" = "grey", "Post-test" = "white"), labels = c("Pre-test", "Post-test"))
WRp10 = WRp10 + scale_x_discrete(labels = c("LV", "HV", "HVB"))
WRp10 = WRp10 + geom_errorbar(aes(ymin = pinyin_score-ci, ymax = pinyin_score+ci), width = .4, position = position_dodge(.9), data = graphdatab)
WRp10 = WRp10 + stat_summary(fun.y = mean, geom = "point", shape = 23, size = 2,position = position_dodge(.9))
WRp10 = WRp10 + theme_bw()+ ggtitle('Word Repetition: Pinyin accuracy')
WRp10 = WRp10 + labs(x = "Variability Condition", y = "Mean Accuracy")
print(WRp10)
```

#### CSTC Graph (Not used)


```
WRp11 = ggplot (means2b, aes(x = condition, y = pinyin_score, fill = session)) 
WRp11 = WRp11 + facet_wrap(~wordtype + aptitude3, ncol = 2)
WRp11 = WRp11 + geom_violin(colour = "black", scale = "count")
WRp11 = WRp11 + scale_fill_manual(name = "Test Session", values = c("Pre-test" = "grey", "Post-test" = "white"), labels = c("Pre-test", "Post-test"))
WRp11 = WRp11 + scale_x_discrete(labels = c("LV", "HV", "HVB"))
WRp11 = WRp11 + geom_errorbar(aes(ymin = pinyin_score-ci, ymax = pinyin_score+ci), width = .4, position = position_dodge(.9), data = graphdata2b)
WRp11 = WRp11 + stat_summary(fun.y = mean, geom = "point", shape = 23, size = 2,position = position_dodge(.9))
WRp11 = WRp11 + theme_bw()+ ggtitle('Word Repetition: Pinyin accuracy')
WRp11 = WRp11 + labs(x = "Variability Condition", y = "Mean Accuracy")
print(WRp11)
```

### Statistical Analysis

#### Main Model (without aptitude predictors; Section 3.5.1.3)


```
NWR2 = lizCenter(NWR2, list("wordtype"))
NWR2 = lizContrasts(NWR2, NWR2$condition, "LV")
nw_glmer = glmer(pinyin_score ~ wordtype.ct * (LV_VERSUS_HV + LV_VERSUS_HVB) * session
              + (wordtype.ct * session||subject)
              , family="binomial", 
              control = glmerControl(optimizer = "bobyqa"), 
              data = NWR2)
```


```
singular fit
```


##### Print out effects at pre-test


```
## possibly relevant at pre test 
kable(get_coeffs(nw_glmer, c("wordtype.ct", "LV_VERSUS_HV", "LV_VERSUS_HVB")), digits = 3)
```


|  | Estimate | Std. Error | z value | Pr(>|z|) |
| --- | --- | --- | --- | --- |
| wordtype.ct | 0.205 | 0.066 | 3.105 | 0.002 |
| LV\_VERSUS\_HV | -0.012 | 0.108 | -0.110 | 0.913 |
| LV\_VERSUS\_HVB | -0.026 | 0.109 | -0.236 | 0.813 |

##### Print out key effects of relevant descriptive statistics test-session and interactions with test-session (i.e. looking effects of training)"


```
kable(get_coeffs(nw_glmer, c("sessionPost-test", "wordtype.ct:sessionPost-test", "LV_VERSUS_HV:sessionPost-test", "LV_VERSUS_HVB:sessionPost-test", "wordtype.ct:LV_VERSUS_HV:sessionPost-test", "wordtype.ct:LV_VERSUS_HVB:sessionPost-test")), digits = 3)
```


|  | Estimate | Std. Error | z value | Pr(>|z|) |
| --- | --- | --- | --- | --- |
| sessionPost-test | 0.153 | 0.045 | 3.378 | 0.001 |
| wordtype.ct:sessionPost-test | 0.056 | 0.090 | 0.621 | 0.535 |
| LV\_VERSUS\_HV:sessionPost-test | 0.051 | 0.110 | 0.459 | 0.646 |
| LV\_VERSUS\_HVB:sessionPost-test | -0.119 | 0.110 | -1.079 | 0.281 |
| wordtype.ct:LV\_VERSUS\_HV:sessionPost-test | 0.113 | 0.219 | 0.514 | 0.607 |
| wordtype.ct:LV\_VERSUS\_HVB:sessionPost-test | -0.140 | 0.219 | -0.637 | 0.524 |


```
dataWRpinyin <- summarySEwithin(means, measurevar = "pinyin_score", withinvars = c("session"), idvar = "subject", na.rm = FALSE, conf.interval = .95)
kable (dataWRpinyin, digits = 3)
```


| session | N | pinyin\_score | pinyin\_score\_norm | sd | se | ci |
| --- | --- | --- | --- | --- | --- | --- |
| Post-test | 118 | 0.577 | 0.577 | 0.088 | 0.008 | 0.016 |
| Pre-test | 118 | 0.544 | 0.544 | 0.091 | 0.008 | 0.017 |

#### Analysis with PCPT accuracy (Section 3.7)


```
NWR2 = lizCenter(NWR2, list("session", "accuracy", "slope"))
nw_glmer_pcpt = glmer(pinyin_score ~ wordtype.ct * (LV_VERSUS_HV + LV_VERSUS_HVB) * session.ct 
              + aptitude_pcpt.ct
              + aptitude_pcpt.ct : session.ct
              + aptitude_pcpt.ct : (LV_VERSUS_HV + LV_VERSUS_HVB) : session.ct 
              + aptitude_pcpt.ct : (LV_VERSUS_HV + LV_VERSUS_HVB) : session.ct : wordtype.ct
              + (wordtype.ct * session.ct||subject)
              , family = "binomial", 
              control = glmerControl(optimizer = "bobyqa"), 
              data = NWR2)
```


```
singular fit
```


##### Check that get the same pattern of results for experimentally manipulated variables as in previous model


```
kable(get_coeffs(nw_glmer_pcpt, c("session.ct",
                            "wordtype.ct:session.ct",
                            "LV_VERSUS_HV:session.ct",
                            "LV_VERSUS_HVB:session.ct",
                            "wordtype.ct:LV_VERSUS_HV:session.ct",
                            "wordtype.ct:LV_VERSUS_HVB:session.ct" )), digits = 3)
```


|  | Estimate | Std. Error | z value | Pr(>|z|) |
| --- | --- | --- | --- | --- |
| session.ct | 0.153 | 0.047 | 3.299 | 0.001 |
| wordtype.ct:session.ct | 0.017 | 0.093 | 0.183 | 0.855 |
| LV\_VERSUS\_HV:session.ct | 0.084 | 0.114 | 0.738 | 0.461 |
| LV\_VERSUS\_HVB:session.ct | -0.142 | 0.111 | -1.282 | 0.200 |
| wordtype.ct:LV\_VERSUS\_HV:session.ct | 0.042 | 0.223 | 0.190 | 0.849 |
| wordtype.ct:LV\_VERSUS\_HVB:session.ct | -0.147 | 0.221 | -0.663 | 0.508 |

##### Print out the effect of individual aptitude and key interactions with aptitude


```
kable(get_coeffs(nw_glmer_pcpt, c("aptitude_pcpt.ct", "session.ct:aptitude_pcpt.ct", "LV_VERSUS_HV:session.ct:aptitude_pcpt.ct", "LV_VERSUS_HVB:session.ct:aptitude_pcpt.ct", "wordtype.ct:LV_VERSUS_HV:session.ct:aptitude_pcpt.ct", "wordtype.ct:LV_VERSUS_HVB:session.ct:aptitude_pcpt.ct")), digits = 3)
```


|  | Estimate | Std. Error | z value | Pr(>|z|) |
| --- | --- | --- | --- | --- |
| aptitude\_pcpt.ct | 0.065 | 0.021 | 3.037 | 0.002 |
| session.ct:aptitude\_pcpt.ct | 0.046 | 0.026 | 1.757 | 0.079 |
| LV\_VERSUS\_HV:session.ct:aptitude\_pcpt.ct | 0.033 | 0.065 | 0.504 | 0.614 |
| LV\_VERSUS\_HVB:session.ct:aptitude\_pcpt.ct | 0.046 | 0.058 | 0.789 | 0.430 |
| wordtype.ct:LV\_VERSUS\_HV:session.ct:aptitude\_pcpt.ct | -0.225 | 0.124 | -1.810 | 0.070 |
| wordtype.ct:LV\_VERSUS\_HVB:session.ct:aptitude\_pcpt.ct | -0.035 | 0.114 | -0.309 | 0.758 |

#### Analysis with CSTC slope


```
nw_glmer_cstc = glmer(pinyin_score ~ wordtype.ct * (LV_VERSUS_HV + LV_VERSUS_HVB) * session.ct 
              + slope.ct
              + slope.ct: aptitude_pcpt.ct
              + slope.ct : (LV_VERSUS_HV + LV_VERSUS_HVB) : session.ct 
              + slope.ct : (LV_VERSUS_HV + LV_VERSUS_HVB) : session.ct : wordtype.ct
              + (wordtype.ct * session.ct|subject)
              , family = "binomial", 
              control = glmerControl(optimizer = "bobyqa"), 
              data = NWR2)
```


```
singular fit
```


##### Check that we get the same pattern of results for the experimentally manipulated variables as in the previous model


```
kable(get_coeffs(nw_glmer_cstc, c("session.ct", "wordtype.ct:session.ct", "wordtype.ct:LV_VERSUS_HV:session.ct", "wordtype.ct:LV_VERSUS_HVB:session.ct")), digits = 3)
```


|  | Estimate | Std. Error | z value | Pr(>|z|) |
| --- | --- | --- | --- | --- |
| session.ct | 0.160 | 0.049 | 3.278 | 0.001 |
| wordtype.ct:session.ct | 0.052 | 0.097 | 0.534 | 0.593 |
| wordtype.ct:LV\_VERSUS\_HV:session.ct | 0.145 | 0.226 | 0.640 | 0.522 |
| wordtype.ct:LV\_VERSUS\_HVB:session.ct | -0.089 | 0.230 | -0.385 | 0.700 |

##### Print out the effect of individual aptitude and key interactions with aptitude


```
kable(get_coeffs(nw_glmer_cstc, c("slope.ct", "slope.ct:aptitude_pcpt.ct", "LV_VERSUS_HV:session.ct:slope.ct", "LV_VERSUS_HVB:session.ct:slope.ct", "wordtype.ct:LV_VERSUS_HV:session.ct:slope.ct", "wordtype.ct:LV_VERSUS_HVB:session.ct:slope.ct")), digits = 3)
```


|  | Estimate | Std. Error | z value | Pr(>|z|) |
| --- | --- | --- | --- | --- |
| slope.ct | 0.004 | 0.040 | 0.108 | 0.914 |
| slope.ct:aptitude\_pcpt.ct | 0.045 | 0.020 | 2.238 | 0.025 |
| LV\_VERSUS\_HV:session.ct:slope.ct | -0.038 | 0.105 | -0.366 | 0.714 |
| LV\_VERSUS\_HVB:session.ct:slope.ct | 0.051 | 0.110 | 0.465 | 0.642 |
| wordtype.ct:LV\_VERSUS\_HV:session.ct:slope.ct | -0.183 | 0.208 | -0.879 | 0.380 |
| wordtype.ct:LV\_VERSUS\_HVB:session.ct:slope.ct | -0.029 | 0.219 | -0.133 | 0.894 |

# Three-interval Oddity Task (Section 3.4.1)

## Label Parameters


```
dis$condition = factor(dis$condition, levels = c("0", "1", "2"), labels = c("LV", "HV", "HVB"))
dis$session = factor(dis$session, levels = c("1", "2"), labels = c("Pre-test", "Post-test"))
dis$trialtype = dis$voicetype = factor(dis$voicetype, levels = c("fff", "ffm", "fmf"), labels = c("Neutral", "Easy", "Hard"))
dis$wordtype = factor(dis$wordtype, levels = c("oldword", "newword"), labels = c("Trained Item", "Untrained Item"))
dis2 = merge(ID_pre, dis)
```

## Figures


```
means = with(dis2, aggregate(score ~ subject + condition + voicetype + wordtype + session, FUN = mean))
meansb = with(dis2, aggregate(score ~ subject + condition + voicetype + wordtype + session + aptitude2+accuracy, FUN = mean))
means2b = with(dis2, aggregate(score ~ subject + condition + voicetype + wordtype + session + aptitude3+slope, FUN = mean))
graphdata <- summarySEwithin(meansb, measurevar = "score", betweenvars = c("condition", "aptitude2"), withinvars = c("wordtype", "session"), idvar = "subject", na.rm = FALSE, conf.interval = .95)
graphdata2 <- summarySEwithin(means2b, measurevar = "score", betweenvars = c("condition", "aptitude3"), withinvars = c("wordtype","session"), idvar = "subject", na.rm = FALSE, conf.interval = .95)
x2 <- summarySEwithin(means, measurevar = "score", betweenvars = c("condition"), withinvars = c("wordtype", "session"), idvar = "subject", na.rm = FALSE, conf.interval = .95)
```


### Main Graph (Figure 5)


```
Dp1 = ggplot (means, aes(x = condition, y = score, fill = session)) 
Dp1 = Dp1 + facet_wrap(~ wordtype, ncol = 2)
Dp1 = Dp1 + geom_violin(colour = "black", scale = "count")
Dp1 = Dp1 + scale_fill_manual(name = "Test Session", values = c("Pre-test" = "grey", "Post-test" = "white"), labels = c("Pre-test", "Post-test"))
Dp1 = Dp1 + scale_x_discrete(labels = c("LV", "HV", "HVB"))
Dp1 = Dp1 + geom_errorbar(aes(ymin = score-ci, ymax = score+ci), width = .4, position = position_dodge(.9), data = x2)
Dp1 = Dp1 + stat_summary(fun.y = mean, geom = "point", shape = 23, size = 2, position = position_dodge(.9))
Dp1 = Dp1 + theme_bw() + ggtitle('Three-interval Oddity')
Dp1 = Dp1 + labs(x = "Variability Condition", y = "Mean Accuracy")
print(Dp1)
```

### PCPT graph (Figure 10, top panel)


```
Dp2 = ggplot (meansb, aes(x = condition, y = score, fill = session))
Dp2 = Dp2 + facet_wrap(~ wordtype + aptitude2, ncol = 2)
Dp2 = Dp2 + geom_violin(colour = "black", scale = "count")
Dp2 = Dp2 + scale_fill_manual(name = "Test Session", values = c("Pre-test" = "grey", "Post-test" = "white"), labels = c("Pre-test", "Post-test"))
Dp2 = Dp2 + scale_x_discrete(labels = c("LV", "HV", "HVB"))
Dp2 = Dp2 + geom_errorbar(aes(ymin = score-ci, ymax = score+ci), width = .4, position = position_dodge(.9), data = graphdata)
Dp2 = Dp2 + stat_summary(fun.y = mean, geom = "point", shape = 23, size = 2, position = position_dodge(.9))
Dp2 = Dp2 + theme_bw()+ ggtitle('Three-interval Oddity')
Dp2 = Dp2 + labs(x = "Variability Condition", y = "Mean Accuracy")
print(Dp2)
```

### CSTC graph (Not included)


```
Dp3 = ggplot (means2b, aes(x = condition, y = score, fill = session)) 
Dp3 = Dp3 + facet_wrap(~ wordtype + aptitude3, ncol = 2)
Dp3 = Dp3 + geom_violin(colour = "black", scale = "count")
Dp3 = Dp3 + scale_fill_manual(name = "Test Session", values = c("Pre-test" = "grey", "Post-test" = "white"), labels = c("Pre-test", "Post-test"))
Dp3 = Dp3 + scale_x_discrete(labels = c("LV", "HV", "HVB"))
Dp3 = Dp3 + geom_errorbar(aes(ymin = score-ci, ymax = score+ci), width = .4, position = position_dodge(.9), data = graphdata2)
Dp3 = Dp3 + stat_summary(fun.y = mean, geom = "point", shape = 23, size = 2, position = position_dodge(.9))
Dp3 = Dp3 + theme_bw() + ggtitle('Three-interval Oddity')
Dp3 = Dp3 + labs(x = "Variability Condition", y = "Mean Accuracy")
print(Dp3)
```

### PCPT Graph (Dot plot, not currently used)


```
means.pre <- subset(meansb, session == "Pre-test")
means.pre = rename(means.pre, c(score = "prescore"))
means.pre$session = NULL
means.post <- subset(meansb, session == "Post-test")
means.post = rename(means.post, c(score ="postscore"))
means.post$session = NULL
means.x = merge(means.pre, means.post)
means.x$diff = means.x$postscore-means.x$prescore
Dp4 = ggplot(means.x, aes(x = accuracy, y = diff))
Dp4=Dp4 + geom_point(aes(shape = factor(condition)))
Dp4 = Dp4 + ggtitle('Three-interval Oddity')
print(Dp4)
```


```
rm(means.pre)
rm(means.post)
rm(means.x)
```

### CSTC Graph (Dot plot, not currently used)


```
means2.pre <- subset(means2b, session == "Pre-test")
means2.pre = rename(means2.pre, c(score = "prescore"))
means2.pre$session = NULL
means2.post <- subset(means2b, session == "Post-test")
means2.post = rename(means2.post, c(score = "postscore"))
means2.post$session = NULL
means2.x = merge(means2.pre, means2.post)
means2.x$diff = means2.x$postscore-means2.x$prescore
Dp5 = ggplot(means2.x, aes(x = slope, y = diff))
Dp5=Dp5 + geom_point(aes(shape = factor(condition)))
Dp5 = Dp5 + ggtitle('Three-interval Oddity')
Dp5
```


```
rm(means2.pre)
rm(means2.post)
rm(means2.x)
```

## Statistical Analysis

### Main model (without aptitude predictors; Section 3.4.1)


```
dis2 = lizCenter(dis2, list("session", "wordtype", "aptitude_pcpt"))
dis2 = lizContrasts(dis2, dis2$condition, "LV")
dis2 = lizContrasts(dis2, dis2$voicetype, "Neutral")
d_glmer = glmer(score ~ session * wordtype.ct * (LV_VERSUS_HV + LV_VERSUS_HVB) 
              + (Neutral_VERSUS_Easy + Neutral_VERSUS_Hard): session
              + (Neutral_VERSUS_Easy + Neutral_VERSUS_Hard) 
              + (session * wordtype.ct||subject)
              , family = "binomial", 
              control = glmerControl(optimizer = "bobyqa"), 
              data = dis2)
```


```
singular fit
```


#### Print out key effects at pre-test session


```
kable(get_coeffs(d_glmer, c("(Intercept)", "wordtype.ct", "LV_VERSUS_HV", "LV_VERSUS_HVB", "Neutral_VERSUS_Easy", "Neutral_VERSUS_Hard")), digits = 3)
```


|  | Estimate | Std. Error | z value | Pr(>|z|) |
| --- | --- | --- | --- | --- |
| (Intercept) | 0.396 | 0.059 | 6.757 | 0.000 |
| wordtype.ct | 0.314 | 0.064 | 4.951 | 0.000 |
| LV\_VERSUS\_HV | -0.002 | 0.143 | -0.011 | 0.991 |
| LV\_VERSUS\_HVB | 0.123 | 0.143 | 0.857 | 0.392 |
| Neutral\_VERSUS\_Easy | 0.400 | 0.079 | 5.091 | 0.000 |
| Neutral\_VERSUS\_Hard | -0.138 | 0.077 | -1.804 | 0.071 |

#### Print out key effects relevant descriptive statistics of test-session and interactions with test-session (i.e. looking effects of training)


```
kable(get_coeffs(d_glmer, c("sessionPost-test", "sessionPost-test:wordtype.ct", "sessionPost-test:LV_VERSUS_HV",  "sessionPost-test:LV_VERSUS_HVB", "sessionPost-test:wordtype.ct:LV_VERSUS_HV", "sessionPost-test:wordtype.ct:LV_VERSUS_HVB")), digits = 3)
```


|  | Estimate | Std. Error | z value | Pr(>|z|) |
| --- | --- | --- | --- | --- |
| sessionPost-test | 0.311 | 0.048 | 6.536 | 0.000 |
| sessionPost-test:wordtype.ct | -0.136 | 0.091 | -1.489 | 0.136 |
| sessionPost-test:LV\_VERSUS\_HV | -0.014 | 0.115 | -0.121 | 0.904 |
| sessionPost-test:LV\_VERSUS\_HVB | 0.013 | 0.116 | 0.114 | 0.909 |
| sessionPost-test:wordtype.ct:LV\_VERSUS\_HV | -0.143 | 0.223 | -0.640 | 0.522 |
| sessionPost-test:wordtype.ct:LV\_VERSUS\_HVB | 0.129 | 0.224 | 0.574 | 0.566 |


```
dataD <- summarySEwithin(means, measurevar = "score", withinvars =  c("session"), idvar = "subject", na.rm = FALSE, conf.interval = .95)
kable (dataD, digits = 3)
```


| session | N | score | score\_norm | sd | se | ci |
| --- | --- | --- | --- | --- | --- | --- |
| Post-test | 360 | 0.661 | 0.661 | 0.193 | 0.010 | 0.020 |
| Pre-test | 360 | 0.592 | 0.592 | 0.210 | 0.011 | 0.022 |

#### Examine if learning is different for the easier/harder trial types and print out relevant descriptive statistics


```
round(get_coeffs(d_glmer, c("sessionPost-test:Neutral_VERSUS_Easy","sessionPost-test:Neutral_VERSUS_Hard")), 3)
```


```
dataD2 <- summarySEwithin(means, measurevar = "score", withinvars =  c("session", "voicetype"), idvar = "subject", na.rm = FALSE, conf.interval = .95)
kable (dataD2, digits = 3)
```


| session | voicetype | N | score | score\_norm | sd | se | ci |
| --- | --- | --- | --- | --- | --- | --- | --- |
| Post-test | Easy | 120 | 0.681 | 0.681 | 0.153 | 0.014 | 0.028 |
| Post-test | Hard | 120 | 0.649 | 0.649 | 0.148 | 0.013 | 0.027 |
| Post-test | Neutral | 120 | 0.653 | 0.653 | 0.145 | 0.013 | 0.026 |
| Pre-test | Easy | 120 | 0.664 | 0.664 | 0.161 | 0.015 | 0.029 |
| Pre-test | Hard | 120 | 0.540 | 0.540 | 0.156 | 0.014 | 0.028 |
| Pre-test | Neutral | 120 | 0.573 | 0.573 | 0.141 | 0.013 | 0.025 |

### Analyses with PCPT accuracy (Section 3.7)


```
dis2 = lizCenter(dis2, list("session.ct"))
d_glmer_pcpt = glmer(score ~ session.ct * wordtype.ct * (LV_VERSUS_HV + LV_VERSUS_HVB) 
              + (Neutral_VERSUS_Easy + Neutral_VERSUS_Hard): session.ct 
              + (Neutral_VERSUS_Easy + Neutral_VERSUS_Hard) 
              + aptitude_pcpt.ct
              + aptitude_pcpt.ct: session.ct
              + aptitude_pcpt.ct : (LV_VERSUS_HV + LV_VERSUS_HVB) : session.ct
              + aptitude_pcpt.ct : (LV_VERSUS_HV + LV_VERSUS_HVB) : session.ct : wordtype.ct
              + (session.ct * wordtype.ct||subject)
              , family = "binomial", 
              control = glmerControl(optimizer = "bobyqa"), 
              data = dis2)
```


```
singular fit
```


#### Print out key effects of test-session and interactions with test-session (i.e. looking effects of training)


```
kable(get_coeffs(d_glmer_pcpt, c("(Intercept)", "session.ct", "session.ct:wordtype.ct", "session.ct:LV_VERSUS_HV", "session.ct:LV_VERSUS_HVB", "session.ct:wordtype.ct:LV_VERSUS_HV",  "session.ct:wordtype.ct:LV_VERSUS_HVB")), digits = 3)
```


|  | Estimate | Std. Error | z value | Pr(>|z|) |
| --- | --- | --- | --- | --- |
| (Intercept) | 0.549 | 0.057 | 9.711 | 0.000 |
| session.ct | 0.304 | 0.048 | 6.366 | 0.000 |
| session.ct:wordtype.ct | -0.173 | 0.095 | -1.824 | 0.068 |
| session.ct:LV\_VERSUS\_HV | -0.005 | 0.117 | -0.042 | 0.967 |
| session.ct:LV\_VERSUS\_HVB | 0.000 | 0.114 | -0.001 | 0.999 |
| session.ct:wordtype.ct:LV\_VERSUS\_HV | -0.205 | 0.228 | -0.899 | 0.369 |
| session.ct:wordtype.ct:LV\_VERSUS\_HVB | 0.095 | 0.227 | 0.418 | 0.676 |

#### Check that we get the same pattern of results for the experimentally manipulated variables as in the previous model


```
get_coeffs(d_glmer_pcpt, c("aptitude_pcpt.ct", "session.ct:aptitude_pcpt.ct", "session.ct:LV_VERSUS_HV:aptitude_pcpt.ct", "session.ct:LV_VERSUS_HVB:aptitude_pcpt.ct", "session.ct:wordtype.ct:LV_VERSUS_HV:aptitude_pcpt.ct", "session.ct:wordtype.ct:LV_VERSUS_HVB:aptitude_pcpt.ct"))
```

### Analyses-with CSTC slope


```
dis2 = lizCenter(dis2, c("slope"))
d_glmer_cstc = glmer(score ~ session.ct * wordtype.ct * (LV_VERSUS_HV + LV_VERSUS_HVB) 
              + (Neutral_VERSUS_Easy + Neutral_VERSUS_Hard): session.ct 
              + (Neutral_VERSUS_Easy + Neutral_VERSUS_Hard) 
              + slope.ct
              + slope.ct: session.ct
              + slope.ct : (LV_VERSUS_HV + LV_VERSUS_HVB) : session.ct
              + slope.ct : (LV_VERSUS_HV + LV_VERSUS_HVB) : session.ct: wordtype.ct
              + (session.ct * wordtype.ct||subject)
              , family = "binomial", 
              control = glmerControl(optimizer = "bobyqa"), 
              data = dis2)
```


```
singular fit
```


#### Print out key effects of test-session and interactions with test-session (i.e. looking effects of training)


```
kable(get_coeffs(d_glmer_cstc, c("(Intercept)", "session.ct", "session.ct:wordtype.ct", "session.ct:LV_VERSUS_HV", "session.ct:LV_VERSUS_HVB", "session.ct:wordtype.ct:LV_VERSUS_HV", "session.ct:wordtype.ct:LV_VERSUS_HVB")), digits = 3)
```


|  | Estimate | Std. Error | z value | Pr(>|z|) |
| --- | --- | --- | --- | --- |
| (Intercept) | 0.549 | 0.058 | 9.481 | 0.000 |
| session.ct | 0.301 | 0.050 | 6.044 | 0.000 |
| session.ct:wordtype.ct | -0.194 | 0.098 | -1.978 | 0.048 |
| session.ct:LV\_VERSUS\_HV | -0.030 | 0.115 | -0.260 | 0.795 |
| session.ct:LV\_VERSUS\_HVB | -0.012 | 0.126 | -0.096 | 0.923 |
| session.ct:wordtype.ct:LV\_VERSUS\_HV | -0.067 | 0.229 | -0.295 | 0.768 |
| session.ct:wordtype.ct:LV\_VERSUS\_HVB | 0.114 | 0.234 | 0.485 | 0.627 |

#### Check that we get the same pattern of results for the experimentally manipulated variables as in the previous model


```
get_coeffs(d_glmer_cstc,  c("slope.ct", "session.ct:slope.ct", "session.ct:LV_VERSUS_HV:slope.ct", "session.ct:LV_VERSUS_HVB:slope.ct",   "session.ct:wordtype.ct:LV_VERSUS_HV:slope.ct", "session.ct:wordtype.ct:LV_VERSUS_HVB:slope.ct"))
```

# Training (Section 3.3)

## Parameters with labels


```
## Note: the ID_train data frame already has aptitude2 and aptitude3 in 
train$condition = factor(train$condition, levels = c("0","2","1"), labels = c("LV", "HVB", "HV"))
train2 = merge(ID_pre, train)
means = with(train2, aggregate(score ~ subject + session + condition + aptitude2, FUN = mean))
means2 = with(train2, aggregate(score ~ subject + session + condition + aptitude3, FUN = mean))
```

## Figures


```
x <- summarySEwithin(means, measurevar = "score", betweenvars = c("condition"), withinvars = c("session"), idvar = "subject", na.rm = FALSE, conf.interval = .95)
```


```
Automatically converting the following non-factors to factors: session
```


```
graphdata <- summarySEwithin(means, measurevar = "score", betweenvars = c("condition", "aptitude2"), withinvars = c("session"), idvar = "subject", na.rm = FALSE, conf.interval =.95)
```


```
Automatically converting the following non-factors to factors: session
```


```
graphdata2 <- summarySEwithin(means2, measurevar = "score", betweenvars = c("condition", "aptitude3"), withinvars = c("session"), idvar = "subject", na.rm = FALSE, conf.interval = .95)
```


```
Automatically converting the following non-factors to factors: session
```


### Main Graph (Figure 4)


```
Tp1 = ggplot(x, aes(x = session, y = score, group = condition, colour = condition)) + geom_line(aes(linetype = condition), size = 0.8) + geom_point(aes(shape = condition), size = 2.5)
Tp1 = Tp1 + geom_errorbar(aes(ymin = score-ci, ymax = score+ci), width = .2)
Tp1 = Tp1 + scale_linetype_discrete(name = "Variability Condition", breaks = c("LV","HV", "HVB"), labels = c("LV", "HV", "HVB")) + scale_shape_discrete(name = "Variability Condition", breaks = c("LV","HV", "HVB"), labels = c("LV", "HV", "HVB")) + scale_colour_grey(name = "Variability Condition", breaks = c("LV","HV", "HVB"), labels = c("LV", "HV", "HVB"))
Tp1 = Tp1 + coord_cartesian(ylim = c(0.5, 1.0))
Tp1 = Tp1 + theme_bw()
Tp1 = Tp1 + ggtitle('Training')
Tp1 = Tp1 + theme(panel.background = element_rect(fill = 'white'), panel.grid.major = element_line(colour = "white"))
Tp1 = Tp1 + labs(x = "Training Session", y = "Mean Accuracy")
print(Tp1)
```

### PCPT Graph (Figure 10, Bottom panel)


```
Tp2 = ggplot(graphdata, aes(x = session, y = score, group = condition, colour = condition)) + geom_line(aes(linetype = condition),size = 0.8) + geom_point(aes(shape = condition), size = 2.5)
Tp2 = Tp2 + facet_wrap(~aptitude2, ncol = 2)
Tp2 = Tp2 + geom_errorbar(aes(ymin = score-ci, ymax = score+ci), width = .2)
Tp2 = Tp2 + theme_bw()
Tp2 = Tp2 + scale_linetype_discrete(name = "Variablity Condtion", breaks = c("LV","HV", "HVB"), labels = c("LV", "HV", "HVB")) + scale_shape_discrete(name = "Variablity Condtion", breaks = c("LV","HV", "HVB"), labels = c("LV", "HV", "HVB"))+ scale_colour_grey(name = "Variablity Condtion", breaks = c("LV","HV", "HVB"), labels = c("LV", "HV", "HVB"))
Tp2 = Tp2 + coord_cartesian(ylim = c(0.5, 1.0))
Tp2 = Tp2 + ggtitle('Training')
Tp2 = Tp2 + theme(panel.background = element_rect(fill = 'white'), panel.grid.major = element_line(colour = "white"))
Tp2 = Tp2 + labs(x = "Training Session", y = "Mean Accuracy")
print(Tp2)
```

### CSTC graph (Not used)


```
Tp3 = ggplot(graphdata2, aes(x = session, y = score, group = condition, colour = condition)) + geom_line(aes(linetype = condition),size = 0.8) + geom_point(aes(shape = condition), size = 2.5)
Tp3 = Tp3 + facet_wrap(~ aptitude3, ncol = 2)
Tp3 = Tp3 + geom_errorbar(aes(ymin = score-ci, ymax = score+ci), width = .2)
Tp3 = Tp3 + theme_bw()
Tp3 = Tp3 + scale_linetype_discrete(name = "Variability Condition", breaks = c("LV","HV", "HVB"), labels = c("LV", "HV", "HVB")) + scale_shape_discrete(name = "Variability Condition", breaks = c("LV","HV", "HVB"), labels = c("LV", "HV", "HVB")) + scale_colour_grey(name = "Variability Condition", breaks = c("LV","HV", "HVB"), labels = c("LV", "HV", "HVB"))
Tp3 = Tp3 + coord_cartesian(ylim = c(0.5, 1.0))
Tp3 = Tp3 + ggtitle('Training')
Tp3 = Tp3 + theme(panel.background = element_rect(fill = 'white'), panel.grid.major = element_line(colour = "white"))
Tp3 = Tp3 + labs(x = "Training Session", y = "Mean Accuracy")
print(Tp3)
```

## Statistical Analysis

### Main Model (without aptitude predictors; Section 3.3)


```
## Note that a full model with all three conditions in didn't converge so model is split into two (converging) models
train2 = lizContrasts(train2, train2$condition, "LV")
train2$aptitude2 = relevel(train2$aptitude2, ref="LA")
train2 = lizCenter(train2, list("session","aptitude_pcpt", "aptitude2"))
train2.noHVB = lizCenter(subset(train2, condition != "HVB"), list("session", "condition", "aptitude_pcpt", "slope", "aptitude2"))
train2.noHV = lizCenter(subset(train2, condition != "HV"), list("session", "condition", "aptitude_pcpt", "slope","aptitude2"))
t_glmer_LVvHV = glmer(score ~ session.ct * condition.ct
              + (session||subject)
              , family="binomial", 
              control = glmerControl(optimizer = "bobyqa"), 
              data = train2.noHVB)
t_glmer_LVvHVB = glmer(score ~ session.ct * condition.ct
              + (session||subject)
              , family = "binomial", 
              control = glmerControl(optimizer = "bobyqa"), 
              data = train2.noHV)
```


#### Print key cofficients


```
kable(summary(t_glmer_LVvHV)$coefficients, digits = 6)
```

|  | Estimate | Std. Error | z value | Pr(>|z|) |
| --- | --- | --- | --- | --- |
| (Intercept) | 2.196171 | 0.158085 | 13.892378 | 0e+00 |
| session.ct | 0.492404 | 0.042730 | 11.523717 | 0e+00 |
| condition.ct | -0.784734 | 0.156087 | -5.027547 | 0e+00 |
| session.ct:condition.ct | -0.193688 | 0.042161 | -4.593945 | 4e-06 |

```
kable(summary(t_glmer_LVvHVB)$coefficients, digits = 3)
```


|  | Estimate | Std. Error | z value | Pr(>|z|) |
| --- | --- | --- | --- | --- |
| (Intercept) | 2.615 | 0.170 | 15.378 | 0.000 |
| session.ct | 0.526 | 0.043 | 12.172 | 0.000 |
| condition.ct | -0.830 | 0.318 | -2.610 | 0.009 |
| session.ct:condition.ct | -0.352 | 0.081 | -4.328 | 0.000 |

### Analysis with PCPT accuracy (Section 3.7)


```
# Note: models separate HV & HVB won't converge with both aptitude and session as predictors. The original model with all conditions but without session is used. 
t_glmer_pcpt = glmer(score ~ (LV_VERSUS_HV + LV_VERSUS_HVB) * aptitude_pcpt.ct 
                + (1|subject)
              , family = "binomial", 
              control = glmerControl(optimizer = "bobyqa"), 
              data = train2)
```


#### Print key cofficients


```
kable (round(summary(t_glmer_pcpt)$coefficients, 6), digits = 3)
```


|  | Estimate | Std. Error | z value | Pr(>|z|) |
| --- | --- | --- | --- | --- |
| (Intercept) | 1.823 | 0.087 | 21.034 | 0.000 |
| LV\_VERSUS\_HV | -1.033 | 0.167 | -6.197 | 0.000 |
| LV\_VERSUS\_HVB | -0.506 | 0.189 | -2.681 | 0.007 |
| aptitude\_pcpt.ct | 0.129 | 0.048 | 2.698 | 0.007 |
| LV\_VERSUS\_HV:aptitude\_pcpt.ct | -0.037 | 0.111 | -0.332 | 0.740 |
| LV\_VERSUS\_HVB:aptitude\_pcpt.ct | 0.026 | 0.100 | 0.260 | 0.795 |

### Extra analysis requested by reviewers, with categorical aptitude measure from PCPT accuracy


```
# Note: models separate HV & HVB won't converge with both aptitude and session as predictors. The original model with all conditions but without session is used. 
t_glmer_pcpt2 = glmer(score ~ (LV_VERSUS_HV + LV_VERSUS_HVB) * aptitude2.ct 
                + (1|subject)
              , family = "binomial", 
              control = glmerControl(optimizer = "bobyqa"), 
              data = train2)
```


#### Print key cofficients


```
kable (round(summary(t_glmer_pcpt2)$coefficients, 6), digits = 3)
```


|  | Estimate | Std. Error | z value | Pr(>|z|) |
| --- | --- | --- | --- | --- |
| (Intercept) | 1.829 | 0.089 | 20.467 | 0.000 |
| LV\_VERSUS\_HV | -1.099 | 0.187 | -5.871 | 0.000 |
| LV\_VERSUS\_HVB | -0.528 | 0.177 | -2.984 | 0.003 |
| aptitude2.ct | 0.269 | 0.162 | 1.664 | 0.096 |
| LV\_VERSUS\_HV:aptitude2.ct | -0.317 | 0.315 | -1.004 | 0.315 |
| LV\_VERSUS\_HVB:aptitude2.ct | 0.213 | 0.288 | 0.740 | 0.459 |

### Analysis with CSTC slope


```
# Note: full model didn't converge, so removed slope.ct:condition.ct:session.ct
t_glmer_LVvHV_cstc = glmer(score ~ session.ct * condition.ct + slope.ct + (slope.ct : condition.ct)
              + (session||subject)
              , family = "binomial" , 
              control = glmerControl(optimizer = "bobyqa"), 
              data = train2.noHVB)
kable (round(summary(t_glmer_LVvHV_cstc)$coefficients, 6), digits = 3)
```

|  | Estimate | Std. Error | z value | Pr(>|z|) |
| --- | --- | --- | --- | --- |
| (Intercept) | 2.145 | 0.153 | 14.053 | 0.000 |
| session.ct | 0.490 | 0.042 | 11.652 | 0.000 |
| condition.ct | -0.731 | 0.152 | -4.820 | 0.000 |
| slope.ct | 0.154 | 0.052 | 2.945 | 0.003 |
| session.ct:condition.ct | -0.190 | 0.042 | -4.560 | 0.000 |
| condition.ct:slope.ct | -0.109 | 0.052 | -2.085 | 0.037 |

```
t_glmer_LVvHVB_cstc = glmer(score ~ session.ct * condition.ct + slope.ct + (slope.ct : condition.ct)
              + (session||subject)
              , family = "binomial", 
              control = glmerControl(optimizer = "bobyqa"), 
              data = train2.noHV)
kable (round(summary(t_glmer_LVvHVB_cstc)$coefficients, 6), digits = 3)
```


|  | Estimate | Std. Error | z value | Pr(>|z|) |
| --- | --- | --- | --- | --- |
| (Intercept) | 2.554 | 0.170 | 15.030 | 0.000 |
| session.ct | 0.524 | 0.043 | 12.172 | 0.000 |
| condition.ct | -0.605 | 0.329 | -1.838 | 0.066 |
| slope.ct | 0.227 | 0.081 | 2.802 | 0.005 |
| session.ct:condition.ct | -0.347 | 0.083 | -4.164 | 0.000 |
| condition.ct:slope.ct | -0.093 | 0.170 | -0.548 | 0.584 |

# Picture Identification (Section 3.4.2)

## Label Parameters


```
PI$condition = factor(PI$condition, levels = c("0", "1", "2"), labels = c("LV", "HV", "HVB"))
PI$voicetype = factor(PI$voicetype, levels = c("nv1", "tv1"), labels = c("Untrained Voice", "Trained voice"))
PI2 = merge(ID_pre, PI)
```

## Figures


```
means = with(PI2, aggregate(score ~ subject + condition + voicetype, FUN = mean))
IDmeans = with (PI2, aggregate(aptitude_pcpt ~ subject + condition + voicetype, FUN = mean))
means = merge (means, IDmeans)
meansb = with(PI2, aggregate(score ~ subject + condition + voicetype + aptitude2, FUN = mean))
means2b = with(PI2, aggregate(score ~ subject + condition + voicetype + aptitude3, FUN = mean))
x <- summarySEwithin(means, measurevar = "score", betweenvars = c("condition"), withinvars = c("voicetype"), idvar = "subject", na.rm = FALSE, conf.interval = .95)
graphdata <- summarySEwithin(meansb, measurevar = "score", betweenvars = c("condition", "aptitude2"), withinvars = c("voicetype"), idvar = "subject", na.rm = FALSE, conf.interval = .95)
graphdata2 <- summarySEwithin(means2b, measurevar = "score", betweenvars = c("condition", "aptitude3"), withinvars = c("voicetype"), idvar = "subject", na.rm = FALSE, conf.interval = .95)
```


### Main Graph (Figure 6)


```
PIp1 = ggplot (means, aes(x = condition, y = score, fill = voicetype)) 
PIp1 = PIp1 + geom_violin(colour = "black", scale = "count")
PIp1 = PIp1 + scale_fill_manual(name = "Voice type", values = c("Untrained Voice" = "grey", "Trained voice" = "white"), labels = c("Untrained Voice", "Trained voice"))
PIp1 = PIp1 + scale_x_discrete(labels = c("LV", "HV", "HVB"))
PIp1 = PIp1 + geom_errorbar(aes(ymin = score-ci, ymax = score+ci), width = .4, position = position_dodge(.9), data = x)
PIp1 = PIp1 + stat_summary(fun.y = mean, geom = "point", shape = 23, size = 2,position = position_dodge(.9))
PIp1 = PIp1 + ggtitle('Picture Identification')
PIp1 = PIp1 + labs(x = "Variability Condition", y = "Mean Accuracy")
PIp1 = PIp1 + theme_bw()
print(PIp1)
```

### Main Graph 2 (Scatter Plot, Figure 11, Bottom panel, right)


```
PIp0.1m = ggplot(means, aes(aptitude_pcpt, score, shape = condition, colour = condition)) 
PIp0.1m = PIp0.1m + geom_point(aes (shape = condition), size = 2) + geom_smooth(se = FALSE, method = lm, aes(linetype = condition))
PIp0.1m = PIp0.1m + scale_linetype_discrete(name = " ", breaks = c("LV","HV", "HVB"), labels = c("LV", "HV", "HVB")) + scale_shape_discrete(name = " ", breaks = c("LV", "HV", "HVB"), labels = c("LV", "HV", "HVB")) + scale_colour_grey(name = " ", breaks = c("LV","HV", "HVB"), labels = c("LV", "HV", "HVB"))
PIp0.1m = PIp0.1m + labs(x = "PCPT Score", y = "Mean Accuracy") +  ggtitle(' ')
print(PIp0.1m)
```

### PCPT Graph (Figure 11 Bottom panel, left)


```
PIp2 = ggplot (meansb, aes(x = condition, y = score, fill = voicetype)) 
PIp2 = PIp2 + facet_wrap(~aptitude2, ncol = 2)
PIp2 = PIp2 + geom_violin(colour = "black", scale = "count")
PIp2 = PIp2 + scale_fill_manual(name = "Voice type", values = c("Untrained Voice" = "grey", "Trained voice" = "white"), labels = c("Untrained Voice", "Trained voice"))
PIp2 = PIp2 + scale_x_discrete(labels = c("LV", "HV", "HVB"))
PIp2 = PIp2 + geom_errorbar(aes(ymin = score-ci, ymax = score+ci), width = .4, position = position_dodge(.9), data = graphdata)
PIp2 = PIp2 + stat_summary(fun.y = mean, geom = "point", shape = 23, size = 2,position = position_dodge(.9))
PIp2 = PIp2 + ggtitle('Picture Identification')
PIp2 = PIp2 + labs(x = "Variability Condition", y = "Mean Accuracy")
PIp2 = PIp2 + theme_bw()
PIp2.m = PIp2 + theme(legend.position = c(0.8, 0.1), legend.key.size = unit(0.3,"cm"), legend.title = element_text(size=10), legend.text = element_text(size = 8))
print(PIp2)
```

### CSTC graph (Not used)


```
PIp3 = ggplot (means2b, aes(x = condition, y = score, fill = voicetype)) 
PIp3 = PIp3 + facet_wrap(~aptitude3, ncol = 2)
PIp3 = PIp3 + geom_violin(colour = "black", scale = "count")
PIp3 = PIp3 + scale_fill_manual(name = "Voice type", values = c("Untrained Voice" = "grey", "Trained voice" = "white"), labels = c("Untrained Voice", "Trained voice"))
PIp3 = PIp3 + scale_x_discrete(labels = c("Low Variability", "High Variability", "High Varaibility Blocking"))
PIp3 = PIp3 + geom_errorbar(aes(ymin = score-ci, ymax = score+ci), width = .4, position = position_dodge(.9), data = graphdata2)
PIp3 = PIp3 + stat_summary(fun.y = mean, geom = "point", shape = 23, size = 2,position = position_dodge(.9))
PIp3 = PIp3 + ggtitle('Picture Identification')
PIp3 = PIp3 + labs(x = "Variability Condition", y = "Mean Accuracy")
PIp3 = PIp3 + theme_bw()
print(PIp3)
```

## Statistical Analysis

### Main Model (without individual difference measures; Section 3.4.2)


```
PI2 = lizCenter(PI2, list("voicetype", "aptitude_pcpt", "slope"))
PI2 = lizContrasts(PI2, PI2$condition, "LV")
p_glmer = glmer(score ~ voicetype.ct * (LV_VERSUS_HV + LV_VERSUS_HVB)
              + (voicetype.ct|subject)
              , family = "binomial", 
              control = glmerControl(optimizer = "bobyqa"), 
              data = PI2)
```


#### Print out key effects


```
kable(summary(p_glmer)$coefficients, digits = 3)
```


|  | Estimate | Std. Error | z value | Pr(>|z|) |
| --- | --- | --- | --- | --- |
| (Intercept) | 2.259 | 0.135 | 16.704 | 0.000 |
| voicetype.ct | 1.067 | 0.163 | 6.532 | 0.000 |
| LV\_VERSUS\_HV | -0.711 | 0.318 | -2.233 | 0.026 |
| LV\_VERSUS\_HVB | -0.144 | 0.324 | -0.444 | 0.657 |
| voicetype.ct:LV\_VERSUS\_HV | -1.187 | 0.346 | -3.432 | 0.001 |
| voicetype.ct:LV\_VERSUS\_HVB | -1.105 | 0.359 | -3.079 | 0.002 |

#### Re-fit the model with a separate slope for voicetyp.ct for each condition"


```
PI2 = lizCenter(PI2, list("voicetype", "aptitude_pcpt", "slope"))
PI2 = lizContrasts(PI2, PI2$condition, "LV")
p_glmerb = glmer(score ~ 
                   voicetype.ct :  condition
                 +(LV_VERSUS_HV + LV_VERSUS_HVB)
                 + (voicetype.ct|subject)
                 , family = "binomial",
                 control = glmerControl(optimizer = "bobyqa"), 
                 data = PI2)
```

#### To check it is the same model


```
anova(p_glmer, p_glmerb)
```


```
Data: PI2
Models:
p_glmer: score ~ voicetype.ct * (LV_VERSUS_HV + LV_VERSUS_HVB) + (voicetype.ct | 
p_glmer:     subject)
p_glmerb: score ~ voicetype.ct:condition + (LV_VERSUS_HV + LV_VERSUS_HVB) + 
p_glmerb:     (voicetype.ct | subject)
         Df    AIC    BIC  logLik deviance Chisq Chi Df Pr(>Chisq)
p_glmer   9 3182.8 3240.1 -1582.4   3164.8                        
p_glmerb  9 3182.8 3240.1 -1582.4   3164.8     0      0          1
```

#### Print out effect of voice-type for each condition


```
round(get_coeffs(p_glmerb, c("voicetype.ct:conditionLV", "voicetype.ct:conditionHV", "voicetype.ct:conditionHVB")), 3)
```

#### Re-fit the model with a separate slope for the contrast between conditions for trained and untrained nouns


```
PI2 = lizCenter(PI2, list("voicetype", "aptitude_pcpt", "slope"))
PI2 = lizContrasts(PI2, PI2$condition, "LV")
p_glmerc = glmer(score ~ 
                voicetype : LV_VERSUS_HV 
               + voicetype : LV_VERSUS_HVB
               + voicetype.ct 
               + (voicetype.ct|subject)
              , family = "binomial", 
              control = glmerControl(optimizer = "bobyqa"), 
              data = PI2)
```

#### To check it is the same model


```
anova(p_glmer, p_glmerc)
```


```
Data: PI2
Models:
p_glmer: score ~ voicetype.ct * (LV_VERSUS_HV + LV_VERSUS_HVB) + (voicetype.ct | 
p_glmer:     subject)
p_glmerc: score ~ voicetype:LV_VERSUS_HV + voicetype:LV_VERSUS_HVB + voicetype.ct + 
p_glmerc:     (voicetype.ct | subject)
         Df    AIC    BIC  logLik deviance Chisq Chi Df Pr(>Chisq)
p_glmer   9 3182.8 3240.1 -1582.4   3164.8                        
p_glmerc  9 3182.8 3240.1 -1582.4   3164.8     0      0          1
```

#### Print out the contrast between conditions for trained and untrained nouns


```
kable(summary(p_glmerc)$coeff, digits = 3)
```


|  | Estimate | Std. Error | z value | Pr(>|z|) |
| --- | --- | --- | --- | --- |
| (Intercept) | 2.259 | 0.135 | 16.704 | 0.000 |
| voicetype.ct | 1.067 | 0.163 | 6.532 | 0.000 |
| voicetypeUntrained Voice:LV\_VERSUS\_HV | -0.118 | 0.263 | -0.448 | 0.654 |
| voicetypeTrained voice:LV\_VERSUS\_HV | -1.304 | 0.440 | -2.965 | 0.003 |
| voicetypeUntrained Voice:LV\_VERSUS\_HVB | 0.409 | 0.270 | 1.512 | 0.130 |
| voicetypeTrained voice:LV\_VERSUS\_HVB | -0.696 | 0.448 | -1.553 | 0.120 |

### Analysis with PCPT accuracy (Section 3.7)


```
p_glmer_pcpt = glmer(score ~ voicetype.ct * (LV_VERSUS_HV + LV_VERSUS_HVB) * aptitude_pcpt.ct
                + (voicetype.ct|subject)
                , family = "binomial", 
                control = glmerControl(optimizer = "bobyqa"), 
                data = PI2)
```


#### Check that we get the same pattern of results for the experimentally manipulated variables as in the previous model


```
kable(get_coeffs(p_glmer_pcpt, c("voicetype.ct", "LV_VERSUS_HV", "LV_VERSUS_HVB", "voicetype.ct:LV_VERSUS_HV", "voicetype.ct:LV_VERSUS_HVB")), digits = 3)
```


|  | Estimate | Std. Error | z value | Pr(>|z|) |
| --- | --- | --- | --- | --- |
| voicetype.ct | 1.144 | 0.166 | 6.892 | 0.000 |
| LV\_VERSUS\_HV | -0.604 | 0.326 | -1.854 | 0.064 |
| LV\_VERSUS\_HVB | -0.197 | 0.318 | -0.619 | 0.536 |
| voicetype.ct:LV\_VERSUS\_HV | -0.964 | 0.352 | -2.738 | 0.006 |
| voicetype.ct:LV\_VERSUS\_HVB | -1.013 | 0.346 | -2.928 | 0.003 |

#### Print out the effect of individual aptitude and key interactions with aptitude


```
kable(get_coeffs(p_glmer_pcpt, c("aptitude_pcpt.ct", "voicetype.ct:aptitude_pcpt.ct", "LV_VERSUS_HV:aptitude_pcpt.ct", "LV_VERSUS_HVB:aptitude_pcpt.ct", "voicetype.ct:LV_VERSUS_HV:aptitude_pcpt.ct", "voicetype.ct:LV_VERSUS_HVB:aptitude_pcpt.ct")), digits = 3)
```


|  | Estimate | Std. Error | z value | Pr(>|z|) |
| --- | --- | --- | --- | --- |
| aptitude\_pcpt.ct | 0.148 | 0.075 | 1.962 | 0.050 |
| voicetype.ct:aptitude\_pcpt.ct | -0.028 | 0.086 | -0.325 | 0.745 |
| LV\_VERSUS\_HV:aptitude\_pcpt.ct | -0.023 | 0.189 | -0.124 | 0.901 |
| LV\_VERSUS\_HVB:aptitude\_pcpt.ct | 0.014 | 0.165 | 0.085 | 0.932 |
| voicetype.ct:LV\_VERSUS\_HV:aptitude\_pcpt.ct | 0.347 | 0.212 | 1.632 | 0.103 |
| voicetype.ct:LV\_VERSUS\_HVB:aptitude\_pcpt.ct | -0.107 | 0.185 | -0.575 | 0.566 |

### Analysis with CSTC slope


```
p_glmer_cstc = glmer(score ~ voicetype.ct * (LV_VERSUS_HV + LV_VERSUS_HVB) * slope.ct
                + (voicetype.ct|subject)
                , family = "binomial", 
                control = glmerControl(optimizer = "bobyqa"), 
                data = PI2)
```


#### Check that we get the same pattern of results for the experimentally manipulated variables as in the previous model


```
kable(get_coeffs(p_glmer_cstc, c("voicetype.ct", "LV_VERSUS_HV",  "LV_VERSUS_HVB", "voicetype.ct:LV_VERSUS_HV", "voicetype.ct:LV_VERSUS_HVB")), digits = 3)
```


|  | Estimate | Std. Error | z value | Pr(>|z|) |
| --- | --- | --- | --- | --- |
| voicetype.ct | 1.035 | 0.165 | 6.280 | 0.000 |
| LV\_VERSUS\_HV | -0.583 | 0.317 | -1.836 | 0.066 |
| LV\_VERSUS\_HVB | -0.058 | 0.347 | -0.167 | 0.867 |
| voicetype.ct:LV\_VERSUS\_HV | -1.178 | 0.347 | -3.398 | 0.001 |
| voicetype.ct:LV\_VERSUS\_HVB | -1.220 | 0.384 | -3.178 | 0.001 |

#### Print out the effect of individual aptitude and key interactions with aptitude


```
kable(get_coeffs(p_glmer_cstc, c("slope.ct", "voicetype.ct:slope.ct", "LV_VERSUS_HV:slope.ct", "LV_VERSUS_HVB:slope.ct", "voicetype.ct:LV_VERSUS_HV:slope.ct", "voicetype.ct:LV_VERSUS_HVB:slope.ct")), digits = 3)
```


|  | Estimate | Std. Error | z value | Pr(>|z|) |
| --- | --- | --- | --- | --- |
| slope.ct | 0.087 | 0.131 | 0.663 | 0.507 |
| voicetype.ct:slope.ct | -0.115 | 0.142 | -0.807 | 0.420 |
| LV\_VERSUS\_HV:slope.ct | -0.282 | 0.293 | -0.965 | 0.335 |
| LV\_VERSUS\_HVB:slope.ct | -0.404 | 0.325 | -1.242 | 0.214 |
| voicetype.ct:LV\_VERSUS\_HV:slope.ct | 0.028 | 0.327 | 0.085 | 0.932 |
| voicetype.ct:LV\_VERSUS\_HVB:slope.ct | -0.238 | 0.364 | -0.653 | 0.514 |

# Picture Naming (Section 3.5.2)

## Picture Naming - Tone accuracy

### Label parameters and remove unidentifiable trials


```
NPN = CPN[CPN$tone != 0, ]
NPN$condition = factor(NPN$condition, levels = c("0", "1", "2"), labels = c("LV", "HV", "HVB"))
NPN2 = merge(ID_pre, NPN)
```

### Figures


```
means = with(NPN2, aggregate(tone_score ~ subject + condition, FUN = mean))
IDmeans = with (NPN2, aggregate(aptitude_pcpt ~ subject + condition, FUN = mean))
means = merge (means, IDmeans)
meansb = with(NPN2, aggregate(tone_score ~ subject + condition + aptitude2, FUN = mean))
means2b = with(NPN2, aggregate(tone_score ~ subject + condition + aptitude3, FUN = mean))
xb <- summarySE(meansb, measurevar = "tone_score", groupvars = c("condition"), na.rm = FALSE, conf.interval = .95)
graphdatab <- summarySE(meansb, measurevar = "tone_score", groupvars = c("condition", "aptitude2"), na.rm = FALSE, conf.interval = .95)
graphdata2b <- summarySE(means2b, measurevar = "tone_score", groupvars = c("condition", "aptitude3"), na.rm = FALSE, conf.interval = .95)
```


#### Main Figure (Figure 9, left)


```
PNp1 = ggplot (means, aes(x = condition, y = tone_score)) 
PNp1 = PNp1 + geom_violin(colour = "black", scale = "count")
PNp1 = PNp1 + scale_x_discrete(labels = c("LV", "HV", "HVB"))
PNp1 = PNp1 + geom_errorbar(aes(ymin = tone_score-ci, ymax = tone_score+ci), width = .4, position = position_dodge(.9), data = xb)
PNp1 = PNp1 + stat_summary(fun.y = mean, geom = "point", shape = 23, size = 2,position = position_dodge(.9))
PNp1 = PNp1 +theme_bw() + ggtitle('Picture Naming - Tone accuracy')
PNp1 = PNp1 + labs(x = "Variability Condition", y = "Mean Accuracy")
print(PNp1)
```

#### Main Figure (Scatter plot, Figure 11, Top panel, right)


```
PNp0.1 = ggplot(means, aes(aptitude_pcpt, tone_score,shape = condition, colour = condition)) 
PNp0.1 = PNp0.1 + geom_point(aes (shape = condition), size = 2) + geom_smooth(se = FALSE, method = lm, aes(linetype = condition))
PNp0.1 = PNp0.1 + scale_linetype_discrete(name = "Variability Condition", breaks = c("LV","HV", "HVB"), labels = c("LV", "HV", "HVB")) + scale_shape_discrete(name = "Variability Condition", breaks = c("LV","HV", "HVB"), labels = c("LV", "HV", "HVB")) + scale_colour_grey(name = "Variability Condition", breaks = c("LV","HV", "HVB"), labels = c("LV", "HV", "HVB"))
PNp0.1 = PNp0.1 + labs(x = "PCPT Score",y = NULL) + ggtitle(' ')
PNp0.1.m = PNp0.1 + scale_linetype_discrete(name = " ", breaks = c("LV","HV", "HVB"), labels = c("LV", "HV", "HVB")) + scale_shape_discrete(name = " ", breaks = c("LV","HV", "HVB"), labels = c("LV", "HV", "HVB")) + scale_colour_grey(name = " ", breaks = c("LV","HV", "HVB"), labels = c("LV", "HV", "HVB"))
```


```
Scale for 'linetype' is already present. Adding another scale for 'linetype', which will replace the existing scale.
Scale for 'shape' is already present. Adding another scale for 'shape', which will replace the existing scale.
Scale for 'colour' is already present. Adding another scale for 'colour', which will replace the existing scale.
```


```
PNp0.1.m = PNp0.1.m + labs(x = "PCPT Score", y = "Mean Accuracy") + ggtitle(' ')
print(PNp0.1)
```

#### PCPT Graph (Figure 11 Top panel,left)


```
PNp2 = ggplot (meansb, aes(x = condition, y = tone_score)) 
PNp2 = PNp2 + geom_violin(colour = "black", scale = "count")
PNp2 = PNp2 + facet_wrap(~aptitude2, ncol = 2)
PNp2 = PNp2 + scale_x_discrete(labels = c("LV", "HV", "HVB"))
PNp2 = PNp2 + geom_errorbar(aes(ymin = tone_score-ci, ymax = tone_score+ci), width = .4, position = position_dodge(.9), data = graphdatab)
PNp2 = PNp2 + stat_summary(fun.y = mean, geom = "point", shape = 23, size = 2, position = position_dodge(.9))
PNp2 = PNp2 + theme_bw()+ ggtitle('Picture Naming: Tone accuracy')
PNp2 = PNp2 + labs(x = "Variability Condition", y = "Mean Accuracy")
print(PNp2)
```

#### CSTC graph (Not used)


```
PNp3 = ggplot (means2b, aes(x = condition,y = tone_score, fill = aptitude3)) 
PNp3 = PNp3 + geom_violin(colour = "black", scale = "count")
PNp3 = PNp3 + scale_fill_manual(name = "Aptitude", values = c("LA" = "grey", "HA" = "white"), labels = c("LA", "HA"))
PNp3 = PNp3 + scale_x_discrete(labels = c("LV", "HV", "HVB"))
PNp3 = PNp3 + geom_errorbar(aes(ymin = tone_score-ci, ymax = tone_score+ci), width = .4, position = position_dodge(.9), data = graphdata2b)
PNp3 = PNp3 + stat_summary(fun.y = mean, geom = "point", shape = 23, size = 2,position = position_dodge(.9))
PNp3 = PNp3 + theme_bw()+ ggtitle('Picture Naming: Tone accuracy')
PNp3 = PNp3 + labs(x = "Variability Condition", y = "Mean Accuracy")
print(PNp3)
```

### Statistical Analysis

#### Main model (without aptitude predictors; Section 3.5.2)


```
NPN2 = lizCenter(NPN2, list("aptitude_pcpt", "slope"))
NPN2 = lizContrasts(NPN2, NPN2$condition, "LV")
NPN_glmer = glmer(tone_score ~ (LV_VERSUS_HV + LV_VERSUS_HVB) 
              + (1|subject)
              , family = "binomial", 
              control = glmerControl(optimizer = "bobyqa"), 
              data = NPN2)
```


##### Print out effects


```
kable(summary(NPN_glmer)$coefficients, digits = 3)
```


|  | Estimate | Std. Error | z value | Pr(>|z|) |
| --- | --- | --- | --- | --- |
| (Intercept) | -0.022 | 0.079 | -0.283 | 0.777 |
| LV\_VERSUS\_HV | -0.344 | 0.190 | -1.806 | 0.071 |
| LV\_VERSUS\_HVB | -0.100 | 0.193 | -0.516 | 0.606 |

#### Analysis with PCPT accuracy (Section 3.7)


```
NPN_glmer_pcpt = glmer(tone_score ~ (LV_VERSUS_HV + LV_VERSUS_HVB) * aptitude_pcpt.ct
              + (1|subject)
              , family = "binomial", 
              control = glmerControl(optimizer = "bobyqa"), 
              data = NPN2)
```


##### Check that we get the same pattern of results for the experimentally manipulated variables as in the previous model


```
kable(get_coeffs(NPN_glmer_pcpt, c("(Intercept)", "LV_VERSUS_HV", "LV_VERSUS_HVB")), digits = 3)
```


|  | Estimate | Std. Error | z value | Pr(>|z|) |
| --- | --- | --- | --- | --- |
| (Intercept) | -0.056 | 0.076 | -0.729 | 0.466 |
| LV\_VERSUS\_HV | -0.340 | 0.186 | -1.830 | 0.067 |
| LV\_VERSUS\_HVB | -0.165 | 0.184 | -0.893 | 0.372 |

##### Print out the effect of individual aptitude and key interactions with aptitude


```
summary(NPN_glmer_pcpt)$coeff
```


```
                                  Estimate Std. Error    z value   Pr(>|z|)
(Intercept)                    -0.05571730 0.07642671 -0.7290292 0.46598383
LV_VERSUS_HV                   -0.34027230 0.18594921 -1.8299206 0.06726181
LV_VERSUS_HVB                  -0.16467235 0.18441131 -0.8929623 0.37187735
aptitude_pcpt.ct                0.08108510 0.04294653  1.8880476 0.05901957
LV_VERSUS_HV:aptitude_pcpt.ct  -0.08804532 0.10505701 -0.8380719 0.40199034
LV_VERSUS_HVB:aptitude_pcpt.ct  0.11659040 0.09578213  1.2172459 0.22351066
```


```
kable(get_coeffs(NPN_glmer_pcpt, c("aptitude_pcpt.ct", "LV_VERSUS_HV:aptitude_pcpt.ct", "LV_VERSUS_HVB:aptitude_pcpt.ct")), digits = 3)
```


|  | Estimate | Std. Error | z value | Pr(>|z|) |
| --- | --- | --- | --- | --- |
| aptitude\_pcpt.ct | 0.081 | 0.043 | 1.888 | 0.059 |
| LV\_VERSUS\_HV:aptitude\_pcpt.ct | -0.088 | 0.105 | -0.838 | 0.402 |
| LV\_VERSUS\_HVB:aptitude\_pcpt.ct | 0.117 | 0.096 | 1.217 | 0.224 |

#### Analysis with CSTC slope


```
NPN_glmer_cstc = glmer(tone_score ~ (LV_VERSUS_HV + LV_VERSUS_HVB) * slope.ct
              + (1|subject)
              , family = "binomial", 
              control = glmerControl(optimizer = "bobyqa"), 
              data = NPN2)
```


##### Check that we get the same pattern of results for the experimentally manipulated variables as in the previous model


```
kable(get_coeffs(NPN_glmer_cstc, c("(Intercept)", "LV_VERSUS_HV", "LV_VERSUS_HVB")), digits = 3)
```


|  | Estimate | Std. Error | z value | Pr(>|z|) |
| --- | --- | --- | --- | --- |
| (Intercept) | -0.050 | 0.084 | -0.603 | 0.546 |
| LV\_VERSUS\_HV | -0.283 | 0.193 | -1.463 | 0.143 |
| LV\_VERSUS\_HVB | -0.074 | 0.213 | -0.346 | 0.729 |

##### Print out the effect of individual aptitude and key interactions with aptitude


```
kable(get_coeffs(NPN_glmer_cstc, c("slope.ct", "LV_VERSUS_HV:slope.ct", "LV_VERSUS_HVB:slope.ct")), digits = 3)
```


|  | Estimate | Std. Error | z value | Pr(>|z|) |
| --- | --- | --- | --- | --- |
| slope.ct | -0.011 | 0.081 | -0.133 | 0.894 |
| LV\_VERSUS\_HV:slope.ct | -0.227 | 0.176 | -1.290 | 0.197 |
| LV\_VERSUS\_HVB:slope.ct | -0.191 | 0.198 | -0.962 | 0.336 |

## Picture Naming - Pinyin Accuracy

### Figures


```
means = with(NPN2, aggregate(pinyin_score ~ subject + condition, FUN = mean))
IDmeans = with (NPN2, aggregate(aptitude_pcpt ~ subject + condition, FUN = mean))
means = merge (means, IDmeans)
meansb = with(NPN2, aggregate(pinyin_score ~ subject + condition + aptitude2, FUN = mean))
means2b = with(NPN2, aggregate(pinyin_score ~ subject + condition + aptitude3, FUN = mean))
xb<- summarySE(meansb, measurevar = "pinyin_score", groupvars = c("condition"), na.rm = FALSE, conf.interval = .95)
graphdatab <- summarySE(meansb, measurevar = "pinyin_score", groupvars = c("condition", "aptitude2"), na.rm = FALSE, conf.interval = .95)
graphdata2b <- summarySE(means2b, measurevar = "pinyin_score", groupvars = c("condition", "aptitude3"), na.rm = FALSE, conf.interval = .95)
```


#### Main Graph (Figure 9, right)


```
PNp7 = ggplot (means, aes(x = condition, y=pinyin_score)) 
PNp7 = PNp7 + geom_violin(colour = "black", scale = "count")
PNp7 = PNp7 + scale_x_discrete(labels = c("LV", "HV", "HVB"))
PNp7 = PNp7 + geom_errorbar(aes(ymin = pinyin_score-ci, ymax = pinyin_score+ci), width = .4, position = position_dodge(.9), data = xb)
PNp7 = PNp7 + stat_summary(fun.y = mean, geom = "point", shape = 23, size = 2,position = position_dodge(.9))
PNp7 = PNp7 + theme_bw() + ggtitle('Picture Naming: Pinyin Accuracy')
PNp7 = PNp7 + labs(x = "Variability Condition", y = "Mean Accuracy")
print(PNp7)
```

#### Main Graph (Scatter plot, Figure 11, Middle panel, right)


```
PNp0.2 = ggplot(means, aes(aptitude_pcpt, pinyin_score, colour = condition, shape = condition)) 
PNp0.2 = PNp0.2 + geom_point(aes (shape = condition), size = 2) + geom_smooth(se = FALSE, method = lm, aes(linetype = condition))
PNp0.2 = PNp0.2 + scale_linetype_discrete(name = "Variability Condition", breaks = c("LV","HV", "HVB"), labels = c("LV", "HV", "HVB"))+ scale_shape_discrete(name = "Variability Condition", breaks = c("LV","HV", "HVB"), labels = c("LV", "HV", "HVB")) + scale_colour_grey(name = "Variability Condition", breaks = c("LV","HV", "HVB"), labels = c("LV", "HV", "HVB"))
PNp0.2 = PNp0.2 + labs(x = "PCPT Score", y = NULL) +  ggtitle('Scatter Plot')
PNp0.2.m = PNp0.2 + scale_linetype_discrete(name = " ", breaks = c("LV", "HV", "HVB"), labels = c("LV", "HV", "HVB")) + scale_shape_discrete(name = " ", breaks = c("LV","HV", "HVB"), labels = c("LV", "HV", "HVB")) + scale_colour_grey(name = " ", breaks = c("LV","HV", "HVB"), labels = c("LV", "HV", "HVB"))
```


```
Scale for 'linetype' is already present. Adding another scale for 'linetype', which will replace the existing scale.
Scale for 'shape' is already present. Adding another scale for 'shape', which will replace the existing scale.
Scale for 'colour' is already present. Adding another scale for 'colour', which will replace the existing scale.
```


```
PNp0.2.m = PNp0.2.m + labs(x = "PCPT Score", y = "Mean Accuracy") + ggtitle(' ')
print(PNp0.2)
```

#### PCPT Graph (Figure 11, Middle panel,left)


```
PNp8 = ggplot (meansb, aes(x = condition,y = pinyin_score)) 
PNp8 = PNp8 + geom_violin(colour = "black", scale = "count")
PNp8 = PNp8 + facet_wrap(~aptitude2, ncol = 2)
PNp8 = PNp8 + scale_x_discrete(labels = c("LV", "HV", "HVB"))
PNp8 = PNp8 + geom_errorbar(aes(ymin = pinyin_score-ci, ymax = pinyin_score+ci), width = .4, position = position_dodge(.9), data = graphdatab)
PNp8 = PNp8 + stat_summary(fun.y = mean, geom = "point", shape = 23, size = 2,position = position_dodge(.9))
PNp8 = PNp8 +theme_bw()+ ggtitle('Picture Naming: Pinyin Accuracy')
PNp8 = PNp8 + labs(x = "Variability Condition", y = "Mean Accuracy")
print(PNp8)
```

#### CSTC Graph (Not used)


```
PNp9 = ggplot (means2b, aes(x = condition, y = pinyin_score, fill = aptitude3)) 
PNp9 = PNp9 + geom_violin(colour = "black", scale = "count")
PNp9 = PNp9 + scale_fill_manual(name = "aptitude3", values = c("LA" = "grey", "HA" = "white"), labels = c("LA", "HA"))
PNp9 = PNp9 + scale_x_discrete(labels = c("LV", "HV", "HVB"))
PNp9 = PNp9 + geom_errorbar(aes(ymin = pinyin_score-ci, ymax = pinyin_score+ci), width = .4, position = position_dodge(.9), data = graphdata2b)
PNp9 = PNp9 + stat_summary(fun.y = mean, geom = "point", shape = 23, size = 2, position = position_dodge(.9))
PNp9 = PNp9 + theme_bw()+ ggtitle('Picture Naming: Pinyin Accuracy')
PNp9 = PNp9 + labs(x = "Variability Condition", y = "Mean Accuracy")
print(PNp9)
```

### Statistical Analysis

#### Main Model (without individual aptitude measures; section 3.5.2)


```
NPN2 = lizCenter(NPN2, list("aptitude_pcpt", "slope"))
NPN2 = lizContrasts(NPN2, NPN2$condition, "LV")
NPN_glmer = glmer(pinyin_score ~ (LV_VERSUS_HV + LV_VERSUS_HVB) 
              + (1|subject)
              , family = "binomial", 
              control = glmerControl(optimizer = "bobyqa"), 
              data = NPN2)
```

#### Print out key effects


```
kable(summary(NPN_glmer)$coefficients, digits = 3)
```


|  | Estimate | Std. Error | z value | Pr(>|z|) |
| --- | --- | --- | --- | --- |
| (Intercept) | -0.211 | 0.093 | -2.277 | 0.023 |
| LV\_VERSUS\_HV | 0.092 | 0.225 | 0.407 | 0.684 |
| LV\_VERSUS\_HVB | 0.117 | 0.228 | 0.513 | 0.608 |

#### Analysis with PCPT accuracy (Section 3.7)


```
NPN_glmer_pcpt = glmer(pinyin_score ~ (LV_VERSUS_HV + LV_VERSUS_HVB) * aptitude_pcpt.ct
              + (1|subject)
              , family = "binomial", 
              control = glmerControl(optimizer = "bobyqa"), 
              data = NPN2)
```


##### Check that we get the same pattern of results for the experimentally manipulated variables as in the previous model


```
kable(get_coeffs(NPN_glmer_pcpt, c("(Intercept)","LV_VERSUS_HV", "LV_VERSUS_HVB")), digits = 3)
```


|  | Estimate | Std. Error | z value | Pr(>|z|) |
| --- | --- | --- | --- | --- |
| (Intercept) | -0.220 | 0.094 | -2.351 | 0.019 |
| LV\_VERSUS\_HV | 0.016 | 0.228 | 0.070 | 0.944 |
| LV\_VERSUS\_HVB | 0.107 | 0.226 | 0.472 | 0.637 |

##### Print out the effect of individual aptitude and key interactions with aptitude


```
summary(NPN_glmer_pcpt)$coeff
```


```
                                   Estimate Std. Error     z value   Pr(>|z|)
(Intercept)                    -0.220256431 0.09367147 -2.35137167 0.01870434
LV_VERSUS_HV                    0.015938109 0.22817660  0.06984989 0.94431314
LV_VERSUS_HVB                   0.106559346 0.22579689  0.47192565 0.63697986
aptitude_pcpt.ct               -0.073188698 0.05257851 -1.39198894 0.16392575
LV_VERSUS_HV:aptitude_pcpt.ct   0.007484762 0.12884024  0.05809336 0.95367427
LV_VERSUS_HVB:aptitude_pcpt.ct  0.083974132 0.11694469  0.71806707 0.47271593
```


```
kable(get_coeffs(NPN_glmer_pcpt, c("aptitude_pcpt.ct", "LV_VERSUS_HV:aptitude_pcpt.ct", "LV_VERSUS_HVB:aptitude_pcpt.ct")), digits = 3)
```


|  | Estimate | Std. Error | z value | Pr(>|z|) |
| --- | --- | --- | --- | --- |
| aptitude\_pcpt.ct | -0.073 | 0.053 | -1.392 | 0.164 |
| LV\_VERSUS\_HV:aptitude\_pcpt.ct | 0.007 | 0.129 | 0.058 | 0.954 |
| LV\_VERSUS\_HVB:aptitude\_pcpt.ct | 0.084 | 0.117 | 0.718 | 0.473 |

#### Analysis with CSTC slope


```
NPN_glmer_cstc = glmer(pinyin_score ~ (LV_VERSUS_HV + LV_VERSUS_HVB) * slope.ct
              + (1|subject)
              , family = "binomial", 
              control = glmerControl(optimizer = "bobyqa"),  
              data = NPN2)
```


##### Check that we get the same pattern of results for the experimentally manipulated variables as in the previous model


```
kable(get_coeffs(NPN_glmer_cstc, c("(Intercept)","LV_VERSUS_HV", "LV_VERSUS_HVB")), digits = 3)
```


|  | Estimate | Std. Error | z value | Pr(>|z|) |
| --- | --- | --- | --- | --- |
| (Intercept) | -0.269 | 0.099 | -2.719 | 0.007 |
| LV\_VERSUS\_HV | 0.094 | 0.226 | 0.415 | 0.678 |
| LV\_VERSUS\_HVB | -0.068 | 0.253 | -0.267 | 0.790 |

##### Print out the effect of individual aptitude and key interactions with aptitude


```
kable(get_coeffs(NPN_glmer_cstc, c("slope.ct", "LV_VERSUS_HV:slope.ct", "LV_VERSUS_HVB:slope.ct")), digits = 3)
```


|  | Estimate | Std. Error | z value | Pr(>|z|) |
| --- | --- | --- | --- | --- |
| slope.ct | -0.157 | 0.097 | -1.622 | 0.105 |
| LV\_VERSUS\_HV:slope.ct | -0.111 | 0.205 | -0.542 | 0.588 |
| LV\_VERSUS\_HVB:slope.ct | -0.347 | 0.241 | -1.442 | 0.149 |

LS0tDQp0aXRsZTogJ01hbmRhcmluIFRyYWluaW5nIFN0dWR5OiBIaWdoIHZlcnN1cyBMb3cgU3BlYWtlciBWYXJpYWJpbGl0eScNCm91dHB1dDoNCiAgaHRtbF9ub3RlYm9vazoNCiAgICB0b2M6IHllcw0KICAgIHRvY19kZXB0aDogNA0KICB3b3JkX2RvY3VtZW50Og0KICAgIHRvYzogeWVzDQogICAgdG9jX2RlcHRoOiAnNCcNCiAgaHRtbF9kb2N1bWVudDoNCiAgICBkZl9wcmludDogcGFnZWQNCiAgICB0b2M6IHllcw0KICAgIHRvY19kZXB0aDogJzQnDQpkYXRlOiAiSnVseSwgMjAxOCINCi0tLQ0KIyBMb2FkIFBhY2thZ2VzIGFuZCBIZWxwZXIgRnVuY3Rpb25zDQoNCiMjIFBhY2thZ2VzIA0KDQpgYGB7cn0NCnJtKGxpc3Q9bHMoKSkNCg0Kc3VwcHJlc3NQYWNrYWdlU3RhcnR1cE1lc3NhZ2VzKGxpYnJhcnkoZ2dwbG90MikpDQpzdXBwcmVzc1BhY2thZ2VTdGFydHVwTWVzc2FnZXMobGlicmFyeShsbWVyVGVzdCkpDQpzdXBwcmVzc1BhY2thZ2VTdGFydHVwTWVzc2FnZXMobGlicmFyeShsbWU0KSkNCnN1cHByZXNzUGFja2FnZVN0YXJ0dXBNZXNzYWdlcyhsaWJyYXJ5KE1hdHJpeCkpDQpzdXBwcmVzc1BhY2thZ2VTdGFydHVwTWVzc2FnZXMobGlicmFyeShncmlkRXh0cmEpKQ0Kc3VwcHJlc3NQYWNrYWdlU3RhcnR1cE1lc3NhZ2VzKGxpYnJhcnkocGx5cikpDQpzdXBwcmVzc1BhY2thZ2VTdGFydHVwTWVzc2FnZXMobGlicmFyeShrbml0cikpDQpzdXBwcmVzc1BhY2thZ2VTdGFydHVwTWVzc2FnZXMobGlicmFyeShmbXNiKSkNCnN1cHByZXNzUGFja2FnZVN0YXJ0dXBNZXNzYWdlcyhsaWJyYXJ5KGlycikpDQpzdXBwcmVzc1BhY2thZ2VTdGFydHVwTWVzc2FnZXMobGlicmFyeShjb3dwbG90KSkNCnN1cHByZXNzUGFja2FnZVN0YXJ0dXBNZXNzYWdlcyhsaWJyYXJ5KGthYmxlRXh0cmEpKQ0Kc3VwcHJlc3NQYWNrYWdlU3RhcnR1cE1lc3NhZ2VzKHRoZW1lX3NldCh0aGVtZV9idygpKSkNCm9wdHNfY2h1bmskc2V0KGZpZy53aWR0aD04LCBmaWcuaGVpZ2h0PTUsIGVjaG89VFJVRSwgd2FybmluZz1GQUxTRSwgbWVzc2FnZT1GQUxTRSwgY2FjaGU9VFJVRSkNCmBgYA0KDQojIyBIZWxwZXIgRnVuY3Rpb25zDQoNCiMjIyBsb2dvZGRzDQoNClRoaXMgZnVuY3Rpb24gdGFrZXMgYSBwZXJjZW50YWdlIGFuZCByZXR1cm5zIHRoZSBsb2cgb2Rkcy4gDQoNCmBgYHtyfQ0KbG9nb2RkcyA8LSBmdW5jdGlvbihwKXtsb2cocC8oMTAwLXApKX0gIA0KYGBgDQoNCiMjIyBTdW1tYXJ5U0UNCg0KVGhpcyBmdW5jdGlvbiBjYW4gYmUgZm91bmQgb24gdGhlIHdlYnNpdGUgIkNvb2tib29rIGZvciBSIi4NCg0KaHR0cDovL3d3dy5jb29rYm9vay1yLmNvbS9HcmFwaHMvUGxvdHRpbmdfbWVhbnNfYW5kX2Vycm9yX2JhcnNfKGdncGxvdDIpLyNIZWxwZXINCg0KSXQgc3VtbWFyaXplcyBkYXRhLCBnaXZpbmcgY291bnQsIG1lYW4sIHN0YW5kYXJkIGRldmlhdGlvbiwgc3RhbmRhcmQgZXJyb3Igb2YgdGhlIG1lYW4sIGFuZCBjb25maWRlbmNlIGludGVydmFsIChkZWZhdWx0IDk1JSkuDQoNCi0gZGF0YTogYSBkYXRhIGZyYW1lDQotIG1lYXN1cmV2YXI6IHRoZSBuYW1lIG9mIGEgY29sdW1uIHRoYXQgY29udGFpbnMgdGhlIHZhcmlhYmxlIHRvIGJlIHN1bW1hcml6ZWQNCi0gZ3JvdXB2YXJzOiBhIHZlY3RvciBjb250YWluaW5nIG5hbWVzIG9mIGNvbHVtbnMgdGhhdCBjb250YWluIGdyb3VwaW5nIHZhcmlhYmxlcw0KLSBuYS5ybTogYSBib29sZWFuIHRoYXQgaW5kaWNhdGVzIHdoZXRoZXIgdG8gaWdub3JlIE5BJ3MNCi0gY29uZi5pbnRlcnZhbDogdGhlIHBlcmNlbnQgcmFuZ2Ugb2YgdGhlIGNvbmZpZGVuY2UgaW50ZXJ2YWwgKGRlZmF1bHQgaXMgOTUlKQ0KDQpgYGB7cn0NCnN1bW1hcnlTRSA8LSBmdW5jdGlvbihkYXRhID0gTlVMTCwgbWVhc3VyZXZhciwgZ3JvdXB2YXJzID0gTlVMTCwgbmEucm0gPSBGQUxTRSwgY29uZi5pbnRlcnZhbCA9IC45NSwgLmRyb3AgPSBUUlVFKSB7cmVxdWlyZShwbHlyKQ0KDQogICAgIyBOZXcgdmVyc2lvbiBvZiBsZW5ndGggd2hpY2ggY2FuIGhhbmRsZSBOQSdzOiBpZiBuYS5ybT09VCwgZG9uJ3QgY291bnQgdGhlbQ0KICAgIGxlbmd0aDIgPC0gZnVuY3Rpb24gKHgsIG5hLnJtID0gRkFMU0UpIHsNCiAgICAgICAgaWYgKG5hLnJtKSBzdW0oIWlzLm5hKHgpKQ0KICAgICAgICBlbHNlICAgICAgIGxlbmd0aCh4KQ0KICAgIH0NCg0KICAgICMgVGhpcyBkb2VzIHRoZSBzdW1tYXJ5LiBGb3IgZWFjaCBncm91cCdzIGRhdGEgZnJhbWUsIHJldHVybiBhIHZlY3RvciB3aXRoDQogICAgIyBOLCBtZWFuLCBhbmQgc2QNCiAgICBkYXRhYyA8LSBkZHBseShkYXRhLCBncm91cHZhcnMsIC5kcm9wID0gLmRyb3AsDQogICAgICAuZnVuID0gZnVuY3Rpb24oeHgsIGNvbCkgew0KICAgICAgICBjKE4gICAgPSBsZW5ndGgyKHh4W1tjb2xdXSwgbmEucm0gPSBuYS5ybSksDQogICAgICAgICAgbWVhbiA9IG1lYW4gICAoeHhbW2NvbF1dLCBuYS5ybSA9IG5hLnJtKSwNCiAgICAgICAgICBzZCAgID0gc2QgICAgICh4eFtbY29sXV0sIG5hLnJtID0gbmEucm0pDQogICAgICAgICkNCiAgICAgIH0sDQogICAgICBtZWFzdXJldmFyDQogICAgKQ0KDQogICAgIyBSZW5hbWUgdGhlICJtZWFuIiBjb2x1bW4gICAgDQogICAgZGF0YWMgPC0gcmVuYW1lKGRhdGFjLCBjKCJtZWFuIiA9IG1lYXN1cmV2YXIpKQ0KDQogICAgZGF0YWMkc2UgPC0gZGF0YWMkc2QgLyBzcXJ0KGRhdGFjJE4pICAjIENhbGN1bGF0ZSBzdGFuZGFyZCBlcnJvciBvZiB0aGUgbWVhbg0KDQogICAgIyBDb25maWRlbmNlIGludGVydmFsIG11bHRpcGxpZXIgZm9yIHN0YW5kYXJkIGVycm9yDQogICAgIyBDYWxjdWxhdGUgdC1zdGF0aXN0aWMgZm9yIGNvbmZpZGVuY2UgaW50ZXJ2YWw6IA0KICAgICMgZS5nLiwgaWYgY29uZi5pbnRlcnZhbCBpcyAuOTUsIHVzZSAuOTc1IChhYm92ZS9iZWxvdyksIGFuZCB1c2UgZGY9Ti0xDQogICAgY2lNdWx0IDwtIHF0KGNvbmYuaW50ZXJ2YWwvMiArIC41LCBkYXRhYyROLTEpDQogICAgZGF0YWMkY2kgPC0gZGF0YWMkc2UgKiBjaU11bHQNCg0KICAgIHJldHVybihkYXRhYykNCn0NCmBgYA0KDQojIyMgU3VtbWFyeVNFd2l0aGluDQoNClRoaXMgZnVuY3Rpb24gY2FuIGJlIGZvdW5kIG9uIHRoZSAgd2Vic2l0ZSAiQ29va2Jvb2sgZm9yIFIiLg0KDQpodHRwOi8vd3d3LmNvb2tib29rLXIuY29tL0dyYXBocy9QbG90dGluZ19tZWFuc19hbmRfZXJyb3JfYmFyc18oZ2dwbG90MikvI0hlbHBlciANCg0KRnJvbSB0aGF0IHdlYnNpdGU6DQoNClN1bW1hcml6ZXMgZGF0YSwgaGFuZGxpbmcgd2l0aGluLXN1YmplY3QgdmFyaWFibGVzIGJ5IHJlbW92aW5nIGludGVyLXN1YmplY3QgdmFyaWFiaWxpdHkuIEl0IHdpbGwgc3RpbGwgd29yayBpZiB0aGVyZSBhcmUgbm8gd2l0aGluLXN1YmplY3QgdmFyaWFibGVzLiBHaXZlcyBjb3VudCwgdW4tbm9ybWVkIG1lYW4sIG5vcm1lZCBtZWFuICh3aXRoIHNhbWUgYmV0d2Vlbi1ncm91cCBtZWFuKSwgc3RhbmRhcmQgZGV2aWF0aW9uLCBzdGFuZGFyZCBlcnJvciBvZiB0aGUgbWVhbiwgYW5kIGNvbmZpZGVuY2UgaW50ZXJ2YWwuIElmIHRoZXJlIGFyZSB3aXRoaW4tc3ViamVjdCB2YXJpYWJsZXMsIGl0IGNhbGN1bGF0ZXMgYWRqdXN0ZWQgdmFsdWVzIHVzaW5nIHRoZSBtZXRob2QgZnJvbSBNb3JleSAoMjAwOCkuDQoNCi0gZGF0YTogYSBkYXRhIGZyYW1lDQotIG1lYXN1cmV2YXI6IHRoZSBuYW1lIG9mIGEgY29sdW1uIHRoYXQgY29udGFpbnMgdGhlIHZhcmlhYmxlIHRvIGJlIHN1bW1hcml6ZWQNCi0gYmV0d2VlbnZhcnM6IGEgdmVjdG9yIGNvbnRhaW5pbmcgbmFtZXMgb2YgY29sdW1ucyB0aGF0IGFyZSBiZXR3ZWVuLXN1YmplY3RzIHZhcmlhYmxlcw0KLSB3aXRoaW52YXJzOiBhIHZlY3RvciBjb250YWluaW5nIG5hbWVzIG9mIGNvbHVtbnMgdGhhdCBhcmUgd2l0aGluLXN1YmplY3RzIHZhcmlhYmxlcw0KLSBpZHZhcjogdGhlIG5hbWUgb2YgYSBjb2x1bW4gdGhhdCBpZGVudGlmaWVzIGVhY2ggc3ViamVjdCAob3IgbWF0Y2hlZCBzdWJqZWN0cykNCi0gbmEucm06IGEgYm9vbGVhbiB0aGF0IGluZGljYXRlcyB3aGV0aGVyIHRvIGlnbm9yZSBOQSdzDQotIGNvbmYuaW50ZXJ2YWw6IHRoZSBwZXJjZW50IHJhbmdlIG9mIHRoZSBjb25maWRlbmNlIGludGVydmFsIChkZWZhdWx0IGlzIDk1JSkNCg0KYGBge3J9DQpzdW1tYXJ5U0V3aXRoaW4gPC0gZnVuY3Rpb24oZGF0YSA9IE5VTEwsIG1lYXN1cmV2YXIsIGJldHdlZW52YXJzID0gTlVMTCwgd2l0aGludmFycyA9IE5VTEwsDQogICAgICAgICAgICAgICAgICAgICAgICAgICAgaWR2YXIgPSBOVUxMLCBuYS5ybSA9IEZBTFNFLCBjb25mLmludGVydmFsID0gLjk1LCAuZHJvcCA9IFRSVUUpIHsNCg0KICAjIEVuc3VyZSB0aGF0IHRoZSBiZXR3ZWVudmFycyBhbmQgd2l0aGludmFycyBhcmUgZmFjdG9ycw0KICBmYWN0b3J2YXJzIDwtIHZhcHBseShkYXRhWywgYyhiZXR3ZWVudmFycywgd2l0aGludmFycyksIGRyb3AgPSBGQUxTRV0sDQogICAgRlVOID0gaXMuZmFjdG9yLCBGVU4uVkFMVUUgPSBsb2dpY2FsKDEpKQ0KDQogIGlmICghYWxsKGZhY3RvcnZhcnMpKSB7DQogICAgbm9uZmFjdG9ydmFycyA8LSBuYW1lcyhmYWN0b3J2YXJzKVshZmFjdG9ydmFyc10NCiAgICBtZXNzYWdlKCJBdXRvbWF0aWNhbGx5IGNvbnZlcnRpbmcgdGhlIGZvbGxvd2luZyBub24tZmFjdG9ycyB0byBmYWN0b3JzOiAiLA0KICAgICAgICAgICAgcGFzdGUobm9uZmFjdG9ydmFycywgY29sbGFwc2UgPSAiLCAiKSkNCiAgICBkYXRhW25vbmZhY3RvcnZhcnNdIDwtIGxhcHBseShkYXRhW25vbmZhY3RvcnZhcnNdLCBmYWN0b3IpDQogIH0NCg0KICAjIEdldCB0aGUgbWVhbnMgZnJvbSB0aGUgdW4tbm9ybWVkIGRhdGENCiAgZGF0YWMgPC0gc3VtbWFyeVNFKGRhdGEsIG1lYXN1cmV2YXIsIGdyb3VwdmFycyA9IGMoYmV0d2VlbnZhcnMsIHdpdGhpbnZhcnMpLA0KICAgICAgICAgICAgICAgICAgICAgbmEucm0gPSBuYS5ybSwgY29uZi5pbnRlcnZhbCA9IGNvbmYuaW50ZXJ2YWwsIC5kcm9wID0gLmRyb3ApDQoNCiAgIyBEcm9wIGFsbCB0aGUgdW51c2VkIGNvbHVtbnMgKHRoZXNlIHdpbGwgYmUgY2FsY3VsYXRlZCB3aXRoIG5vcm1lZCBkYXRhKQ0KICBkYXRhYyRzZCA8LSBOVUxMDQogIGRhdGFjJHNlIDwtIE5VTEwNCiAgZGF0YWMkY2kgPC0gTlVMTA0KDQogICMgTm9ybSBlYWNoIHN1YmplY3QncyBkYXRhDQogIG5kYXRhIDwtIG5vcm1EYXRhV2l0aGluKGRhdGEsIGlkdmFyLCBtZWFzdXJldmFyLCBiZXR3ZWVudmFycywgbmEucm0sIC5kcm9wID0gLmRyb3ApDQoNCiAgIyBUaGlzIGlzIHRoZSBuYW1lIG9mIHRoZSBuZXcgY29sdW1uDQogIG1lYXN1cmV2YXJfbiA8LSBwYXN0ZShtZWFzdXJldmFyLCAiX25vcm0iLCBzZXAgPSIiKQ0KDQogICMgQ29sbGFwc2UgdGhlIG5vcm1lZCBkYXRhIC0gbm93IHdlIGNhbiB0cmVhdCBiZXR3ZWVuIGFuZCB3aXRoaW4gdmFycyB0aGUgc2FtZQ0KICBuZGF0YWMgPC0gc3VtbWFyeVNFKG5kYXRhLCBtZWFzdXJldmFyX24sIGdyb3VwdmFycyA9IGMoYmV0d2VlbnZhcnMsIHdpdGhpbnZhcnMpLA0KICAgICAgICAgICAgICAgICAgICAgIG5hLnJtID0gbmEucm0sIGNvbmYuaW50ZXJ2YWwgPSBjb25mLmludGVydmFsLCAuZHJvcCA9IC5kcm9wKQ0KDQogICMgQXBwbHkgY29ycmVjdGlvbiBmcm9tIE1vcmV5ICgyMDA4KSB0byB0aGUgc3RhbmRhcmQgZXJyb3IgYW5kIGNvbmZpZGVuY2UgaW50ZXJ2YWwNCiAgIyBHZXQgdGhlIHByb2R1Y3Qgb2YgdGhlIG51bWJlciBvZiBjb25kaXRpb25zIG9mIHdpdGhpbi1TIHZhcmlhYmxlcw0KICBuV2l0aGluR3JvdXBzICAgIDwtIHByb2QodmFwcGx5KG5kYXRhY1ssd2l0aGludmFycywgZHJvcCA9IEZBTFNFXSwgRlVOID0gbmxldmVscywNCiAgICAgICAgICAgICAgICAgICAgICAgICAgIEZVTi5WQUxVRSA9IG51bWVyaWMoMSkpKQ0KICBjb3JyZWN0aW9uRmFjdG9yIDwtIHNxcnQoIG5XaXRoaW5Hcm91cHMgLyAobldpdGhpbkdyb3Vwcy0xKSApDQoNCiAgIyBBcHBseSB0aGUgY29ycmVjdGlvbiBmYWN0b3INCiAgbmRhdGFjJHNkIDwtIG5kYXRhYyRzZCAqIGNvcnJlY3Rpb25GYWN0b3INCiAgbmRhdGFjJHNlIDwtIG5kYXRhYyRzZSAqIGNvcnJlY3Rpb25GYWN0b3INCiAgbmRhdGFjJGNpIDwtIG5kYXRhYyRjaSAqIGNvcnJlY3Rpb25GYWN0b3INCg0KICAjIENvbWJpbmUgdGhlIHVuLW5vcm1lZCBtZWFucyB3aXRoIHRoZSBub3JtZWQgcmVzdWx0cw0KICBtZXJnZShkYXRhYywgbmRhdGFjKQ0KfQ0KYGBgDQoNCiMjIyBub3JtRGF0YVdpdGhpbg0KDQpUaGlzIGZ1bmN0aW9uIGlzIHVzZWQgYnkgdGhlIFN1bW1hcnlTRVdpdGhpbiBmdW5jdGlvbiBhYm92ZS4gSXQgY2FuIGJlIGZvdW5kIG9uIHRoZSB3ZWJzaXRlICJDb29rYm9vayBmb3IgUiIuDQoNCmh0dHA6Ly93d3cuY29va2Jvb2stci5jb20vR3JhcGhzL1Bsb3R0aW5nX21lYW5zX2FuZF9lcnJvcl9iYXJzXyhnZ3Bsb3QyKS8jSGVscGVyDQoNCkZyb20gdGhhdCB3ZWJzaXRlOg0KDQpOb3JtcyB0aGUgZGF0YSB3aXRoaW4gc3BlY2lmaWVkIGdyb3VwcyBpbiBhIGRhdGEgZnJhbWU7IGl0IG5vcm1hbGl6ZXMgZWFjaCBzdWJqZWN0IChpZGVudGlmaWVkIGJ5IGlkdmFyKSBzbyB0aGF0IHRoZXkgaGF2ZSB0aGUgc2FtZSBtZWFuLCB3aXRoaW4gZWFjaCBncm91cCBzcGVjaWZpZWQgYnkgYmV0d2VlbnZhcnMuDQoNCi0gZGF0YTogYSBkYXRhIGZyYW1lDQotIGlkdmFyOiB0aGUgbmFtZSBvZiBhIGNvbHVtbiB0aGF0IGlkZW50aWZpZXMgZWFjaCBzdWJqZWN0IChvciBtYXRjaGVkIHN1YmplY3RzKQ0KLSBtZWFzdXJldmFyOiB0aGUgbmFtZSBvZiBhIGNvbHVtbiB0aGF0IGNvbnRhaW5zIHRoZSB2YXJpYWJsZSB0byBiZSBzdW1tYXJpemVkDQotIGJldHdlZW52YXJzOiBhIHZlY3RvciBjb250YWluaW5nIG5hbWVzIG9mIGNvbHVtbnMgdGhhdCBhcmUgYmV0d2Vlbi1zdWJqZWN0cyB2YXJpYWJsZXMNCi0gbmEucm06IGEgYm9vbGVhbiB0aGF0IGluZGljYXRlcyB3aGV0aGVyIHRvIGlnbm9yZSBOQXMNCg0KYGBge3J9DQpub3JtRGF0YVdpdGhpbiA8LSBmdW5jdGlvbihkYXRhID0gTlVMTCwgaWR2YXIsIG1lYXN1cmV2YXIsIGJldHdlZW52YXJzID0gTlVMTCwNCiAgICAgICAgICAgICAgICAgICAgICAgICAgIG5hLnJtID0gRkFMU0UsIC5kcm9wID0gVFJVRSkgew0KICAgICNsaWJyYXJ5KHBseXIpDQoNCiAgICAjIE1lYXN1cmUgdmFyIG9uIGxlZnQsIGlkdmFyICsgYmV0d2VlbiB2YXJzIG9uIHJpZ2h0IG9mIGZvcm11bGEuDQogICAgZGF0YS5zdWJqTWVhbiA8LSBkZHBseShkYXRhLCBjKGlkdmFyLCBiZXR3ZWVudmFycyksIC5kcm9wID0gLmRyb3AsDQogICAgIC5mdW4gPSBmdW5jdGlvbih4eCwgY29sLCBuYS5ybSkgew0KICAgICAgICBjKHN1YmpNZWFuID0gbWVhbih4eFssY29sXSwgbmEucm0gPSBuYS5ybSkpDQogICAgICB9LA0KICAgICAgbWVhc3VyZXZhciwNCiAgICAgIG5hLnJtDQogICAgKQ0KDQogICAgIyBQdXQgdGhlIHN1YmplY3QgbWVhbnMgd2l0aCBvcmlnaW5hbCBkYXRhDQogICAgZGF0YSA8LSBtZXJnZShkYXRhLCBkYXRhLnN1YmpNZWFuKQ0KDQogICAgIyBHZXQgdGhlIG5vcm1hbGl6ZWQgZGF0YSBpbiBhIG5ldyBjb2x1bW4NCiAgICBtZWFzdXJlTm9ybWVkVmFyIDwtIHBhc3RlKG1lYXN1cmV2YXIsICJfbm9ybSIsIHNlcCA9ICIiKQ0KICAgIGRhdGFbLG1lYXN1cmVOb3JtZWRWYXJdIDwtIGRhdGFbLG1lYXN1cmV2YXJdIC0gZGF0YVssInN1YmpNZWFuIl0gKw0KICAgICAgICAgICAgICAgICAgICAgICAgICAgICAgIG1lYW4oZGF0YVssbWVhc3VyZXZhcl0sIG5hLnJtID0gbmEucm0pDQoNCiAgICAjIFJlbW92ZSB0aGlzIHN1YmplY3QgbWVhbiBjb2x1bW4NCiAgICBkYXRhJHN1YmpNZWFuIDwtIE5VTEwNCg0KICAgIHJldHVybihkYXRhKQ0KfQ0KYGBgDQoNCiMjIyBteUNlbnRlcg0KDQpUaGlzIGZ1bmN0aW9uIG91dHB1dHMgdGhlIGNlbnRlcmVkIHZhbHVlcyBvZiBhIHZhcmlhYmxlLCB3aGljaCBjYW4gYmUgYSBudW1lcmljIHZhcmlhYmxlLCBhIGZhY3Rvciwgb3IgYSBkYXRhIGZyYW1lLiBJdCB3YXMgdGFrZW4gZnJvbQ0KRmxvcmlhbiBKYWVnZXIncyBibG9nIC0gaHR0cHM6Ly9obHBsYWIud29yZHByZXNzLmNvbS8yMDA5LzA0LzI3L2NlbnRlcmluZy1zZXZlcmFsLXZhcmlhYmxlcy8uDQoNCkZyb20gaGlzIGJsb2c6DQoNCi0gSWYgdGhlIGlucHV0IGlzIGEgbnVtZXJpYyB2YXJpYWJsZSwgdGhlIG91dHB1dCBpcyB0aGUgY2VudGVyZWQgdmFyaWFibGUNCg0KLSBJZiB0aGUgaW5wdXQgaXMgYSBmYWN0b3IsIHRoZSBvdXRwdXQgaXMgYSBudW1lcmljIHZhcmlhYmxlIHdpdGggY2VudGVyZWQgZmFjdG9yIGxldmVsIHZhbHVlcy4gVGhhdCBpcywgdGhlIGZhY3RvcidzIGxldmVscyBhcmUgY29udmVydGVkIGludG8gbnVtZXJpY2FsIHZhbHVlcyBpbiB0aGVpciBpbmhlcmVudCBvcmRlciAoaWYgbm90IHNwZWNpZmllZCBvdGhlcndpc2UsICBSIGRlZmF1bHRzIHRvIGFscGhhbnVtZXJpY2FsIG9yZGVyKS4gTW9yZSBzcGVjaWZpY2FsbHksIHRoaXMgY2VudGVycyBhbnkgYmluYXJ5IGZhY3RvciBzbyB0aGF0IHRoZSB2YWx1ZSBiZWxvdyAwIHdpbGwgYmUgdGhlIDFzdCBsZXZlbCBvZiB0aGUgb3JpZ2luYWwgZmFjdG9yLCBhbmQgdGhlIHZhbHVlIGFib3ZlIDAgd2lsbCBiZSB0aGUgMm5kIGxldmVsLg0KDQotIElmIHRoZSBpbnB1dCBpcyBhIGRhdGEgZnJhbWUgb3IgbWF0cml4LCB0aGUgb3V0cHV0IGlzIGEgbmV3IG1hdHJpeCBvZiB0aGUgc2FtZSBkaW1lbnNpb24gYW5kIHdpdGggdGhlIGNlbnRlcmVkIHZhbHVlcyBhbmQgY29sdW1uIG5hbWVzIHRoYXQgY29ycmVzcG9uZCB0byB0aGUgY29sbmFtZXMoKSBvZiB0aGUgaW5wdXQgcHJlY2VkZWQgYnkgImMiIChlLmcuICJWYXJpYWJsZTEiIHdpbGwgYmUgImNWYXJpYWJsZTEiKS4NCg0KYGBge3J9DQpteUNlbnRlciA9IGZ1bmN0aW9uKHgpIHsNCiAgaWYgKGlzLm51bWVyaWMoeCkpIHsgcmV0dXJuKHggLSBtZWFuKHgsIG5hLnJtID0gVCkpIH0NCglpZiAoaXMuZmFjdG9yKHgpKSB7DQoJCXg9IGFzLm51bWVyaWMoeCkNCgkJcmV0dXJuKHggLSBtZWFuKHgsIG5hLnJtID0gVCkpDQoJfQ0KCWlmIChpcy5kYXRhLmZyYW1lKHgpIHx8IGlzLm1hdHJpeCh4KSkgew0KCQltPSBtYXRyaXgobnJvdyA9IG5yb3coeCksIG5jb2wgPSBuY29sKHgpKQ0KCQljb2xuYW1lcyhtKT0gcGFzdGUoImMiLCBjb2xuYW1lcyh4KSwgc2VwID0gIiIpDQoJDQoJCWZvciAoaSBpbiAxOm5jb2woeCkpIHsNCgkJDQoJCQltWyxpXSA9IG15Q2VudGVyKHhbLGldKQ0KCQl9DQoJCXJldHVybihhcy5kYXRhLmZyYW1lKG0pKQ0KCX0NCn0NCmBgYA0KDQojIyMgbGl6Q2VudGVyDQoNClRoaXMgZnVuY3Rpb24gcHJvdmlkZXMgYSB3cmFwcGVyIGFyb3VuZCBteUNlbnRlciBhbGxvd2luZyB5b3UgdG8gY2VudGVyIGEgc3BlY2lmaWMgbGlzdCBvZiB2YXJpYWJsZXMgZnJvbSBhIGRhdGEgZnJhbWUuIA0KDQotIHg6IGRhdGEgZnJhbWUNCi0gbGlzdGZuYW1lOiBhIGxpc3Qgb2YgdGhlIHZhcmlhYmxlcyB0byBiZSBjZW50ZXJlZCAoZS5nLiBsaXN0KHZhcmlhYmxlMSwgdmFyaWFibGUyKSkNCg0KVGhlIG91dHB1dCBpcyBhIGNvcHkgb2YgdGhlIGRhdGEgZnJhbWUgd2l0aCBhIGNvbHVtbiAoYWx3YXlzIGEgbnVtZXJpYyB2YXJpYWJsZSkgYWRkZWQgZm9yIGVhY2ggb2YgdGhlIGNlbnRlcmVkIHZhcmlhYmxlcy4gVGhlc2UgY29sdW1ucyBhcmUgbGFiZWxsZWQgd2l0aCB0aGUgZWFjaCBjb2x1bW4ncyBwcmV2aW91cyBuYW1lLCBidXQgd2l0aCAiLmN0IiBhcHBlbmRlZCAoZS5nLiwgInZhcmlhYmxlMSIgd2lsbCBiZWNvbWUgInZhcmlhYmxlMS5jdCIpLg0KDQoNCmBgYHtyfQ0KbGl6Q2VudGVyID0gZnVuY3Rpb24oeCwgbGlzdGZuYW1lKSANCnsNCglmb3IgKGkgaW4gMTpsZW5ndGgobGlzdGZuYW1lKSkgDQoJew0KCQlmbmFtZSA9IGFzLmNoYXJhY3RlcihsaXN0Zm5hbWVbaV0pDQoJCXhbcGFzdGUoZm5hbWUsIi5jdCIsIHNlcD0iIildID0gbXlDZW50ZXIoeFtmbmFtZV0pDQoJfQ0KCQkNCglyZXR1cm4oeCkNCn0NCmBgYAkNCg0KIyMjIGxpekNvbnRyYXN0cw0KDQpUaGlzIGZ1bmN0aW9uIGNhbiBiZSB1c2VkIHRvIGNyZWF0ZSB0d28gY2VudGVyZWQgZHVtbXkgdmFyaWFibGVzIHdoaWNoIHN0YW5kIGluIHBsYWNlIG9mIGEgdGhyZWUtd2F5IGZhY3RvciAoY29uZGl0aW9uKS4gVGhpcyBhbGxvd3MgdXMgdG8gaW5zcGVjdCBlYWNoIGNvbnRyYXN0IHNlcGFyYXRlbHksIGFzIHdlbGwgYXMgdGhlaXIgaW50ZXJhY3Rpb25zIHdpdGggb3RoZXIgZmFjdG9ycy4gT3RoZXIgZml4ZWQgZWZmZWN0cyBpbiB0aGUgbW9kZWwgY2FuIGJlIGV2YWx1YXRlZCBhcyB0aGUgYXZlcmFnZSBlZmZlY3RzIGFjcm9zcyBhbGwgbGV2ZWxzIG9mIHRoZSBmYWN0b3IuDQoNClRoZSBmdW5jdGlvbiB0YWtlcyBhIGRhdGEgZnJhbWUgKGQpLCBhIGZhY3RvciBmcm9tIHRoYXQgZGF0YWJhc2UgKGNvbmRpdGlvbiksIHdoaWNoIG11c3QgaGF2ZSB0aHJlZSBsZXZlbHMsIGFuZCB0aGUgbmFtZSBvZiB0aGUgbGV2ZWwgb2YgdGhlIGZhY3RvciB3aGljaCBpcyB0byBiZSB1c2VkIGFzIHRoZSBiYXNlbGluZSBmb3IgdGhlIGNvbnRyYXN0cyAoYmFzZSBsZXZlbCkuDQoNCkZvciBleGFtcGxlLCBpZiBkIGlzIGEgZGF0YSBmcmFtZSB3aXRoIGEgZmFjdG9yICJjb25kaXRpb24iIHdpdGggdGhyZWUgbGV2ZWxzICIoImxleF9za2V3IiwgImxleF9ub3NrZXciLCBhbmQgICJtaXhlZCIpIHRoZW4gbGl6Q29udHJhc3RzKGQsIGQkY29uZGl0aW9uLCAibGV4X25vX3NrZXciKSByZXR1cm5zIGEgZGF0YSBmcmFtZSB3aXRoIHR3byAobnVtZXJpYykgY29sdW1ucyBhZGRlZCBsYWJlbGxlZCAgImxleF9ub3NrZXdfVkVSU1VTX2xleF9taXhlZCIgYW5kICJsZXhfbm9za2V3X1ZFUlNVU19sZXhfc2tldyIuIA0KDQpXaGVyZXZlciB5b3Ugd291bGQgbm9ybWFsbHkgdXNlICJjb25kaXRpb24iaW4gYSBmb3JtdWxhIGluIGFuIExNRSwgaXQgY2FuIGJlIHJlcGxhY2VkIGJ5IChsZXhfbm9za2V3X1ZFUlNVU19sZXhfbWl4ZWQgKyAibGV4X25vc2tld19WRVJTVVNfbGV4X3NrZXcpIGUuZy4gfiAoYSAqIGNvbmRpdGlvbikgYmVjb21lcyB+IChhICogIChsZXhfbm9za2V3X1ZFUlNVU19sZXhfbWl4ZWQgKyBsZXhfbm9za2V3X1ZFUlNVU19sZXhfc2tldykpLg0KDQpgYGB7cn0NCmxpekNvbnRyYXN0cyA9IGZ1bmN0aW9uKGQsIGNvbmRpdGlvbiwgYmFzZWxldmVsKSANCnsNCiAgDQoJY29uZGl0aW9uID0gZmFjdG9yKGNvbmRpdGlvbikNCiAgY29uZGl0aW9uID0gcmVsZXZlbChjb25kaXRpb24sIGJhc2VsZXZlbCkNCg0KCWEgPSAoY29udHJhc3RzKGNvbmRpdGlvbiktYXBwbHkoY29udHJhc3RzKGNvbmRpdGlvbiksMixtZWFuKSkNCglkJGR1bW15MVtjb25kaXRpb24gPT0gcm93bmFtZXMoYSlbMV1dIDwtIGFbMV0gDQoJZCRkdW1teTFbY29uZGl0aW9uID09IHJvd25hbWVzKGEpWzJdXSA8LSBhWzJdIA0KCWQkZHVtbXkxW2NvbmRpdGlvbiA9PSByb3duYW1lcyhhKVszXV0gPC0gYVszXSANCglkJGR1bW15Mltjb25kaXRpb24gPT0gcm93bmFtZXMoYSlbMV1dIDwtIGFbNF0gDQoJZCRkdW1teTJbY29uZGl0aW9uID09IHJvd25hbWVzKGEpWzJdXSA8LSBhWzVdIA0KCWQkZHVtbXkyW2NvbmRpdGlvbiA9PSByb3duYW1lcyhhKVszXV0gPC0gYVs2XSANCg0KCW5hbWUxID0gcGFzdGUoYmFzZWxldmVsLCByb3duYW1lcyhhKVsyXSxzZXAgPSJfVkVSU1VTXyIpDQoJbmFtZTIgPSBwYXN0ZShiYXNlbGV2ZWwsIHJvd25hbWVzKGEpWzNdLHNlcCA9Il9WRVJTVVNfIikNCg0KCWRbbmFtZTFdID0gZCRkdW1teTEJDQoJZFtuYW1lMl0gPSBkJGR1bW15MgkNCg0KCWQkZHVtbXkxIDwtTlVMTAkNCglkJGR1bW15MiA8LU5VTEwJDQoJDQoJcmV0dXJuKGQpDQp9DQpgYGAgIA0KDQojIyMgZmlsdGVyMg0KDQpUaGlzIGlzIGEgZnVuY3Rpb24gd2hpY2ggZmlsdGVycyBhIGNvbHVtbiBvZiBkYXRhIHJlbW92aW5nIHZhbHVlcyB3aGljaCBhIGNlcnRhaW4gbnVtYmVyIG9mIHN0YW5kYXJkIGRldmlhdGlvbnMgYWJvdmUvYmVsb3cgdGhlIG1lYW4gZm9yIHRoYXQgcGFydGljaXBhbnQsIHBvc3NpYmx5IGluIHNvbWUgY29uZGl0aW9uL3N1Yi1jb25kaXRpb24uDQoNCi0gaW06IHRoZSBpbnB1dCBtYXRyaXggKGEgZGF0YSBmcmFtZSkNCi0gc3ZuOiBhIGxpc3Qgb2YgdGhlIG5hbWVzIG9mIGZhY3RvcnMgdG8gYmUgZ3JvdXAgYnkgKHN1YmplY3QgbmFtZSArIG9uZSBvciBtb3JlIGNvbmRpdGlvbnMpDQotIGZuOiB0aGUgbmFtZSBvZiB0aGUgY29sdW1uIGNvbnRhaW5pbmcgdGhlIGRhdGEgdG8gYmUgZmlsdGVyZWQNCi0gbGltOiBob3cgbWFueSBzdGFuZGFyZCBkZXZpYXRpb25zIGFib3ZlL2JlbG93IHRoZSBtZWFuIHRvIGZpbHRlcg0KDQpUaGUgZnVuY3Rpb24gcmV0dXJucyBhbiBpbnB1dCBtYXRyaXggaWRlbnRpY2FsIHRvIHRoZSBvcmlnaW5hbCBpbnB1dCBtYXRyaXggYnV0IHdpdGggYWRkaXRpb25hbCBjb2x1bW5zIGdpdmluZyB0aGUgZ3JvdXAgbWVhbnMgYW5kIHRoZSAiZmlsdGVyZWQiIGRhdGENCg0KYGBge3J9DQpmaWx0ZXIyID0gZnVuY3Rpb24oaW0sIHN2biwgZm4sIGxpbSkNCnsNCiAgIyMgd29yayBvdXQgbWVhbnMgZm9yIGVhY2ggc3ViamVjdCBmb3IgZWFjaCB3b3JkDQoNCnggPSBsaXN0KCkNCnkgPSAiIg0KDQpmb3IobiBpbiBzdm4pIHggPSBhcHBlbmQoaW1bbl0seCkNCmZvcihuIGluIHN2bikgeSA9IHBhc3RlKHksbixzZXAgPSAiXyIpDQoNCm1lYW5zID0gYWdncmVnYXRlKGltW2ZuXSwgYnkgPSB4LCBtZWFuLCBuYS5ybSA9IFQpDQpoZWFkKG1lYW5zKQ0Kbm9jb2xzID0gZGltKG1lYW5zKVsyXQ0KY29sbmFtZXMobWVhbnMpW25vY29sc10gPSAibWVhbnMiDQoNCnNkcyA9IGFnZ3JlZ2F0ZShpbVtmbl0sIGJ5ID0geCwgc2QsIG5hLnJtID0gVCkNCmhlYWQoc2RzKQ0Kbm9jb2xzID0gZGltKHNkcylbMl0NCmNvbG5hbWVzKHNkcylbbm9jb2xzXSA9ICJzZHMiDQoNCmdzID0gbWVyZ2UobWVhbnMsc2RzKQ0KDQojIyBiZWNhdXNlIGlmIHRoZXJlIGlzIGp1c3Qgb25lIHZhbHVlIGl0IGRvZXMgbm90IGhhdmUgYSBzdGFuZGFyZCBkZXZpYXRpb24gYW5kIHdlIGRvIG5vdCB3YW50IHRvIGp1c3QgZGlzcmVnYXJkIGFsbCBvZiB0aGVzZQ0KZ3Mkc2RzW2lzLm5hKGdzJHNkcyldID0gMCANCg0KZ3MkbWF4ID0gZ3MkbWVhbnMgKyBsaW0qZ3Mkc2RzDQpncyRtaW4gPSBncyRtZWFucyAtIGxpbSpncyRzZHMNCg0KaW0yID0gbWVyZ2UoaW0sIGdzLCBzb3J0ID0gRikNCg0KaW0yW3Bhc3RlKGZuLCAiZmlsdCIsIHNlcCA9ICIiKV0gPSBpbTJbZm5dDQpjbiA9IGRpbShpbTIpWzJdICMjIGdldCBjb2xudW1iZXIgKGxhc3Qgb25lIGFkZGVkKQ0KDQppbTJbLGNuXVtpbTJbLGZuXSA+IGltMiRtYXhdID0gIiINCg0KaW0yWyxjbl1baW0yWyxmbl0gPCBpbTIkbWluXSA9ICIiDQoNCmltMlssY25dID0gYXMubnVtZXJpYyhpbTJbLGNuXSkNCg0KbmFtZXMoaW0yKVtuYW1lcyhpbTIpID09ICJtZWFucyJdID0gcGFzdGUoIm1lYW4iLCB5LCBzZXAgPSAiXyIpIA0KbmFtZXMoaW0yKVtuYW1lcyhpbTIpID09InNkcyJdID0gcGFzdGUoInNkIiwgeSwgc2VwID0gIl8iKSANCm5hbWVzKGltMilbbmFtZXMoaW0yKSA9PSJtYXgiXSA9IHBhc3RlKCJtYXgiLCB5LCBzZXAgPSAiXyIpIA0KbmFtZXMoaW0yKVtuYW1lcyhpbTIpID09Im1pbiJdID0gcGFzdGUoIm1pbiIsIHksIHNlcCA9ICJfIikgDQoNCnJldHVybihpbTIpDQp9DQpgYGAgIA0KDQojIyMgZ2V0X2NvZWZmcw0KDQpUaGlzIGZ1bmN0aW9uIGFsbG93cyB1cyB0byBpbnNwZWN0IHBhcnRpY3VsYXIgY29lZmZpY2llbnRzIGZyb20gdGhlIG91dHB1dCBvZiBhbiBMTUUgbW9kZWwgYnkgcHV0dGluZyB0aGVtIGluIHRhYmxlLg0KDQotIHg6IHRoZSBvdXRwdXQgcmV0dXJuZWQgd2hlbiBydW5uaW5nIGxtZXIgb3IgZ2xtZXIgKGkuZS4gYW4gb2JqZWN0IG9mIHR5cGUgbG1lck1vZCBvciBnbG1lck1vZCkNCi0gbGlzdDogYSBsaXN0IG9mIHRoZSBuYW1lcyBvZiB0aGUgY29lZmZpY2llbnRzIHRvIGJlIGV4dHJhY3RlZCAoZS5nLiBjKCJ2YXJpYWJsZTEiLCAidmFyaWFibGUxOnZhcmlhYmxlMiIpKSANCg0KYGBge3J9DQpnZXRfY29lZmZzIDwtIGZ1bmN0aW9uKHgsbGlzdCl7KGFzLmRhdGEuZnJhbWUoc3VtbWFyeSh4KSRjb2VmZmljaWVudHMpW2xpc3QsXSl9DQpgYGAgDQoNCiMgTG9hZCBEYXRhc2V0cw0KDQpgYGB7cn0NCnNldHdkKCJkYXRhZmlsZXMiKQ0KcGNwdCA8LSByZWFkLmNzdigiUENQVC5jc3YiKQ0KY3N0YyA8LXJlYWQuY3N2KCJDU1RDLWN1cnZlLmNzdiIsIHNlcCA9ICIsIiwgc3RyaW5nc0FzRmFjdG9ycyA9IEYpIA0KZGlzIDwtIHJlYWQuY3N2KCJEaXNjcmltaW5hdGlvbi5jc3YiKQ0KdHJhaW4gPC0gcmVhZC5jc3YoIlRyYWluaW5nLmNzdiIpDQpQSSA8LSByZWFkLmNzdigiUGljdHVyZSBJZGVudGlmaWNhdGlvbi5jc3YiKQ0KUFJPIDwtIHJlYWQuY3N2KCJQcm9kdWN0aW9uX2FsbC5jc3YiKQ0Kc2V0d2QoIi4uIikNCmBgYA0KIyBJbmRpdmlkdWFsIEFwdGl0dWRlIFRhc2sgMTogUGl0Y2ggQ29udG91ciBQZXJjZXB0aW9uIFRlc3QgKFNlY3Rpb24gMy4yLjEpDQoNCiMjIEZpZ3VyZSAoRmlndXJlIDMpDQoNCmBgYHtyfQ0KcGNwdCRjb25kaXRpb24gPSBmYWN0b3IocGNwdCRjb25kaXRpb24sIGxldmVscyA9IGMoIjAiLCAiMSIsICIyIiksIGxhYmVscyA9IGMoIkxWIiwgIkhWIiwgIkhWQiIpKQ0KcGNwdCRzZXNzaW9uID0gZmFjdG9yKHBjcHQkc2Vzc2lvbiwgbGV2ZWxzID0gYygiMSIsICIyIiksIGxhYmVscyA9IGMoIlByZS10ZXN0IiwgIlBvc3QtdGVzdCIpKQ0KbWVhbnMgPSB3aXRoKHBjcHQsIGFnZ3JlZ2F0ZShhY2N1cmFjeSB+IHN1YmplY3QgKyBzZXNzaW9uICsgY29uZGl0aW9uLCBGVU4gPSBtZWFuKSkNCg0KeCA8LSBzdW1tYXJ5U0V3aXRoaW4obWVhbnMsIG1lYXN1cmV2YXIgPSAiYWNjdXJhY3kiLCBiZXR3ZWVudmFycyA9IGMoImNvbmRpdGlvbiIpLCB3aXRoaW52YXJzID0gYygic2Vzc2lvbiIpLCBpZHZhciA9ICJzdWJqZWN0IiwgbmEucm0gPSBGQUxTRSwgY29uZi5pbnRlcnZhbCA9IC45NSkNCmBgYA0KDQpgYGB7cn0NCnAxID0gZ2dwbG90IChtZWFucywgYWVzKHggPSBjb25kaXRpb24sIHkgPSBhY2N1cmFjeSwgZmlsbCA9IHNlc3Npb24pKSANCnAxID0gcDEgKyBnZW9tX3Zpb2xpbihjb2xvdXIgPSAiYmxhY2siLCBzY2FsZSA9ICJjb3VudCIpDQpwMSA9IHAxICsgc2NhbGVfZmlsbF9tYW51YWwobmFtZSA9ICJUZXN0IFNlc3Npb24iLCB2YWx1ZXMgPSBjKCJQcmUtdGVzdCIgPSAiZ3JleSIsICJQb3N0LXRlc3QiID0gIndoaXRlIiksIGxhYmVscyA9IGMoIlByZS10ZXN0IiwgIlBvc3QtdGVzdCIpKQ0KcDEgPSBwMSArIHNjYWxlX3hfZGlzY3JldGUobGFiZWxzID0gYygiTFYiLCAiSFYiLCAiSFZCIikpDQpwMSA9IHAxICsgZ2VvbV9lcnJvcmJhcihhZXMoeW1pbiA9IGFjY3VyYWN5LWNpLCB5bWF4ID0gYWNjdXJhY3krY2kpLCB3aWR0aCA9IC40LCBwb3NpdGlvbiA9IHBvc2l0aW9uX2RvZGdlKC45KSwgZGF0YSA9IHgpDQpwMSA9IHAxICsgc3RhdF9zdW1tYXJ5KGZ1bi55ID0gbWVhbiwgZ2VvbSA9ICJwb2ludCIsIHNoYXBlID0gMjMsIHNpemUgPSAyLHBvc2l0aW9uID0gcG9zaXRpb25fZG9kZ2UoLjkpKQ0KcDEgPSBwMSArdGhlbWVfYncoKSsgbGFicyh4ID0gIlZhcmlhYmlsaXR5IENvbmRpdGlvbiIsIHkgPSAiTWVhbiBBY2N1cmFjeSIpDQpwcmludChwMSkNCmBgYA0KDQojIyBTdGF0aXN0aWNhbCBBbmFseXNpcyAoU2VjdGlvbiAzLjIuMSkNCg0KDQpgYGB7cn0NCnBjcHQgPSBsaXpDb250cmFzdHMocGNwdCwgcGNwdCRjb25kaXRpb24sICJMViIpDQoNCnBjcHRfZ2xtZXIxID0gZ2xtZXIoYWNjdXJhY3kgfiANCiAgICAgICAgICAgICAgc2Vzc2lvbiAqIChMVl9WRVJTVVNfSFYgKyBMVl9WRVJTVVNfSFZCKQ0KICAgICAgICAgICAgICArIChzZXNzaW9ufHN1YmplY3QpLA0KICAgICAgICAgICAgICBmYW1pbHkgPSAiYmlub21pYWwiLCANCiAgICAgICAgICAgICAgY29udHJvbCA9IGdsbWVyQ29udHJvbChvcHRpbWl6ZXIgPSAiYm9ieXFhIiksIA0KICAgICAgICAgICAgICBkYXRhID0gcGNwdCkNCmBgYA0KDQoNCmBgYHtyfQ0Ka2FibGUgKHJvdW5kKHN1bW1hcnkocGNwdF9nbG1lcjEpJGNvZWZmaWNpZW50cywgNiksIGRpZ2l0cyA9IDMpDQpgYGANCg0KDQpgYGB7cn0NCmRhdGFwY3B0IDwtIHN1bW1hcnlTRXdpdGhpbihtZWFucywgbWVhc3VyZXZhciA9ICJhY2N1cmFjeSIsIHdpdGhpbnZhcnMgPSBjKCJzZXNzaW9uIiksIGlkdmFyID0gInN1YmplY3QiLCBuYS5ybSA9IEZBTFNFLCBjb25mLmludGVydmFsID0gLjk1KQ0Ka2FibGUoZGF0YXBjcHQsIGRpZ2l0cyA9IDMpDQpgYGANCg0KIyBJbmRpdmlkdWFsIEFwdGl0dWRlIFRhc2sgMjogQ2F0ZWdvcmlzYXRpb24gb2YgU3ludGhlc2l6ZWQgVG9uYWwgQ29udGludWEgKFNlY3Rpb24gMy4yLjIpDQoNCldlIGFuYWx5c2VkIGRhdGEgZnJvbSB0aGUgQ1NUQyB0YXNrIGFkYXB0ZWQgZnJvbSBTYWRha2F0YSBhbmQgTWNRdWVlbiAoMjAxNCkuIFdlIGZpcnN0IGF0dGVtcHRlZCB0byBmb2xsb3cgdGhlIHByb2NlZHVyZXMgaW4gdGhlaXIgcGFwZXIuIFNwZWNpZmljYWxseSwgdG8gcXVhbnRpZnkgcGVyZm9ybWFuY2UgaW4gdGhpcyB0YXNrLCBlYWNoIHN1YmplY3RzJyBjYXRlZ29yaXphdGlvbiBjdXJ2ZSB3YXMgZml0dGVkIHRvIGEgbG9naXN0aWMgZnVuY3Rpb24gdXNpbmcgdGhlIExvZ2lzdGljIEN1cnZlIEZpdCBmdW5jdGlvbiBpbiBTUFNTIGFuZCBhIHNsb3BlIGNvZWZmaWNpZW50IHdhcyBjYWxjdWxhdGVkIChKb2FuaXNzZSwgTWFuaXMsIEtlYXRpbmcsICYgU2VpZGVuYmVyZywgMjAwMCkgd2hpY2ggd2FzIHRha2VuIHRvIGluZGljYXRlIHRoZSBwYXJ0aWNpcGFudHMgYWJpbGl0eSB0byBjYXRlZ29yaXplIHRoZSB0d28gdG9uZXMsIHdpdGggc21hbGxlciBzbG9wZXMgaW5kaWNhdGluZyBiZXR0ZXIgcGVyZm9ybWFuY2UgKHBlcmZlY3Qgc2xvcGUgPSAwLjA0MikuIFNhZGFrYXRhIGFuZCBNY1F1ZWVuICgyMDE0KSwgcmVwb3J0IHRoYXQgdGhleSByZW1vdmVkIGFueSBwYXJ0aWNpcGFudCB3aXRoIGEgc2xvcGUgbWVhc3VyZSBncmVhdGVyIHRoYW4gMS4yIGZyb20gdGhlIGFuYWx5c2lzLCBzdWdnZXN0aW5nIHRoYXQgc2xvcGVzIGFib3ZlIHRoaXMgdGhyZXNob2xkIHdlcmUgY29uc2lkZXJlZCB0byBiZSBwb29ybHkgZml0LiBIb3dldmVyIGZvbGxvd2luZyB0aGlzIHByb2Nlc3Mgd2l0aCBvdXIgcGFydGljaXBhbnRzLCB0aGUgbWFqb3JpdHkgd2VyZSBhYm92ZSB0aGlzIHRocmVzaG9sZCAoNDMgb3V0IG9mIDYwKS4gR2l2ZW4gdGhpcywgd2UgYXR0ZW1wdGVkIGFuIGFsdGVybmF0aXZlIG1ldGhvZCBvZiBkZXJpdmluZyBzbG9wZSBjb2VmZmljaWVudHMgZm9yIGVhY2ggcGFydGljaXBhbnQgdXNpbmcgYSBsb2dpc3RpYyBtaXhlZCBlZmZlY3QgbW9kZWwgKFNjaHVsdHosIExsYW5vcywgJiBGcmFuY2lzLCAyMDAzKSB3aXRoIHRoZSBwcmVkaWN0ZWQgdmFyaWFibGUgYmVpbmcgd2hpY2ggb2YgdHdvIHRvbmVzIHRoZSBwYXJ0aWNpcGFudHMgY2hvc2Ugb24gZWFjaCB0cmlhbCBhbmQgdGhlIHByZWRpY3RvciBiZWluZyB0aGUgdG9uZSBzdGVwIHByZXNlbnRlZCBvbiBlYWNoIHRyaWFsICh2YXJ5aW5nIGZyb20gMS02IHdoZXJlIDEgaXMgbW9zdCBsaWtlIFRvbmUgMiwgYW5kIDYgaXMgbW9zdCBsaWtlIFRvbmUgMykuIFJhbmRvbSBpbnRlcmNlcHRzIGFuZCBzbG9wZXMgZm9yIHRvbmUgc3RlcCB3ZXJlIGZpdCBieSBwYXJ0aWNpcGFudCBhbmQgdGhlIGluZGl2aWR1YWwgc2xvcGUgY29lZmZpY2llbnRzIGZvciBlYWNoIHBhcnRpY2lwYW50IHdlcmUgZXh0cmFjdGVkIGZyb20gdGhlIGJ5LXBhcnRpY2lwYW50IHJhbmRvbSBzbG9wZXMgZml0IGluIHRoZSBtb2RlbC4gQmVjYXVzZSB0aGUgcmFuZG9tIHNsb3BlcyByZXByZXNlbnQgYWRqdXN0bWVudHMgdG8gdGhlIGZpeGVkIGVmZmVjdCBzbG9wZSwgbW9yZSBwb3NpdGl2ZSBzbG9wZXMgcmVwcmVzZW50IHNoYXJwZXIgY2F0ZWdvcml6YXRpb24gcmVzcG9uc2VzLCBpLmUuIG1vcmUgc2Vuc2l0aXZpdHkgdG8gZGlmZmVyZW5jZXMgaW4gdG9uZSBzdGVwLCB3aGlsZSBtb3JlIG5lZ2F0aXZlIHNsb3BlcyByZXByZXNlbnQgZmxhdHRlciBjYXRlZ29yaXphdGlvbiByZXNwb25zZXMsIG9yIGluIGV4dHJlbWUgY2FzZXMgcmV2ZXJzZWQgcmVzcG9uc2VzLCBhbmQgc2xvcGVzIGNsb3NlIHRvIDAgcmVmbGVjdCByZXNwb25zZXMgY2xvc2UgdG8gdGhlIG1lYW4uIFRoZXNlIHNsb3BlcyBjb3VsZCB0aHVzIGJlIHRha2VuIHRvIGJlIGluZGljYXRvcnMgb2YgaW5kaXZpZHVhbCBkaWZmZXJlbmNlcy4gV2hlbiB3ZSBjb21wYXJlZCB0aGVzZSBzbG9wZXMgdG8gdGhlIG9uZXMgZGVyaXZlZCBmcm9tIFNQU1Mgd2UgZm91bmQgdGhhdCB0aGV5IHdlcmUgdmVyeSBzaW1pbGFyLiBUaGUgc2FtZSBwYXJ0aWNpcGFudHMgd2hvIGZhaWxlZCB0aGUgY3JpdGVyaWEgb2YgU2FkYWthdGEgYW5kIE1jUXVlZW4gYWxzbyBoYWQgdmVyeSBzaGFsbG93IHNsb3BlcyB1c2luZyB0aGUgbG9naXN0aWMgcmVncmVzc2lvbiBtZXRob2QuIFRoZSBhbmFseXNlcyBjb25kdWN0ZWQgaW4gdGhpcyBzY3JpcHQgd2VyZSBkb25lIHdpdGggdGhlIGluZGl2aWR1YWwgZGlmZmVyZW5jZSBtZWFzdXJlIGV4dHJhY3RlZCBmcm9tIHRoZSBsb2dpc3RpYyByZWdyZXNzaW9uLg0KDQpUaGlzIGZpZ3VyZSBzaG93cyB0aGUgaW5kaXZpZHVhbCBjdXJ2ZXMgZm9yIGVhY2ggZ3JvdXAuIEl0IGFsc28gc2hvd3MgdGhlIHJhdyBkYXRhIHRoYXQgdGhlIHNsb3BlcyBhcmUgYmFzZWQgb24uDQoNCmBgYHtyfQ0KY3N0YyRzZXNzaW9uID0gZmFjdG9yKGNzdGMkc2Vzc2lvbiwgbGV2ZWxzID0gYygiMSIsIjIiKSwgbGFiZWxzID0gYygiUHJlLXRlc3QiLCAiUG9zdC10ZXN0IikpDQpjc3RjJGNvbmRpdGlvbiA9IGZhY3Rvcihjc3RjJGNvbmRpdGlvbiwgbGV2ZWxzID0gYygiMCIsICIxIiwgIjIiKSwgbGFiZWxzID0gYygiTFYiLCAiSFYiLCAiSFZCIikpDQoNCiMgVHdvIHBhcnRpY2lwYW50cyBzaG93ZWQgdGhlIGNvbXBsZXRlbHkgcmV2ZXJzZWQgcGF0dGVybi4gSGVyZSB3ZSByZXZlcnNlIHRoZSBjb2Rpbmcgb2YgdGhlc2UgMiBwYXJ0aWNpcGFudHMNCmNzdGMkY2F0ZWdvcnlbY3N0YyRzdWJqZWN0ID09ICI1Inxjc3RjJHN1YmplY3QgPT0gIjM2Il0gPC0gYWJzKGNzdGMkY2F0ZWdvcnlbY3N0YyRzdWJqZWN0ID09ICI1Inxjc3RjJHN1YmplY3QgPT0gIjM2Il0gLSAxKQ0KDQpnZ3Bsb3QoY3N0YywgYWVzKHggPSBzdGVwLCB5ID0gY2F0ZWdvcnkpKSArIA0KICBzdGF0X3Ntb290aChhZXMoZ3JvdXAgPSBzdWJqZWN0KSwgbWV0aG9kID0gImdsbSIsIG1ldGhvZC5hcmdzID0gbGlzdChmYW1pbHkgPSAiYmlub21pYWwiKSwgc2l6ZSA9IDAuNSwgc2UgPSBGQUxTRSwgc2hvdy5sZWdlbmQgPSBGQUxTRSwgY29sb3IgPSAiI2NjZTJlZiIpICsgIyBpbmRpdmlkdWFsIHJlZ3Jlc3Npb24gbGluZXMNCiAgc3RhdF9zbW9vdGgobWV0aG9kID0gImdsbSIsIG1ldGhvZC5hcmdzID0gbGlzdChmYW1pbHkgPSAiYmlub21pYWwiKSwgc2l6ZSA9IDIsIHNlID0gRkFMU0UsIHNob3cubGVnZW5kID0gRkFMU0UsIGNvbG9yID0gIiNCMDEzMjYiKSArICMgZ3JvdXAgbWVhbiByZWdyZXNzaW9uIGxpbmUNCiAgeWxpbSgwLCAxKSArIHNjYWxlX3hfY29udGludW91cyhicmVha3MgPSAxOjcpICsgZmFjZXRfZ3JpZCh+Y29uZGl0aW9uKSArDQogIHhsYWIoInRvbmUgc3RlcCIpICsgeWxhYigiUHJvcG9ydGlvbiBUb25lIFggcmVzcG9uc2VzIikgKyB0aGVtZV9idygpICsNCiAgdGhlbWUocGxvdC50aXRsZSA9IGVsZW1lbnRfdGV4dChoanVzdCA9IDAuNSwgZmFjZSA9ICJib2xkIikpDQpgYGANCg0KIyMgRXh0cmFjdCBzbG9wZSBjb2ZmY2llbnQgZm9yIGVhY2ggcGFydGljaXBhbnQgYW5kIGRlc2NyaXB0aXZlcw0KDQpgYGB7cn0NCmNzdGMkc2Vzc2lvbiA8LSBhcy5udW1lcmljKGNzdGMkc2Vzc2lvbikNCmNzdGMkc2Vzc2lvbi5zdGQgPC0gc2NhbGUoY3N0YyRzZXNzaW9uKQ0KY3N0YyRzdGVwLnN0ZCA8LSBzY2FsZShjc3RjJHN0ZXApDQpmMSA8LSAgZmFjdG9yKGMoIkhWIiwgIkxWIikpDQpkZjEgPC0gZGF0YS5mcmFtZShmMSwgc3ViamVjdCA9IDE6NDApDQpmMSA8LSAgZmFjdG9yKCJIVkIiKQ0KZGYyIDwtIGRhdGEuZnJhbWUoZjEsIHN1YmplY3QgPSA0MTo2MCkNCmRmMyA8LSByYmluZC5maWxsKGRmMSwgZGYyKQ0KDQpsbWZpdHNfcHJlIDwtIGdsbWVyKGNhdGVnb3J5IH4gc3RlcC5zdGQgKyAoc3RlcC5zdGR8c3ViamVjdCksIGRhdGEgPSBjc3RjW2NzdGMkc2Vzc2lvbiA9PSAxLF0sIGZhbWlseSA9ICJiaW5vbWlhbCIsIGNvbnRyb2wgPSBnbG1lckNvbnRyb2wob3B0aW1pemVyID0gImJvYnlxYSIpKQ0KcHJlID0gcmFuZWYobG1maXRzX3ByZSkkc3ViamVjdA0KcHJlID0gY2JpbmQgKHByZSwgZGYzKQ0KcHJlJHNlc3Npb24gPSAiUHJlLXRlc3QiDQoNCmxtZml0c19wb3N0IDwtIGdsbWVyKGNhdGVnb3J5IH4gc3RlcC5zdGQgKyAoc3RlcC5zdGR8c3ViamVjdCksIGRhdGEgPSBjc3RjW2NzdGMkc2Vzc2lvbiA9PSAyLF0sIGZhbWlseSA9ICJiaW5vbWlhbCIsIGNvbnRyb2wgPSBnbG1lckNvbnRyb2wob3B0aW1pemVyID0gImJvYnlxYSIpKQ0KcG9zdCA9IHJhbmVmKGxtZml0c19wb3N0KSRzdWJqZWN0DQpwb3N0ID0gY2JpbmQgKHBvc3QsIGRmMykNCnBvc3Qkc2Vzc2lvbiA9ICJQb3N0LXRlc3QiDQpjc3RjMiA8LSByYmluZChwcmUscG9zdCkNCg0KbmFtZXMoY3N0YzIpW25hbWVzKGNzdGMyKSA9PSAic3RlcC5zdGQiXSA8LSAic2xvcGUiDQpuYW1lcyhjc3RjMilbbmFtZXMoY3N0YzIpID09ICIoSW50ZXJjZXB0KSJdIDwtICJpbnRlcmNlcHQiDQpuYW1lcyhjc3RjMilbbmFtZXMoY3N0YzIpID09ICJmMSJdIDwtICJjb25kaXRpb24iDQoNCiMgcmVvcmRlciB0aGUgbGV2ZWxzIHNvIHRoYXQgdGhleSBtYXRjaCBwcmV2aW91cyBkYXRhIHNldA0KY3N0YzIkY29uZGl0aW9uID0gZmFjdG9yIChjc3RjMiRjb25kaXRpb24sIGxldmVscyA9ICBjKCJMViIsIkhWIiwiSFZCIikgKQ0KY3N0YzIkc2Vzc2lvbiA9IGZhY3RvciAoY3N0YzIkc2Vzc2lvbiwgbGV2ZWxzID0gIGMoIlByZS10ZXN0IiwiUG9zdC10ZXN0IikgKQ0KDQptZWFucyA9IHdpdGgoY3N0YzIsIGFnZ3JlZ2F0ZShzbG9wZSB+IHN1YmplY3QgKyBzZXNzaW9uICsgY29uZGl0aW9uLCBGVU4gPSBtZWFuKSkNCnggPC0gc3VtbWFyeVNFd2l0aGluKGNzdGMyLCBtZWFzdXJldmFyID0gInNsb3BlIiwgYmV0d2VlbnZhcnMgPSBjKCJjb25kaXRpb24iKSwgd2l0aGludmFycyA9IGMoInNlc3Npb24iKSwgaWR2YXIgPSAic3ViamVjdCIsIG5hLnJtID0gRkFMU0UsIGNvbmYuaW50ZXJ2YWwgPSAuOTUpDQoNCmthYmxlKHgsIGRpZ2l0cyA9IDIpDQpgYGANCg0KIyMgR3JhcGgNCg0KYGBge3J9DQoNCnAyID0gZ2dwbG90IChtZWFucywgYWVzKHggPSBjb25kaXRpb24sIHkgPSBzbG9wZSwgZmlsbCA9IHNlc3Npb24pKSANCnAyID0gcDIgKyBnZW9tX3Zpb2xpbihjb2xvdXIgPSAiYmxhY2siLCBzY2FsZSA9ICJjb3VudCIpDQpwMiA9IHAyICsgc2NhbGVfZmlsbF9tYW51YWwobmFtZSA9ICJUZXN0IHNlc3Npb24iLCB2YWx1ZXMgPSBjKCJQcmUtdGVzdCIgPSAiZ3JleSIsICJQb3N0LXRlc3QiID0gIndoaXRlIikpDQpwMiA9IHAyICsgc2NhbGVfeF9kaXNjcmV0ZShsYWJlbHMgPSBjKCJMb3cgVmFyaWFiaWxpdHkiLCAiSGlnaCBWYXJpYWJpbGl0eSIsICJIaWdoIFZhcmFpYmlsaXR5IEJsb2NraW5nIikpDQpwMiA9IHAyICsgZ2VvbV9lcnJvcmJhcihhZXMoeW1pbiA9IHNsb3BlLWNpLCB5bWF4ID0gc2xvcGUrY2kpLCB3aWR0aCA9IC40LCBwb3NpdGlvbiA9IHBvc2l0aW9uX2RvZGdlKC45KSwgZGF0YSA9IHgpDQpwMiA9IHAyICsgc3RhdF9zdW1tYXJ5KGZ1bi55ID0gbWVhbiwgZ2VvbSA9ICJwb2ludCIsIHNoYXBlID0gMjMsIHNpemUgPSAyLCBwb3NpdGlvbiA9IHBvc2l0aW9uX2RvZGdlKC45KSkNCnAyID0gcDIgKyB0aGVtZV9idygpDQpwcmludChwMikNCg0Kcm0obWVhbnMpDQpybSh4KQ0KYGBgDQoNCg0KIyMgU3RhdGlzdGljYWwgQW5hbHlzaXMNCg0KYGBge3J9DQpjc3RjMiA9IGxpekNvbnRyYXN0cyhjc3RjMiwgY3N0YzIkY29uZGl0aW9uLCAiTFYiKQ0KY3N0Y19sbSA9IGxtKHNsb3BlIH4gc2Vzc2lvbiAqIChMVl9WRVJTVVNfSFYgKyBMVl9WRVJTVVNfSFZCKSAsIGRhdGEgPSBjc3RjMikNCmBgYA0KDQpMb29rIGF0IGRpZmZlcmVuY2VzIGJldHdlZW4gY29uZGl0aW9ucyBhdCBwcmUtdGVzdDoNCg0KYGBge3J9DQpyb3VuZCAoZ2V0X2NvZWZmcyhjc3RjX2xtLGMoIkxWX1ZFUlNVU19IViIsIkxWX1ZFUlNVU19IVkIiKSksMykNCmBgYA0KDQoNCg0KIyBMb29rIGF0IHRoZSByZWxhdGlvbnNoaXAgYmV0d2VlbiBQQ1BUIGFuZCBDU1RDIGFuZCBzZXQgdXAgSURfcHJlIHRhYmxlDQoNCmBgYHtyfQ0KSURfcHJlID0gbWVyZ2UoDQogIA0KICB3aXRoKHN1YnNldChwY3B0LCBzZXNzaW9uICVpbiUgIlByZS10ZXN0IiksIGFnZ3JlZ2F0ZShhY2N1cmFjeSB+IHN1YmplY3QgKyBzZXNzaW9uICsgY29uZGl0aW9uLCBGVU4gPSBtZWFuKSksDQogIA0KICB3aXRoKHN1YnNldChjc3RjMiwgc2Vzc2lvbiAlaW4lICJQcmUtdGVzdCIpLCBhZ2dyZWdhdGUoc2xvcGUgfiBzdWJqZWN0ICsgc2Vzc2lvbiArIGNvbmRpdGlvbiwgRlVOID0gbWVhbikpLCBieSA9ICJzdWJqZWN0IikNCklEX3ByZSRhcHRpdHVkZV9wY3B0ID0gSURfcHJlJGFjY3VyYWN5ICoxMA0KSURfcHJlJHNlc3Npb24gPSBOVUxMDQpjb3IudGVzdChJRF9wcmUkc2xvcGUsIElEX3ByZSRhY2N1cmFjeSkNCmBgYA0KDQoNCiMjIENvcnJlbGF0aW9uIEZpZ3VyZQ0KDQpgYGB7cn0NCmdncGxvdChJRF9wcmUsIGFlcyh4ID0gYWNjdXJhY3ksIHkgPSBzbG9wZSwgY29sb3IgPSBjb25kaXRpb24ueCkpICsNCmdlb21fcG9pbnQoc2l6ZSA9IDUpIA0KYGBgDQoNCg0KIyMgQ3JlYXRlIGEgbWVkaWFuIHNwbGl0ICh0aGlzIGlzIHVzZWQgbGF0ZXIgaW4gcGxvdHRpbmcsIGJ1dCBub3QgaW4gYW5hbHlzZXMpDQoNCmBgYHtyfQ0KSURfcHJlJGFwdGl0dWRlMiA9IDENCklEX3ByZSRhcHRpdHVkZTJbSURfcHJlJGFjY3VyYWN5IDwgbWVkaWFuKElEX3ByZSRhY2N1cmFjeSldID0gMA0KDQpJRF9wcmUkYXB0aXR1ZGUzID0gMQ0KSURfcHJlJGFwdGl0dWRlM1tJRF9wcmUkc2xvcGUgPCBtZWRpYW4oSURfcHJlJHNsb3BlKV0gPSAwDQoNCklEX3ByZSRhcHRpdHVkZTIgPSBmYWN0b3IoSURfcHJlJGFwdGl0dWRlMiwgbGV2ZWxzID0gYygiMSIsICIwIiksIGxhYmVscyA9IGMgKCJIQSIsICJMQSIpKQ0KSURfcHJlJGFwdGl0dWRlMyA9IGZhY3RvcihJRF9wcmUkYXB0aXR1ZGUzLCBsZXZlbHMgPSBjKCIwIiwgIjEiKSwgbGFiZWxzID0gYyAoIkhBIiwgIkxBIikpDQpgYGANCg0KIyBQcm9kdWN0aW9uIFJlbGlhYmlsaXR5IEFuYWx5c2lzIChTZWN0aW9uIDMuNS4xLjEpDQoNCiMjIENoZWNraW5nIHRoZSBhY2N1cmFjeSBvZiByYXRpbmdzIG9mIHByb2R1Y3Rpb24gZGF0YSBieSBuYXRpdmUgc3BlYWtlcnMNCg0KYGBge3J9DQojUmVtb3ZlIHBhcnRpY2lwYW50IDQ4IA0KUFJPID0gUFJPWyEoUFJPJHN1YmplY3Q9PSI0OCIpLF0NCg0KUFJPJHNlc3Npb24gPSBmYWN0b3IoUFJPJHNlc3Npb24sIGxldmVscyA9IGMoInByZXRlc3QiLCAicG9zdHRlc3QiLCAibmF0aXZlc3BlYWtlciIsICJwaWN0dXJlbmFtaW5nIiksIGxhYmVscyA9IGMoIlByZS10ZXN0IiwgIlBvc3QtdGVzdCIsIk5hdGl2ZS1TcGVha2VyIiwgIlBpY3R1cmUtTmFtaW5nIikpDQpQQyA9IHN1YnNldChQUk8sIHNlc3Npb24gJWluJSAiTmF0aXZlLVNwZWFrZXIiKQ0KDQptZWFucyA9IG1lYW4oUEMkdG9uZV9zY29yZSkNCm1lYW5zMiA9IG1lYW4oUEMkcGlueWluX3Njb3JlKQ0KbWVhbnMzID0gbWVhbihQQyRyYXRpbmcpDQptZWFuczQgPSBtZWFuKFBDJHRvbmVfc2NvcmVfcmF0ZXIxKQ0KbWVhbnM1ID0gbWVhbihQQyRwaW55aW5fc2NvcmVfcmF0ZXIxKQ0KbWVhbnM2ID0gbWVhbihQQyRyYXRpbmdfcmF0ZXIxKQ0KUmF0ZXIyID0gYyhtZWFucywgbWVhbnMyLCBtZWFuczMpDQpSYXRlcjEgPSBjKG1lYW5zNCwgbWVhbnM1LCBtZWFuczYpDQpUYXNrID0gZmFjdG9yIChjKCJUb25lIGFjY3VyYWN5IiwiUGlueWluIGFjY3VyYWN5IiwiVG9uZSByYXRpbmciKSkNClRhYmxlID0gZGF0YS5mcmFtZSAoIlRhc2siID0gVGFzaywgIlJhdGVyIDEiID0gUmF0ZXIxLCAiUmF0ZXIgMiIgPSBSYXRlcjIpDQpUYWJsZQ0Kcm0obWVhbnMsIG1lYW5zMiwgbWVhbnMzLCBtZWFuczQsIG1lYW5zNSwgbWVhbnM2LCBSYXRlcjIsIFJhdGVyMSwgVGFzaykNCmBgYA0KDQojIyBDaGVja2luZyB0aGUgY29ycmVsYXRpb24gYmV0d2VlbiBSYXRlcjEgYW5kIFJhdGVyMg0KDQpUaGVzZSBjb3JyZWxhdGlvbnMgYXJlIHJlcG9ydGVkIGZvciByYXRpbmdzIG9mIHRoZSBleHBlcmltZW50YWwgZGF0YQ0KDQpgYGB7cn0NCkNXUiA9IHN1YnNldChQUk8sIHNlc3Npb24gJWluJSBjKCJQcmUtdGVzdCIsICJQb3N0LXRlc3QiKSkNCkNQTiA9IHN1YnNldChQUk8sIHNlc3Npb24gJWluJSAiUGljdHVyZS1OYW1pbmciKQ0KY29yMSA9IEthcHBhLnRlc3QoQ1dSJHRvbmVfc2NvcmUsIENXUiR0b25lX3Njb3JlX3JhdGVyMSwgY29uZi5sZXZlbCA9IDAuOTUpIA0KY29yMiA9IEthcHBhLnRlc3QoQ1dSJHBpbnlpbl9zY29yZSwgQ1dSJHBpbnlpbl9zY29yZV9yYXRlcjEsIGNvbmYubGV2ZWwgPSAwLjk1KSANCmNvcjMgPSBpY2MoY2JpbmQoQ1dSJHJhdGluZywgQ1dSJHJhdGluZ19yYXRlcjEpLCAidHdvd2F5IiwgImNvbnNpc3RlbmN5IikNCmNvcjQgPSBLYXBwYS50ZXN0KENQTiR0b25lX3Njb3JlLCBDUE4kdG9uZV9zY29yZV9yYXRlcjEsIGNvbmYubGV2ZWwgPSAwLjk1KQ0KY29yNSA9IEthcHBhLnRlc3QoQ1BOJHBpbnlpbl9zY29yZSwgQ1BOJHBpbnlpbl9zY29yZV9yYXRlcjEsIGNvbmYubGV2ZWwgPSAwLjk1KQ0KY29yNiA9IGljYyhjYmluZChDUE4kcmF0aW5nLCBDUE4kcmF0aW5nX3JhdGVyMSksICJ0d293YXkiLCAiY29uc2lzdGVuY3kiKQ0KYGBgDQoNCiMjIyBSYXRlciBhY2N1cmFjeSBvZiB0b25lIGlkZW50aWZpY2F0aW9uIGluIFdvcmQgUmVwZXRpdGlvbg0KDQpgYGB7cn0NCmNvcjENCmBgYA0KIyMjIFJhdGVyIGFjY3VyYWN5IG9mIHBpbnlpbiBpZGVudGlmaWNhdGlvbiBpbiBXb3JkIFJlcGV0aXRpb24NCg0KYGBge3J9DQpjb3IyDQpgYGANCg0KIyMjIFJhdGVyIGFjY3VyYWN5IG9mIHRvbmUgcmF0aW5nIGluIFdvcmQgUmVwZXRpdGlvbg0KDQpgYGB7cn0NCmNvcjMNCmBgYA0KDQojIyMgUmF0ZXIgYWNjdXJhY3kgb2YgdG9uZSBpZGVudGlmaWNhdGlvbiBpbiBQaWN0dXJlIE5hbWluZw0KDQpgYGB7cn0NCmNvcjQNCmBgYA0KDQojIyMgUmF0ZXIgYWNjdXJhY3kgb2YgcGlueWluIGlkZW50aWZpY2F0aW9uIGluIFBpY3R1cmUgTmFtaW5nDQoNCmBgYHtyfQ0KY29yNQ0KYGBgDQoNCiMjIyBSYXRlciBhY2N1cmFjeSBvZiB0b25lIHJhdGluZyBpbiBQaWN0dXJlIE5hbWluZw0KDQpgYGB7cn0NCmNvcjYNCmBgYA0KDQojIFdvcmQgUmVwZXRpdGlvbiAoU2VjdGlvbiAzLjUuMSkNCg0KIyMgV29yZCBSZXBldGl0aW9uIC0gVG9uZSBhY2N1cmFjeSAoU2VjdGlvbiAzLjUuMS4yKQ0KDQojIyMgTGFiZWwgUGFyYW1ldGVycyBhbmQgcmVtb3ZlIHVuaWRlbnRpZmlhYmxlIHRyaWFscw0KDQpgYGB7cn0NCk5XUiA9IENXUltDV1IkdG9uZSAhPSAwLCBdDQpOV1IkY29uZGl0aW9uID0gZmFjdG9yKE5XUiRjb25kaXRpb24sIGxldmVscyA9IGMoIjAiLCAiMSIsIjIiKSwgbGFiZWxzID0gYygiTFYiLCAiSFYiLCAiSFZCIikpDQpOV1Ikd29yZHR5cGUgPSBmYWN0b3IoTldSJHdvcmR0eXBlLCBsZXZlbHMgPSBjKCJ0cmFpbmVkIiwgInVudHJhaW5lZCIpLCBsYWJlbHMgPSBjKCJUcmFpbmVkIEl0ZW0iLCAiVW50cmFpbmVkIEl0ZW0iKSkNCg0KTldSMiA9IG1lcmdlKE5XUiwgSURfcHJlKQ0KYGBgDQoNCiMjIyBGaWd1cmVzIA0KDQpgYGB7cn0NCm1lYW5zID0gd2l0aChOV1IyLCBhZ2dyZWdhdGUodG9uZV9zY29yZSB+IHN1YmplY3QgKyBzZXNzaW9uICsgY29uZGl0aW9uICsgd29yZHR5cGUsIEZVTiA9IG1lYW4pKQ0KbWVhbnNiID0gd2l0aChOV1IyLCBhZ2dyZWdhdGUodG9uZV9zY29yZSB+IHN1YmplY3QgKyBzZXNzaW9uICsgY29uZGl0aW9uICsgd29yZHR5cGUgKyBhcHRpdHVkZTIsIEZVTiA9IG1lYW4pKQ0KbWVhbnMyYiA9IHdpdGgoTldSMiwgYWdncmVnYXRlKHRvbmVfc2NvcmUgfiBzdWJqZWN0ICsgc2Vzc2lvbiArIGNvbmRpdGlvbiArIHdvcmR0eXBlICsgYXB0aXR1ZGUzLCBGVU4gPSBtZWFuKSkNCg0KeGIgPC0gc3VtbWFyeVNFd2l0aGluKG1lYW5zLCBtZWFzdXJldmFyID0gInRvbmVfc2NvcmUiLCBiZXR3ZWVudmFycyA9IGMoImNvbmRpdGlvbiIpLCB3aXRoaW52YXJzID0gYygid29yZHR5cGUiLCAic2Vzc2lvbiIpLCBpZHZhciA9ICJzdWJqZWN0IiwgbmEucm0gPSBGQUxTRSwgY29uZi5pbnRlcnZhbCA9IC45NSkNCmdyYXBoZGF0YWIgPC0gc3VtbWFyeVNFd2l0aGluKG1lYW5zYiwgbWVhc3VyZXZhciA9ICJ0b25lX3Njb3JlIiwgYmV0d2VlbnZhcnMgPSBjKCJjb25kaXRpb24iLCAiYXB0aXR1ZGUyIiksIHdpdGhpbnZhcnMgPSBjKCJzZXNzaW9uIiwgIndvcmR0eXBlIiksIGlkdmFyID0gInN1YmplY3QiLCBuYS5ybSA9IEZBTFNFLCBjb25mLmludGVydmFsID0gLjk1KQ0KZ3JhcGhkYXRhMmIgPC0gc3VtbWFyeVNFd2l0aGluKG1lYW5zMmIsIG1lYXN1cmV2YXIgPSAidG9uZV9zY29yZSIsIGJldHdlZW52YXJzID0gYygiY29uZGl0aW9uIiwgImFwdGl0dWRlMyIpLCB3aXRoaW52YXJzID0gYygic2Vzc2lvbiIsICJ3b3JkdHlwZSIpLCBpZHZhciA9ICJzdWJqZWN0IiwgbmEucm0gPSBGQUxTRSwgY29uZi5pbnRlcnZhbCA9IC45NSkNCmBgYA0KDQojIyMjIE1haW4gR3JhcGggKEZpZ3VyZSA3KQ0KDQpgYGB7cn0NCldScDEgPSBnZ3Bsb3QgKG1lYW5zLCBhZXMoeCA9IGNvbmRpdGlvbiwgeSA9IHRvbmVfc2NvcmUsIGZpbGwgPSBzZXNzaW9uKSkgDQpXUnAxID0gV1JwMSArIGZhY2V0X3dyYXAofndvcmR0eXBlLCBuY29sID0gMikNCldScDEgPSBXUnAxICsgZ2VvbV92aW9saW4oY29sb3VyID0gImJsYWNrIiwgc2NhbGUgPSAiY291bnQiKQ0KV1JwMSA9IFdScDEgKyBzY2FsZV9maWxsX21hbnVhbChuYW1lID0gIlRlc3QgU2Vzc2lvbiIsIHZhbHVlcyA9IGMoIlByZS10ZXN0IiA9ICJncmV5IiwgIlBvc3QtdGVzdCIgPSAid2hpdGUiKSwgbGFiZWxzID0gYygiUHJlLXRlc3QiLCAiUG9zdC10ZXN0IikpDQpXUnAxID0gV1JwMSArIHNjYWxlX3hfZGlzY3JldGUobGFiZWxzID0gYygiTFYiLCAiSFYiLCAiSFZCIikpDQpXUnAxID0gV1JwMSArIGdlb21fZXJyb3JiYXIoYWVzKHltaW4gPSB0b25lX3Njb3JlLWNpLCB5bWF4ID0gdG9uZV9zY29yZStjaSksIHdpZHRoID0gLjQsIHBvc2l0aW9uID0gcG9zaXRpb25fZG9kZ2UoLjkpLCBkYXRhID0geGIpDQpXUnAxID0gV1JwMSArIHN0YXRfc3VtbWFyeShmdW4ueSA9IG1lYW4sIGdlb20gPSAicG9pbnQiLCBzaGFwZSA9IDIzLCBzaXplID0gMixwb3NpdGlvbiA9IHBvc2l0aW9uX2RvZGdlKC45KSkNCldScDEgPSBXUnAxICt0aGVtZV9idygpKyBnZ3RpdGxlKCdXb3JkIFJlcGV0aXRpb246IFRvbmUgYWNjdXJhY3knKQ0KV1JwMSA9IFdScDEgKyBsYWJzKHggPSAiVmFyaWFiaWxpdHkgQ29uZGl0aW9uIiwgeSA9ICJNZWFuIEFjY3VyYWN5IikNCg0KcHJpbnQoV1JwMSkNCmBgYA0KDQojIyMjIEdyYXBoIChGaWd1cmUgMTIsIExlZnQgcGFuZWwpDQoNCmBgYHtyfQ0KV1JwMiA9IGdncGxvdCAobWVhbnNiLCBhZXMoeCA9IGNvbmRpdGlvbiwgeSA9IHRvbmVfc2NvcmUsIGZpbGwgPSBzZXNzaW9uKSkgDQpXUnAyID0gV1JwMiArIGZhY2V0X3dyYXAofndvcmR0eXBlICsgYXB0aXR1ZGUyLCBuY29sID0gMikNCldScDIgPSBXUnAyICsgZ2VvbV92aW9saW4oY29sb3VyID0gImJsYWNrIiwgc2NhbGUgPSAiY291bnQiKQ0KV1JwMiA9IFdScDIgKyBzY2FsZV9maWxsX21hbnVhbChuYW1lID0gIlRlc3QgU2Vzc2lvbiIsIHZhbHVlcyA9IGMoIlByZS10ZXN0IiA9ICJncmV5IiwgIlBvc3QtdGVzdCIgPSAid2hpdGUiKSwgbGFiZWxzID0gYygiUHJlLXRlc3QiLCAiUG9zdC10ZXN0IikpDQpXUnAyID0gV1JwMiArIHNjYWxlX3hfZGlzY3JldGUobGFiZWxzID0gYygiTFYiLCAiSFYiLCAiSFZCIikpDQpXUnAyID0gV1JwMiArIGdlb21fZXJyb3JiYXIoYWVzKHltaW4gPSB0b25lX3Njb3JlLWNpLCB5bWF4ID0gdG9uZV9zY29yZStjaSksIHdpZHRoID0gLjQsIHBvc2l0aW9uID0gcG9zaXRpb25fZG9kZ2UoLjkpLCBkYXRhID0gZ3JhcGhkYXRhYikNCldScDIgPSBXUnAyICsgc3RhdF9zdW1tYXJ5KGZ1bi55ID0gbWVhbiwgZ2VvbSA9ICJwb2ludCIsIHNoYXBlID0gMjMsIHNpemUgPSAyLHBvc2l0aW9uID0gcG9zaXRpb25fZG9kZ2UoLjkpKQ0KV1JwMiA9IFdScDIgKyB0aGVtZV9idygpKyBnZ3RpdGxlKCdXb3JkIFJlcGV0aXRpb246IFRvbmUgYWNjdXJhY3knKQ0KV1JwMiA9IFdScDIgKyBsYWJzKHggPSAiVmFyaWFiaWxpdHkgQ29uZGl0aW9uIiwgeSA9ICJNZWFuIEFjY3VyYWN5IikNCldScDIubSA9IFdScDIgKyB0aGVtZShsZWdlbmQucG9zaXRpb24gPSAiTlVMTCIpDQoNCnByaW50KFdScDIpDQpgYGANCg0KIyMjIyBDU1RDIEdyYXBoIChOb3QgdXNlZCkNCg0KYGBge3J9DQpXUnAzID0gZ2dwbG90IChtZWFuczJiLCBhZXMoeCA9IGNvbmRpdGlvbiwgeSA9IHRvbmVfc2NvcmUsIGZpbGwgPSBzZXNzaW9uKSkgDQpXUnAzID0gV1JwMyArIGZhY2V0X3dyYXAofndvcmR0eXBlICsgYXB0aXR1ZGUzLCBuY29sID0gMikNCldScDMgPSBXUnAzICsgZ2VvbV92aW9saW4oY29sb3VyID0gImJsYWNrIiwgc2NhbGUgPSAiY291bnQiKQ0KV1JwMyA9IFdScDMgKyBzY2FsZV9maWxsX21hbnVhbChuYW1lID0gIlRlc3QgU2Vzc2lvbiIsIHZhbHVlcyA9IGMoIlByZS10ZXN0IiA9ICJncmV5IiwgIlBvc3QtdGVzdCIgPSAid2hpdGUiKSwgbGFiZWxzID0gYygiUHJlLXRlc3QiLCAiUG9zdC10ZXN0IikpDQpXUnAzID0gV1JwMyArIHNjYWxlX3hfZGlzY3JldGUobGFiZWxzID0gYygiTFYiLCAiSFYiLCAiSFZCIikpDQpXUnAzID0gV1JwMyArIGdlb21fZXJyb3JiYXIoYWVzKHltaW4gPSB0b25lX3Njb3JlLWNpLCB5bWF4ID0gdG9uZV9zY29yZStjaSksIHdpZHRoID0gLjQsIHBvc2l0aW9uID0gcG9zaXRpb25fZG9kZ2UoLjkpLCBkYXRhID0gZ3JhcGhkYXRhMmIpDQpXUnAzID0gV1JwMyArIHN0YXRfc3VtbWFyeShmdW4ueSA9IG1lYW4sIGdlb20gPSAicG9pbnQiLCBzaGFwZSA9IDIzLCBzaXplID0gMixwb3NpdGlvbiA9IHBvc2l0aW9uX2RvZGdlKC45KSkNCldScDMgPSBXUnAzICsgdGhlbWVfYncoKSsgZ2d0aXRsZSgnV29yZCBSZXBldGl0aW9uOiBUb25lIGFjY3VyYWN5JykNCldScDMgPSBXUnAzICsgbGFicyh4ID0gIlZhcmlhYmlsaXR5IENvbmRpdGlvbiIsIHkgPSAiTWVhbiBBY2N1cmFjeSIpDQoNCnByaW50KFdScDMpDQpgYGANCg0KIyMjIFN0YXRpc3RpY2FsIEFuYWx5c2lzDQoNCiMjIyMgTWFpbiBNb2RlbCAod2l0aG91dCBhcHRpdHVkZSBwcmVkaWN0b3JzOyBTZWN0aW9uIDMuNS4xLjIpDQoNCmBgYHtyfQ0KTldSMiA9IGxpekNlbnRlcihOV1IyLCBsaXN0KCJ3b3JkdHlwZSIsICJjb25kaXRpb24iKSkNCk5XUjIgPSBsaXpDb250cmFzdHMoTldSMiwgTldSMiRjb25kaXRpb24sICJMViIpDQoNCm53X2dsbWVyID0gZ2xtZXIodG9uZV9zY29yZSB+IHdvcmR0eXBlLmN0ICogKExWX1ZFUlNVU19IViArIExWX1ZFUlNVU19IVkIpICogc2Vzc2lvbg0KICAgICAgICAgICAgICArICh3b3JkdHlwZS5jdCAqIHNlc3Npb258fHN1YmplY3QpLA0KICAgICAgICAgICAgICBmYW1pbHkgPSAiYmlub21pYWwiLCANCiAgICAgICAgICAgICAgY29udHJvbCA9IGdsbWVyQ29udHJvbChvcHRpbWl6ZXIgPSAiYm9ieXFhIiksIA0KICAgICAgICAgICAgICBkYXRhID0gTldSMikNCmBgYA0KDQojIyMjIyBQcmludCBvdXQgZWZmZWN0cyBpbiBwcmUtdGVzdA0KDQpgYGB7cn0NCmthYmxlKGdldF9jb2VmZnMobndfZ2xtZXIsIGMoIndvcmR0eXBlLmN0IiwgIkxWX1ZFUlNVU19IViIsICJMVl9WRVJTVVNfSFZCIiApKSwgZGlnaXRzID0gMykNCmBgYA0KDQojIyMjIyBQcmludCBvdXQga2V5IGVmZmVjdHMgYW5kIHJlbGV2YW50IGRlc2NyaXB0aXZlIHN0YXRpc3RpY3Mgb2YgdGVzdC1zZXNzaW9uIGFuZCBpbnRlcmFjdGlvbnMgd2l0aCB0ZXN0LXNlc3Npb24gKGkuZS4gbG9va2luZyBlZmZlY3RzIG9mIG9mIHRyYWluaW5nKQ0KDQpgYGB7cn0NCmthYmxlKGdldF9jb2VmZnMobndfZ2xtZXIsIGMoInNlc3Npb25Qb3N0LXRlc3QiLA0KICAgICAgICAgICAgICAgICAgICAgICAgICAgICJ3b3JkdHlwZS5jdDpzZXNzaW9uUG9zdC10ZXN0IiwNCiAgICAgICAgICAgICAgICAgICAgICAgICAgICAiTFZfVkVSU1VTX0hWOnNlc3Npb25Qb3N0LXRlc3QiLA0KICAgICAgICAgICAgICAgICAgICAgICAgICAgICJMVl9WRVJTVVNfSFZCOnNlc3Npb25Qb3N0LXRlc3QiLA0KICAgICAgICAgICAgICAgICAgICAgICAgICAgICJ3b3JkdHlwZS5jdDpMVl9WRVJTVVNfSFY6c2Vzc2lvblBvc3QtdGVzdCIsDQogICAgICAgICAgICAgICAgICAgICAgICAgICAgIndvcmR0eXBlLmN0OkxWX1ZFUlNVU19IVkI6c2Vzc2lvblBvc3QtdGVzdCIpKSwgZGlnaXRzID0gMykNCmBgYA0KDQpgYGB7cn0NCmRhdGFXUnRvbmU8LSBzdW1tYXJ5U0V3aXRoaW4obWVhbnMsIG1lYXN1cmV2YXIgPSAidG9uZV9zY29yZSIsIHdpdGhpbnZhcnMgPSBjKCJzZXNzaW9uIiksIGlkdmFyID0gInN1YmplY3QiLCBuYS5ybSA9IEZBTFNFLCBjb25mLmludGVydmFsID0gLjk1KQ0Ka2FibGUgKGRhdGFXUnRvbmUsIGRpZ2l0cyA9IDMpDQpgYGANCg0KIyMjIyBBbmFseXNpcyB3aXRoIFBDUFQgYWNjdXJhY3kgKFNlY3Rpb24gMy43KQ0KDQpgYGB7cn0NCk5XUjIgPSBsaXpDZW50ZXIoTldSMiwgbGlzdCgic2Vzc2lvbiIsICJhcHRpdHVkZV9wY3B0IiwgInNsb3BlIiwgIndvcmR0eXBlIikpDQoNCm53X2dsbWVyX3BjcHQgPSBnbG1lcih0b25lX3Njb3JlIH4gd29yZHR5cGUuY3QgKiAoTFZfVkVSU1VTX0hWICsgTFZfVkVSU1VTX0hWQikgKiBzZXNzaW9uLmN0IA0KICAgICAgICAgICAgICArIGFwdGl0dWRlX3BjcHQuY3QNCiAgICAgICAgICAgICAgKyBhcHRpdHVkZV9wY3B0LmN0IDogc2Vzc2lvbi5jdA0KICAgICAgICAgICAgICArIGFwdGl0dWRlX3BjcHQuY3QgOiAoTFZfVkVSU1VTX0hWICsgTFZfVkVSU1VTX0hWQikgOiBzZXNzaW9uLmN0IA0KICAgICAgICAgICAgICArIGFwdGl0dWRlX3BjcHQuY3QgOiAoTFZfVkVSU1VTX0hWICsgTFZfVkVSU1VTX0hWQikgOiBzZXNzaW9uLmN0IDogd29yZHR5cGUuY3QNCiAgICAgICAgICAgICAgKyAod29yZHR5cGUuY3QgKiBzZXNzaW9uLmN0fHxzdWJqZWN0KQ0KICAgICAgICAgICAgICAsIGZhbWlseT0iYmlub21pYWwiICwgDQogICAgICAgICAgICAgIGNvbnRyb2wgPSBnbG1lckNvbnRyb2wob3B0aW1pemVyID0gImJvYnlxYSIpLCANCiAgICAgICAgICAgICAgZGF0YSA9IE5XUjIpDQoNCmBgYA0KDQojIyMjIyBDaGVjayB0aGF0IHdlIGdldCB0aGUgc2FtZSBwYXR0ZXJuIG9mIHJlc3VsdHMgZm9yIHRoZSBleHBlcmltZW50YWxseSBtYW5pcHVsYXRlZCB2YXJpYWJsZXMgYXMgaW4gdGhlIHByZXZpb3VzIG1vZGVsIA0KDQpgYGB7cn0NCmthYmxlKGdldF9jb2VmZnMobndfZ2xtZXJfcGNwdCwgYygic2Vzc2lvbi5jdCIsDQogICAgICAgICAgICAgICAgICAgICAgICAgICAgIndvcmR0eXBlLmN0OnNlc3Npb24uY3QiLA0KICAgICAgICAgICAgICAgICAgICAgICAgICAgICJMVl9WRVJTVVNfSFY6c2Vzc2lvbi5jdCIsDQogICAgICAgICAgICAgICAgICAgICAgICAgICAgIkxWX1ZFUlNVU19IVkI6c2Vzc2lvbi5jdCIsDQogICAgICAgICAgICAgICAgICAgICAgICAgICAgIndvcmR0eXBlLmN0OkxWX1ZFUlNVU19IVjpzZXNzaW9uLmN0IiwNCiAgICAgICAgICAgICAgICAgICAgICAgICAgICAid29yZHR5cGUuY3Q6TFZfVkVSU1VTX0hWQjpzZXNzaW9uLmN0IiApKSwgZGlnaXRzID0gMykNCmBgYA0KDQojIyMjIyBQcmludCBvdXQgdGhlIGVmZmVjdCBvZiBpbmRpdmlkdWFsIGFwdGl0dWRlIGFuZCBrZXkgaW50ZXJhY3Rpb25zIHdpdGggYXB0aXR1ZGUNCg0KYGBge3J9DQprYWJsZShnZXRfY29lZmZzKG53X2dsbWVyX3BjcHQsIGMoImFwdGl0dWRlX3BjcHQuY3QiLA0KICAgICAgICAgICAgICAgICAgICAgICAgICAgICAic2Vzc2lvbi5jdDphcHRpdHVkZV9wY3B0LmN0IiwNCiAgICAgICAgICAgICAgICAgICAgICAgICAgICAgIkxWX1ZFUlNVU19IVjpzZXNzaW9uLmN0OmFwdGl0dWRlX3BjcHQuY3QiLA0KICAgICAgICAgICAgICAgICAgICAgICAgICAgICAiTFZfVkVSU1VTX0hWQjpzZXNzaW9uLmN0OmFwdGl0dWRlX3BjcHQuY3QiLA0KICAgICAgICAgICAgICAgICAgICAgICAgICAgICAid29yZHR5cGUuY3Q6TFZfVkVSU1VTX0hWOnNlc3Npb24uY3Q6YXB0aXR1ZGVfcGNwdC5jdCIsDQogICAgICAgICAgICAgICAgICAgICAgICAgICAgICJ3b3JkdHlwZS5jdDpMVl9WRVJTVVNfSFZCOnNlc3Npb24uY3Q6YXB0aXR1ZGVfcGNwdC5jdCIpKSwgZGlnaXRzID0gMykNCmBgYA0KDQojIyMjIEFuYWx5c2lzIHdpdGggQ1NUQyBzbG9wZSAoU2VjdGlvbiAzLjcpDQoNCmBgYHtyfQ0KI01vZGVsIGRpZCBub3QgY29udmVyZ2Ugc28gcmVtb3ZlIHNsb3BlLmN0IDogKExWX1ZFUlNVU19IViArIExWX1ZFUlNVU19IVkIpIDogc2Vzc2lvbi5jdCA6IHdvcmR0eXBlLmN0DQpud19nbG1lcl9jc3RjID0gZ2xtZXIodG9uZV9zY29yZSB+IHdvcmR0eXBlLmN0ICogKExWX1ZFUlNVU19IViArIExWX1ZFUlNVU19IVkIpICogc2Vzc2lvbi5jdCANCiAgICAgICAgICAgICAgKyBzbG9wZS5jdA0KICAgICAgICAgICAgICArIHNsb3BlLmN0IDogc2Vzc2lvbi5jdA0KICAgICAgICAgICAgICArIHNsb3BlLmN0IDogKExWX1ZFUlNVU19IViArIExWX1ZFUlNVU19IVkIpIDogc2Vzc2lvbi5jdCANCiAgICAgICAgICAgICArICh3b3JkdHlwZS5jdCAqIHNlc3Npb24uY3R8c3ViamVjdCkNCiAgICAgICAgICAgICAgLCBmYW1pbHkgPSAiYmlub21pYWwiICwgDQogICAgICAgICAgICAgIGNvbnRyb2wgPSBnbG1lckNvbnRyb2wob3B0aW1pemVyID0gImJvYnlxYSIsIG9wdEN0cmw9bGlzdChtYXhmdW49MWU2KSksIA0KICAgICAgICAgICAgICBkYXRhID0gTldSMikNCmBgYA0KDQojIyMjIyBDaGVjayB0aGF0IGdldCB0aGUgc2FtZSBwYXR0ZXJuIG9mIHJlc3VsdHMgZm9yIGV4cGVyaW1lbnRhbGx5IG1hbmlwdWxhdGVkIHZhcmlhYmxlcyBhcyBpbiBwcmV2aW91cyBtb2RlbCANCg0KYGBge3J9DQprYWJsZShnZXRfY29lZmZzKG53X2dsbWVyX2NzdGMsIGMoInNlc3Npb24uY3QiLCAid29yZHR5cGUuY3Q6c2Vzc2lvbi5jdCIsICJ3b3JkdHlwZS5jdDpMVl9WRVJTVVNfSFY6c2Vzc2lvbi5jdCIsICJ3b3JkdHlwZS5jdDpMVl9WRVJTVVNfSFZCOnNlc3Npb24uY3QiKSksIGRpZ2l0cyA9IDMpDQpgYGANCg0KIyMjIyMgUHJpbnQgb3V0IHRoZSBlZmZlY3Qgb2YgaW5kaXZpZHVhbCBhcHRpdHVkZSBhbmQga2V5IGludGVyYWN0aW9ucyB3aXRoIGFwdGl0dWRlDQoNCmBgYHtyfQ0Ka2FibGUoZ2V0X2NvZWZmcyhud19nbG1lcl9jc3RjLCBjKCJzbG9wZS5jdCIsICJzZXNzaW9uLmN0OnNsb3BlLmN0IiwgIkxWX1ZFUlNVU19IVjpzZXNzaW9uLmN0OnNsb3BlLmN0IiwiTFZfVkVSU1VTX0hWQjpzZXNzaW9uLmN0OnNsb3BlLmN0IikpLCBkaWdpdHMgPSAzKQ0KYGBgDQoNCiMjIFdvcmQgUmVwZXRpdGlvbiAtIFBpbnlpbiBBY2N1cmFjeSAoU2VjdGlvbiAzLjUuMS4zKQ0KDQojIyMgRmlndXJlcyANCg0KYGBge3J9DQptZWFucyA9IHdpdGgoTldSMiwgYWdncmVnYXRlKHBpbnlpbl9zY29yZSB+IHN1YmplY3QgKyBzZXNzaW9uICsgY29uZGl0aW9uICsgd29yZHR5cGUsIEZVTiA9IG1lYW4pKQ0KbWVhbnNiID0gd2l0aChOV1IyLCBhZ2dyZWdhdGUocGlueWluX3Njb3JlIH4gc3ViamVjdCArIHNlc3Npb24gKyBjb25kaXRpb24gKyB3b3JkdHlwZSArIGFwdGl0dWRlMiwgRlVOID0gbWVhbikpDQptZWFuczJiID0gd2l0aChOV1IyLCBhZ2dyZWdhdGUocGlueWluX3Njb3JlIH4gc3ViamVjdCArIHNlc3Npb24gKyBjb25kaXRpb24gKyB3b3JkdHlwZSArIGFwdGl0dWRlMywgRlVOID0gbWVhbikpDQoNCnhiIDwtIHN1bW1hcnlTRXdpdGhpbihtZWFucywgbWVhc3VyZXZhciA9ICJwaW55aW5fc2NvcmUiLCBiZXR3ZWVudmFycyA9IGMoImNvbmRpdGlvbiIpLCB3aXRoaW52YXJzID0gYygid29yZHR5cGUiLCAic2Vzc2lvbiIpLCBpZHZhciA9ICJzdWJqZWN0IiwgbmEucm0gPSBGQUxTRSwgY29uZi5pbnRlcnZhbCA9IC45NSkNCmdyYXBoZGF0YWIgPC0gc3VtbWFyeVNFd2l0aGluKG1lYW5zYiwgbWVhc3VyZXZhciA9ICJwaW55aW5fc2NvcmUiLCBiZXR3ZWVudmFycyA9IGMoImNvbmRpdGlvbiIsICJhcHRpdHVkZTIiKSwgd2l0aGludmFycyA9IGMoInNlc3Npb24iLCAid29yZHR5cGUiKSwgaWR2YXIgPSAic3ViamVjdCIsIG5hLnJtID0gRkFMU0UsIGNvbmYuaW50ZXJ2YWwgPSAuOTUpDQpncmFwaGRhdGEyYiA8LSBzdW1tYXJ5U0V3aXRoaW4obWVhbnMyYiwgbWVhc3VyZXZhciA9ICJwaW55aW5fc2NvcmUiLCBiZXR3ZWVudmFycyA9IGMoImNvbmRpdGlvbiIsICJhcHRpdHVkZTMiKSwgd2l0aGludmFycyA9IGMoInNlc3Npb24iLCAid29yZHR5cGUiKSwgaWR2YXIgPSAic3ViamVjdCIsIG5hLnJtID0gRkFMU0UsIGNvbmYuaW50ZXJ2YWwgPSAuOTUpDQpgYGANCg0KIyMjIyBNYWluIEdyYXBoIChGaWd1cmUgOCkNCg0KYGBge3J9DQpXUnA5ID0gZ2dwbG90IChtZWFucywgYWVzKHggPSBjb25kaXRpb24sIHkgPSBwaW55aW5fc2NvcmUsIGZpbGwgPSBzZXNzaW9uKSkgDQpXUnA5ID0gV1JwOSArIGZhY2V0X3dyYXAofndvcmR0eXBlLCBuY29sID0gMikNCldScDkgPSBXUnA5ICsgZ2VvbV92aW9saW4oY29sb3VyID0gImJsYWNrIiwgc2NhbGUgPSAiY291bnQiKQ0KV1JwOSA9IFdScDkgKyBzY2FsZV9maWxsX21hbnVhbChuYW1lID0gIlRlc3QgU2Vzc2lvbiIsIHZhbHVlcyA9IGMoIlByZS10ZXN0IiA9ICJncmV5IiwgIlBvc3QtdGVzdCIgPSAid2hpdGUiKSwgbGFiZWxzID0gYygiUHJlLXRlc3QiLCAiUG9zdC10ZXN0IikpDQpXUnA5ID0gV1JwOSArIHNjYWxlX3hfZGlzY3JldGUobGFiZWxzID0gYygiTFYiLCAiSFYiLCAiSFZCIikpDQpXUnA5ID0gV1JwOSArIGdlb21fZXJyb3JiYXIoYWVzKHltaW4gPSBwaW55aW5fc2NvcmUtY2ksIHltYXggPSBwaW55aW5fc2NvcmUrY2kpLCB3aWR0aCA9IC40LCBwb3NpdGlvbiA9IHBvc2l0aW9uX2RvZGdlKC45KSwgZGF0YSA9IHhiKQ0KV1JwOSA9IFdScDkgKyBzdGF0X3N1bW1hcnkoZnVuLnkgPSBtZWFuLCBnZW9tID0gInBvaW50Iiwgc2hhcGUgPSAyMywgc2l6ZSA9IDIsIHBvc2l0aW9uID0gcG9zaXRpb25fZG9kZ2UoLjkpKQ0KV1JwOSA9IFdScDkgKyB0aGVtZV9idygpICsgZ2d0aXRsZSgnV29yZCBSZXBldGl0aW9uOiBQaW55aW4gYWNjdXJhY3knKQ0KV1JwOSA9IFdScDkgKyBsYWJzKHggPSAiVmFyaWFiaWxpdHkgQ29uZGl0aW9uIiwgeSA9ICJNZWFuIEFjY3VyYWN5IikNCnByaW50KFdScDkpDQpgYGANCg0KIyMjIyBQQ1BUIEdyYXBoIChGaWd1cmUgMTIsIFJpZ2h0IHBhbmVsKQ0KDQpgYGB7cn0NCldScDEwID0gZ2dwbG90IChtZWFuc2IsIGFlcyh4ID0gY29uZGl0aW9uLCB5ID0gcGlueWluX3Njb3JlLCBmaWxsID0gc2Vzc2lvbikpIA0KV1JwMTAgPSBXUnAxMCArIGZhY2V0X3dyYXAofndvcmR0eXBlICsgYXB0aXR1ZGUyLCBuY29sID0gMikNCldScDEwID0gV1JwMTAgKyBnZW9tX3Zpb2xpbihjb2xvdXIgPSAiYmxhY2siLCBzY2FsZSA9ICJjb3VudCIpDQpXUnAxMCA9IFdScDEwICsgc2NhbGVfZmlsbF9tYW51YWwobmFtZSA9ICJUZXN0IFNlc3Npb24iLCB2YWx1ZXMgPSBjKCJQcmUtdGVzdCIgPSAiZ3JleSIsICJQb3N0LXRlc3QiID0gIndoaXRlIiksIGxhYmVscyA9IGMoIlByZS10ZXN0IiwgIlBvc3QtdGVzdCIpKQ0KV1JwMTAgPSBXUnAxMCArIHNjYWxlX3hfZGlzY3JldGUobGFiZWxzID0gYygiTFYiLCAiSFYiLCAiSFZCIikpDQpXUnAxMCA9IFdScDEwICsgZ2VvbV9lcnJvcmJhcihhZXMoeW1pbiA9IHBpbnlpbl9zY29yZS1jaSwgeW1heCA9IHBpbnlpbl9zY29yZStjaSksIHdpZHRoID0gLjQsIHBvc2l0aW9uID0gcG9zaXRpb25fZG9kZ2UoLjkpLCBkYXRhID0gZ3JhcGhkYXRhYikNCldScDEwID0gV1JwMTAgKyBzdGF0X3N1bW1hcnkoZnVuLnkgPSBtZWFuLCBnZW9tID0gInBvaW50Iiwgc2hhcGUgPSAyMywgc2l6ZSA9IDIscG9zaXRpb24gPSBwb3NpdGlvbl9kb2RnZSguOSkpDQpXUnAxMCA9IFdScDEwICsgdGhlbWVfYncoKSsgZ2d0aXRsZSgnV29yZCBSZXBldGl0aW9uOiBQaW55aW4gYWNjdXJhY3knKQ0KV1JwMTAgPSBXUnAxMCArIGxhYnMoeCA9ICJWYXJpYWJpbGl0eSBDb25kaXRpb24iLCB5ID0gIk1lYW4gQWNjdXJhY3kiKQ0KcHJpbnQoV1JwMTApDQpgYGANCg0KIyMjIyBDU1RDIEdyYXBoIChOb3QgdXNlZCkNCg0KYGBge3J9DQpXUnAxMSA9IGdncGxvdCAobWVhbnMyYiwgYWVzKHggPSBjb25kaXRpb24sIHkgPSBwaW55aW5fc2NvcmUsIGZpbGwgPSBzZXNzaW9uKSkgDQpXUnAxMSA9IFdScDExICsgZmFjZXRfd3JhcCh+d29yZHR5cGUgKyBhcHRpdHVkZTMsIG5jb2wgPSAyKQ0KV1JwMTEgPSBXUnAxMSArIGdlb21fdmlvbGluKGNvbG91ciA9ICJibGFjayIsIHNjYWxlID0gImNvdW50IikNCldScDExID0gV1JwMTEgKyBzY2FsZV9maWxsX21hbnVhbChuYW1lID0gIlRlc3QgU2Vzc2lvbiIsIHZhbHVlcyA9IGMoIlByZS10ZXN0IiA9ICJncmV5IiwgIlBvc3QtdGVzdCIgPSAid2hpdGUiKSwgbGFiZWxzID0gYygiUHJlLXRlc3QiLCAiUG9zdC10ZXN0IikpDQpXUnAxMSA9IFdScDExICsgc2NhbGVfeF9kaXNjcmV0ZShsYWJlbHMgPSBjKCJMViIsICJIViIsICJIVkIiKSkNCldScDExID0gV1JwMTEgKyBnZW9tX2Vycm9yYmFyKGFlcyh5bWluID0gcGlueWluX3Njb3JlLWNpLCB5bWF4ID0gcGlueWluX3Njb3JlK2NpKSwgd2lkdGggPSAuNCwgcG9zaXRpb24gPSBwb3NpdGlvbl9kb2RnZSguOSksIGRhdGEgPSBncmFwaGRhdGEyYikNCldScDExID0gV1JwMTEgKyBzdGF0X3N1bW1hcnkoZnVuLnkgPSBtZWFuLCBnZW9tID0gInBvaW50Iiwgc2hhcGUgPSAyMywgc2l6ZSA9IDIscG9zaXRpb24gPSBwb3NpdGlvbl9kb2RnZSguOSkpDQpXUnAxMSA9IFdScDExICsgdGhlbWVfYncoKSsgZ2d0aXRsZSgnV29yZCBSZXBldGl0aW9uOiBQaW55aW4gYWNjdXJhY3knKQ0KV1JwMTEgPSBXUnAxMSArIGxhYnMoeCA9ICJWYXJpYWJpbGl0eSBDb25kaXRpb24iLCB5ID0gIk1lYW4gQWNjdXJhY3kiKQ0KcHJpbnQoV1JwMTEpDQpgYGANCg0KIyMjIFN0YXRpc3RpY2FsIEFuYWx5c2lzDQoNCiMjIyMgTWFpbiBNb2RlbCAod2l0aG91dCBhcHRpdHVkZSBwcmVkaWN0b3JzOyBTZWN0aW9uIDMuNS4xLjMpDQoNCmBgYHtyfQ0KTldSMiA9IGxpekNlbnRlcihOV1IyLCBsaXN0KCJ3b3JkdHlwZSIpKQ0KTldSMiA9IGxpekNvbnRyYXN0cyhOV1IyLCBOV1IyJGNvbmRpdGlvbiwgIkxWIikNCg0KbndfZ2xtZXIgPSBnbG1lcihwaW55aW5fc2NvcmUgfiB3b3JkdHlwZS5jdCAqIChMVl9WRVJTVVNfSFYgKyBMVl9WRVJTVVNfSFZCKSAqIHNlc3Npb24NCiAgICAgICAgICAgICAgKyAod29yZHR5cGUuY3QgKiBzZXNzaW9ufHxzdWJqZWN0KQ0KICAgICAgICAgICAgICAsIGZhbWlseT0iYmlub21pYWwiLCANCiAgICAgICAgICAgICAgY29udHJvbCA9IGdsbWVyQ29udHJvbChvcHRpbWl6ZXIgPSAiYm9ieXFhIiksIA0KICAgICAgICAgICAgICBkYXRhID0gTldSMikNCmBgYA0KDQojIyMjIyBQcmludCBvdXQgZWZmZWN0cyBhdCBwcmUtdGVzdA0KDQpgYGB7cn0NCiMjIHBvc3NpYmx5IHJlbGV2YW50IGF0IHByZSB0ZXN0IA0Ka2FibGUoZ2V0X2NvZWZmcyhud19nbG1lciwgYygid29yZHR5cGUuY3QiLCAiTFZfVkVSU1VTX0hWIiwgIkxWX1ZFUlNVU19IVkIiKSksIGRpZ2l0cyA9IDMpDQpgYGANCg0KIyMjIyMgUHJpbnQgb3V0IGtleSBlZmZlY3RzIG9mIHJlbGV2YW50IGRlc2NyaXB0aXZlIHN0YXRpc3RpY3MgIHRlc3Qtc2Vzc2lvbiBhbmQgaW50ZXJhY3Rpb25zIHdpdGggdGVzdC1zZXNzaW9uIChpLmUuIGxvb2tpbmcgZWZmZWN0cyBvZiAgdHJhaW5pbmcpIg0KDQpgYGB7cn0NCmthYmxlKGdldF9jb2VmZnMobndfZ2xtZXIsIGMoInNlc3Npb25Qb3N0LXRlc3QiLCAid29yZHR5cGUuY3Q6c2Vzc2lvblBvc3QtdGVzdCIsICJMVl9WRVJTVVNfSFY6c2Vzc2lvblBvc3QtdGVzdCIsICJMVl9WRVJTVVNfSFZCOnNlc3Npb25Qb3N0LXRlc3QiLCAid29yZHR5cGUuY3Q6TFZfVkVSU1VTX0hWOnNlc3Npb25Qb3N0LXRlc3QiLCAid29yZHR5cGUuY3Q6TFZfVkVSU1VTX0hWQjpzZXNzaW9uUG9zdC10ZXN0IikpLCBkaWdpdHMgPSAzKQ0KYGBgDQoNCmBgYHtyfQ0KZGF0YVdScGlueWluIDwtIHN1bW1hcnlTRXdpdGhpbihtZWFucywgbWVhc3VyZXZhciA9ICJwaW55aW5fc2NvcmUiLCB3aXRoaW52YXJzID0gYygic2Vzc2lvbiIpLCBpZHZhciA9ICJzdWJqZWN0IiwgbmEucm0gPSBGQUxTRSwgY29uZi5pbnRlcnZhbCA9IC45NSkNCmthYmxlIChkYXRhV1JwaW55aW4sIGRpZ2l0cyA9IDMpDQpgYGANCg0KIyMjIyBBbmFseXNpcyB3aXRoIFBDUFQgYWNjdXJhY3kgKFNlY3Rpb24gMy43KQ0KDQpgYGB7cn0NCk5XUjIgPSBsaXpDZW50ZXIoTldSMiwgbGlzdCgic2Vzc2lvbiIsICJhY2N1cmFjeSIsICJzbG9wZSIpKQ0KDQpud19nbG1lcl9wY3B0ID0gZ2xtZXIocGlueWluX3Njb3JlIH4gd29yZHR5cGUuY3QgKiAoTFZfVkVSU1VTX0hWICsgTFZfVkVSU1VTX0hWQikgKiBzZXNzaW9uLmN0IA0KICAgICAgICAgICAgICArIGFwdGl0dWRlX3BjcHQuY3QNCiAgICAgICAgICAgICAgKyBhcHRpdHVkZV9wY3B0LmN0IDogc2Vzc2lvbi5jdA0KICAgICAgICAgICAgICArIGFwdGl0dWRlX3BjcHQuY3QgOiAoTFZfVkVSU1VTX0hWICsgTFZfVkVSU1VTX0hWQikgOiBzZXNzaW9uLmN0IA0KICAgICAgICAgICAgICArIGFwdGl0dWRlX3BjcHQuY3QgOiAoTFZfVkVSU1VTX0hWICsgTFZfVkVSU1VTX0hWQikgOiBzZXNzaW9uLmN0IDogd29yZHR5cGUuY3QNCiAgICAgICAgICAgICAgKyAod29yZHR5cGUuY3QgKiBzZXNzaW9uLmN0fHxzdWJqZWN0KQ0KICAgICAgICAgICAgICAsIGZhbWlseSA9ICJiaW5vbWlhbCIsIA0KICAgICAgICAgICAgICBjb250cm9sID0gZ2xtZXJDb250cm9sKG9wdGltaXplciA9ICJib2J5cWEiKSwgDQogICAgICAgICAgICAgIGRhdGEgPSBOV1IyKQ0KYGBgDQoNCiMjIyMjIENoZWNrIHRoYXQgZ2V0IHRoZSBzYW1lIHBhdHRlcm4gb2YgcmVzdWx0cyBmb3IgZXhwZXJpbWVudGFsbHkgbWFuaXB1bGF0ZWQgdmFyaWFibGVzIGFzIGluIHByZXZpb3VzIG1vZGVsDQoNCmBgYHtyfQ0Ka2FibGUoZ2V0X2NvZWZmcyhud19nbG1lcl9wY3B0LCBjKCJzZXNzaW9uLmN0IiwNCiAgICAgICAgICAgICAgICAgICAgICAgICAgICAid29yZHR5cGUuY3Q6c2Vzc2lvbi5jdCIsDQogICAgICAgICAgICAgICAgICAgICAgICAgICAgIkxWX1ZFUlNVU19IVjpzZXNzaW9uLmN0IiwNCiAgICAgICAgICAgICAgICAgICAgICAgICAgICAiTFZfVkVSU1VTX0hWQjpzZXNzaW9uLmN0IiwNCiAgICAgICAgICAgICAgICAgICAgICAgICAgICAid29yZHR5cGUuY3Q6TFZfVkVSU1VTX0hWOnNlc3Npb24uY3QiLA0KICAgICAgICAgICAgICAgICAgICAgICAgICAgICJ3b3JkdHlwZS5jdDpMVl9WRVJTVVNfSFZCOnNlc3Npb24uY3QiICkpLCBkaWdpdHMgPSAzKQ0KYGBgDQoNCiMjIyMjIFByaW50IG91dCB0aGUgZWZmZWN0IG9mIGluZGl2aWR1YWwgYXB0aXR1ZGUgYW5kIGtleSBpbnRlcmFjdGlvbnMgd2l0aCBhcHRpdHVkZQ0KDQpgYGB7cn0NCmthYmxlKGdldF9jb2VmZnMobndfZ2xtZXJfcGNwdCwgYygiYXB0aXR1ZGVfcGNwdC5jdCIsICJzZXNzaW9uLmN0OmFwdGl0dWRlX3BjcHQuY3QiLCAiTFZfVkVSU1VTX0hWOnNlc3Npb24uY3Q6YXB0aXR1ZGVfcGNwdC5jdCIsICJMVl9WRVJTVVNfSFZCOnNlc3Npb24uY3Q6YXB0aXR1ZGVfcGNwdC5jdCIsICJ3b3JkdHlwZS5jdDpMVl9WRVJTVVNfSFY6c2Vzc2lvbi5jdDphcHRpdHVkZV9wY3B0LmN0IiwgIndvcmR0eXBlLmN0OkxWX1ZFUlNVU19IVkI6c2Vzc2lvbi5jdDphcHRpdHVkZV9wY3B0LmN0IikpLCBkaWdpdHMgPSAzKQ0KYGBgDQoNCiMjIyMgQW5hbHlzaXMgd2l0aCBDU1RDIHNsb3BlIA0KDQpgYGB7cn0NCm53X2dsbWVyX2NzdGMgPSBnbG1lcihwaW55aW5fc2NvcmUgfiB3b3JkdHlwZS5jdCAqIChMVl9WRVJTVVNfSFYgKyBMVl9WRVJTVVNfSFZCKSAqIHNlc3Npb24uY3QgDQogICAgICAgICAgICAgICsgc2xvcGUuY3QNCiAgICAgICAgICAgICAgKyBzbG9wZS5jdDogYXB0aXR1ZGVfcGNwdC5jdA0KICAgICAgICAgICAgICArIHNsb3BlLmN0IDogKExWX1ZFUlNVU19IViArIExWX1ZFUlNVU19IVkIpIDogc2Vzc2lvbi5jdCANCiAgICAgICAgICAgICAgKyBzbG9wZS5jdCA6IChMVl9WRVJTVVNfSFYgKyBMVl9WRVJTVVNfSFZCKSA6IHNlc3Npb24uY3QgOiB3b3JkdHlwZS5jdA0KICAgICAgICAgICAgICArICh3b3JkdHlwZS5jdCAqIHNlc3Npb24uY3R8c3ViamVjdCkNCiAgICAgICAgICAgICAgLCBmYW1pbHkgPSAiYmlub21pYWwiLCANCiAgICAgICAgICAgICAgY29udHJvbCA9IGdsbWVyQ29udHJvbChvcHRpbWl6ZXIgPSAiYm9ieXFhIiksIA0KICAgICAgICAgICAgICBkYXRhID0gTldSMikNCmBgYA0KDQojIyMjIyBDaGVjayB0aGF0IHdlIGdldCB0aGUgc2FtZSBwYXR0ZXJuIG9mIHJlc3VsdHMgZm9yIHRoZSBleHBlcmltZW50YWxseSBtYW5pcHVsYXRlZCB2YXJpYWJsZXMgYXMgaW4gdGhlIHByZXZpb3VzIG1vZGVsDQoNCmBgYHtyfQ0Ka2FibGUoZ2V0X2NvZWZmcyhud19nbG1lcl9jc3RjLCBjKCJzZXNzaW9uLmN0IiwgIndvcmR0eXBlLmN0OnNlc3Npb24uY3QiLCAid29yZHR5cGUuY3Q6TFZfVkVSU1VTX0hWOnNlc3Npb24uY3QiLCAid29yZHR5cGUuY3Q6TFZfVkVSU1VTX0hWQjpzZXNzaW9uLmN0IikpLCBkaWdpdHMgPSAzKQ0KYGBgDQoNCiMjIyMjIFByaW50IG91dCB0aGUgZWZmZWN0IG9mIGluZGl2aWR1YWwgYXB0aXR1ZGUgYW5kIGtleSBpbnRlcmFjdGlvbnMgd2l0aCBhcHRpdHVkZQ0KDQpgYGB7cn0NCmthYmxlKGdldF9jb2VmZnMobndfZ2xtZXJfY3N0YywgYygic2xvcGUuY3QiLCAic2xvcGUuY3Q6YXB0aXR1ZGVfcGNwdC5jdCIsICJMVl9WRVJTVVNfSFY6c2Vzc2lvbi5jdDpzbG9wZS5jdCIsICJMVl9WRVJTVVNfSFZCOnNlc3Npb24uY3Q6c2xvcGUuY3QiLCAid29yZHR5cGUuY3Q6TFZfVkVSU1VTX0hWOnNlc3Npb24uY3Q6c2xvcGUuY3QiLCAid29yZHR5cGUuY3Q6TFZfVkVSU1VTX0hWQjpzZXNzaW9uLmN0OnNsb3BlLmN0IikpLCBkaWdpdHMgPSAzKQ0KYGBgDQoNCiMgVGhyZWUtaW50ZXJ2YWwgT2RkaXR5IFRhc2sgKFNlY3Rpb24gMy40LjEpDQoNCiMjIExhYmVsIFBhcmFtZXRlcnMNCg0KYGBge3J9DQpkaXMkY29uZGl0aW9uID0gZmFjdG9yKGRpcyRjb25kaXRpb24sIGxldmVscyA9IGMoIjAiLCAiMSIsICIyIiksIGxhYmVscyA9IGMoIkxWIiwgIkhWIiwgIkhWQiIpKQ0KZGlzJHNlc3Npb24gPSBmYWN0b3IoZGlzJHNlc3Npb24sIGxldmVscyA9IGMoIjEiLCAiMiIpLCBsYWJlbHMgPSBjKCJQcmUtdGVzdCIsICJQb3N0LXRlc3QiKSkNCmRpcyR0cmlhbHR5cGUgPSBkaXMkdm9pY2V0eXBlID0gZmFjdG9yKGRpcyR2b2ljZXR5cGUsIGxldmVscyA9IGMoImZmZiIsICJmZm0iLCAiZm1mIiksIGxhYmVscyA9IGMoIk5ldXRyYWwiLCAiRWFzeSIsICJIYXJkIikpDQpkaXMkd29yZHR5cGUgPSBmYWN0b3IoZGlzJHdvcmR0eXBlLCBsZXZlbHMgPSBjKCJvbGR3b3JkIiwgIm5ld3dvcmQiKSwgbGFiZWxzID0gYygiVHJhaW5lZCBJdGVtIiwgIlVudHJhaW5lZCBJdGVtIikpDQpkaXMyID0gbWVyZ2UoSURfcHJlLCBkaXMpDQpgYGANCg0KIyMgRmlndXJlcw0KDQpgYGB7cn0NCm1lYW5zID0gd2l0aChkaXMyLCBhZ2dyZWdhdGUoc2NvcmUgfiBzdWJqZWN0ICsgY29uZGl0aW9uICsgdm9pY2V0eXBlICsgd29yZHR5cGUgKyBzZXNzaW9uLCBGVU4gPSBtZWFuKSkNCm1lYW5zYiA9IHdpdGgoZGlzMiwgYWdncmVnYXRlKHNjb3JlIH4gc3ViamVjdCArIGNvbmRpdGlvbiArIHZvaWNldHlwZSArIHdvcmR0eXBlICsgc2Vzc2lvbiArIGFwdGl0dWRlMithY2N1cmFjeSwgRlVOID0gbWVhbikpDQptZWFuczJiID0gd2l0aChkaXMyLCBhZ2dyZWdhdGUoc2NvcmUgfiBzdWJqZWN0ICsgY29uZGl0aW9uICsgdm9pY2V0eXBlICsgd29yZHR5cGUgKyBzZXNzaW9uICsgYXB0aXR1ZGUzK3Nsb3BlLCBGVU4gPSBtZWFuKSkNCg0KZ3JhcGhkYXRhIDwtIHN1bW1hcnlTRXdpdGhpbihtZWFuc2IsIG1lYXN1cmV2YXIgPSAic2NvcmUiLCBiZXR3ZWVudmFycyA9IGMoImNvbmRpdGlvbiIsICJhcHRpdHVkZTIiKSwgd2l0aGludmFycyA9IGMoIndvcmR0eXBlIiwgInNlc3Npb24iKSwgaWR2YXIgPSAic3ViamVjdCIsIG5hLnJtID0gRkFMU0UsIGNvbmYuaW50ZXJ2YWwgPSAuOTUpDQoNCmdyYXBoZGF0YTIgPC0gc3VtbWFyeVNFd2l0aGluKG1lYW5zMmIsIG1lYXN1cmV2YXIgPSAic2NvcmUiLCBiZXR3ZWVudmFycyA9IGMoImNvbmRpdGlvbiIsICJhcHRpdHVkZTMiKSwgd2l0aGludmFycyA9IGMoIndvcmR0eXBlIiwic2Vzc2lvbiIpLCBpZHZhciA9ICJzdWJqZWN0IiwgbmEucm0gPSBGQUxTRSwgY29uZi5pbnRlcnZhbCA9IC45NSkNCg0KeDIgPC0gc3VtbWFyeVNFd2l0aGluKG1lYW5zLCBtZWFzdXJldmFyID0gInNjb3JlIiwgYmV0d2VlbnZhcnMgPSBjKCJjb25kaXRpb24iKSwgd2l0aGludmFycyA9IGMoIndvcmR0eXBlIiwgInNlc3Npb24iKSwgaWR2YXIgPSAic3ViamVjdCIsIG5hLnJtID0gRkFMU0UsIGNvbmYuaW50ZXJ2YWwgPSAuOTUpDQpgYGANCg0KIyMjIE1haW4gR3JhcGggKEZpZ3VyZSA1KQ0KDQpgYGB7cn0NCkRwMSA9IGdncGxvdCAobWVhbnMsIGFlcyh4ID0gY29uZGl0aW9uLCB5ID0gc2NvcmUsIGZpbGwgPSBzZXNzaW9uKSkgDQpEcDEgPSBEcDEgKyBmYWNldF93cmFwKH4gd29yZHR5cGUsIG5jb2wgPSAyKQ0KRHAxID0gRHAxICsgZ2VvbV92aW9saW4oY29sb3VyID0gImJsYWNrIiwgc2NhbGUgPSAiY291bnQiKQ0KRHAxID0gRHAxICsgc2NhbGVfZmlsbF9tYW51YWwobmFtZSA9ICJUZXN0IFNlc3Npb24iLCB2YWx1ZXMgPSBjKCJQcmUtdGVzdCIgPSAiZ3JleSIsICJQb3N0LXRlc3QiID0gIndoaXRlIiksIGxhYmVscyA9IGMoIlByZS10ZXN0IiwgIlBvc3QtdGVzdCIpKQ0KRHAxID0gRHAxICsgc2NhbGVfeF9kaXNjcmV0ZShsYWJlbHMgPSBjKCJMViIsICJIViIsICJIVkIiKSkNCkRwMSA9IERwMSArIGdlb21fZXJyb3JiYXIoYWVzKHltaW4gPSBzY29yZS1jaSwgeW1heCA9IHNjb3JlK2NpKSwgd2lkdGggPSAuNCwgcG9zaXRpb24gPSBwb3NpdGlvbl9kb2RnZSguOSksIGRhdGEgPSB4MikNCkRwMSA9IERwMSArIHN0YXRfc3VtbWFyeShmdW4ueSA9IG1lYW4sIGdlb20gPSAicG9pbnQiLCBzaGFwZSA9IDIzLCBzaXplID0gMiwgcG9zaXRpb24gPSBwb3NpdGlvbl9kb2RnZSguOSkpDQpEcDEgPSBEcDEgKyB0aGVtZV9idygpICsgZ2d0aXRsZSgnVGhyZWUtaW50ZXJ2YWwgT2RkaXR5JykNCkRwMSA9IERwMSArIGxhYnMoeCA9ICJWYXJpYWJpbGl0eSBDb25kaXRpb24iLCB5ID0gIk1lYW4gQWNjdXJhY3kiKQ0KcHJpbnQoRHAxKQ0KYGBgDQoNCiMjIyBQQ1BUIGdyYXBoIChGaWd1cmUgMTAsIHRvcCBwYW5lbCkNCg0KYGBge3J9DQpEcDIgPSBnZ3Bsb3QgKG1lYW5zYiwgYWVzKHggPSBjb25kaXRpb24sIHkgPSBzY29yZSwgZmlsbCA9IHNlc3Npb24pKQ0KRHAyID0gRHAyICsgZmFjZXRfd3JhcCh+IHdvcmR0eXBlICsgYXB0aXR1ZGUyLCBuY29sID0gMikNCkRwMiA9IERwMiArIGdlb21fdmlvbGluKGNvbG91ciA9ICJibGFjayIsIHNjYWxlID0gImNvdW50IikNCkRwMiA9IERwMiArIHNjYWxlX2ZpbGxfbWFudWFsKG5hbWUgPSAiVGVzdCBTZXNzaW9uIiwgdmFsdWVzID0gYygiUHJlLXRlc3QiID0gImdyZXkiLCAiUG9zdC10ZXN0IiA9ICJ3aGl0ZSIpLCBsYWJlbHMgPSBjKCJQcmUtdGVzdCIsICJQb3N0LXRlc3QiKSkNCkRwMiA9IERwMiArIHNjYWxlX3hfZGlzY3JldGUobGFiZWxzID0gYygiTFYiLCAiSFYiLCAiSFZCIikpDQpEcDIgPSBEcDIgKyBnZW9tX2Vycm9yYmFyKGFlcyh5bWluID0gc2NvcmUtY2ksIHltYXggPSBzY29yZStjaSksIHdpZHRoID0gLjQsIHBvc2l0aW9uID0gcG9zaXRpb25fZG9kZ2UoLjkpLCBkYXRhID0gZ3JhcGhkYXRhKQ0KRHAyID0gRHAyICsgc3RhdF9zdW1tYXJ5KGZ1bi55ID0gbWVhbiwgZ2VvbSA9ICJwb2ludCIsIHNoYXBlID0gMjMsIHNpemUgPSAyLCBwb3NpdGlvbiA9IHBvc2l0aW9uX2RvZGdlKC45KSkNCkRwMiA9IERwMiArIHRoZW1lX2J3KCkrIGdndGl0bGUoJ1RocmVlLWludGVydmFsIE9kZGl0eScpDQpEcDIgPSBEcDIgKyBsYWJzKHggPSAiVmFyaWFiaWxpdHkgQ29uZGl0aW9uIiwgeSA9ICJNZWFuIEFjY3VyYWN5IikNCnByaW50KERwMikNCmBgYA0KDQojIyMgQ1NUQyBncmFwaCAoTm90IGluY2x1ZGVkKQ0KDQpgYGB7cn0NCkRwMyA9IGdncGxvdCAobWVhbnMyYiwgYWVzKHggPSBjb25kaXRpb24sIHkgPSBzY29yZSwgZmlsbCA9IHNlc3Npb24pKSANCkRwMyA9IERwMyArIGZhY2V0X3dyYXAofiB3b3JkdHlwZSArIGFwdGl0dWRlMywgbmNvbCA9IDIpDQpEcDMgPSBEcDMgKyBnZW9tX3Zpb2xpbihjb2xvdXIgPSAiYmxhY2siLCBzY2FsZSA9ICJjb3VudCIpDQpEcDMgPSBEcDMgKyBzY2FsZV9maWxsX21hbnVhbChuYW1lID0gIlRlc3QgU2Vzc2lvbiIsIHZhbHVlcyA9IGMoIlByZS10ZXN0IiA9ICJncmV5IiwgIlBvc3QtdGVzdCIgPSAid2hpdGUiKSwgbGFiZWxzID0gYygiUHJlLXRlc3QiLCAiUG9zdC10ZXN0IikpDQpEcDMgPSBEcDMgKyBzY2FsZV94X2Rpc2NyZXRlKGxhYmVscyA9IGMoIkxWIiwgIkhWIiwgIkhWQiIpKQ0KRHAzID0gRHAzICsgZ2VvbV9lcnJvcmJhcihhZXMoeW1pbiA9IHNjb3JlLWNpLCB5bWF4ID0gc2NvcmUrY2kpLCB3aWR0aCA9IC40LCBwb3NpdGlvbiA9IHBvc2l0aW9uX2RvZGdlKC45KSwgZGF0YSA9IGdyYXBoZGF0YTIpDQpEcDMgPSBEcDMgKyBzdGF0X3N1bW1hcnkoZnVuLnkgPSBtZWFuLCBnZW9tID0gInBvaW50Iiwgc2hhcGUgPSAyMywgc2l6ZSA9IDIsIHBvc2l0aW9uID0gcG9zaXRpb25fZG9kZ2UoLjkpKQ0KRHAzID0gRHAzICsgdGhlbWVfYncoKSArIGdndGl0bGUoJ1RocmVlLWludGVydmFsIE9kZGl0eScpDQpEcDMgPSBEcDMgKyBsYWJzKHggPSAiVmFyaWFiaWxpdHkgQ29uZGl0aW9uIiwgeSA9ICJNZWFuIEFjY3VyYWN5IikNCnByaW50KERwMykNCmBgYA0KDQojIyMgUENQVCBHcmFwaCAoRG90IHBsb3QsIG5vdCBjdXJyZW50bHkgdXNlZCkNCg0KYGBge3J9DQptZWFucy5wcmUgPC0gc3Vic2V0KG1lYW5zYiwgc2Vzc2lvbiA9PSAiUHJlLXRlc3QiKQ0KbWVhbnMucHJlID0gcmVuYW1lKG1lYW5zLnByZSwgYyhzY29yZSA9ICJwcmVzY29yZSIpKQ0KbWVhbnMucHJlJHNlc3Npb24gPSBOVUxMDQptZWFucy5wb3N0IDwtIHN1YnNldChtZWFuc2IsIHNlc3Npb24gPT0gIlBvc3QtdGVzdCIpDQptZWFucy5wb3N0ID0gcmVuYW1lKG1lYW5zLnBvc3QsIGMoc2NvcmUgPSJwb3N0c2NvcmUiKSkNCm1lYW5zLnBvc3Qkc2Vzc2lvbiA9IE5VTEwNCg0KbWVhbnMueCA9IG1lcmdlKG1lYW5zLnByZSwgbWVhbnMucG9zdCkNCg0KbWVhbnMueCRkaWZmID0gbWVhbnMueCRwb3N0c2NvcmUtbWVhbnMueCRwcmVzY29yZQ0KDQpEcDQgPSBnZ3Bsb3QobWVhbnMueCwgYWVzKHggPSBhY2N1cmFjeSwgeSA9IGRpZmYpKQ0KRHA0PURwNCArIGdlb21fcG9pbnQoYWVzKHNoYXBlID0gZmFjdG9yKGNvbmRpdGlvbikpKQ0KRHA0ID0gRHA0ICsgZ2d0aXRsZSgnVGhyZWUtaW50ZXJ2YWwgT2RkaXR5JykNCg0KcHJpbnQoRHA0KQ0KDQpybShtZWFucy5wcmUpDQpybShtZWFucy5wb3N0KQ0Kcm0obWVhbnMueCkNCmBgYA0KDQojIyMgQ1NUQyBHcmFwaCAoRG90IHBsb3QsIG5vdCBjdXJyZW50bHkgdXNlZCkNCg0KYGBge3J9DQptZWFuczIucHJlIDwtIHN1YnNldChtZWFuczJiLCBzZXNzaW9uID09ICJQcmUtdGVzdCIpDQptZWFuczIucHJlID0gcmVuYW1lKG1lYW5zMi5wcmUsIGMoc2NvcmUgPSAicHJlc2NvcmUiKSkNCm1lYW5zMi5wcmUkc2Vzc2lvbiA9IE5VTEwNCm1lYW5zMi5wb3N0IDwtIHN1YnNldChtZWFuczJiLCBzZXNzaW9uID09ICJQb3N0LXRlc3QiKQ0KbWVhbnMyLnBvc3QgPSByZW5hbWUobWVhbnMyLnBvc3QsIGMoc2NvcmUgPSAicG9zdHNjb3JlIikpDQptZWFuczIucG9zdCRzZXNzaW9uID0gTlVMTA0KDQptZWFuczIueCA9IG1lcmdlKG1lYW5zMi5wcmUsIG1lYW5zMi5wb3N0KQ0KDQptZWFuczIueCRkaWZmID0gbWVhbnMyLngkcG9zdHNjb3JlLW1lYW5zMi54JHByZXNjb3JlDQoNCkRwNSA9IGdncGxvdChtZWFuczIueCwgYWVzKHggPSBzbG9wZSwgeSA9IGRpZmYpKQ0KRHA1PURwNSArIGdlb21fcG9pbnQoYWVzKHNoYXBlID0gZmFjdG9yKGNvbmRpdGlvbikpKQ0KRHA1ID0gRHA1ICsgZ2d0aXRsZSgnVGhyZWUtaW50ZXJ2YWwgT2RkaXR5JykNCkRwNQ0KDQpybShtZWFuczIucHJlKQ0Kcm0obWVhbnMyLnBvc3QpDQpybShtZWFuczIueCkNCmBgYA0KDQojIyBTdGF0aXN0aWNhbCBBbmFseXNpcyANCg0KIyMjIE1haW4gbW9kZWwgKHdpdGhvdXQgYXB0aXR1ZGUgcHJlZGljdG9yczsgU2VjdGlvbiAzLjQuMSkNCg0KYGBge3J9DQpkaXMyID0gbGl6Q2VudGVyKGRpczIsIGxpc3QoInNlc3Npb24iLCAid29yZHR5cGUiLCAiYXB0aXR1ZGVfcGNwdCIpKQ0KZGlzMiA9IGxpekNvbnRyYXN0cyhkaXMyLCBkaXMyJGNvbmRpdGlvbiwgIkxWIikNCmRpczIgPSBsaXpDb250cmFzdHMoZGlzMiwgZGlzMiR2b2ljZXR5cGUsICJOZXV0cmFsIikNCg0KZF9nbG1lciA9IGdsbWVyKHNjb3JlIH4gc2Vzc2lvbiAqIHdvcmR0eXBlLmN0ICogKExWX1ZFUlNVU19IViArIExWX1ZFUlNVU19IVkIpIA0KICAgICAgICAgICAgICArIChOZXV0cmFsX1ZFUlNVU19FYXN5ICsgTmV1dHJhbF9WRVJTVVNfSGFyZCk6IHNlc3Npb24NCiAgICAgICAgICAgICAgKyAoTmV1dHJhbF9WRVJTVVNfRWFzeSArIE5ldXRyYWxfVkVSU1VTX0hhcmQpIA0KICAgICAgICAgICAgICArIChzZXNzaW9uICogd29yZHR5cGUuY3R8fHN1YmplY3QpDQogICAgICAgICAgICAgICwgZmFtaWx5ID0gImJpbm9taWFsIiwgDQogICAgICAgICAgICAgIGNvbnRyb2wgPSBnbG1lckNvbnRyb2wob3B0aW1pemVyID0gImJvYnlxYSIpLCANCiAgICAgICAgICAgICAgZGF0YSA9IGRpczIpDQpgYGANCg0KIyMjIyBQcmludCBvdXQga2V5IGVmZmVjdHMgYXQgcHJlLXRlc3Qgc2Vzc2lvbg0KDQpgYGB7cn0NCmthYmxlKGdldF9jb2VmZnMoZF9nbG1lciwgYygiKEludGVyY2VwdCkiLCAid29yZHR5cGUuY3QiLCAiTFZfVkVSU1VTX0hWIiwgIkxWX1ZFUlNVU19IVkIiLCAiTmV1dHJhbF9WRVJTVVNfRWFzeSIsICJOZXV0cmFsX1ZFUlNVU19IYXJkIikpLCBkaWdpdHMgPSAzKQ0KYGBgDQoNCg0KDQojIyMjIFByaW50IG91dCBrZXkgZWZmZWN0cyByZWxldmFudCBkZXNjcmlwdGl2ZSBzdGF0aXN0aWNzIG9mIHRlc3Qtc2Vzc2lvbiBhbmQgaW50ZXJhY3Rpb25zIHdpdGggdGVzdC1zZXNzaW9uIChpLmUuIGxvb2tpbmcgZWZmZWN0cyBvZiAgdHJhaW5pbmcpDQoNCmBgYHtyfQ0Ka2FibGUoZ2V0X2NvZWZmcyhkX2dsbWVyLCBjKCJzZXNzaW9uUG9zdC10ZXN0IiwgInNlc3Npb25Qb3N0LXRlc3Q6d29yZHR5cGUuY3QiLCAic2Vzc2lvblBvc3QtdGVzdDpMVl9WRVJTVVNfSFYiLCAgInNlc3Npb25Qb3N0LXRlc3Q6TFZfVkVSU1VTX0hWQiIsICJzZXNzaW9uUG9zdC10ZXN0OndvcmR0eXBlLmN0OkxWX1ZFUlNVU19IViIsICJzZXNzaW9uUG9zdC10ZXN0OndvcmR0eXBlLmN0OkxWX1ZFUlNVU19IVkIiKSksIGRpZ2l0cyA9IDMpDQpgYGANCg0KYGBge3J9DQpkYXRhRCA8LSBzdW1tYXJ5U0V3aXRoaW4obWVhbnMsIG1lYXN1cmV2YXIgPSAic2NvcmUiLCB3aXRoaW52YXJzID0gIGMoInNlc3Npb24iKSwgaWR2YXIgPSAic3ViamVjdCIsIG5hLnJtID0gRkFMU0UsIGNvbmYuaW50ZXJ2YWwgPSAuOTUpDQprYWJsZSAoZGF0YUQsIGRpZ2l0cyA9IDMpDQpgYGANCg0KIyMjIyBFeGFtaW5lIGlmIGxlYXJuaW5nIGlzIGRpZmZlcmVudCBmb3IgdGhlIGVhc2llci9oYXJkZXIgdHJpYWwgdHlwZXMgYW5kIHByaW50IG91dCByZWxldmFudCBkZXNjcmlwdGl2ZSBzdGF0aXN0aWNzIA0KDQpgYGB7cn0NCnJvdW5kKGdldF9jb2VmZnMoZF9nbG1lciwgYygic2Vzc2lvblBvc3QtdGVzdDpOZXV0cmFsX1ZFUlNVU19FYXN5Iiwic2Vzc2lvblBvc3QtdGVzdDpOZXV0cmFsX1ZFUlNVU19IYXJkIikpLCAzKQ0KYGBgDQpgYGB7cn0NCmRhdGFEMiA8LSBzdW1tYXJ5U0V3aXRoaW4obWVhbnMsIG1lYXN1cmV2YXIgPSAic2NvcmUiLCB3aXRoaW52YXJzID0gIGMoInNlc3Npb24iLCAidm9pY2V0eXBlIiksIGlkdmFyID0gInN1YmplY3QiLCBuYS5ybSA9IEZBTFNFLCBjb25mLmludGVydmFsID0gLjk1KQ0Ka2FibGUgKGRhdGFEMiwgZGlnaXRzID0gMykNCmBgYA0KIyMjIEFuYWx5c2VzIHdpdGggUENQVCBhY2N1cmFjeSAoU2VjdGlvbiAzLjcpDQoNCmBgYHtyfQ0KZGlzMiA9IGxpekNlbnRlcihkaXMyLCBsaXN0KCJzZXNzaW9uLmN0IikpDQoNCmRfZ2xtZXJfcGNwdCA9IGdsbWVyKHNjb3JlIH4gc2Vzc2lvbi5jdCAqIHdvcmR0eXBlLmN0ICogKExWX1ZFUlNVU19IViArIExWX1ZFUlNVU19IVkIpIA0KICAgICAgICAgICAgICArIChOZXV0cmFsX1ZFUlNVU19FYXN5ICsgTmV1dHJhbF9WRVJTVVNfSGFyZCk6IHNlc3Npb24uY3QgDQogICAgICAgICAgICAgICsgKE5ldXRyYWxfVkVSU1VTX0Vhc3kgKyBOZXV0cmFsX1ZFUlNVU19IYXJkKSANCiAgICAgICAgICAgICAgKyBhcHRpdHVkZV9wY3B0LmN0DQogICAgICAgICAgICAgICsgYXB0aXR1ZGVfcGNwdC5jdDogc2Vzc2lvbi5jdA0KICAgICAgICAgICAgICArIGFwdGl0dWRlX3BjcHQuY3QgOiAoTFZfVkVSU1VTX0hWICsgTFZfVkVSU1VTX0hWQikgOiBzZXNzaW9uLmN0DQogICAgICAgICAgICAgICsgYXB0aXR1ZGVfcGNwdC5jdCA6IChMVl9WRVJTVVNfSFYgKyBMVl9WRVJTVVNfSFZCKSA6IHNlc3Npb24uY3QgOiB3b3JkdHlwZS5jdA0KICAgICAgICAgICAgICArIChzZXNzaW9uLmN0ICogd29yZHR5cGUuY3R8fHN1YmplY3QpDQogICAgICAgICAgICAgICwgZmFtaWx5ID0gImJpbm9taWFsIiwgDQogICAgICAgICAgICAgIGNvbnRyb2wgPSBnbG1lckNvbnRyb2wob3B0aW1pemVyID0gImJvYnlxYSIpLCANCiAgICAgICAgICAgICAgZGF0YSA9IGRpczIpDQpgYGAgDQoNCiMjIyMgUHJpbnQgb3V0IGtleSBlZmZlY3RzIG9mIHRlc3Qtc2Vzc2lvbiBhbmQgaW50ZXJhY3Rpb25zIHdpdGggdGVzdC1zZXNzaW9uIChpLmUuIGxvb2tpbmcgZWZmZWN0cyBvZiAgdHJhaW5pbmcpDQoNCmBgYHtyfQ0Ka2FibGUoZ2V0X2NvZWZmcyhkX2dsbWVyX3BjcHQsIGMoIihJbnRlcmNlcHQpIiwgInNlc3Npb24uY3QiLCAic2Vzc2lvbi5jdDp3b3JkdHlwZS5jdCIsICJzZXNzaW9uLmN0OkxWX1ZFUlNVU19IViIsICJzZXNzaW9uLmN0OkxWX1ZFUlNVU19IVkIiLCAic2Vzc2lvbi5jdDp3b3JkdHlwZS5jdDpMVl9WRVJTVVNfSFYiLCAgInNlc3Npb24uY3Q6d29yZHR5cGUuY3Q6TFZfVkVSU1VTX0hWQiIpKSwgZGlnaXRzID0gMykNCmBgYCANCg0KIyMjIyBDaGVjayB0aGF0IHdlIGdldCB0aGUgc2FtZSBwYXR0ZXJuIG9mIHJlc3VsdHMgZm9yIHRoZSBleHBlcmltZW50YWxseSBtYW5pcHVsYXRlZCB2YXJpYWJsZXMgYXMgaW4gdGhlIHByZXZpb3VzIG1vZGVsDQoNCmBgYHtyfQ0KZ2V0X2NvZWZmcyhkX2dsbWVyX3BjcHQsIGMoImFwdGl0dWRlX3BjcHQuY3QiLCAic2Vzc2lvbi5jdDphcHRpdHVkZV9wY3B0LmN0IiwgInNlc3Npb24uY3Q6TFZfVkVSU1VTX0hWOmFwdGl0dWRlX3BjcHQuY3QiLCAic2Vzc2lvbi5jdDpMVl9WRVJTVVNfSFZCOmFwdGl0dWRlX3BjcHQuY3QiLCAic2Vzc2lvbi5jdDp3b3JkdHlwZS5jdDpMVl9WRVJTVVNfSFY6YXB0aXR1ZGVfcGNwdC5jdCIsICJzZXNzaW9uLmN0OndvcmR0eXBlLmN0OkxWX1ZFUlNVU19IVkI6YXB0aXR1ZGVfcGNwdC5jdCIpKQ0KYGBgIA0KDQojIyMgQW5hbHlzZXMtd2l0aCBDU1RDIHNsb3BlDQoNCmBgYHtyfQ0KZGlzMiA9IGxpekNlbnRlcihkaXMyLCBjKCJzbG9wZSIpKQ0KDQpkX2dsbWVyX2NzdGMgPSBnbG1lcihzY29yZSB+IHNlc3Npb24uY3QgKiB3b3JkdHlwZS5jdCAqIChMVl9WRVJTVVNfSFYgKyBMVl9WRVJTVVNfSFZCKSANCiAgICAgICAgICAgICAgKyAoTmV1dHJhbF9WRVJTVVNfRWFzeSArIE5ldXRyYWxfVkVSU1VTX0hhcmQpOiBzZXNzaW9uLmN0IA0KICAgICAgICAgICAgICArIChOZXV0cmFsX1ZFUlNVU19FYXN5ICsgTmV1dHJhbF9WRVJTVVNfSGFyZCkgDQogICAgICAgICAgICAgICsgc2xvcGUuY3QNCiAgICAgICAgICAgICAgKyBzbG9wZS5jdDogc2Vzc2lvbi5jdA0KICAgICAgICAgICAgICArIHNsb3BlLmN0IDogKExWX1ZFUlNVU19IViArIExWX1ZFUlNVU19IVkIpIDogc2Vzc2lvbi5jdA0KICAgICAgICAgICAgICArIHNsb3BlLmN0IDogKExWX1ZFUlNVU19IViArIExWX1ZFUlNVU19IVkIpIDogc2Vzc2lvbi5jdDogd29yZHR5cGUuY3QNCiAgICAgICAgICAgICAgKyAoc2Vzc2lvbi5jdCAqIHdvcmR0eXBlLmN0fHxzdWJqZWN0KQ0KICAgICAgICAgICAgICAsIGZhbWlseSA9ICJiaW5vbWlhbCIsIA0KICAgICAgICAgICAgICBjb250cm9sID0gZ2xtZXJDb250cm9sKG9wdGltaXplciA9ICJib2J5cWEiKSwgDQogICAgICAgICAgICAgIGRhdGEgPSBkaXMyKQ0KYGBgDQoNCiMjIyMgUHJpbnQgb3V0IGtleSBlZmZlY3RzIG9mIHRlc3Qtc2Vzc2lvbiBhbmQgaW50ZXJhY3Rpb25zIHdpdGggdGVzdC1zZXNzaW9uIChpLmUuIGxvb2tpbmcgZWZmZWN0cyBvZiAgdHJhaW5pbmcpDQoNCmBgYHtyfQ0Ka2FibGUoZ2V0X2NvZWZmcyhkX2dsbWVyX2NzdGMsIGMoIihJbnRlcmNlcHQpIiwgInNlc3Npb24uY3QiLCAic2Vzc2lvbi5jdDp3b3JkdHlwZS5jdCIsICJzZXNzaW9uLmN0OkxWX1ZFUlNVU19IViIsICJzZXNzaW9uLmN0OkxWX1ZFUlNVU19IVkIiLCAic2Vzc2lvbi5jdDp3b3JkdHlwZS5jdDpMVl9WRVJTVVNfSFYiLCAic2Vzc2lvbi5jdDp3b3JkdHlwZS5jdDpMVl9WRVJTVVNfSFZCIikpLCBkaWdpdHMgPSAzKQ0KYGBgIA0KDQojIyMjIENoZWNrIHRoYXQgd2UgZ2V0IHRoZSBzYW1lIHBhdHRlcm4gb2YgcmVzdWx0cyBmb3IgdGhlIGV4cGVyaW1lbnRhbGx5IG1hbmlwdWxhdGVkIHZhcmlhYmxlcyBhcyBpbiB0aGUgcHJldmlvdXMgbW9kZWwNCg0KYGBge3J9DQpnZXRfY29lZmZzKGRfZ2xtZXJfY3N0YywgIGMoInNsb3BlLmN0IiwgInNlc3Npb24uY3Q6c2xvcGUuY3QiLCAic2Vzc2lvbi5jdDpMVl9WRVJTVVNfSFY6c2xvcGUuY3QiLCAic2Vzc2lvbi5jdDpMVl9WRVJTVVNfSFZCOnNsb3BlLmN0IiwgICAic2Vzc2lvbi5jdDp3b3JkdHlwZS5jdDpMVl9WRVJTVVNfSFY6c2xvcGUuY3QiLCAic2Vzc2lvbi5jdDp3b3JkdHlwZS5jdDpMVl9WRVJTVVNfSFZCOnNsb3BlLmN0IikpDQpgYGAgDQoNCiMgVHJhaW5pbmcgKFNlY3Rpb24gMy4zKQ0KDQojIyBQYXJhbWV0ZXJzIHdpdGggbGFiZWxzDQoNCmBgYHtyfQ0KIyMgTm90ZTogdGhlIElEX3RyYWluIGRhdGEgZnJhbWUgYWxyZWFkeSBoYXMgYXB0aXR1ZGUyIGFuZCBhcHRpdHVkZTMgaW4gDQoNCnRyYWluJGNvbmRpdGlvbiA9IGZhY3Rvcih0cmFpbiRjb25kaXRpb24sIGxldmVscyA9IGMoIjAiLCIyIiwiMSIpLCBsYWJlbHMgPSBjKCJMViIsICJIVkIiLCAiSFYiKSkNCg0KdHJhaW4yID0gbWVyZ2UoSURfcHJlLCB0cmFpbikNCg0KbWVhbnMgPSB3aXRoKHRyYWluMiwgYWdncmVnYXRlKHNjb3JlIH4gc3ViamVjdCArIHNlc3Npb24gKyBjb25kaXRpb24gKyBhcHRpdHVkZTIsIEZVTiA9IG1lYW4pKQ0KbWVhbnMyID0gd2l0aCh0cmFpbjIsIGFnZ3JlZ2F0ZShzY29yZSB+IHN1YmplY3QgKyBzZXNzaW9uICsgY29uZGl0aW9uICsgYXB0aXR1ZGUzLCBGVU4gPSBtZWFuKSkNCmBgYA0KDQojIyBGaWd1cmVzDQoNCmBgYHtyfQ0KeCA8LSBzdW1tYXJ5U0V3aXRoaW4obWVhbnMsIG1lYXN1cmV2YXIgPSAic2NvcmUiLCBiZXR3ZWVudmFycyA9IGMoImNvbmRpdGlvbiIpLCB3aXRoaW52YXJzID0gYygic2Vzc2lvbiIpLCBpZHZhciA9ICJzdWJqZWN0IiwgbmEucm0gPSBGQUxTRSwgY29uZi5pbnRlcnZhbCA9IC45NSkNCmdyYXBoZGF0YSA8LSBzdW1tYXJ5U0V3aXRoaW4obWVhbnMsIG1lYXN1cmV2YXIgPSAic2NvcmUiLCBiZXR3ZWVudmFycyA9IGMoImNvbmRpdGlvbiIsICJhcHRpdHVkZTIiKSwgd2l0aGludmFycyA9IGMoInNlc3Npb24iKSwgaWR2YXIgPSAic3ViamVjdCIsIG5hLnJtID0gRkFMU0UsIGNvbmYuaW50ZXJ2YWwgPS45NSkNCmdyYXBoZGF0YTIgPC0gc3VtbWFyeVNFd2l0aGluKG1lYW5zMiwgbWVhc3VyZXZhciA9ICJzY29yZSIsIGJldHdlZW52YXJzID0gYygiY29uZGl0aW9uIiwgImFwdGl0dWRlMyIpLCB3aXRoaW52YXJzID0gYygic2Vzc2lvbiIpLCBpZHZhciA9ICJzdWJqZWN0IiwgbmEucm0gPSBGQUxTRSwgY29uZi5pbnRlcnZhbCA9IC45NSkNCmBgYA0KDQojIyMgTWFpbiBHcmFwaCAoRmlndXJlIDQpDQoNCmBgYHtyfQ0KVHAxID0gZ2dwbG90KHgsIGFlcyh4ID0gc2Vzc2lvbiwgeSA9IHNjb3JlLCBncm91cCA9IGNvbmRpdGlvbiwgY29sb3VyID0gY29uZGl0aW9uKSkgKyBnZW9tX2xpbmUoYWVzKGxpbmV0eXBlID0gY29uZGl0aW9uKSwgc2l6ZSA9IDAuOCkgKyBnZW9tX3BvaW50KGFlcyhzaGFwZSA9IGNvbmRpdGlvbiksIHNpemUgPSAyLjUpDQpUcDEgPSBUcDEgKyBnZW9tX2Vycm9yYmFyKGFlcyh5bWluID0gc2NvcmUtY2ksIHltYXggPSBzY29yZStjaSksIHdpZHRoID0gLjIpDQpUcDEgPSBUcDEgKyBzY2FsZV9saW5ldHlwZV9kaXNjcmV0ZShuYW1lID0gIlZhcmlhYmlsaXR5IENvbmRpdGlvbiIsIGJyZWFrcyA9IGMoIkxWIiwiSFYiLCAiSFZCIiksIGxhYmVscyA9IGMoIkxWIiwgIkhWIiwgIkhWQiIpKSArIHNjYWxlX3NoYXBlX2Rpc2NyZXRlKG5hbWUgPSAiVmFyaWFiaWxpdHkgQ29uZGl0aW9uIiwgYnJlYWtzID0gYygiTFYiLCJIViIsICJIVkIiKSwgbGFiZWxzID0gYygiTFYiLCAiSFYiLCAiSFZCIikpICsgc2NhbGVfY29sb3VyX2dyZXkobmFtZSA9ICJWYXJpYWJpbGl0eSBDb25kaXRpb24iLCBicmVha3MgPSBjKCJMViIsIkhWIiwgIkhWQiIpLCBsYWJlbHMgPSBjKCJMViIsICJIViIsICJIVkIiKSkNClRwMSA9IFRwMSArIGNvb3JkX2NhcnRlc2lhbih5bGltID0gYygwLjUsIDEuMCkpDQpUcDEgPSBUcDEgKyB0aGVtZV9idygpDQpUcDEgPSBUcDEgKyBnZ3RpdGxlKCdUcmFpbmluZycpDQpUcDEgPSBUcDEgKyB0aGVtZShwYW5lbC5iYWNrZ3JvdW5kID0gZWxlbWVudF9yZWN0KGZpbGwgPSAnd2hpdGUnKSwgcGFuZWwuZ3JpZC5tYWpvciA9IGVsZW1lbnRfbGluZShjb2xvdXIgPSAid2hpdGUiKSkNClRwMSA9IFRwMSArIGxhYnMoeCA9ICJUcmFpbmluZyBTZXNzaW9uIiwgeSA9ICJNZWFuIEFjY3VyYWN5IikNCnByaW50KFRwMSkNCmBgYA0KDQojIyMgUENQVCBHcmFwaCAoRmlndXJlIDEwLCBCb3R0b20gcGFuZWwpDQoNCmBgYHtyfQ0KVHAyID0gZ2dwbG90KGdyYXBoZGF0YSwgYWVzKHggPSBzZXNzaW9uLCB5ID0gc2NvcmUsIGdyb3VwID0gY29uZGl0aW9uLCBjb2xvdXIgPSBjb25kaXRpb24pKSArIGdlb21fbGluZShhZXMobGluZXR5cGUgPSBjb25kaXRpb24pLHNpemUgPSAwLjgpICsgZ2VvbV9wb2ludChhZXMoc2hhcGUgPSBjb25kaXRpb24pLCBzaXplID0gMi41KQ0KVHAyID0gVHAyICsgZmFjZXRfd3JhcCh+YXB0aXR1ZGUyLCBuY29sID0gMikNClRwMiA9IFRwMiArIGdlb21fZXJyb3JiYXIoYWVzKHltaW4gPSBzY29yZS1jaSwgeW1heCA9IHNjb3JlK2NpKSwgd2lkdGggPSAuMikNClRwMiA9IFRwMiArIHRoZW1lX2J3KCkNClRwMiA9IFRwMiArIHNjYWxlX2xpbmV0eXBlX2Rpc2NyZXRlKG5hbWUgPSAiVmFyaWFibGl0eSBDb25kdGlvbiIsIGJyZWFrcyA9IGMoIkxWIiwiSFYiLCAiSFZCIiksIGxhYmVscyA9IGMoIkxWIiwgIkhWIiwgIkhWQiIpKSArIHNjYWxlX3NoYXBlX2Rpc2NyZXRlKG5hbWUgPSAiVmFyaWFibGl0eSBDb25kdGlvbiIsIGJyZWFrcyA9IGMoIkxWIiwiSFYiLCAiSFZCIiksIGxhYmVscyA9IGMoIkxWIiwgIkhWIiwgIkhWQiIpKSsgc2NhbGVfY29sb3VyX2dyZXkobmFtZSA9ICJWYXJpYWJsaXR5IENvbmR0aW9uIiwgYnJlYWtzID0gYygiTFYiLCJIViIsICJIVkIiKSwgbGFiZWxzID0gYygiTFYiLCAiSFYiLCAiSFZCIikpDQpUcDIgPSBUcDIgKyBjb29yZF9jYXJ0ZXNpYW4oeWxpbSA9IGMoMC41LCAxLjApKQ0KVHAyID0gVHAyICsgZ2d0aXRsZSgnVHJhaW5pbmcnKQ0KVHAyID0gVHAyICsgdGhlbWUocGFuZWwuYmFja2dyb3VuZCA9IGVsZW1lbnRfcmVjdChmaWxsID0gJ3doaXRlJyksIHBhbmVsLmdyaWQubWFqb3IgPSBlbGVtZW50X2xpbmUoY29sb3VyID0gIndoaXRlIikpDQpUcDIgPSBUcDIgKyBsYWJzKHggPSAiVHJhaW5pbmcgU2Vzc2lvbiIsIHkgPSAiTWVhbiBBY2N1cmFjeSIpDQpwcmludChUcDIpDQpgYGANCg0KIyMjIENTVEMgZ3JhcGggKE5vdCB1c2VkKQ0KDQpgYGB7cn0NClRwMyA9IGdncGxvdChncmFwaGRhdGEyLCBhZXMoeCA9IHNlc3Npb24sIHkgPSBzY29yZSwgZ3JvdXAgPSBjb25kaXRpb24sIGNvbG91ciA9IGNvbmRpdGlvbikpICsgZ2VvbV9saW5lKGFlcyhsaW5ldHlwZSA9IGNvbmRpdGlvbiksc2l6ZSA9IDAuOCkgKyBnZW9tX3BvaW50KGFlcyhzaGFwZSA9IGNvbmRpdGlvbiksIHNpemUgPSAyLjUpDQpUcDMgPSBUcDMgKyBmYWNldF93cmFwKH4gYXB0aXR1ZGUzLCBuY29sID0gMikNClRwMyA9IFRwMyArIGdlb21fZXJyb3JiYXIoYWVzKHltaW4gPSBzY29yZS1jaSwgeW1heCA9IHNjb3JlK2NpKSwgd2lkdGggPSAuMikNClRwMyA9IFRwMyArIHRoZW1lX2J3KCkNClRwMyA9IFRwMyArIHNjYWxlX2xpbmV0eXBlX2Rpc2NyZXRlKG5hbWUgPSAiVmFyaWFiaWxpdHkgQ29uZGl0aW9uIiwgYnJlYWtzID0gYygiTFYiLCJIViIsICJIVkIiKSwgbGFiZWxzID0gYygiTFYiLCAiSFYiLCAiSFZCIikpICsgc2NhbGVfc2hhcGVfZGlzY3JldGUobmFtZSA9ICJWYXJpYWJpbGl0eSBDb25kaXRpb24iLCBicmVha3MgPSBjKCJMViIsIkhWIiwgIkhWQiIpLCBsYWJlbHMgPSBjKCJMViIsICJIViIsICJIVkIiKSkgKyBzY2FsZV9jb2xvdXJfZ3JleShuYW1lID0gIlZhcmlhYmlsaXR5IENvbmRpdGlvbiIsIGJyZWFrcyA9IGMoIkxWIiwiSFYiLCAiSFZCIiksIGxhYmVscyA9IGMoIkxWIiwgIkhWIiwgIkhWQiIpKQ0KVHAzID0gVHAzICsgY29vcmRfY2FydGVzaWFuKHlsaW0gPSBjKDAuNSwgMS4wKSkNClRwMyA9IFRwMyArIGdndGl0bGUoJ1RyYWluaW5nJykNClRwMyA9IFRwMyArIHRoZW1lKHBhbmVsLmJhY2tncm91bmQgPSBlbGVtZW50X3JlY3QoZmlsbCA9ICd3aGl0ZScpLCBwYW5lbC5ncmlkLm1ham9yID0gZWxlbWVudF9saW5lKGNvbG91ciA9ICJ3aGl0ZSIpKQ0KVHAzID0gVHAzICsgbGFicyh4ID0gIlRyYWluaW5nIFNlc3Npb24iLCB5ID0gIk1lYW4gQWNjdXJhY3kiKQ0KcHJpbnQoVHAzKQ0KYGBgDQoNCiMjIFN0YXRpc3RpY2FsIEFuYWx5c2lzDQoNCiMjIyBNYWluIE1vZGVsICh3aXRob3V0IGFwdGl0dWRlIHByZWRpY3RvcnM7IFNlY3Rpb24gMy4zKQ0KDQpgYGB7cn0NCiMjIE5vdGUgdGhhdCBhIGZ1bGwgbW9kZWwgd2l0aCBhbGwgdGhyZWUgY29uZGl0aW9ucyBpbiBkaWRuJ3QgY29udmVyZ2Ugc28gbW9kZWwgaXMgc3BsaXQgaW50byB0d28gKGNvbnZlcmdpbmcpIG1vZGVscw0KdHJhaW4yID0gbGl6Q29udHJhc3RzKHRyYWluMiwgdHJhaW4yJGNvbmRpdGlvbiwgIkxWIikNCnRyYWluMiRhcHRpdHVkZTIgPSByZWxldmVsKHRyYWluMiRhcHRpdHVkZTIsIHJlZj0iTEEiKQ0KdHJhaW4yID0gbGl6Q2VudGVyKHRyYWluMiwgbGlzdCgic2Vzc2lvbiIsImFwdGl0dWRlX3BjcHQiLCAiYXB0aXR1ZGUyIikpDQp0cmFpbjIubm9IVkIgPSBsaXpDZW50ZXIoc3Vic2V0KHRyYWluMiwgY29uZGl0aW9uICE9ICJIVkIiKSwgbGlzdCgic2Vzc2lvbiIsICJjb25kaXRpb24iLCAiYXB0aXR1ZGVfcGNwdCIsICJzbG9wZSIsICJhcHRpdHVkZTIiKSkNCnRyYWluMi5ub0hWID0gbGl6Q2VudGVyKHN1YnNldCh0cmFpbjIsIGNvbmRpdGlvbiAhPSAiSFYiKSwgbGlzdCgic2Vzc2lvbiIsICJjb25kaXRpb24iLCAiYXB0aXR1ZGVfcGNwdCIsICJzbG9wZSIsImFwdGl0dWRlMiIpKQ0KDQp0X2dsbWVyX0xWdkhWID0gZ2xtZXIoc2NvcmUgfiBzZXNzaW9uLmN0ICogY29uZGl0aW9uLmN0DQogICAgICAgICAgICAgICsgKHNlc3Npb258fHN1YmplY3QpDQogICAgICAgICAgICAgICwgZmFtaWx5PSJiaW5vbWlhbCIsIA0KICAgICAgICAgICAgICBjb250cm9sID0gZ2xtZXJDb250cm9sKG9wdGltaXplciA9ICJib2J5cWEiKSwgDQogICAgICAgICAgICAgIGRhdGEgPSB0cmFpbjIubm9IVkIpDQoNCnRfZ2xtZXJfTFZ2SFZCID0gZ2xtZXIoc2NvcmUgfiBzZXNzaW9uLmN0ICogY29uZGl0aW9uLmN0DQogICAgICAgICAgICAgICsgKHNlc3Npb258fHN1YmplY3QpDQogICAgICAgICAgICAgICwgZmFtaWx5ID0gImJpbm9taWFsIiwgDQogICAgICAgICAgICAgIGNvbnRyb2wgPSBnbG1lckNvbnRyb2wob3B0aW1pemVyID0gImJvYnlxYSIpLCANCiAgICAgICAgICAgICAgZGF0YSA9IHRyYWluMi5ub0hWKQ0KYGBgDQoNCiMjIyMgUHJpbnQga2V5IGNvZmZpY2llbnRzDQoNCmBgYHtyfQ0Ka2FibGUoc3VtbWFyeSh0X2dsbWVyX0xWdkhWKSRjb2VmZmljaWVudHMsIGRpZ2l0cyA9IDYpDQprYWJsZShzdW1tYXJ5KHRfZ2xtZXJfTFZ2SFZCKSRjb2VmZmljaWVudHMsIGRpZ2l0cyA9IDMpDQpgYGANCg0KIyMjIEFuYWx5c2lzIHdpdGggUENQVCBhY2N1cmFjeSAoU2VjdGlvbiAzLjcpDQpgYGB7cn0NCiMgTm90ZTogbW9kZWxzIHNlcGFyYXRlIEhWICYgSFZCIHdvbid0IGNvbnZlcmdlIHdpdGggYm90aCBhcHRpdHVkZSBhbmQgc2Vzc2lvbiBhcyBwcmVkaWN0b3JzLiBUaGUgb3JpZ2luYWwgbW9kZWwgd2l0aCBhbGwgY29uZGl0aW9ucyBidXQgd2l0aG91dCBzZXNzaW9uIGlzIHVzZWQuIA0KDQoNCnRfZ2xtZXJfcGNwdCA9IGdsbWVyKHNjb3JlIH4gKExWX1ZFUlNVU19IViArIExWX1ZFUlNVU19IVkIpICogYXB0aXR1ZGVfcGNwdC5jdCANCiAgICAgICAgICAgICAgICArICgxfHN1YmplY3QpDQogICAgICAgICAgICAgICwgZmFtaWx5ID0gImJpbm9taWFsIiwgDQogICAgICAgICAgICAgIGNvbnRyb2wgPSBnbG1lckNvbnRyb2wob3B0aW1pemVyID0gImJvYnlxYSIpLCANCiAgICAgICAgICAgICAgZGF0YSA9IHRyYWluMikNCg0KYGBgDQoNCg0KDQojIyMjIFByaW50IGtleSBjb2ZmaWNpZW50cw0KDQpgYGB7cn0NCmthYmxlIChyb3VuZChzdW1tYXJ5KHRfZ2xtZXJfcGNwdCkkY29lZmZpY2llbnRzLCA2KSwgZGlnaXRzID0gMykNCg0KDQpgYGANCg0KIyMjIEV4dHJhIGFuYWx5c2lzIHJlcXVlc3RlZCBieSByZXZpZXdlcnMsIHdpdGggY2F0ZWdvcmljYWwgYXB0aXR1ZGUgbWVhc3VyZSBmcm9tIFBDUFQgYWNjdXJhY3kNCg0KYGBge3J9DQojIE5vdGU6IG1vZGVscyBzZXBhcmF0ZSBIViAmIEhWQiB3b24ndCBjb252ZXJnZSB3aXRoIGJvdGggYXB0aXR1ZGUgYW5kIHNlc3Npb24gYXMgcHJlZGljdG9ycy4gVGhlIG9yaWdpbmFsIG1vZGVsIHdpdGggYWxsIGNvbmRpdGlvbnMgYnV0IHdpdGhvdXQgc2Vzc2lvbiBpcyB1c2VkLiANCg0KDQp0X2dsbWVyX3BjcHQyID0gZ2xtZXIoc2NvcmUgfiAoTFZfVkVSU1VTX0hWICsgTFZfVkVSU1VTX0hWQikgKiBhcHRpdHVkZTIuY3QgDQogICAgICAgICAgICAgICAgKyAoMXxzdWJqZWN0KQ0KICAgICAgICAgICAgICAsIGZhbWlseSA9ICJiaW5vbWlhbCIsIA0KICAgICAgICAgICAgICBjb250cm9sID0gZ2xtZXJDb250cm9sKG9wdGltaXplciA9ICJib2J5cWEiKSwgDQogICAgICAgICAgICAgIGRhdGEgPSB0cmFpbjIpDQoNCmBgYA0KDQojIyMjIFByaW50IGtleSBjb2ZmaWNpZW50cw0KDQpgYGB7cn0NCmthYmxlIChyb3VuZChzdW1tYXJ5KHRfZ2xtZXJfcGNwdDIpJGNvZWZmaWNpZW50cywgNiksIGRpZ2l0cyA9IDMpDQpgYGANCg0KIyMjIEFuYWx5c2lzIHdpdGggQ1NUQyBzbG9wZQ0KDQpgYGB7cn0NCiMgTm90ZTogZnVsbCBtb2RlbCBkaWRuJ3QgY29udmVyZ2UsIHNvIHJlbW92ZWQgc2xvcGUuY3Q6Y29uZGl0aW9uLmN0OnNlc3Npb24uY3QNCg0KdF9nbG1lcl9MVnZIVl9jc3RjID0gZ2xtZXIoc2NvcmUgfiBzZXNzaW9uLmN0ICogY29uZGl0aW9uLmN0ICsgc2xvcGUuY3QgKyAoc2xvcGUuY3QgOiBjb25kaXRpb24uY3QpDQogICAgICAgICAgICAgICsgKHNlc3Npb258fHN1YmplY3QpDQogICAgICAgICAgICAgICwgZmFtaWx5ID0gImJpbm9taWFsIiAsIA0KICAgICAgICAgICAgICBjb250cm9sID0gZ2xtZXJDb250cm9sKG9wdGltaXplciA9ICJib2J5cWEiKSwgDQogICAgICAgICAgICAgIGRhdGEgPSB0cmFpbjIubm9IVkIpDQoNCmthYmxlIChyb3VuZChzdW1tYXJ5KHRfZ2xtZXJfTFZ2SFZfY3N0YykkY29lZmZpY2llbnRzLCA2KSwgZGlnaXRzID0gMykNCg0KdF9nbG1lcl9MVnZIVkJfY3N0YyA9IGdsbWVyKHNjb3JlIH4gc2Vzc2lvbi5jdCAqIGNvbmRpdGlvbi5jdCArIHNsb3BlLmN0ICsgKHNsb3BlLmN0IDogY29uZGl0aW9uLmN0KQ0KICAgICAgICAgICAgICArIChzZXNzaW9ufHxzdWJqZWN0KQ0KICAgICAgICAgICAgICAsIGZhbWlseSA9ICJiaW5vbWlhbCIsIA0KICAgICAgICAgICAgICBjb250cm9sID0gZ2xtZXJDb250cm9sKG9wdGltaXplciA9ICJib2J5cWEiKSwgDQogICAgICAgICAgICAgIGRhdGEgPSB0cmFpbjIubm9IVikNCg0Ka2FibGUgKHJvdW5kKHN1bW1hcnkodF9nbG1lcl9MVnZIVkJfY3N0YykkY29lZmZpY2llbnRzLCA2KSwgZGlnaXRzID0gMykNCmBgYA0KDQojIFBpY3R1cmUgSWRlbnRpZmljYXRpb24gKFNlY3Rpb24gMy40LjIpDQoNCiMjIExhYmVsIFBhcmFtZXRlcnMNCg0KYGBge3J9DQpQSSRjb25kaXRpb24gPSBmYWN0b3IoUEkkY29uZGl0aW9uLCBsZXZlbHMgPSBjKCIwIiwgIjEiLCAiMiIpLCBsYWJlbHMgPSBjKCJMViIsICJIViIsICJIVkIiKSkNClBJJHZvaWNldHlwZSA9IGZhY3RvcihQSSR2b2ljZXR5cGUsIGxldmVscyA9IGMoIm52MSIsICJ0djEiKSwgbGFiZWxzID0gYygiVW50cmFpbmVkIFZvaWNlIiwgIlRyYWluZWQgdm9pY2UiKSkNCg0KUEkyID0gbWVyZ2UoSURfcHJlLCBQSSkNCmBgYA0KDQojIyBGaWd1cmVzDQoNCmBgYHtyfQ0KbWVhbnMgPSB3aXRoKFBJMiwgYWdncmVnYXRlKHNjb3JlIH4gc3ViamVjdCArIGNvbmRpdGlvbiArIHZvaWNldHlwZSwgRlVOID0gbWVhbikpDQpJRG1lYW5zID0gd2l0aCAoUEkyLCBhZ2dyZWdhdGUoYXB0aXR1ZGVfcGNwdCB+IHN1YmplY3QgKyBjb25kaXRpb24gKyB2b2ljZXR5cGUsIEZVTiA9IG1lYW4pKQ0KbWVhbnMgPSBtZXJnZSAobWVhbnMsIElEbWVhbnMpDQptZWFuc2IgPSB3aXRoKFBJMiwgYWdncmVnYXRlKHNjb3JlIH4gc3ViamVjdCArIGNvbmRpdGlvbiArIHZvaWNldHlwZSArIGFwdGl0dWRlMiwgRlVOID0gbWVhbikpDQptZWFuczJiID0gd2l0aChQSTIsIGFnZ3JlZ2F0ZShzY29yZSB+IHN1YmplY3QgKyBjb25kaXRpb24gKyB2b2ljZXR5cGUgKyBhcHRpdHVkZTMsIEZVTiA9IG1lYW4pKQ0KDQp4IDwtIHN1bW1hcnlTRXdpdGhpbihtZWFucywgbWVhc3VyZXZhciA9ICJzY29yZSIsIGJldHdlZW52YXJzID0gYygiY29uZGl0aW9uIiksIHdpdGhpbnZhcnMgPSBjKCJ2b2ljZXR5cGUiKSwgaWR2YXIgPSAic3ViamVjdCIsIG5hLnJtID0gRkFMU0UsIGNvbmYuaW50ZXJ2YWwgPSAuOTUpDQpncmFwaGRhdGEgPC0gc3VtbWFyeVNFd2l0aGluKG1lYW5zYiwgbWVhc3VyZXZhciA9ICJzY29yZSIsIGJldHdlZW52YXJzID0gYygiY29uZGl0aW9uIiwgImFwdGl0dWRlMiIpLCB3aXRoaW52YXJzID0gYygidm9pY2V0eXBlIiksIGlkdmFyID0gInN1YmplY3QiLCBuYS5ybSA9IEZBTFNFLCBjb25mLmludGVydmFsID0gLjk1KQ0KZ3JhcGhkYXRhMiA8LSBzdW1tYXJ5U0V3aXRoaW4obWVhbnMyYiwgbWVhc3VyZXZhciA9ICJzY29yZSIsIGJldHdlZW52YXJzID0gYygiY29uZGl0aW9uIiwgImFwdGl0dWRlMyIpLCB3aXRoaW52YXJzID0gYygidm9pY2V0eXBlIiksIGlkdmFyID0gInN1YmplY3QiLCBuYS5ybSA9IEZBTFNFLCBjb25mLmludGVydmFsID0gLjk1KQ0KYGBgDQoNCiMjIyBNYWluIEdyYXBoIChGaWd1cmUgNikNCg0KYGBge3J9DQpQSXAxID0gZ2dwbG90IChtZWFucywgYWVzKHggPSBjb25kaXRpb24sIHkgPSBzY29yZSwgZmlsbCA9IHZvaWNldHlwZSkpIA0KUElwMSA9IFBJcDEgKyBnZW9tX3Zpb2xpbihjb2xvdXIgPSAiYmxhY2siLCBzY2FsZSA9ICJjb3VudCIpDQpQSXAxID0gUElwMSArIHNjYWxlX2ZpbGxfbWFudWFsKG5hbWUgPSAiVm9pY2UgdHlwZSIsIHZhbHVlcyA9IGMoIlVudHJhaW5lZCBWb2ljZSIgPSAiZ3JleSIsICJUcmFpbmVkIHZvaWNlIiA9ICJ3aGl0ZSIpLCBsYWJlbHMgPSBjKCJVbnRyYWluZWQgVm9pY2UiLCAiVHJhaW5lZCB2b2ljZSIpKQ0KUElwMSA9IFBJcDEgKyBzY2FsZV94X2Rpc2NyZXRlKGxhYmVscyA9IGMoIkxWIiwgIkhWIiwgIkhWQiIpKQ0KUElwMSA9IFBJcDEgKyBnZW9tX2Vycm9yYmFyKGFlcyh5bWluID0gc2NvcmUtY2ksIHltYXggPSBzY29yZStjaSksIHdpZHRoID0gLjQsIHBvc2l0aW9uID0gcG9zaXRpb25fZG9kZ2UoLjkpLCBkYXRhID0geCkNClBJcDEgPSBQSXAxICsgc3RhdF9zdW1tYXJ5KGZ1bi55ID0gbWVhbiwgZ2VvbSA9ICJwb2ludCIsIHNoYXBlID0gMjMsIHNpemUgPSAyLHBvc2l0aW9uID0gcG9zaXRpb25fZG9kZ2UoLjkpKQ0KUElwMSA9IFBJcDEgKyBnZ3RpdGxlKCdQaWN0dXJlIElkZW50aWZpY2F0aW9uJykNClBJcDEgPSBQSXAxICsgbGFicyh4ID0gIlZhcmlhYmlsaXR5IENvbmRpdGlvbiIsIHkgPSAiTWVhbiBBY2N1cmFjeSIpDQpQSXAxID0gUElwMSArIHRoZW1lX2J3KCkNCnByaW50KFBJcDEpDQpgYGANCg0KIyMjIE1haW4gR3JhcGggMiAoU2NhdHRlciBQbG90LCBGaWd1cmUgMTEsIEJvdHRvbSBwYW5lbCwgcmlnaHQpDQoNCmBgYHtyfQ0KUElwMC4xbSA9IGdncGxvdChtZWFucywgYWVzKGFwdGl0dWRlX3BjcHQsIHNjb3JlLCBzaGFwZSA9IGNvbmRpdGlvbiwgY29sb3VyID0gY29uZGl0aW9uKSkgDQpQSXAwLjFtID0gUElwMC4xbSArIGdlb21fcG9pbnQoYWVzIChzaGFwZSA9IGNvbmRpdGlvbiksIHNpemUgPSAyKSArIGdlb21fc21vb3RoKHNlID0gRkFMU0UsIG1ldGhvZCA9IGxtLCBhZXMobGluZXR5cGUgPSBjb25kaXRpb24pKQ0KUElwMC4xbSA9IFBJcDAuMW0gKyBzY2FsZV9saW5ldHlwZV9kaXNjcmV0ZShuYW1lID0gIiAiLCBicmVha3MgPSBjKCJMViIsIkhWIiwgIkhWQiIpLCBsYWJlbHMgPSBjKCJMViIsICJIViIsICJIVkIiKSkgKyBzY2FsZV9zaGFwZV9kaXNjcmV0ZShuYW1lID0gIiAiLCBicmVha3MgPSBjKCJMViIsICJIViIsICJIVkIiKSwgbGFiZWxzID0gYygiTFYiLCAiSFYiLCAiSFZCIikpICsgc2NhbGVfY29sb3VyX2dyZXkobmFtZSA9ICIgIiwgYnJlYWtzID0gYygiTFYiLCJIViIsICJIVkIiKSwgbGFiZWxzID0gYygiTFYiLCAiSFYiLCAiSFZCIikpDQpQSXAwLjFtID0gUElwMC4xbSArIGxhYnMoeCA9ICJQQ1BUIFNjb3JlIiwgeSA9ICJNZWFuIEFjY3VyYWN5IikgKyAgZ2d0aXRsZSgnICcpDQpwcmludChQSXAwLjFtKQ0KYGBgDQoNCiMjIyBQQ1BUIEdyYXBoIChGaWd1cmUgMTEgQm90dG9tIHBhbmVsLCBsZWZ0KQ0KDQpgYGB7cn0NClBJcDIgPSBnZ3Bsb3QgKG1lYW5zYiwgYWVzKHggPSBjb25kaXRpb24sIHkgPSBzY29yZSwgZmlsbCA9IHZvaWNldHlwZSkpIA0KUElwMiA9IFBJcDIgKyBmYWNldF93cmFwKH5hcHRpdHVkZTIsIG5jb2wgPSAyKQ0KUElwMiA9IFBJcDIgKyBnZW9tX3Zpb2xpbihjb2xvdXIgPSAiYmxhY2siLCBzY2FsZSA9ICJjb3VudCIpDQpQSXAyID0gUElwMiArIHNjYWxlX2ZpbGxfbWFudWFsKG5hbWUgPSAiVm9pY2UgdHlwZSIsIHZhbHVlcyA9IGMoIlVudHJhaW5lZCBWb2ljZSIgPSAiZ3JleSIsICJUcmFpbmVkIHZvaWNlIiA9ICJ3aGl0ZSIpLCBsYWJlbHMgPSBjKCJVbnRyYWluZWQgVm9pY2UiLCAiVHJhaW5lZCB2b2ljZSIpKQ0KUElwMiA9IFBJcDIgKyBzY2FsZV94X2Rpc2NyZXRlKGxhYmVscyA9IGMoIkxWIiwgIkhWIiwgIkhWQiIpKQ0KUElwMiA9IFBJcDIgKyBnZW9tX2Vycm9yYmFyKGFlcyh5bWluID0gc2NvcmUtY2ksIHltYXggPSBzY29yZStjaSksIHdpZHRoID0gLjQsIHBvc2l0aW9uID0gcG9zaXRpb25fZG9kZ2UoLjkpLCBkYXRhID0gZ3JhcGhkYXRhKQ0KUElwMiA9IFBJcDIgKyBzdGF0X3N1bW1hcnkoZnVuLnkgPSBtZWFuLCBnZW9tID0gInBvaW50Iiwgc2hhcGUgPSAyMywgc2l6ZSA9IDIscG9zaXRpb24gPSBwb3NpdGlvbl9kb2RnZSguOSkpDQpQSXAyID0gUElwMiArIGdndGl0bGUoJ1BpY3R1cmUgSWRlbnRpZmljYXRpb24nKQ0KUElwMiA9IFBJcDIgKyBsYWJzKHggPSAiVmFyaWFiaWxpdHkgQ29uZGl0aW9uIiwgeSA9ICJNZWFuIEFjY3VyYWN5IikNClBJcDIgPSBQSXAyICsgdGhlbWVfYncoKQ0KUElwMi5tID0gUElwMiArIHRoZW1lKGxlZ2VuZC5wb3NpdGlvbiA9IGMoMC44LCAwLjEpLCBsZWdlbmQua2V5LnNpemUgPSB1bml0KDAuMywiY20iKSwgbGVnZW5kLnRpdGxlID0gZWxlbWVudF90ZXh0KHNpemU9MTApLCBsZWdlbmQudGV4dCA9IGVsZW1lbnRfdGV4dChzaXplID0gOCkpDQpwcmludChQSXAyKQ0KYGBgDQoNCiMjIyBDU1RDIGdyYXBoIChOb3QgdXNlZCkNCg0KYGBge3J9DQpQSXAzID0gZ2dwbG90IChtZWFuczJiLCBhZXMoeCA9IGNvbmRpdGlvbiwgeSA9IHNjb3JlLCBmaWxsID0gdm9pY2V0eXBlKSkgDQpQSXAzID0gUElwMyArIGZhY2V0X3dyYXAofmFwdGl0dWRlMywgbmNvbCA9IDIpDQpQSXAzID0gUElwMyArIGdlb21fdmlvbGluKGNvbG91ciA9ICJibGFjayIsIHNjYWxlID0gImNvdW50IikNClBJcDMgPSBQSXAzICsgc2NhbGVfZmlsbF9tYW51YWwobmFtZSA9ICJWb2ljZSB0eXBlIiwgdmFsdWVzID0gYygiVW50cmFpbmVkIFZvaWNlIiA9ICJncmV5IiwgIlRyYWluZWQgdm9pY2UiID0gIndoaXRlIiksIGxhYmVscyA9IGMoIlVudHJhaW5lZCBWb2ljZSIsICJUcmFpbmVkIHZvaWNlIikpDQpQSXAzID0gUElwMyArIHNjYWxlX3hfZGlzY3JldGUobGFiZWxzID0gYygiTG93IFZhcmlhYmlsaXR5IiwgIkhpZ2ggVmFyaWFiaWxpdHkiLCAiSGlnaCBWYXJhaWJpbGl0eSBCbG9ja2luZyIpKQ0KUElwMyA9IFBJcDMgKyBnZW9tX2Vycm9yYmFyKGFlcyh5bWluID0gc2NvcmUtY2ksIHltYXggPSBzY29yZStjaSksIHdpZHRoID0gLjQsIHBvc2l0aW9uID0gcG9zaXRpb25fZG9kZ2UoLjkpLCBkYXRhID0gZ3JhcGhkYXRhMikNClBJcDMgPSBQSXAzICsgc3RhdF9zdW1tYXJ5KGZ1bi55ID0gbWVhbiwgZ2VvbSA9ICJwb2ludCIsIHNoYXBlID0gMjMsIHNpemUgPSAyLHBvc2l0aW9uID0gcG9zaXRpb25fZG9kZ2UoLjkpKQ0KUElwMyA9IFBJcDMgKyBnZ3RpdGxlKCdQaWN0dXJlIElkZW50aWZpY2F0aW9uJykNClBJcDMgPSBQSXAzICsgbGFicyh4ID0gIlZhcmlhYmlsaXR5IENvbmRpdGlvbiIsIHkgPSAiTWVhbiBBY2N1cmFjeSIpDQpQSXAzID0gUElwMyArIHRoZW1lX2J3KCkNCnByaW50KFBJcDMpDQpgYGANCg0KIyMgU3RhdGlzdGljYWwgQW5hbHlzaXMNCg0KIyMjIE1haW4gTW9kZWwgKHdpdGhvdXQgaW5kaXZpZHVhbCBkaWZmZXJlbmNlIG1lYXN1cmVzOyBTZWN0aW9uIDMuNC4yKQ0KDQpgYGB7cn0NClBJMiA9IGxpekNlbnRlcihQSTIsIGxpc3QoInZvaWNldHlwZSIsICJhcHRpdHVkZV9wY3B0IiwgInNsb3BlIikpDQpQSTIgPSBsaXpDb250cmFzdHMoUEkyLCBQSTIkY29uZGl0aW9uLCAiTFYiKQ0KDQpwX2dsbWVyID0gZ2xtZXIoc2NvcmUgfiB2b2ljZXR5cGUuY3QgKiAoTFZfVkVSU1VTX0hWICsgTFZfVkVSU1VTX0hWQikNCiAgICAgICAgICAgICAgKyAodm9pY2V0eXBlLmN0fHN1YmplY3QpDQogICAgICAgICAgICAgICwgZmFtaWx5ID0gImJpbm9taWFsIiwgDQogICAgICAgICAgICAgIGNvbnRyb2wgPSBnbG1lckNvbnRyb2wob3B0aW1pemVyID0gImJvYnlxYSIpLCANCiAgICAgICAgICAgICAgZGF0YSA9IFBJMikNCmBgYA0KDQojIyMjIFByaW50IG91dCBrZXkgZWZmZWN0cw0KDQpgYGB7cn0NCmthYmxlKHN1bW1hcnkocF9nbG1lcikkY29lZmZpY2llbnRzLCBkaWdpdHMgPSAzKQ0KYGBgDQoNCiMjIyMgUmUtZml0IHRoZSBtb2RlbCB3aXRoIGEgc2VwYXJhdGUgc2xvcGUgZm9yIHZvaWNldHlwLmN0IGZvciBlYWNoIGNvbmRpdGlvbiINCg0KYGBge3J9DQpQSTIgPSBsaXpDZW50ZXIoUEkyLCBsaXN0KCJ2b2ljZXR5cGUiLCAiYXB0aXR1ZGVfcGNwdCIsICJzbG9wZSIpKQ0KUEkyID0gbGl6Q29udHJhc3RzKFBJMiwgUEkyJGNvbmRpdGlvbiwgIkxWIikNCg0KcF9nbG1lcmIgPSBnbG1lcihzY29yZSB+IA0KICAgICAgICAgICAgICAgICAgIHZvaWNldHlwZS5jdCA6ICBjb25kaXRpb24NCiAgICAgICAgICAgICAgICAgKyhMVl9WRVJTVVNfSFYgKyBMVl9WRVJTVVNfSFZCKQ0KICAgICAgICAgICAgICAgICArICh2b2ljZXR5cGUuY3R8c3ViamVjdCkNCiAgICAgICAgICAgICAgICAgLCBmYW1pbHkgPSAiYmlub21pYWwiLA0KICAgICAgICAgICAgICAgICBjb250cm9sID0gZ2xtZXJDb250cm9sKG9wdGltaXplciA9ICJib2J5cWEiKSwgDQogICAgICAgICAgICAgICAgIGRhdGEgPSBQSTIpDQpgYGANCg0KIyMjIyBUbyBjaGVjayBpdCBpcyB0aGUgc2FtZSBtb2RlbA0KDQpgYGB7cn0NCmFub3ZhKHBfZ2xtZXIsIHBfZ2xtZXJiKQ0KYGBgDQoNCiMjIyMgUHJpbnQgb3V0IGVmZmVjdCBvZiB2b2ljZS10eXBlIGZvciBlYWNoIGNvbmRpdGlvbg0KDQpgYGB7cn0NCnJvdW5kKGdldF9jb2VmZnMocF9nbG1lcmIsIGMoInZvaWNldHlwZS5jdDpjb25kaXRpb25MViIsICJ2b2ljZXR5cGUuY3Q6Y29uZGl0aW9uSFYiLCAidm9pY2V0eXBlLmN0OmNvbmRpdGlvbkhWQiIpKSwgMykNCmBgYA0KDQojIyMjIFJlLWZpdCB0aGUgbW9kZWwgd2l0aCBhIHNlcGFyYXRlIHNsb3BlIGZvciB0aGUgY29udHJhc3QgYmV0d2VlbiBjb25kaXRpb25zIGZvciB0cmFpbmVkIGFuZCB1bnRyYWluZWQgbm91bnMNCg0KYGBge3J9DQpQSTIgPSBsaXpDZW50ZXIoUEkyLCBsaXN0KCJ2b2ljZXR5cGUiLCAiYXB0aXR1ZGVfcGNwdCIsICJzbG9wZSIpKQ0KUEkyID0gbGl6Q29udHJhc3RzKFBJMiwgUEkyJGNvbmRpdGlvbiwgIkxWIikNCg0KcF9nbG1lcmMgPSBnbG1lcihzY29yZSB+IA0KICAgICAgICAgICAgICAgIHZvaWNldHlwZSA6IExWX1ZFUlNVU19IViANCiAgICAgICAgICAgICAgICsgdm9pY2V0eXBlIDogTFZfVkVSU1VTX0hWQg0KICAgICAgICAgICAgICAgKyB2b2ljZXR5cGUuY3QgDQogICAgICAgICAgICAgICArICh2b2ljZXR5cGUuY3R8c3ViamVjdCkNCiAgICAgICAgICAgICAgLCBmYW1pbHkgPSAiYmlub21pYWwiLCANCiAgICAgICAgICAgICAgY29udHJvbCA9IGdsbWVyQ29udHJvbChvcHRpbWl6ZXIgPSAiYm9ieXFhIiksIA0KICAgICAgICAgICAgICBkYXRhID0gUEkyKQ0KYGBgDQoNCiMjIyMgVG8gY2hlY2sgaXQgaXMgdGhlIHNhbWUgbW9kZWwNCg0KYGBge3J9DQphbm92YShwX2dsbWVyLCBwX2dsbWVyYykNCmBgYA0KDQojIyMjIFByaW50IG91dCB0aGUgY29udHJhc3QgYmV0d2VlbiBjb25kaXRpb25zIGZvciB0cmFpbmVkIGFuZCB1bnRyYWluZWQgbm91bnMNCg0KYGBge3J9DQprYWJsZShzdW1tYXJ5KHBfZ2xtZXJjKSRjb2VmZiwgZGlnaXRzID0gMykNCmBgYA0KDQojIyMgQW5hbHlzaXMgd2l0aCBQQ1BUIGFjY3VyYWN5IChTZWN0aW9uIDMuNykNCg0KYGBge3J9DQpwX2dsbWVyX3BjcHQgPSBnbG1lcihzY29yZSB+IHZvaWNldHlwZS5jdCAqIChMVl9WRVJTVVNfSFYgKyBMVl9WRVJTVVNfSFZCKSAqIGFwdGl0dWRlX3BjcHQuY3QNCiAgICAgICAgICAgICAgICArICh2b2ljZXR5cGUuY3R8c3ViamVjdCkNCiAgICAgICAgICAgICAgICAsIGZhbWlseSA9ICJiaW5vbWlhbCIsIA0KICAgICAgICAgICAgICAgIGNvbnRyb2wgPSBnbG1lckNvbnRyb2wob3B0aW1pemVyID0gImJvYnlxYSIpLCANCiAgICAgICAgICAgICAgICBkYXRhID0gUEkyKQ0KYGBgDQoNCiMjIyMgQ2hlY2sgdGhhdCB3ZSBnZXQgdGhlIHNhbWUgcGF0dGVybiBvZiByZXN1bHRzIGZvciB0aGUgZXhwZXJpbWVudGFsbHkgbWFuaXB1bGF0ZWQgdmFyaWFibGVzIGFzIGluIHRoZSBwcmV2aW91cyBtb2RlbA0KDQpgYGB7cn0NCmthYmxlKGdldF9jb2VmZnMocF9nbG1lcl9wY3B0LCBjKCJ2b2ljZXR5cGUuY3QiLCAiTFZfVkVSU1VTX0hWIiwgIkxWX1ZFUlNVU19IVkIiLCAidm9pY2V0eXBlLmN0OkxWX1ZFUlNVU19IViIsICJ2b2ljZXR5cGUuY3Q6TFZfVkVSU1VTX0hWQiIpKSwgZGlnaXRzID0gMykNCmBgYA0KDQojIyMjIFByaW50IG91dCB0aGUgZWZmZWN0IG9mIGluZGl2aWR1YWwgYXB0aXR1ZGUgYW5kIGtleSBpbnRlcmFjdGlvbnMgd2l0aCBhcHRpdHVkZQ0KDQpgYGB7cn0NCmthYmxlKGdldF9jb2VmZnMocF9nbG1lcl9wY3B0LCBjKCJhcHRpdHVkZV9wY3B0LmN0IiwgInZvaWNldHlwZS5jdDphcHRpdHVkZV9wY3B0LmN0IiwgIkxWX1ZFUlNVU19IVjphcHRpdHVkZV9wY3B0LmN0IiwgIkxWX1ZFUlNVU19IVkI6YXB0aXR1ZGVfcGNwdC5jdCIsICJ2b2ljZXR5cGUuY3Q6TFZfVkVSU1VTX0hWOmFwdGl0dWRlX3BjcHQuY3QiLCAidm9pY2V0eXBlLmN0OkxWX1ZFUlNVU19IVkI6YXB0aXR1ZGVfcGNwdC5jdCIpKSwgZGlnaXRzID0gMykNCmBgYA0KDQojIyMgQW5hbHlzaXMgd2l0aCBDU1RDIHNsb3BlDQoNCmBgYHtyfQ0KcF9nbG1lcl9jc3RjID0gZ2xtZXIoc2NvcmUgfiB2b2ljZXR5cGUuY3QgKiAoTFZfVkVSU1VTX0hWICsgTFZfVkVSU1VTX0hWQikgKiBzbG9wZS5jdA0KICAgICAgICAgICAgICAgICsgKHZvaWNldHlwZS5jdHxzdWJqZWN0KQ0KICAgICAgICAgICAgICAgICwgZmFtaWx5ID0gImJpbm9taWFsIiwgDQogICAgICAgICAgICAgICAgY29udHJvbCA9IGdsbWVyQ29udHJvbChvcHRpbWl6ZXIgPSAiYm9ieXFhIiksIA0KICAgICAgICAgICAgICAgIGRhdGEgPSBQSTIpDQpgYGANCg0KIyMjIyBDaGVjayB0aGF0IHdlIGdldCB0aGUgc2FtZSBwYXR0ZXJuIG9mIHJlc3VsdHMgZm9yIHRoZSBleHBlcmltZW50YWxseSBtYW5pcHVsYXRlZCB2YXJpYWJsZXMgYXMgaW4gdGhlIHByZXZpb3VzIG1vZGVsDQoNCmBgYHtyfQ0Ka2FibGUoZ2V0X2NvZWZmcyhwX2dsbWVyX2NzdGMsIGMoInZvaWNldHlwZS5jdCIsICJMVl9WRVJTVVNfSFYiLCAgIkxWX1ZFUlNVU19IVkIiLCAidm9pY2V0eXBlLmN0OkxWX1ZFUlNVU19IViIsICJ2b2ljZXR5cGUuY3Q6TFZfVkVSU1VTX0hWQiIpKSwgZGlnaXRzID0gMykNCmBgYA0KDQojIyMjIFByaW50IG91dCB0aGUgZWZmZWN0IG9mIGluZGl2aWR1YWwgYXB0aXR1ZGUgYW5kIGtleSBpbnRlcmFjdGlvbnMgd2l0aCBhcHRpdHVkZQ0KDQpgYGB7cn0NCmthYmxlKGdldF9jb2VmZnMocF9nbG1lcl9jc3RjLCBjKCJzbG9wZS5jdCIsICJ2b2ljZXR5cGUuY3Q6c2xvcGUuY3QiLCAiTFZfVkVSU1VTX0hWOnNsb3BlLmN0IiwgIkxWX1ZFUlNVU19IVkI6c2xvcGUuY3QiLCAidm9pY2V0eXBlLmN0OkxWX1ZFUlNVU19IVjpzbG9wZS5jdCIsICJ2b2ljZXR5cGUuY3Q6TFZfVkVSU1VTX0hWQjpzbG9wZS5jdCIpKSwgZGlnaXRzID0gMykNCmBgYA0KDQojIFBpY3R1cmUgTmFtaW5nIChTZWN0aW9uIDMuNS4yKQ0KDQojIyBQaWN0dXJlIE5hbWluZyAtIFRvbmUgYWNjdXJhY3kNCg0KIyMjIExhYmVsIHBhcmFtZXRlcnMgYW5kIHJlbW92ZSB1bmlkZW50aWZpYWJsZSB0cmlhbHMNCg0KYGBge3J9DQpOUE4gPSBDUE5bQ1BOJHRvbmUgIT0gMCwgXQ0KTlBOJGNvbmRpdGlvbiA9IGZhY3RvcihOUE4kY29uZGl0aW9uLCBsZXZlbHMgPSBjKCIwIiwgIjEiLCAiMiIpLCBsYWJlbHMgPSBjKCJMViIsICJIViIsICJIVkIiKSkNCk5QTjIgPSBtZXJnZShJRF9wcmUsIE5QTikNCmBgYA0KDQojIyMgRmlndXJlcw0KDQpgYGB7cn0NCm1lYW5zID0gd2l0aChOUE4yLCBhZ2dyZWdhdGUodG9uZV9zY29yZSB+IHN1YmplY3QgKyBjb25kaXRpb24sIEZVTiA9IG1lYW4pKQ0KSURtZWFucyA9IHdpdGggKE5QTjIsIGFnZ3JlZ2F0ZShhcHRpdHVkZV9wY3B0IH4gc3ViamVjdCArIGNvbmRpdGlvbiwgRlVOID0gbWVhbikpDQptZWFucyA9IG1lcmdlIChtZWFucywgSURtZWFucykNCm1lYW5zYiA9IHdpdGgoTlBOMiwgYWdncmVnYXRlKHRvbmVfc2NvcmUgfiBzdWJqZWN0ICsgY29uZGl0aW9uICsgYXB0aXR1ZGUyLCBGVU4gPSBtZWFuKSkNCm1lYW5zMmIgPSB3aXRoKE5QTjIsIGFnZ3JlZ2F0ZSh0b25lX3Njb3JlIH4gc3ViamVjdCArIGNvbmRpdGlvbiArIGFwdGl0dWRlMywgRlVOID0gbWVhbikpDQoNCnhiIDwtIHN1bW1hcnlTRShtZWFuc2IsIG1lYXN1cmV2YXIgPSAidG9uZV9zY29yZSIsIGdyb3VwdmFycyA9IGMoImNvbmRpdGlvbiIpLCBuYS5ybSA9IEZBTFNFLCBjb25mLmludGVydmFsID0gLjk1KQ0KZ3JhcGhkYXRhYiA8LSBzdW1tYXJ5U0UobWVhbnNiLCBtZWFzdXJldmFyID0gInRvbmVfc2NvcmUiLCBncm91cHZhcnMgPSBjKCJjb25kaXRpb24iLCAiYXB0aXR1ZGUyIiksIG5hLnJtID0gRkFMU0UsIGNvbmYuaW50ZXJ2YWwgPSAuOTUpDQpncmFwaGRhdGEyYiA8LSBzdW1tYXJ5U0UobWVhbnMyYiwgbWVhc3VyZXZhciA9ICJ0b25lX3Njb3JlIiwgZ3JvdXB2YXJzID0gYygiY29uZGl0aW9uIiwgImFwdGl0dWRlMyIpLCBuYS5ybSA9IEZBTFNFLCBjb25mLmludGVydmFsID0gLjk1KQ0KYGBgDQoNCiMjIyMgTWFpbiBGaWd1cmUgKEZpZ3VyZSA5LCBsZWZ0KQ0KDQpgYGB7cn0NClBOcDEgPSBnZ3Bsb3QgKG1lYW5zLCBhZXMoeCA9IGNvbmRpdGlvbiwgeSA9IHRvbmVfc2NvcmUpKSANClBOcDEgPSBQTnAxICsgZ2VvbV92aW9saW4oY29sb3VyID0gImJsYWNrIiwgc2NhbGUgPSAiY291bnQiKQ0KUE5wMSA9IFBOcDEgKyBzY2FsZV94X2Rpc2NyZXRlKGxhYmVscyA9IGMoIkxWIiwgIkhWIiwgIkhWQiIpKQ0KUE5wMSA9IFBOcDEgKyBnZW9tX2Vycm9yYmFyKGFlcyh5bWluID0gdG9uZV9zY29yZS1jaSwgeW1heCA9IHRvbmVfc2NvcmUrY2kpLCB3aWR0aCA9IC40LCBwb3NpdGlvbiA9IHBvc2l0aW9uX2RvZGdlKC45KSwgZGF0YSA9IHhiKQ0KUE5wMSA9IFBOcDEgKyBzdGF0X3N1bW1hcnkoZnVuLnkgPSBtZWFuLCBnZW9tID0gInBvaW50Iiwgc2hhcGUgPSAyMywgc2l6ZSA9IDIscG9zaXRpb24gPSBwb3NpdGlvbl9kb2RnZSguOSkpDQpQTnAxID0gUE5wMSArdGhlbWVfYncoKSArIGdndGl0bGUoJ1BpY3R1cmUgTmFtaW5nIC0gVG9uZSBhY2N1cmFjeScpDQpQTnAxID0gUE5wMSArIGxhYnMoeCA9ICJWYXJpYWJpbGl0eSBDb25kaXRpb24iLCB5ID0gIk1lYW4gQWNjdXJhY3kiKQ0KcHJpbnQoUE5wMSkNCmBgYA0KDQojIyMjIE1haW4gRmlndXJlIChTY2F0dGVyIHBsb3QsIEZpZ3VyZSAxMSwgVG9wIHBhbmVsLCByaWdodCkNCg0KYGBge3J9DQpQTnAwLjEgPSBnZ3Bsb3QobWVhbnMsIGFlcyhhcHRpdHVkZV9wY3B0LCB0b25lX3Njb3JlLHNoYXBlID0gY29uZGl0aW9uLCBjb2xvdXIgPSBjb25kaXRpb24pKSANClBOcDAuMSA9IFBOcDAuMSArIGdlb21fcG9pbnQoYWVzIChzaGFwZSA9IGNvbmRpdGlvbiksIHNpemUgPSAyKSArIGdlb21fc21vb3RoKHNlID0gRkFMU0UsIG1ldGhvZCA9IGxtLCBhZXMobGluZXR5cGUgPSBjb25kaXRpb24pKQ0KUE5wMC4xID0gUE5wMC4xICsgc2NhbGVfbGluZXR5cGVfZGlzY3JldGUobmFtZSA9ICJWYXJpYWJpbGl0eSBDb25kaXRpb24iLCBicmVha3MgPSBjKCJMViIsIkhWIiwgIkhWQiIpLCBsYWJlbHMgPSBjKCJMViIsICJIViIsICJIVkIiKSkgKyBzY2FsZV9zaGFwZV9kaXNjcmV0ZShuYW1lID0gIlZhcmlhYmlsaXR5IENvbmRpdGlvbiIsIGJyZWFrcyA9IGMoIkxWIiwiSFYiLCAiSFZCIiksIGxhYmVscyA9IGMoIkxWIiwgIkhWIiwgIkhWQiIpKSArIHNjYWxlX2NvbG91cl9ncmV5KG5hbWUgPSAiVmFyaWFiaWxpdHkgQ29uZGl0aW9uIiwgYnJlYWtzID0gYygiTFYiLCJIViIsICJIVkIiKSwgbGFiZWxzID0gYygiTFYiLCAiSFYiLCAiSFZCIikpDQpQTnAwLjEgPSBQTnAwLjEgKyBsYWJzKHggPSAiUENQVCBTY29yZSIseSA9IE5VTEwpICsgZ2d0aXRsZSgnICcpDQoNClBOcDAuMS5tID0gUE5wMC4xICsgc2NhbGVfbGluZXR5cGVfZGlzY3JldGUobmFtZSA9ICIgIiwgYnJlYWtzID0gYygiTFYiLCJIViIsICJIVkIiKSwgbGFiZWxzID0gYygiTFYiLCAiSFYiLCAiSFZCIikpICsgc2NhbGVfc2hhcGVfZGlzY3JldGUobmFtZSA9ICIgIiwgYnJlYWtzID0gYygiTFYiLCJIViIsICJIVkIiKSwgbGFiZWxzID0gYygiTFYiLCAiSFYiLCAiSFZCIikpICsgc2NhbGVfY29sb3VyX2dyZXkobmFtZSA9ICIgIiwgYnJlYWtzID0gYygiTFYiLCJIViIsICJIVkIiKSwgbGFiZWxzID0gYygiTFYiLCAiSFYiLCAiSFZCIikpDQpQTnAwLjEubSA9IFBOcDAuMS5tICsgbGFicyh4ID0gIlBDUFQgU2NvcmUiLCB5ID0gIk1lYW4gQWNjdXJhY3kiKSArIGdndGl0bGUoJyAnKQ0KDQpwcmludChQTnAwLjEpDQpgYGANCg0KIyMjIyBQQ1BUIEdyYXBoIChGaWd1cmUgMTEgVG9wIHBhbmVsLGxlZnQpDQoNCmBgYHtyfQ0KUE5wMiA9IGdncGxvdCAobWVhbnNiLCBhZXMoeCA9IGNvbmRpdGlvbiwgeSA9IHRvbmVfc2NvcmUpKSANClBOcDIgPSBQTnAyICsgZ2VvbV92aW9saW4oY29sb3VyID0gImJsYWNrIiwgc2NhbGUgPSAiY291bnQiKQ0KUE5wMiA9IFBOcDIgKyBmYWNldF93cmFwKH5hcHRpdHVkZTIsIG5jb2wgPSAyKQ0KUE5wMiA9IFBOcDIgKyBzY2FsZV94X2Rpc2NyZXRlKGxhYmVscyA9IGMoIkxWIiwgIkhWIiwgIkhWQiIpKQ0KUE5wMiA9IFBOcDIgKyBnZW9tX2Vycm9yYmFyKGFlcyh5bWluID0gdG9uZV9zY29yZS1jaSwgeW1heCA9IHRvbmVfc2NvcmUrY2kpLCB3aWR0aCA9IC40LCBwb3NpdGlvbiA9IHBvc2l0aW9uX2RvZGdlKC45KSwgZGF0YSA9IGdyYXBoZGF0YWIpDQpQTnAyID0gUE5wMiArIHN0YXRfc3VtbWFyeShmdW4ueSA9IG1lYW4sIGdlb20gPSAicG9pbnQiLCBzaGFwZSA9IDIzLCBzaXplID0gMiwgcG9zaXRpb24gPSBwb3NpdGlvbl9kb2RnZSguOSkpDQpQTnAyID0gUE5wMiArIHRoZW1lX2J3KCkrIGdndGl0bGUoJ1BpY3R1cmUgTmFtaW5nOiBUb25lIGFjY3VyYWN5JykNClBOcDIgPSBQTnAyICsgbGFicyh4ID0gIlZhcmlhYmlsaXR5IENvbmRpdGlvbiIsIHkgPSAiTWVhbiBBY2N1cmFjeSIpDQpwcmludChQTnAyKQ0KYGBgDQoNCiMjIyMgQ1NUQyBncmFwaCAoTm90IHVzZWQpDQoNCmBgYHtyfQ0KUE5wMyA9IGdncGxvdCAobWVhbnMyYiwgYWVzKHggPSBjb25kaXRpb24seSA9IHRvbmVfc2NvcmUsIGZpbGwgPSBhcHRpdHVkZTMpKSANClBOcDMgPSBQTnAzICsgZ2VvbV92aW9saW4oY29sb3VyID0gImJsYWNrIiwgc2NhbGUgPSAiY291bnQiKQ0KUE5wMyA9IFBOcDMgKyBzY2FsZV9maWxsX21hbnVhbChuYW1lID0gIkFwdGl0dWRlIiwgdmFsdWVzID0gYygiTEEiID0gImdyZXkiLCAiSEEiID0gIndoaXRlIiksIGxhYmVscyA9IGMoIkxBIiwgIkhBIikpDQpQTnAzID0gUE5wMyArIHNjYWxlX3hfZGlzY3JldGUobGFiZWxzID0gYygiTFYiLCAiSFYiLCAiSFZCIikpDQpQTnAzID0gUE5wMyArIGdlb21fZXJyb3JiYXIoYWVzKHltaW4gPSB0b25lX3Njb3JlLWNpLCB5bWF4ID0gdG9uZV9zY29yZStjaSksIHdpZHRoID0gLjQsIHBvc2l0aW9uID0gcG9zaXRpb25fZG9kZ2UoLjkpLCBkYXRhID0gZ3JhcGhkYXRhMmIpDQpQTnAzID0gUE5wMyArIHN0YXRfc3VtbWFyeShmdW4ueSA9IG1lYW4sIGdlb20gPSAicG9pbnQiLCBzaGFwZSA9IDIzLCBzaXplID0gMixwb3NpdGlvbiA9IHBvc2l0aW9uX2RvZGdlKC45KSkNClBOcDMgPSBQTnAzICsgdGhlbWVfYncoKSsgZ2d0aXRsZSgnUGljdHVyZSBOYW1pbmc6IFRvbmUgYWNjdXJhY3knKQ0KUE5wMyA9IFBOcDMgKyBsYWJzKHggPSAiVmFyaWFiaWxpdHkgQ29uZGl0aW9uIiwgeSA9ICJNZWFuIEFjY3VyYWN5IikNCnByaW50KFBOcDMpDQpgYGANCg0KIyMjIFN0YXRpc3RpY2FsIEFuYWx5c2lzDQoNCiMjIyMgTWFpbiBtb2RlbCAod2l0aG91dCBhcHRpdHVkZSBwcmVkaWN0b3JzOyBTZWN0aW9uIDMuNS4yKQ0KDQpgYGB7cn0NCk5QTjIgPSBsaXpDZW50ZXIoTlBOMiwgbGlzdCgiYXB0aXR1ZGVfcGNwdCIsICJzbG9wZSIpKQ0KTlBOMiA9IGxpekNvbnRyYXN0cyhOUE4yLCBOUE4yJGNvbmRpdGlvbiwgIkxWIikNCg0KTlBOX2dsbWVyID0gZ2xtZXIodG9uZV9zY29yZSB+IChMVl9WRVJTVVNfSFYgKyBMVl9WRVJTVVNfSFZCKSANCiAgICAgICAgICAgICAgKyAoMXxzdWJqZWN0KQ0KICAgICAgICAgICAgICAsIGZhbWlseSA9ICJiaW5vbWlhbCIsIA0KICAgICAgICAgICAgICBjb250cm9sID0gZ2xtZXJDb250cm9sKG9wdGltaXplciA9ICJib2J5cWEiKSwgDQogICAgICAgICAgICAgIGRhdGEgPSBOUE4yKQ0KYGBgDQoNCiMjIyMjIFByaW50IG91dCBlZmZlY3RzDQoNCmBgYHtyfQ0Ka2FibGUoc3VtbWFyeShOUE5fZ2xtZXIpJGNvZWZmaWNpZW50cywgZGlnaXRzID0gMykNCmBgYA0KDQojIyMjIEFuYWx5c2lzIHdpdGggUENQVCBhY2N1cmFjeSAoU2VjdGlvbiAzLjcpDQoNCmBgYHtyfQ0KDQpOUE5fZ2xtZXJfcGNwdCA9IGdsbWVyKHRvbmVfc2NvcmUgfiAoTFZfVkVSU1VTX0hWICsgTFZfVkVSU1VTX0hWQikgKiBhcHRpdHVkZV9wY3B0LmN0DQogICAgICAgICAgICAgICsgKDF8c3ViamVjdCkNCiAgICAgICAgICAgICAgLCBmYW1pbHkgPSAiYmlub21pYWwiLCANCiAgICAgICAgICAgICAgY29udHJvbCA9IGdsbWVyQ29udHJvbChvcHRpbWl6ZXIgPSAiYm9ieXFhIiksIA0KICAgICAgICAgICAgICBkYXRhID0gTlBOMikNCmBgYA0KDQojIyMjIyBDaGVjayB0aGF0IHdlIGdldCB0aGUgc2FtZSBwYXR0ZXJuIG9mIHJlc3VsdHMgZm9yIHRoZSBleHBlcmltZW50YWxseSBtYW5pcHVsYXRlZCB2YXJpYWJsZXMgYXMgaW4gdGhlIHByZXZpb3VzIG1vZGVsDQoNCmBgYHtyfQ0Ka2FibGUoZ2V0X2NvZWZmcyhOUE5fZ2xtZXJfcGNwdCwgYygiKEludGVyY2VwdCkiLCAiTFZfVkVSU1VTX0hWIiwgIkxWX1ZFUlNVU19IVkIiKSksIGRpZ2l0cyA9IDMpDQpgYGANCg0KIyMjIyMgUHJpbnQgb3V0IHRoZSBlZmZlY3Qgb2YgaW5kaXZpZHVhbCBhcHRpdHVkZSBhbmQga2V5IGludGVyYWN0aW9ucyB3aXRoIGFwdGl0dWRlDQoNCmBgYHtyfQ0Kc3VtbWFyeShOUE5fZ2xtZXJfcGNwdCkkY29lZmYNCmthYmxlKGdldF9jb2VmZnMoTlBOX2dsbWVyX3BjcHQsIGMoImFwdGl0dWRlX3BjcHQuY3QiLCAiTFZfVkVSU1VTX0hWOmFwdGl0dWRlX3BjcHQuY3QiLCAiTFZfVkVSU1VTX0hWQjphcHRpdHVkZV9wY3B0LmN0IikpLCBkaWdpdHMgPSAzKQ0KYGBgDQoNCiMjIyMgQW5hbHlzaXMgd2l0aCBDU1RDIHNsb3BlDQoNCmBgYHtyfQ0KTlBOX2dsbWVyX2NzdGMgPSBnbG1lcih0b25lX3Njb3JlIH4gKExWX1ZFUlNVU19IViArIExWX1ZFUlNVU19IVkIpICogc2xvcGUuY3QNCiAgICAgICAgICAgICAgKyAoMXxzdWJqZWN0KQ0KICAgICAgICAgICAgICAsIGZhbWlseSA9ICJiaW5vbWlhbCIsIA0KICAgICAgICAgICAgICBjb250cm9sID0gZ2xtZXJDb250cm9sKG9wdGltaXplciA9ICJib2J5cWEiKSwgDQogICAgICAgICAgICAgIGRhdGEgPSBOUE4yKQ0KYGBgDQoNCiMjIyMjIENoZWNrIHRoYXQgd2UgZ2V0IHRoZSBzYW1lIHBhdHRlcm4gb2YgcmVzdWx0cyBmb3IgdGhlIGV4cGVyaW1lbnRhbGx5IG1hbmlwdWxhdGVkIHZhcmlhYmxlcyBhcyBpbiB0aGUgcHJldmlvdXMgbW9kZWwNCg0KYGBge3J9DQprYWJsZShnZXRfY29lZmZzKE5QTl9nbG1lcl9jc3RjLCBjKCIoSW50ZXJjZXB0KSIsICJMVl9WRVJTVVNfSFYiLCAiTFZfVkVSU1VTX0hWQiIpKSwgZGlnaXRzID0gMykNCmBgYA0KDQojIyMjIyBQcmludCBvdXQgdGhlIGVmZmVjdCBvZiBpbmRpdmlkdWFsIGFwdGl0dWRlIGFuZCBrZXkgaW50ZXJhY3Rpb25zIHdpdGggYXB0aXR1ZGUNCg0KYGBge3J9DQprYWJsZShnZXRfY29lZmZzKE5QTl9nbG1lcl9jc3RjLCBjKCJzbG9wZS5jdCIsICJMVl9WRVJTVVNfSFY6c2xvcGUuY3QiLCAiTFZfVkVSU1VTX0hWQjpzbG9wZS5jdCIpKSwgZGlnaXRzID0gMykNCmBgYA0KDQojIyBQaWN0dXJlIE5hbWluZyAtIFBpbnlpbiBBY2N1cmFjeQ0KDQojIyMgRmlndXJlcw0KDQpgYGB7cn0NCm1lYW5zID0gd2l0aChOUE4yLCBhZ2dyZWdhdGUocGlueWluX3Njb3JlIH4gc3ViamVjdCArIGNvbmRpdGlvbiwgRlVOID0gbWVhbikpDQpJRG1lYW5zID0gd2l0aCAoTlBOMiwgYWdncmVnYXRlKGFwdGl0dWRlX3BjcHQgfiBzdWJqZWN0ICsgY29uZGl0aW9uLCBGVU4gPSBtZWFuKSkNCm1lYW5zID0gbWVyZ2UgKG1lYW5zLCBJRG1lYW5zKQ0KbWVhbnNiID0gd2l0aChOUE4yLCBhZ2dyZWdhdGUocGlueWluX3Njb3JlIH4gc3ViamVjdCArIGNvbmRpdGlvbiArIGFwdGl0dWRlMiwgRlVOID0gbWVhbikpDQptZWFuczJiID0gd2l0aChOUE4yLCBhZ2dyZWdhdGUocGlueWluX3Njb3JlIH4gc3ViamVjdCArIGNvbmRpdGlvbiArIGFwdGl0dWRlMywgRlVOID0gbWVhbikpDQoNCnhiPC0gc3VtbWFyeVNFKG1lYW5zYiwgbWVhc3VyZXZhciA9ICJwaW55aW5fc2NvcmUiLCBncm91cHZhcnMgPSBjKCJjb25kaXRpb24iKSwgbmEucm0gPSBGQUxTRSwgY29uZi5pbnRlcnZhbCA9IC45NSkNCmdyYXBoZGF0YWIgPC0gc3VtbWFyeVNFKG1lYW5zYiwgbWVhc3VyZXZhciA9ICJwaW55aW5fc2NvcmUiLCBncm91cHZhcnMgPSBjKCJjb25kaXRpb24iLCAiYXB0aXR1ZGUyIiksIG5hLnJtID0gRkFMU0UsIGNvbmYuaW50ZXJ2YWwgPSAuOTUpDQpncmFwaGRhdGEyYiA8LSBzdW1tYXJ5U0UobWVhbnMyYiwgbWVhc3VyZXZhciA9ICJwaW55aW5fc2NvcmUiLCBncm91cHZhcnMgPSBjKCJjb25kaXRpb24iLCAiYXB0aXR1ZGUzIiksIG5hLnJtID0gRkFMU0UsIGNvbmYuaW50ZXJ2YWwgPSAuOTUpDQpgYGANCg0KIyMjIyBNYWluIEdyYXBoIChGaWd1cmUgOSwgcmlnaHQpDQoNCmBgYHtyfQ0KUE5wNyA9IGdncGxvdCAobWVhbnMsIGFlcyh4ID0gY29uZGl0aW9uLCB5PXBpbnlpbl9zY29yZSkpIA0KUE5wNyA9IFBOcDcgKyBnZW9tX3Zpb2xpbihjb2xvdXIgPSAiYmxhY2siLCBzY2FsZSA9ICJjb3VudCIpDQpQTnA3ID0gUE5wNyArIHNjYWxlX3hfZGlzY3JldGUobGFiZWxzID0gYygiTFYiLCAiSFYiLCAiSFZCIikpDQpQTnA3ID0gUE5wNyArIGdlb21fZXJyb3JiYXIoYWVzKHltaW4gPSBwaW55aW5fc2NvcmUtY2ksIHltYXggPSBwaW55aW5fc2NvcmUrY2kpLCB3aWR0aCA9IC40LCBwb3NpdGlvbiA9IHBvc2l0aW9uX2RvZGdlKC45KSwgZGF0YSA9IHhiKQ0KUE5wNyA9IFBOcDcgKyBzdGF0X3N1bW1hcnkoZnVuLnkgPSBtZWFuLCBnZW9tID0gInBvaW50Iiwgc2hhcGUgPSAyMywgc2l6ZSA9IDIscG9zaXRpb24gPSBwb3NpdGlvbl9kb2RnZSguOSkpDQpQTnA3ID0gUE5wNyArIHRoZW1lX2J3KCkgKyBnZ3RpdGxlKCdQaWN0dXJlIE5hbWluZzogUGlueWluIEFjY3VyYWN5JykNClBOcDcgPSBQTnA3ICsgbGFicyh4ID0gIlZhcmlhYmlsaXR5IENvbmRpdGlvbiIsIHkgPSAiTWVhbiBBY2N1cmFjeSIpDQpwcmludChQTnA3KQ0KYGBgDQoNCiMjIyMgTWFpbiBHcmFwaCAoU2NhdHRlciBwbG90LCBGaWd1cmUgMTEsIE1pZGRsZSBwYW5lbCwgcmlnaHQpDQoNCmBgYHtyfQ0KUE5wMC4yID0gZ2dwbG90KG1lYW5zLCBhZXMoYXB0aXR1ZGVfcGNwdCwgcGlueWluX3Njb3JlLCBjb2xvdXIgPSBjb25kaXRpb24sIHNoYXBlID0gY29uZGl0aW9uKSkgDQpQTnAwLjIgPSBQTnAwLjIgKyBnZW9tX3BvaW50KGFlcyAoc2hhcGUgPSBjb25kaXRpb24pLCBzaXplID0gMikgKyBnZW9tX3Ntb290aChzZSA9IEZBTFNFLCBtZXRob2QgPSBsbSwgYWVzKGxpbmV0eXBlID0gY29uZGl0aW9uKSkNClBOcDAuMiA9IFBOcDAuMiArIHNjYWxlX2xpbmV0eXBlX2Rpc2NyZXRlKG5hbWUgPSAiVmFyaWFiaWxpdHkgQ29uZGl0aW9uIiwgYnJlYWtzID0gYygiTFYiLCJIViIsICJIVkIiKSwgbGFiZWxzID0gYygiTFYiLCAiSFYiLCAiSFZCIikpKyBzY2FsZV9zaGFwZV9kaXNjcmV0ZShuYW1lID0gIlZhcmlhYmlsaXR5IENvbmRpdGlvbiIsIGJyZWFrcyA9IGMoIkxWIiwiSFYiLCAiSFZCIiksIGxhYmVscyA9IGMoIkxWIiwgIkhWIiwgIkhWQiIpKSArIHNjYWxlX2NvbG91cl9ncmV5KG5hbWUgPSAiVmFyaWFiaWxpdHkgQ29uZGl0aW9uIiwgYnJlYWtzID0gYygiTFYiLCJIViIsICJIVkIiKSwgbGFiZWxzID0gYygiTFYiLCAiSFYiLCAiSFZCIikpDQpQTnAwLjIgPSBQTnAwLjIgKyBsYWJzKHggPSAiUENQVCBTY29yZSIsIHkgPSBOVUxMKSArICBnZ3RpdGxlKCdTY2F0dGVyIFBsb3QnKQ0KDQpQTnAwLjIubSA9IFBOcDAuMiArIHNjYWxlX2xpbmV0eXBlX2Rpc2NyZXRlKG5hbWUgPSAiICIsIGJyZWFrcyA9IGMoIkxWIiwgIkhWIiwgIkhWQiIpLCBsYWJlbHMgPSBjKCJMViIsICJIViIsICJIVkIiKSkgKyBzY2FsZV9zaGFwZV9kaXNjcmV0ZShuYW1lID0gIiAiLCBicmVha3MgPSBjKCJMViIsIkhWIiwgIkhWQiIpLCBsYWJlbHMgPSBjKCJMViIsICJIViIsICJIVkIiKSkgKyBzY2FsZV9jb2xvdXJfZ3JleShuYW1lID0gIiAiLCBicmVha3MgPSBjKCJMViIsIkhWIiwgIkhWQiIpLCBsYWJlbHMgPSBjKCJMViIsICJIViIsICJIVkIiKSkNClBOcDAuMi5tID0gUE5wMC4yLm0gKyBsYWJzKHggPSAiUENQVCBTY29yZSIsIHkgPSAiTWVhbiBBY2N1cmFjeSIpICsgZ2d0aXRsZSgnICcpDQoNCnByaW50KFBOcDAuMikNCmBgYA0KDQojIyMjIFBDUFQgR3JhcGggKEZpZ3VyZSAxMSwgTWlkZGxlIHBhbmVsLGxlZnQpDQoNCmBgYHtyfQ0KUE5wOCA9IGdncGxvdCAobWVhbnNiLCBhZXMoeCA9IGNvbmRpdGlvbix5ID0gcGlueWluX3Njb3JlKSkgDQpQTnA4ID0gUE5wOCArIGdlb21fdmlvbGluKGNvbG91ciA9ICJibGFjayIsIHNjYWxlID0gImNvdW50IikNClBOcDggPSBQTnA4ICsgZmFjZXRfd3JhcCh+YXB0aXR1ZGUyLCBuY29sID0gMikNClBOcDggPSBQTnA4ICsgc2NhbGVfeF9kaXNjcmV0ZShsYWJlbHMgPSBjKCJMViIsICJIViIsICJIVkIiKSkNClBOcDggPSBQTnA4ICsgZ2VvbV9lcnJvcmJhcihhZXMoeW1pbiA9IHBpbnlpbl9zY29yZS1jaSwgeW1heCA9IHBpbnlpbl9zY29yZStjaSksIHdpZHRoID0gLjQsIHBvc2l0aW9uID0gcG9zaXRpb25fZG9kZ2UoLjkpLCBkYXRhID0gZ3JhcGhkYXRhYikNClBOcDggPSBQTnA4ICsgc3RhdF9zdW1tYXJ5KGZ1bi55ID0gbWVhbiwgZ2VvbSA9ICJwb2ludCIsIHNoYXBlID0gMjMsIHNpemUgPSAyLHBvc2l0aW9uID0gcG9zaXRpb25fZG9kZ2UoLjkpKQ0KUE5wOCA9IFBOcDggK3RoZW1lX2J3KCkrIGdndGl0bGUoJ1BpY3R1cmUgTmFtaW5nOiBQaW55aW4gQWNjdXJhY3knKQ0KUE5wOCA9IFBOcDggKyBsYWJzKHggPSAiVmFyaWFiaWxpdHkgQ29uZGl0aW9uIiwgeSA9ICJNZWFuIEFjY3VyYWN5IikNCnByaW50KFBOcDgpDQpgYGANCg0KIyMjIyBDU1RDIEdyYXBoIChOb3QgdXNlZCkNCg0KYGBge3J9DQpQTnA5ID0gZ2dwbG90IChtZWFuczJiLCBhZXMoeCA9IGNvbmRpdGlvbiwgeSA9IHBpbnlpbl9zY29yZSwgZmlsbCA9IGFwdGl0dWRlMykpIA0KUE5wOSA9IFBOcDkgKyBnZW9tX3Zpb2xpbihjb2xvdXIgPSAiYmxhY2siLCBzY2FsZSA9ICJjb3VudCIpDQpQTnA5ID0gUE5wOSArIHNjYWxlX2ZpbGxfbWFudWFsKG5hbWUgPSAiYXB0aXR1ZGUzIiwgdmFsdWVzID0gYygiTEEiID0gImdyZXkiLCAiSEEiID0gIndoaXRlIiksIGxhYmVscyA9IGMoIkxBIiwgIkhBIikpDQpQTnA5ID0gUE5wOSArIHNjYWxlX3hfZGlzY3JldGUobGFiZWxzID0gYygiTFYiLCAiSFYiLCAiSFZCIikpDQpQTnA5ID0gUE5wOSArIGdlb21fZXJyb3JiYXIoYWVzKHltaW4gPSBwaW55aW5fc2NvcmUtY2ksIHltYXggPSBwaW55aW5fc2NvcmUrY2kpLCB3aWR0aCA9IC40LCBwb3NpdGlvbiA9IHBvc2l0aW9uX2RvZGdlKC45KSwgZGF0YSA9IGdyYXBoZGF0YTJiKQ0KUE5wOSA9IFBOcDkgKyBzdGF0X3N1bW1hcnkoZnVuLnkgPSBtZWFuLCBnZW9tID0gInBvaW50Iiwgc2hhcGUgPSAyMywgc2l6ZSA9IDIsIHBvc2l0aW9uID0gcG9zaXRpb25fZG9kZ2UoLjkpKQ0KUE5wOSA9IFBOcDkgKyB0aGVtZV9idygpKyBnZ3RpdGxlKCdQaWN0dXJlIE5hbWluZzogUGlueWluIEFjY3VyYWN5JykNClBOcDkgPSBQTnA5ICsgbGFicyh4ID0gIlZhcmlhYmlsaXR5IENvbmRpdGlvbiIsIHkgPSAiTWVhbiBBY2N1cmFjeSIpDQpwcmludChQTnA5KQ0KYGBgDQoNCiMjIyBTdGF0aXN0aWNhbCBBbmFseXNpcw0KDQojIyMjIE1haW4gTW9kZWwgKHdpdGhvdXQgaW5kaXZpZHVhbCBhcHRpdHVkZSBtZWFzdXJlczsgc2VjdGlvbiAzLjUuMikNCg0KYGBge3J9DQpOUE4yID0gbGl6Q2VudGVyKE5QTjIsIGxpc3QoImFwdGl0dWRlX3BjcHQiLCAic2xvcGUiKSkNCk5QTjIgPSBsaXpDb250cmFzdHMoTlBOMiwgTlBOMiRjb25kaXRpb24sICJMViIpDQoNCk5QTl9nbG1lciA9IGdsbWVyKHBpbnlpbl9zY29yZSB+IChMVl9WRVJTVVNfSFYgKyBMVl9WRVJTVVNfSFZCKSANCiAgICAgICAgICAgICAgKyAoMXxzdWJqZWN0KQ0KICAgICAgICAgICAgICAsIGZhbWlseSA9ICJiaW5vbWlhbCIsIA0KICAgICAgICAgICAgICBjb250cm9sID0gZ2xtZXJDb250cm9sKG9wdGltaXplciA9ICJib2J5cWEiKSwgDQogICAgICAgICAgICAgIGRhdGEgPSBOUE4yKQ0KYGBgDQoNCiMjIyMgUHJpbnQgb3V0IGtleSBlZmZlY3RzDQoNCmBgYHtyfQ0Ka2FibGUoc3VtbWFyeShOUE5fZ2xtZXIpJGNvZWZmaWNpZW50cywgZGlnaXRzID0gMykNCmBgYA0KDQojIyMjIEFuYWx5c2lzIHdpdGggUENQVCBhY2N1cmFjeSAoU2VjdGlvbiAzLjcpDQoNCmBgYHtyfQ0KTlBOX2dsbWVyX3BjcHQgPSBnbG1lcihwaW55aW5fc2NvcmUgfiAoTFZfVkVSU1VTX0hWICsgTFZfVkVSU1VTX0hWQikgKiBhcHRpdHVkZV9wY3B0LmN0DQogICAgICAgICAgICAgICsgKDF8c3ViamVjdCkNCiAgICAgICAgICAgICAgLCBmYW1pbHkgPSAiYmlub21pYWwiLCANCiAgICAgICAgICAgICAgY29udHJvbCA9IGdsbWVyQ29udHJvbChvcHRpbWl6ZXIgPSAiYm9ieXFhIiksIA0KICAgICAgICAgICAgICBkYXRhID0gTlBOMikNCmBgYA0KDQojIyMjIyBDaGVjayB0aGF0IHdlIGdldCB0aGUgc2FtZSBwYXR0ZXJuIG9mIHJlc3VsdHMgZm9yIHRoZSBleHBlcmltZW50YWxseSBtYW5pcHVsYXRlZCB2YXJpYWJsZXMgYXMgaW4gdGhlIHByZXZpb3VzIG1vZGVsDQoNCmBgYHtyfQ0Ka2FibGUoZ2V0X2NvZWZmcyhOUE5fZ2xtZXJfcGNwdCwgYygiKEludGVyY2VwdCkiLCJMVl9WRVJTVVNfSFYiLCAiTFZfVkVSU1VTX0hWQiIpKSwgZGlnaXRzID0gMykNCmBgYA0KDQojIyMjIyBQcmludCBvdXQgdGhlIGVmZmVjdCBvZiBpbmRpdmlkdWFsIGFwdGl0dWRlIGFuZCBrZXkgaW50ZXJhY3Rpb25zIHdpdGggYXB0aXR1ZGUNCg0KYGBge3J9DQpzdW1tYXJ5KE5QTl9nbG1lcl9wY3B0KSRjb2VmZg0Ka2FibGUoZ2V0X2NvZWZmcyhOUE5fZ2xtZXJfcGNwdCwgYygiYXB0aXR1ZGVfcGNwdC5jdCIsICJMVl9WRVJTVVNfSFY6YXB0aXR1ZGVfcGNwdC5jdCIsICJMVl9WRVJTVVNfSFZCOmFwdGl0dWRlX3BjcHQuY3QiKSksIGRpZ2l0cyA9IDMpDQpgYGANCg0KIyMjIyBBbmFseXNpcyB3aXRoIENTVEMgc2xvcGUNCg0KYGBge3J9DQpOUE5fZ2xtZXJfY3N0YyA9IGdsbWVyKHBpbnlpbl9zY29yZSB+IChMVl9WRVJTVVNfSFYgKyBMVl9WRVJTVVNfSFZCKSAqIHNsb3BlLmN0DQogICAgICAgICAgICAgICsgKDF8c3ViamVjdCkNCiAgICAgICAgICAgICAgLCBmYW1pbHkgPSAiYmlub21pYWwiLCANCiAgICAgICAgICAgICAgY29udHJvbCA9IGdsbWVyQ29udHJvbChvcHRpbWl6ZXIgPSAiYm9ieXFhIiksICANCiAgICAgICAgICAgICAgZGF0YSA9IE5QTjIpDQpgYGANCg0KIyMjIyMgQ2hlY2sgdGhhdCB3ZSBnZXQgdGhlIHNhbWUgcGF0dGVybiBvZiByZXN1bHRzIGZvciB0aGUgZXhwZXJpbWVudGFsbHkgbWFuaXB1bGF0ZWQgdmFyaWFibGVzIGFzIGluIHRoZSBwcmV2aW91cyBtb2RlbA0KDQpgYGB7cn0NCmthYmxlKGdldF9jb2VmZnMoTlBOX2dsbWVyX2NzdGMsIGMoIihJbnRlcmNlcHQpIiwiTFZfVkVSU1VTX0hWIiwgIkxWX1ZFUlNVU19IVkIiKSksIGRpZ2l0cyA9IDMpDQpgYGANCg0KIyMjIyMgUHJpbnQgb3V0IHRoZSBlZmZlY3Qgb2YgaW5kaXZpZHVhbCBhcHRpdHVkZSBhbmQga2V5IGludGVyYWN0aW9ucyB3aXRoIGFwdGl0dWRlDQoNCmBgYHtyfQ0Ka2FibGUoZ2V0X2NvZWZmcyhOUE5fZ2xtZXJfY3N0YywgYygic2xvcGUuY3QiLCAiTFZfVkVSU1VTX0hWOnNsb3BlLmN0IiwgIkxWX1ZFUlNVU19IVkI6c2xvcGUuY3QiKSksIGRpZ2l0cyA9IDMpDQpgYGA=
