# Supplementary material for: The effects of high versus low talker variability and individual aptitude on phonetic training of Mandarin lexical tones
Source: PeerJ. 2019 Aug 9;7:e7191. doi: 10.7717/peerj.7191 (PMC6690337; doi:10.7717/peerj.7191)
Supplement: Supplemental Information 5 [file peerj-07-7191-s005.html]

Bayesian analysis supplement script


Code 

- Show All Code
- Hide All Code
- Download Rmd

# Bayesian analysis supplement script

- Packages and Helper functions
  - Packages
  - Helper Functions
    - logodds
    - inverse\_logodds
    - myCenter
    - lizCenter
    - lizContrasts
    - get\_coeffs
    - Bf
    - Bf\_table
    - Bf\_range
    - addBF\_powercalc
    - addBf\_ranges
- Load and set up data sets
- Evidence for/ against hypothesis of greater generalization to novel voices/ in production after multiple voice training
  - Notes
  - Picture Identification
  - Picture Naming: tone accuracy
  - Picture Naming: pinyin accuracy
  - Word Repetition: tone accuracy
  - Word Repetition: pinyin accuracy
  - Three-interval Oddity Discrimination Task
  - compute bFs and ranges
- Evidence for/against hypothesis that participants with higher aptitude benefit more from HV training
  - Notes:
  - Picture Identification
  - Picture Naming
  - Word Repetition
  - 3 Interval oddity
  - training data
  - compute bFs and ranges
  - estimate sample increase

# Packages and Helper functions

## Packages


```
rm(list=ls())
suppressPackageStartupMessages(library(ggplot2))
suppressPackageStartupMessages(library(lmerTest))
suppressPackageStartupMessages(library(lme4))
suppressPackageStartupMessages(library(Matrix))
suppressPackageStartupMessages(library(gridExtra))
suppressPackageStartupMessages(library(plyr))
suppressPackageStartupMessages(library(knitr))
suppressPackageStartupMessages(library(irr))
suppressPackageStartupMessages(library(cowplot))
suppressPackageStartupMessages(theme_set(theme_bw()))
opts_chunk$set(fig.width=8, fig.height=5, echo=TRUE, warning=FALSE, message=FALSE, cache=TRUE)
suppressPackageStartupMessages(library("kableExtra"))
```

## Helper Functions

### logodds

This function takes a proportion and returns the log odds.


```
logodds <- function(p){log(p/(1-p))}
```

### inverse\_logodds

This function takes a log odds and returns the equivalent proportion.


```
inverse_logodds <- function(lo) {exp(lo)/(1+exp(lo)) }
```

### myCenter

This function outputs the centered values of a variable, which can be a numeric variable, a factor, or a data frame. It was taken from Florian Jaeger’s blog - https://hlplab.wordpress.com/2009/04/27/centering-several-variables/.

From his blog:

- If the input is a numeric variable, the output is the centered variable
- If the input is a factor, the output is a numeric variable with centered factor level values. That is, the factor’s levels are converted into numerical values in their inherent order (if not specified otherwise, R defaults to alphanumerical order). More specifically, this centers any binary factor so that the value below 0 will be the 1st level of the original factor, and the value above 0 will be the 2nd level.
- If the input is a data frame or matrix, the output is a new matrix of the same dimension and with the centered values and column names that correspond to the colnames() of the input preceded by “c” (e.g. “Variable1” will be “cVariable1”).


```
myCenter = function(x) {
  if (is.numeric(x)) { return(x - mean(x, na.rm = T)) }
    if (is.factor(x)) {
        x= as.numeric(x)
        return(x - mean(x, na.rm = T))
    }
    if (is.data.frame(x) || is.matrix(x)) {
        m= matrix(nrow = nrow(x), ncol = ncol(x))
        colnames(m)= paste("c", colnames(x), sep = "")
    
        for (i in 1:ncol(x)) {
        
            m[,i] = myCenter(x[,i])
        }
        return(as.data.frame(m))
    }
}
```

### lizCenter

This function provides a wrapper around myCenter allowing you to center a specific list of variables from a data frame.

- x: data frame
- listfname: a list of the variables to be centered (e.g. list(variable1, variable2))

The output is a copy of the data frame with a column (always a numeric variable) added for each of the centered variables. These columns are labelled with the each column’s previous name, but with “.ct” appended (e.g., “variable1” will become “variable1.ct”).


```
lizCenter = function(x, listfname) 
{
    for (i in 1:length(listfname)) 
    {
        fname = as.character(listfname[i])
        x[paste(fname,".ct", sep="")] = myCenter(x[fname])
    }
        
    return(x)
}
```

### lizContrasts

This function can be used to create two centered dummy variables which stand in place of a three-way factor (condition). This allows us to inspect each contrast separately, as well as their interactions with other factors. Other fixed effects in the model can be evaluated as the average effects across all levels of the factor.

The function takes a data frame (d), a factor from that database (condition), which must have three levels, and the name of the level of the factor which is to be used as the baseline for the contrasts (base level).

For example, if d is a data frame with a factor “condition” with three levels “(”lex\_skew“,”lex\_noskew“, and”mixed“) then lizContrasts(d, d$condition,”lex\_no\_skew“) returns a data frame with two (numeric) columns added labelled”lex\_noskew\_VERSUS\_lex\_mixed" and “lex\_noskew\_VERSUS\_lex\_skew”.

Wherever you would normally use “condition” in a formula in an LME, it can be replaced by (lex\_noskew\_VERSUS\_lex\_mixed + “lex\_noskew\_VERSUS\_lex\_skew) e.g. ~ (a \* condition) becomes ~ (a \* (lex\_noskew\_VERSUS\_lex\_mixed + lex\_noskew\_VERSUS\_lex\_skew)).


```
lizContrasts = function(d, condition, baselevel) 
{
  
    condition = factor(condition)
  condition = relevel(condition, baselevel)
    a = contrasts(condition)-apply(contrasts(condition),2,mean)
    d$dummy1[condition == rownames(a)[1]] <- a[1] 
    d$dummy1[condition == rownames(a)[2]] <- a[2] 
    d$dummy1[condition == rownames(a)[3]] <- a[3] 
    d$dummy2[condition == rownames(a)[1]] <- a[4] 
    d$dummy2[condition == rownames(a)[2]] <- a[5] 
    d$dummy2[condition == rownames(a)[3]] <- a[6] 
    name1 = paste(baselevel, rownames(a)[2],sep ="_VERSUS_")
    name2 = paste(baselevel, rownames(a)[3],sep ="_VERSUS_")
    d[name1] = d$dummy1 
    d[name2] = d$dummy2 
    d$dummy1 <-NULL 
    d$dummy2 <-NULL 
    
    return(d)
}
```

### get\_coeffs

This function allows us to inspect particular coefficients from the output of an LME model by putting them in a table.

- x: the output returned when running lmer or glmer (i.e. an object of type lmerMod or glmerMod)
- list: a list of the names of the coefficients to be extracted (e.g. c(“variable1”, “variable1:variable2”))


```
get_coeffs <- function(x,list){(as.data.frame(summary(x)$coefficients)[list,])}
```

### Bf

This function is equivalent to the Dienes (2008) calculator which can be found here: http://www.lifesci.sussex.ac.uk/home/Zoltan\_Dienes/inference/Bayes.htm.

The code was provided by Baguely and Kayne (2010) and can be found here: http://www.academia.edu/427288/Review\_of\_Understanding\_psychology\_as\_a\_science\_An\_introduction\_to\_scientific\_and\_statistical\_inference


```
Bf <- function(sd, obtained, uniform, lower = 0, upper = 1, meanoftheory = 0, sdtheory = 1, tail = 1){
  area <- 0
  if(identical(uniform, 1)){
    theta <- lower
    range <- upper - lower
    incr <- range / 2000
    for (A in -1000:1000){
      theta <- theta + incr
      dist_theta <- 1 / range
      height <- dist_theta * dnorm(obtained, theta, sd)
      area <- area + height * incr
    }
  }else
  {theta <- meanoftheory - 5 * sdtheory
  incr <- sdtheory / 200
  for (A in -1000:1000){
    theta <- theta + incr
    dist_theta <- dnorm(theta, meanoftheory, sdtheory)
    if(identical(tail, 1)){
      if (theta <= 0){
        dist_theta <- 0
      } else {
        dist_theta <- dist_theta * 2
      }
    }
    height <- dist_theta * dnorm(obtained, theta, sd)
    area <- area + height * incr
  }
  }
  LikelihoodTheory <- area
  Likelihoodnull <- dnorm(obtained, 0, sd)
  BayesFactor <- LikelihoodTheory / Likelihoodnull
  ret <- list("LikelihoodTheory" = LikelihoodTheory,"Likelihoodnull" = Likelihoodnull, "BayesFactor" = BayesFactor)
  ret
}
```

### Bf\_table

This works by calling the Bf function above on a dataframe which has the following columns: contrast std.Error estimate h1\_sd h1\_motivation (some text)

It computes the BF for each row using std.Error and estimate as the model of the data, and h1 as a half normal with mean = 0 and sd = h1\_sd and tails = tails.

The function allows for positive and negative h1 values. If sd\_theory is negative, since the BF function requires a positive value for sdtheory, both sdtheory and obtained are multiplied by -1.


```
#df
Bf_table<-function(df)
{
  Bfs = vector('double')
  estimates = vector('double')
  sterrors = vector('double')
  sdtheorys = vector('double')
  contrasts  = as.character(df$contrast)
  motivation = as.character(df$h1_motivation)
  
  df$tails = as.numeric(df$tails)
  for (i in 1:dim(df)[1]){
    #i=5
    sd_error = df$std.Error[i]
    obtained = df$estimate[i]
    stdtheory= df$h1_sd[i]
    tail = df$tails[i]
    
    if(df$h1_sd[i]<0) {
      stdtheory = h1_sd[i]*-1
      obtained = (df$estimate[i]*-1)
    }
    
    
    Bfs[i] = Bf( sd_error, obtained, uniform = 0, meanoftheory = 0, sdtheory=stdtheory , tail = tail)$BayesFactor
    estimates[i] = obtained
    sdtheorys[i] = stdtheory
    sterrors[i] = sd_error
  }
  
  df2 = data.frame(cbind(contrasts,  round(sterrors, 3), round(estimates,3), round(sdtheorys,3),    motivation, df$tails, round(Bfs,3)    )   )
  colnames(df2) = c("contrast", "std.Error", "estimate", "sdtheory",   "h1 motivation", "tail", "Bf" )
  
  return(df2)
  
  df2[i,]
  
}
```

### Bf\_range

This works with the Bf function above. It requires the obtained mean and SE for the current sample and works out what the BF would be for a range of predicted means (which are set to be sdtheoryrange (with meanoftheory=0).


```
Bf_range<-function(sd, obtained, meanoftheory=0, sdtheoryrange, tail=1)
{
  
  x = c(0)
  y = c(0)
  
  for(sdi in sdtheoryrange)
  {
    #sdi = sdtheoryrange[1]
    B = as.numeric(Bf(sd, obtained, meanoftheory=0, uniform = 0, sdtheory=sdi, tail)[3])
    
    #following line corrects for the fact that the calcuator does not correctly compute BF when sdtheory==0; this code ensures that if sdtheory ==0, BF=1
    
    if (sdi ==0 ) {B=1}
    
    x= append(x,sdi)  
    y= append(y,B)
    output = cbind(x,y)
    
  } 
  output = output[-1,] 
  colnames(output) = c("sdtheory", "BF")
  return(output) 
}
```

### addBF\_powercalc

This function takes as its input the output of BF table above (BF\_df), the number of participants in the current sample (N) and the maximum number of participants we would consider testing. It then works out what the minimal N would be to get a substantial BF, using the principle that the standard error is proportional to the square root of new-sample/ N (see Dienes video: https://www.youtube.com/watch?v=10Lsm\_o\_GRg “how many participants might I need”). It returns the table with an additional column saying how many participants would be needed to get substantial evidence for the null or H1, or that we still wouldn’t have substantial evidence either way even with the maximum number of participants.


```
addBf_powercalc <-function(Bf_df, N, max)
{
  Number = vector() 
  for (b in 1:dim(Bf_df)[1]){
    for(newN in N : max){
      
      BF=  as.numeric(Bf(sd=as.numeric(as.character(Bf_df$std.Error[b]))*sqrt(N/newN), obtained=as.numeric(as.character(Bf_df$estimate[b])), meanoftheory=0, uniform = 0, sdtheory=as.numeric(as.character(Bf_df$h1_sd[b])), tail=as.numeric(as.character(Bf_df$tail[b]))))[3]
      
      if(BF>=3) {
        Number[b] = paste ("evidence for H1 with", newN, "participants")
        break
      } else if (BF< (1/3)){
        Number[b] = paste ("evidence for H0 with", newN, "participants")
        break
      } else if(max== newN) { 
        Number[b] = paste ("evidence still ambiguous with", max, "participants")
      }
    }
        
  }
  df2 = cbind(Bf_df, Number)
  colnames(df2)[ncol(df2)]= "N needed"
  return (df2 )
}
```

### addBf\_ranges

This function takes as its input a dataframe which is the output of the BF\_table function. Each row provides the values which give the model of the data. It also requires a range of values to test as sd of h1. The function adds an additional column to this table which writes out the range of values within this range which give values which meet the criteria for: (i) strong evidence for null (BF < 1/10); substantial evidence for null (BF < 1/3); ambiguous (3 > BF > 1/3 ); substantial evidence for H1 (BF >= 10).


```
addBf_ranges <-function(Bf_df, sdtheoryrange)
{
  
  BFranges = vector()  
  for (b in 1:dim(Bf_df)[1]){
    #b=1
  range = Bf_range(sd=as.numeric(as.character(Bf_df$std.Error[b])), obtained=as.numeric(as.character(Bf_df$estimate[b])), meanoftheory=0, sdtheoryrange=sdtheoryrange, tail=as.numeric(as.character(Bf_df$tail[b])))
  
  from_table = vector()
  to_table = vector()
  cat = vector()
  category_table = vector()
  
  for(i in 1:dim(range)[1]) {       # go through each value in the range and categorize it
  
  #range = read.csv("range.csv")
  #i=1
  
  #categorize current BF 
  if (range[i,2] <= (1/10)) { 
  cat[i] = "strong_null"      ## NOT below or equal to 1/10
  } else if (range[i,2] <= (1/3)) {
  cat[i] = "subst_null" ## NOT below or equal to 1/10, IS below or equal to 1/3 
  } else if (range[i,2] < 3) { ## NOT below or equal to 1/10, NOT below or equal to 1/3, IS below 3 
  cat[i] = "ambiguous"
  } else if (range[i,2] >= 10) {## NOT below or equal to 1/10, NOT below or equal to 1/3, NOT below 3, IS above or equal to 10 
  cat[i] = "strong_h1"
  } else {                ## NOT below or equal to 1/10, NOT below or equal to 1/3, NOT below 3, NOT above or equal to 10 
  cat[i] = "subst_h1"
  }
  
  # adjust the table
  j = length(category_table) 
  
  if (i==1){                      # first one
  category_table[j+1] = cat[i]   
  from_table[j+1] = range[i,1]
  
  } else if (cat[i] != cat[i-1]) { # NOT the first one, IS one where need to start new range 
  to_table[j] = range[i-1,1]
  category_table[j+1] = cat[i]
  from_table[j+1] = range[i,1]
  } 
  
  if (i==dim(range)[1]){        # if its the last one, finish off the table
  to_table[j] = range[i,1]
  }
  }
  
  
  # go through the little table and turn it int a string of ranges  
  string = ""
  for(i in 1: length(category_table)){
  string = paste(string, category_table[i], ": From" , round(from_table[i],4) , "To" , round(to_table[i],4) , ";")
  }
  
  BFranges[b] = string
  }
  out = cbind(Bf_df, BFranges)
  return(out)
}
```

# Load and set up data sets


```
#setwd("datafiles")
#getwd()
pcpt <- read.csv("PCPT.csv")
dis <- read.csv("Discrimination.csv")
train <- read.csv("Training.csv")
PI <- read.csv("Picture Identification.csv")
PRO <- read.csv("Production_all.csv")
setwd("..")
```


```
The working directory was changed to C:/Users/liz/Dropbox/SharedFolders/mandarin_paper_liz_hanyu_helen_megan/PeerJ_Revision/key files inside a notebook chunk. The working directory will be reset when the chunk is finished running. Use the knitr root.dir option in the setup chunk to change the working directory for notebook chunks.
```


```
#Set up aptitude measure- i.e. pcpt pre-test scores 
pcpt$condition = factor(pcpt$condition, levels = c("0", "1", "2"), labels = c("LV", "HV", "HVB"))
pcpt$session = factor(pcpt$session, levels = c("1", "2"), labels = c("Pre-test", "Post-test"))
ID_pre = with(subset(pcpt, session == "Pre-test"), aggregate(accuracy ~ subject + session + condition, FUN = mean))
ID_pre$session = NULL
ID_pre$aptitude = ID_pre$accuracy *10
# Set up date for PI 
PI$condition = factor(PI$condition, levels = c("0", "1", "2"), labels = c("LV", "HV", "HVB"))
PI$voicetype = factor(PI$voicetype, levels = c("nv1", "tv1"), labels = c("New voice", "Trained voice"))
PI2 = merge(ID_pre, PI)
# setting up production data
PRO$session = factor(PRO$session, levels = c("pretest", "posttest", "nativespeaker", "picturenaming"), labels = c("Pre-test", "Post-test","Native-Speaker", "Picture-Naming"))
#Get rid of participant48's production data and other unidentifiable trials with quality issues. 
PRO = PRO[!(PRO$subject=="48"),]
PRO = PRO[PRO$tone != 0, ]
PC = subset(PRO, session == "Native-Speaker")
CWR = subset(PRO, session %in% c("Pre-test", "Post-test"))
CPN = subset(PRO, session == "Picture-Naming")
## Set up data for Word Repetition
NWR = CWR
NWR$condition = factor(NWR$condition, levels = c("0", "1","2"), labels = c("LV", "HV", "HVB"))
NWR$wordtype = factor(NWR$wordtype, levels = c("trained", "untrained"), labels = c("Trained", "Untrained"))
NWR2 = merge(NWR, ID_pre)
## Set up data for Picture Naming 
NPN = CPN
NPN$condition = factor(NPN$condition, levels = c("0", "1", "2"), labels = c("LV", "HV", "HVB"))
NPN2 = merge(ID_pre, NPN)
# Set up date for 3IO
dis$condition = factor(dis$condition, levels = c("0", "1", "2"), labels = c("LV", "HV", "HVB"))
dis$session = factor(dis$session, levels = c("1", "2"), labels = c("Pre-test", "Post-test"))
dis$trialtype = dis$voicetype = factor(dis$voicetype, levels = c("fff", "ffm", "fmf"), labels = c("Neutral", "Easy", "Hard"))
dis$wordtype = factor(dis$wordtype, levels = c("newword", "oldword"), labels = c("Untrained Item", "Trained Item"))
dis2 = merge(ID_pre, dis)
#Set up date for Training
train$condition = factor(train$condition, levels = c("0","2","1"), labels = c("LV", "HVB", "HV"))
train2 = merge(ID_pre, train)
```

# Evidence for/ against hypothesis of greater generalization to novel voices/ in production after multiple voice training

## Notes

Key goal is to look for evidence for the hypothesis that participants show better generalization to novel voices, or in production, after HV or HVB training than after LV training.

Points: 1) To have maximum data (maximize the evidence), we look for evidence for greater learning in both HV/HVB than in LV. To get this, we code a binary contrast LV versus HV+HVB (centered). For the post-tests (Picture ID/Picture Naming) we are interested in the evidence for a main effect of this contrast. For the pre-post tests, we are interested in the interaction between this contrast and session. 2) In PI test only untrained voice test items are relevant since we don’t predict better performance with HV for trained items. 3) In 3IO test and Word Repetition: we look at trained and untrained items combined (to maximize power and because it’s all generalization away from trained *voice*) 4) In the production measures - Word Rep and Picture Naming - we are interested in both tone and pinyin. The latter is relevant given the findings of Barcroft and Sommers that there is more generalization in production after exposure to multiple voices. However for Word Rep, since we didn’t see any overall improvement from pre- to post-, we don’t look at whether there is evidence that they improve more in one condition that the other. 6) Following Diennes 2008, we model H1 using a half normal with a mean of 0 and an sd set to be an estimate of H1. This biases smaller values. We take our estimates of H1 using estimates from elsewhere within the data which inform the likely size of the effect. The method for informing each one is explained below. We take BF < 1/3 to be substantial evidence for the null and BF > 3 to be substantial evidence for H1; values in between are ambiguous. In addition to computing the BF for a particular value of H1 for each contrast, we also compute a “robustness region” for each Bayes factor which shows the range of values of H1 over which we have ambiguous/substantial-evidenceH1/ substantial-evidenceH0.

## Picture Identification

Run lmer model with just novel voices data and factor condition2 levels LV versus HV+HVB


```
PI2$condition2 = PI2$condition
PI2$condition2[PI2$condition=="HVB"]= "HV"
PI2.NewVoice = subset (PI2, voicetype == "New voice")
PI2.NewVoice= lizCenter(PI2.NewVoice, list("voicetype", "aptitude", "condition2"))
p_glmer = glmer(score ~  condition2.ct
                + (1|subject)
                , family = "binomial", 
                control = glmerControl(optimizer = "bobyqa"), 
                data = PI2.NewVoice)
kable(round(summary(p_glmer)$coeff,3))
```


|  | Estimate | Std. Error | z value | Pr(>|z|) |
| --- | --- | --- | --- | --- |
| (Intercept) | 1.71 | 0.111 | 15.364 | 0.000 |
| condition2.ct | 0.13 | 0.228 | 0.570 | 0.569 |


Model of the data is the estimate and st.error for condition2.ct

Value to inform H1:

Work out a plausible maximum - and use half this as the estimate - using the intercept (grand mean).

- if “LV” is the logodds of the proportion correct in LV condition
- “HV” is the logodds of the proportion correct in HV condtion

Assumptions for plausible maximum:  
- minimal performance in LV - i.e. at chance (note chance here is logodds of 50%, i.e. 0) - above chance in HV, meaning that post-test drives all of the effect

thus: condition = (HV - chance) - (LV - chance) = HV - chance - 0 = HV - 0 = HV

intercept = (HV+LV))/2 = (HV+0))/2 = HV/2

2 \* intercept = HV condition = 2 \* intercept

since this is an estimate of the maximum value of condition, we halve it to get our estimate to use as the sd of the half normal:

sdtheory = intercept

values for Bayes factor calculation (computed below)


```
contrast = "PI, NovelVoice: LV versus HV"
std.Error = round(summary(p_glmer)$coeff["condition2.ct", "Std. Error" ],3)
estimate = round(summary(p_glmer)$coeff["condition2.ct", "Estimate" ],3)
h1_sd = round(summary(p_glmer)$coeff["(Intercept)", "Estimate" ],3)
tails = 1
PI_table = data.frame(cbind(contrast, std.Error, estimate, h1_sd,tails))  
kable(PI_table)
```


| contrast | std.Error | estimate | h1\_sd | tails |
| --- | --- | --- | --- | --- |
| PI, NovelVoice: LV versus HV | 0.228 | 0.13 | 1.71 | 1 |

## Picture Naming: tone accuracy

Run lmer model with factor condition2 levels LV versus HV+HVB


```
NPN2$condition2 = NPN2$condition
NPN2$condition2[NPN2$condition=="HVB"]= "HV"
NPN2 = lizCenter(NPN2, list("condition2"))
NPN_glmer = glmer(tone_score ~ condition2.ct
                  + (1|subject)
                  , family = "binomial", 
                  control = glmerControl(optimizer = "bobyqa"), 
                  data = NPN2)
kable(round(summary(NPN_glmer)$coeff,3))
```


|  | Estimate | Std. Error | z value | Pr(>|z|) |
| --- | --- | --- | --- | --- |
| (Intercept) | -0.023 | 0.080 | -0.284 | 0.776 |
| condition2.ct | -0.225 | 0.168 | -1.341 | 0.180 |


Model of the data is the estimate and st.error for condition2.ct Value to inform H1:

Work out a plausible maximum - and use half this as the estimate - using the intercept (grand mean).

if “LV” is the logodds of the proportion correct in LV condition and “HV” is the logodds of the proportion correct in HV condition

Assumptions for plausible maximum: - chance production of tones in LV condition - 1/4 (i.e. if they were at chance in producing each of four tones) in logodds space - above minimal performance in HV, meaning that HV drives all of the difference between the intercept and chance

thus: condition = (HV - chance) - (LV - chance) = HV - logodds(1/4) - 0 = HV - logodds(1/4)

intercept = (HV+LV)/2 2 *intercept = HV + LV = HV + logodds(1/4) HV = 2*  intercept - logodds(1/4)

subbing in for HV:

condition = (2 \* intercept - logodds(1/4)) - logodds(1/4) = 2\*(intercept - logodds(1/4))

since this is an estimate of the maximum value of condition, we halve it to get our estimate to use as the sd of the half normal:

sdtheory = intercept - logodds(1/4)


```
contrast = "PN: LV versus HV"
std.Error = round(summary(NPN_glmer)$coeff["condition2.ct", "Std. Error" ],3)
estimate = round(summary(NPN_glmer)$coeff["condition2.ct", "Estimate" ],3)
h1_sd = round(summary(NPN_glmer)$coeff["(Intercept)", "Estimate" ]- logodds(1/4),3)
tails = 1
PN_table = data.frame(cbind(contrast, std.Error, estimate, h1_sd,tails))  
kable(PN_table)
```


| contrast | std.Error | estimate | h1\_sd | tails |
| --- | --- | --- | --- | --- |
| PN: LV versus HV | 0.168 | -0.225 | 1.076 | 1 |

## Picture Naming: pinyin accuracy

Run lmer model with factor condition2 levels LV versus HV+HVB


```
NPN2$condition2 = NPN2$condition
NPN2$condition2[NPN2$condition=="HVB"]= "HV"
#table(NPN2$condition2, NPN2$condition)
NPN2 = lizCenter(NPN2, list("condition2"))
NPN_pinyin_glmer = glmer(pinyin_score ~ condition2.ct
                  + (1|subject)
                  , family = "binomial", 
                  control = glmerControl(optimizer = "bobyqa"), 
                  data = NPN2)
kable(round(summary(NPN_pinyin_glmer)$coeff,3))
```


|  | Estimate | Std. Error | z value | Pr(>|z|) |
| --- | --- | --- | --- | --- |
| (Intercept) | -0.212 | 0.093 | -2.289 | 0.022 |
| condition2.ct | 0.104 | 0.196 | 0.531 | 0.595 |


Model of the data is the estimate and st.error for condition2.ct Value to inform H1:

Work out a plausible maximum - and use half this as the estimate - using the intercept (grand mean).

if “LV” is the logodds of the proportion correct in LV condition and “HV” is the logodds of the proportion correct in HV condition

Assumptions for plausible maximum: - minimal performance in LV - this is actually 0% but we can’t compute this in logodds space. We therefore set this to be equivalent to one correct (i.e. 1/72) in logodds space - above minimal performance in HV, meaning that HV drives all of the difference between the intercept and the minimum

thus: condition = (HV - minimum) - (LV - minimum) = HV - logodds(1/72) - 0 = HV - logodds(1/72)

intercept = (HV+LV)/2 2 \* intercept = HV+LV = HV + logodds(1/72) HV = 2 \* intercept - logodds(1/72)

subbing in for HV:

condition = (2 \* intercept - logodds(1/72)) - logodds(1/72) = 2\*(intercept - logodds(1/72))

since this is an estimate of the maximum value of condition, we halve it to get our estimate to use as the sd of the half normal:

sdtheory = intercept - logodds(1/72)


```
contrast = "PN, pinyin: LV versus HV"
std.Error = round(summary(NPN_pinyin_glmer)$coeff["condition2.ct", "Std. Error" ],3)
estimate = round(summary(NPN_pinyin_glmer)$coeff["condition2.ct", "Estimate" ],3)
h1_sd = round(summary(NPN_pinyin_glmer)$coeff["(Intercept)", "Estimate" ]- logodds(1/72),3)
tails = 1
PN_pinyin_table = data.frame(cbind(contrast, std.Error, estimate, h1_sd,tails))  
kable(PN_pinyin_table)
```


| contrast | std.Error | estimate | h1\_sd | tails |
| --- | --- | --- | --- | --- |
| PN, pinyin: LV versus HV | 0.196 | 0.104 | 4.05 | 1 |

## Word Repetition: tone accuracy

Run lmer model with factor condition2 levels LV versus HV+HVB


```
NWR2$condition2 = NWR2$condition
NWR2$condition2[NWR2$condition == "HVB"]= "HV"
NWR2 = lizCenter(NWR2, list("wordtype","condition2"))
nw_glmer = glmer(tone_score ~ wordtype.ct * condition2.ct * session
              + (wordtype.ct * session||subject)
              , family="binomial", 
              control = glmerControl(optimizer = "bobyqa"), 
              data = NWR2)
kable(round(summary(nw_glmer)$coeff,3))
```


|  | Estimate | Std. Error | z value | Pr(>|z|) |
| --- | --- | --- | --- | --- |
| (Intercept) | 0.958 | 0.073 | 13.207 | 0.000 |
| wordtype.ct | -0.020 | 0.070 | -0.288 | 0.773 |
| condition2.ct | 0.060 | 0.153 | 0.394 | 0.693 |
| sessionPost-test | 0.395 | 0.075 | 5.291 | 0.000 |
| wordtype.ct:condition2.ct | 0.081 | 0.148 | 0.546 | 0.585 |
| wordtype.ct:sessionPost-test | 0.132 | 0.104 | 1.263 | 0.207 |
| condition2.ct:sessionPost-test | -0.108 | 0.157 | -0.689 | 0.491 |
| wordtype.ct:condition2.ct:sessionPost-test | -0.073 | 0.220 | -0.331 | 0.741 |


Model of the data is the estimate and st.error for condition2.ct:sessionPost-test (condition by session)

Value to inform H1:

Work out a plausible maximum - and use half this as the estimate - using the main effect of session (overall difference between pre and post) .

if “LV.POST” is the logodds of the proportion correct in LV condition at posttest “LV.PRE” is the logodds of the proportion correct in LV condition at pretest “HV.POST” is the logodds of the proportion correct in HV condition at posttest “HV.PRE” is the logodds of the proportion correct in HV condition at pretest

Assumptions for plausible maximum: - improvement from pre to post in HV - NO improvement from pre to post in LV i.e. LV.POST=LV.PRE, meaning all of the effect of session comes from the HV condition

thus: condition by session = (HV.POST - HV.PRE) - (LV.POST- LV.PRE) = (HV.POST - HV.PRE)

session  
= ((HV.POST - HV.PRE) + (LV.POST- LV.PRE))/2 = (HV.POST - HV.PRE)/2

2 \* session = (HV.POST - HV.PRE)

condition by session = 2 \* session

since this is an estimate of the maximum value of condition, we halve it to get our estimate to use as the sd of the half normal:

sdtheory = session  
(which is sessionPost-test in the above model)

Tone\_accuracy


```
contrast =  "Word Repetition, Tone score: LV versus HV by session"
std.Error = round(summary(nw_glmer)$coeff["condition2.ct:sessionPost-test", "Std. Error" ],3)
estimate = round(summary(nw_glmer)$coeff["condition2.ct:sessionPost-test", "Estimate" ],3)
h1_sd = round(summary(nw_glmer)$coeff["sessionPost-test", "Estimate" ],3)
tails = 1
wordrep_table = data.frame(cbind(contrast, std.Error, estimate, h1_sd,tails))  
kable(wordrep_table)
```


| contrast | std.Error | estimate | h1\_sd | tails |
| --- | --- | --- | --- | --- |
| Word Repetition, Tone score: LV versus HV by session | 0.157 | -0.108 | 0.395 | 1 |

## Word Repetition: pinyin accuracy

Run lmer model with factor condition2 levels LV versus HV+HVB


```
nw_glmer_pinyin = glmer(pinyin_score ~ wordtype.ct * condition2.ct * session
              + (wordtype.ct * session||subject)
              , family="binomial", 
              control = glmerControl(optimizer = "bobyqa"), 
              data = NWR2)
```


```
unable to evaluate scaled gradientModel failed to converge: degenerate  Hessian with 1 negative eigenvalues
```


```
kable(round(summary(nw_glmer_pinyin)$coeff,3))
```


|  | Estimate | Std. Error | z value | Pr(>|z|) |
| --- | --- | --- | --- | --- |
| (Intercept) | 0.181 | 0.047 | 3.871 | 0.000 |
| wordtype.ct | 0.204 | 0.066 | 3.067 | 0.002 |
| condition2.ct | -0.019 | 0.099 | -0.190 | 0.850 |
| sessionPost-test | 0.152 | 0.045 | 3.366 | 0.001 |
| wordtype.ct:condition2.ct | 0.069 | 0.140 | 0.494 | 0.621 |
| wordtype.ct:sessionPost-test | 0.055 | 0.090 | 0.614 | 0.539 |
| condition2.ct:sessionPost-test | -0.034 | 0.095 | -0.356 | 0.722 |
| wordtype.ct:condition2.ct:sessionPost-test | -0.013 | 0.189 | -0.070 | 0.944 |


Model of the data is the estimate and st.error for condition2.ct:sessionPost-test (condition by session)

Value to inform H1:

Work out a plausible maximum - and use half this as the estimate - using the main effect of session (overall difference between pre and post) .

if “LV.POST” is the logodds of the proportion correct in LV condition at posttest “LV.PRE” is the logodds of the proportion correct in LV condition at pretest “HV.POST” is the logodds of the proportion correct in HV condition at posttest “HV.PRE” is the logodds of the proportion correct in HV condition at pretest

Assumptions for plausible maximum: - improvement from pre to post in HV - NO improvement from pre to post in LV i.e. LV.POST=LV.PRE, meaning all of the effect of session comes from the HV condition

thus: condition by session = (HV.POST - HV.PRE) - (LV.POST- LV.PRE) = (HV.POST - HV.PRE)

session  
= ((HV.POST - HV.PRE) + (LV.POST- LV.PRE))/2 = (HV.POST - HV.PRE)/2

2 \* session = (HV.POST - HV.PRE)

condition by session = 2 \* session

since this is an estimate of the maximum value of condition, we halve it to get our estimate to use as the sd of the half normal:

sdtheory = session  
(which is sessionPost-test in the above model)


```
contrast =  "Word Repetition, Pinyin score: LV versus HV by session"
std.Error = round(summary(nw_glmer_pinyin)$coeff["condition2.ct:sessionPost-test", "Std. Error" ],3)
estimate = round(summary(nw_glmer_pinyin)$coeff["condition2.ct:sessionPost-test", "Estimate" ],3)
h1_sd = round(summary(nw_glmer_pinyin)$coeff["sessionPost-test", "Estimate" ],3)
tails = 1
wordrep_table_pinyin = data.frame(cbind(contrast, std.Error, estimate, h1_sd,tails))  
kable(wordrep_table_pinyin)
```


| contrast | std.Error | estimate | h1\_sd | tails |
| --- | --- | --- | --- | --- |
| Word Repetition, Pinyin score: LV versus HV by session | 0.095 | -0.034 | 0.152 | 1 |

## Three-interval Oddity Discrimination Task

Run lmer model with factor condition2 levels LV versus HV+HVB


```
dis2$condition2 = dis2$condition
dis2$condition2[dis2$condition == "HVB"]= "HV"
dis2 = lizCenter(dis2, list( "wordtype", "aptitude","condition2"))
dis2 = lizContrasts(dis2, dis2$condition, "LV")
dis2 = lizContrasts(dis2, dis2$trialtype, "Neutral")
d_glmer = glmer(score ~ session * wordtype.ct *  condition2.ct
                + (Neutral_VERSUS_Easy + Neutral_VERSUS_Hard): session
                + (Neutral_VERSUS_Easy + Neutral_VERSUS_Hard) 
                + (session * wordtype.ct||subject)
                , family = "binomial", 
                control = glmerControl(optimizer = "bobyqa"), 
                data = dis2)
kable(round(summary(d_glmer)$coeff,3))
```


|  | Estimate | Std. Error | z value | Pr(>|z|) |
| --- | --- | --- | --- | --- |
| (Intercept) | 0.396 | 0.059 | 6.711 | 0.000 |
| sessionPost-test | 0.310 | 0.048 | 6.523 | 0.000 |
| wordtype.ct | -0.314 | 0.064 | -4.951 | 0.000 |
| condition2.ct | 0.061 | 0.125 | 0.486 | 0.627 |
| Neutral\_VERSUS\_Easy | 0.400 | 0.079 | 5.091 | 0.000 |
| Neutral\_VERSUS\_Hard | -0.138 | 0.077 | -1.805 | 0.071 |
| sessionPost-test:wordtype.ct | 0.138 | 0.091 | 1.511 | 0.131 |
| sessionPost-test:condition2.ct | -0.001 | 0.100 | -0.014 | 0.989 |
| wordtype.ct:condition2.ct | -0.020 | 0.134 | -0.150 | 0.881 |
| sessionPost-test:Neutral\_VERSUS\_Easy | -0.270 | 0.113 | -2.395 | 0.017 |
| sessionPost-test:Neutral\_VERSUS\_Hard | 0.117 | 0.111 | 1.056 | 0.291 |
| sessionPost-test:wordtype.ct:condition2.ct | 0.010 | 0.193 | 0.050 | 0.960 |


Model of the data is the estimate and st.error for sessionPost-test:condition2.ct (condition by session)

Value to inform H1 (note: this is identical to wordrep above):

Work out a plausible maximum - and use half this as the estimate - using the main effect of session (overall difference between pre and post) .

if “LV.POST” is the logodds of the proportion correct in LV condition at posttest “LV.PRE” is the logodds of the proportion correct in LV condition at pretest “HV.POST” is the logodds of the proportion correct in HV condition at posttest “HV.PRE” is the logodds of the proportion correct in HV condition at pretest

Assumptions for plausible maximum: - improvement from pre to post in HV - NO improvement from pre to post in LV i.e. LV.POST=LV.PRE, meaning all of the effect of session comes from the HV condition

thus: condition by session = (HV.POST - HV.PRE) - (LV.POST- LV.PRE) = (HV.POST - HV.PRE)

session  
= ((HV.POST - HV.PRE) + (LV.POST- LV.PRE))/2 = (HV.POST - HV.PRE)/2

2 \* session = (HV.POST - HV.PRE)

condition by session = 2 \* session

since this is an estimate of the maximum value of condition, we halve it to get our estimate to use as the sd of the half normal:

sdtheory = session  
(which is sessionPost-test in the above model)


```
contrast = "3 Int. Odd, Tone score: LV by HV by session"
std.Error = round(summary(d_glmer)$coeff["sessionPost-test:condition2.ct", "Std. Error" ],3)
estimate = round(summary(d_glmer)$coeff["sessionPost-test:condition2.ct", "Estimate" ],3)
h1_sd = round(summary(d_glmer)$coeff["sessionPost-test", "Estimate" ],3)
tails = 1
discrim_table = data.frame(cbind(contrast, std.Error, estimate, h1_sd,tails))  
kable(discrim_table)
```


| contrast | std.Error | estimate | h1\_sd | tails |
| --- | --- | --- | --- | --- |
| 3 Int. Odd, Tone score: LV by HV by session | 0.1 | -0.001 | 0.31 | 1 |

## compute bFs and ranges


```
#df = rbind(wordrep_table,discrim_table, wordrep_ap_table, discrim_ap_table)
df= rbind(PI_table, PN_table, PN_pinyin_table, wordrep_table, wordrep_table_pinyin, discrim_table)
df$std.Error = as.numeric(as.character(df$std.Error))
df$estimate = as.numeric(as.character(df$estimate))
df$h1_sd = as.numeric(as.character(df$h1_sd))
df$tails = as.numeric(as.character(df$tails))
df$h1_motivation = ""
df2 = Bf_table(df)
#kable(df2)
#kable(addBf_ranges2(df2))
sdtheoryrange = seq( from = 0, to = 10 , length.out = 100)
addBf_ranges(df2,sdtheoryrange ) %>%
  kable() %>%
  kable_styling()
```


| contrast | std.Error | estimate | sdtheory | h1 motivation | tail | Bf | BFranges |
| --- | --- | --- | --- | --- | --- | --- | --- |
| PI, NovelVoice: LV versus HV | 0.228 | 0.13 | 1.71 |  | 1 | 0.219 | ambiguous : From 0 To 1.0101 ; subst\_null : From 1.1111 To 3.7374 ; strong\_null : From 3.8384 To 10 ; |
| PN: LV versus HV | 0.168 | -0.225 | 1.076 |  | 1 | 0.067 | ambiguous : From 0 To 0.101 ; subst\_null : From 0.202 To 0.7071 ; strong\_null : From 0.8081 To 10 ; |
| PN, pinyin: LV versus HV | 0.196 | 0.104 | 4.05 |  | 1 | 0.08 | ambiguous : From 0 To 0.9091 ; subst\_null : From 1.0101 To 3.0303 ; strong\_null : From 3.1313 To 10 ; |
| Word Repetition, Tone score: LV versus HV by session | 0.157 | -0.108 | 0.395 |  | 1 | 0.239 | ambiguous : From 0 To 0.202 ; subst\_null : From 0.303 To 0.9091 ; strong\_null : From 1.0101 To 10 ; |
| Word Repetition, Pinyin score: LV versus HV by session | 0.095 | -0.034 | 0.152 |  | 1 | 0.421 | ambiguous : From 0 To 0.202 ; subst\_null : From 0.303 To 0.7071 ; strong\_null : From 0.8081 To 10 ; |
| 3 Int. Odd, Tone score: LV by HV by session | 0.1 | -0.001 | 0.31 |  | 1 | 0.303 | ambiguous : From 0 To 0.202 ; subst\_null : From 0.303 To 0.9091 ; strong\_null : From 1.0101 To 10 ; |


We have substantial evidence for the null in each test apart from Word Rep pinyin. Note that robustness regions indicate that we would have also have obtained substantial BFs using smaller values than those we used. Note that for evidence for H0, the maximum scale factor is always infinity.

# Evidence for/against hypothesis that participants with higher aptitude benefit more from HV training

## Notes:

Points: 1) Unlike above, we also look at training data here. This is because in Sadakata & McQueen the interaction was found with trained items, so it isn’t just generalization trials that are relevant. 2) To have maximum data, for all of the tasks except training, we do what we did above and look for evidence for greater learning in both HV/HVB than in LV, i.e. we code a binary contrast LV versus HV+HVB (centered). For training, we saw in the previous analysis that HV and HVB are quite different, so here we look at LV v HV and LV v HVB separately (also note we have a lot more data points per participant for training). 3) For the post-tests (PI/PN) we are interested in the evidence for an interaction between the condition contrast and aptitude. For the pre-post tests, we are interested in the interaction between thecontrast, aptitude and session. For training we look at the evidence for an interaction between each condition contrast and aptitude. (NOTE: interaction with session would also be relevant but the model won’t converge with that in - even if we remove all of the slopes.) 4) For PI test, to have maximum power, we look at combination of trained and untrained voices (note: this is unlike what we did above, where we only looked at untrained voices. However, for the interaction with aptitude, one previous study found evidence with trained voices, and one with untrained, so we look across both). 5) In 3IO and Word Repetition test: we look at trained and untrained items combined. 6) In the production measures - WR and PN - we are only interested in tone since our aptitude measure is relevant for tone.

7. As above, following Dienes 2008, we model H1 using a half normal with a mean of 0 and an sd set to be an estimate of H1. This biases smaller values. We take our estimates of H1 using estimates from elsewhere within the data which inform the likely size of the effect. The method for informing each one is explained below. We take BF < 1/3 to be substnatial evidence for the null and BF > 3 to be substantial evidence for H1; values in between are ambiguous. In addition to computing the BF for a particular value of H1 for each contrast, we also compute a “robustness region” for each Bayes factor which shows the range of values of H1 over which we have ambiguous/substantial-evidenceH1/ substantial-evidenceH0)

## Picture Identification

Run lmer model factor condition2 levels LV versus HV+HVB


```
PI2$condition2 = PI2$condition
PI2$condition2[PI2$condition=="HVB"]= "HV"
PI2 = lizCenter(PI2, list("voicetype", "aptitude", "condition2"))
p_ap_glmer = glmer(score ~  condition2.ct *aptitude.ct
                + (1|subject)
                , family = "binomial", 
                control = glmerControl(optimizer = "bobyqa"), 
                data = PI2)
kable(round(summary(p_ap_glmer)$coeff,3))
```


|  | Estimate | Std. Error | z value | Pr(>|z|) |
| --- | --- | --- | --- | --- |
| (Intercept) | 2.094 | 0.116 | 18.087 | 0.000 |
| condition2.ct | -0.166 | 0.241 | -0.690 | 0.490 |
| aptitude.ct | 0.171 | 0.064 | 2.699 | 0.007 |
| condition2.ct:aptitude.ct | 0.006 | 0.127 | 0.044 | 0.965 |


Model of the data is the estimate and st.error for condition2.ct:aptitude.ct (condition by aptitude)

Value to inform H1:

Work out a plausible maximum - and use half this as the estimate - using the main effect of aptitude (= aptitude.ct - overall difference in performance on test for one step in aptitude (which is set to be 10% points on the pcpt test)).

if “LV.aptitude” is the effect of aptitude in the LV condition “HV.aptitude” is the effect of aptitude in the HV condition

Assumptions for plausible maximum: - positive value of aptitudein HV - NO effect of aptitude in LV (=0) [note: no reason to think we would get NEGATIVE effect of aptitude in either condition, based on previous data]

thus: condition by aptitude = HV.aptitude-LV.aptitude = HV.aptitude-0

aptitude = (HV.aptitude+LV.aptitude)/2 = (HV.aptitude)/2

2 \* aptitude = HV.aptitude-0

condition by aptitude = 2 \* aptitude

since this is an estimate of the maximum value of condition, we halve it to get our estimate to use as the sd of the half normal:

sdtheory = aptitude  
(which is aptitude.ct in the above model)


```
contrast = "PI_ap: LV versus HV by aptitude"
std.Error = round(summary(p_ap_glmer)$coeff["condition2.ct:aptitude.ct", "Std. Error" ],3)
estimate = round(summary(p_ap_glmer)$coeff["condition2.ct:aptitude.ct", "Estimate" ],3)
h1_sd = round(summary(p_ap_glmer)$coeff["aptitude.ct", "Estimate" ],3)
tails = 1
PI_ap_table = data.frame(cbind(contrast, std.Error, estimate, h1_sd,tails))  
kable(PI_ap_table)
```


| contrast | std.Error | estimate | h1\_sd | tails |
| --- | --- | --- | --- | --- |
| PI\_ap: LV versus HV by aptitude | 0.127 | 0.006 | 0.171 | 1 |

## Picture Naming

Run lmer model with factor condition2 levels LV versus HV+HVB


```
NPN2$condition2 = NPN2$condition
NPN2$condition2[NPN2$condition=="HVB"]= "HV"
NPN2 = lizCenter(NPN2, list("condition2","aptitude"))
NPN_ap_glmer = glmer(tone_score ~ condition2.ct*aptitude.ct
                  + (1|subject)
                  , family = "binomial", 
                  control = glmerControl(optimizer = "bobyqa"), 
                  data = NPN2)
kable(round(summary(NPN_ap_glmer)$coeff,3))
```


|  | Estimate | Std. Error | z value | Pr(>|z|) |
| --- | --- | --- | --- | --- |
| (Intercept) | -0.020 | 0.076 | -0.259 | 0.795 |
| condition2.ct | -0.201 | 0.161 | -1.252 | 0.211 |
| aptitude.ct | 0.099 | 0.042 | 2.350 | 0.019 |
| condition2.ct:aptitude.ct | 0.042 | 0.083 | 0.502 | 0.616 |


Model of the data is the estimate and st.error for condition2.ct:aptitude.ct (condition by aptitude)

Value to inform H1:

Work out a plausible maximum - and use half this as the estimate - using the main effect of aptitude (= aptitude.ct - overall difference in performance on test for one step in aptitude (which is set to be 10% points on the pcpt test)).

if “LV.aptitude” is the effect of aptitude in the LV condition “HV.aptitude” is the effect of aptitude in the HV condition

Assumptions for plausible maximum: - positive value of aptitude in HV - NO effect of aptitude in LV (=0) [note: no reason to think would get NEGATIVE effect of aptitude in either condition, based on previous data]

thus: condition by aptitude = HV.aptitude-LV.aptitude = HV.aptitude-0

aptitude = (HV.aptitude+LV.aptitude)/2 = (HV.aptitude)/2

2 \* aptitude = HV.aptitude-0

condition by aptitude = 2 \* aptitude

since this is an estimate of the maximum value of condition, we halve it to get our estimate to use as the sd of the half normal:

sdtheory = aptitude  
(which is aptitude.ct in the above model)


```
contrast = "PN_ap: LV versus HV by aptitude"
std.Error = round(summary(NPN_ap_glmer)$coeff["condition2.ct:aptitude.ct", "Std. Error" ],3)
estimate = round(summary(NPN_ap_glmer)$coeff["condition2.ct:aptitude.ct", "Estimate" ],3)
h1_sd = round(summary(NPN_ap_glmer)$coeff["aptitude.ct", "Estimate" ],3)
tails = 1
PN_ap_table = data.frame(cbind(contrast, std.Error, estimate, h1_sd,tails))  
kable(PN_ap_table)
```


| contrast | std.Error | estimate | h1\_sd | tails |
| --- | --- | --- | --- | --- |
| PN\_ap: LV versus HV by aptitude | 0.083 | 0.042 | 0.099 | 1 |

## Word Repetition

Run lmer model with factor condition2 levels LV versus HV+HVB. Also run second model which is used just to get overall value of aptitude at pre-test


```
NWR2$condition2 = NWR2$condition
NWR2$condition2[NWR2$condition == "HVB"]= "HV"
NWR2 = lizCenter(NWR2, list("session", "aptitude",  "wordtype","condition2"))
NWR2 = lizContrasts(NWR2, NWR2$condition, "LV")
nw_glmer_pcpt = glmer(tone_score ~ wordtype.ct * condition2.ct * session.ct 
              + aptitude.ct
              + aptitude.ct : session.ct
              + aptitude.ct : condition2.ct: session.ct 
              + aptitude.ct : condition2.ct: session.ct : wordtype.ct
              + (wordtype.ct * session.ct||subject)
              , family="binomial" , 
              control = glmerControl(optimizer = "bobyqa"), 
              data = NWR2)
kable(round(summary(nw_glmer_pcpt)$coeff,3))
```


|  | Estimate | Std. Error | z value | Pr(>|z|) |
| --- | --- | --- | --- | --- |
| (Intercept) | 1.157 | 0.054 | 21.389 | 0.000 |
| wordtype.ct | 0.046 | 0.052 | 0.880 | 0.379 |
| condition2.ct | 0.027 | 0.114 | 0.238 | 0.812 |
| session.ct | 0.415 | 0.075 | 5.562 | 0.000 |
| aptitude.ct | 0.073 | 0.029 | 2.469 | 0.014 |
| wordtype.ct:condition2.ct | 0.044 | 0.110 | 0.402 | 0.688 |
| wordtype.ct:session.ct | 0.129 | 0.104 | 1.234 | 0.217 |
| condition2.ct:session.ct | -0.102 | 0.157 | -0.652 | 0.514 |
| session.ct:aptitude.ct | 0.031 | 0.042 | 0.754 | 0.451 |
| wordtype.ct:condition2.ct:session.ct | -0.072 | 0.219 | -0.330 | 0.741 |
| condition2.ct:session.ct:aptitude.ct | 0.091 | 0.082 | 1.113 | 0.266 |
| wordtype.ct:condition2.ct:session.ct:aptitude.ct | -0.052 | 0.113 | -0.461 | 0.645 |


```
NWR3pre = subset(NWR2, session== "Pre-test")
NWR3pre = lizCenter(NWR3pre, list("session", "aptitude",  "wordtype","condition"))
NWR3pre = lizContrasts(NWR3pre, NWR3pre$condition, "LV")
nw_glmer_pcpt_PRE = glmer(tone_score ~ wordtype.ct * condition2.ct
              + aptitude.ct
                          + aptitude.ct :condition2.ct
              + aptitude.ct : condition2.ct : wordtype.ct
              + (wordtype.ct ||subject)
              , family="binomial" , 
              control = glmerControl(optimizer = "bobyqa"), 
              data = NWR3pre)
kable(round(summary(nw_glmer_pcpt_PRE)$coeff,3))
```


|  | Estimate | Std. Error | z value | Pr(>|z|) |
| --- | --- | --- | --- | --- |
| (Intercept) | 0.954 | 0.071 | 13.463 | 0.000 |
| wordtype.ct | -0.020 | 0.070 | -0.282 | 0.778 |
| condition2.ct | 0.074 | 0.150 | 0.493 | 0.622 |
| aptitude.ct | 0.059 | 0.039 | 1.498 | 0.134 |
| wordtype.ct:condition2.ct | 0.081 | 0.148 | 0.547 | 0.584 |
| condition2.ct:aptitude.ct | -0.031 | 0.078 | -0.395 | 0.693 |
| wordtype.ct:condition2.ct:aptitude.ct | 0.008 | 0.077 | 0.104 | 0.918 |


Model of the data is the estimate and st.error for session.ct:condition2.ct:aptitude.ct (session by aptitude by condition)

For our estimate of H1, need to work out a plausible maximum value of aptitude that we expect in any cell - we get this from the scale.

We do this as follows: maximum possible value of aptitude (max\_aptitude), working from the scale: Assume participant with minimal value of aptitude is at chance in producing one of the four tones (1/4 in log odds) and participant with maximum value of aptitude is at ceiling in producing the correct tone - i.e. 72/72 (since 72 test items) - but since we compute this in logodds use 71/72. Then to work out the change in tone-score for a 1-step change in aptitude we need to divide it by the length of the aptitude predictor (max value of aptitude for any participant - minimal value of aptitude for any participant).


```
length = max(NWR2$aptitude.ct) - min(NWR2$aptitude.ct)
length
```


```
[1] 6.5625
```


```
max_aptitude_wr = (logodds(71/72)- logodds(1/4))/length
max_aptitude_wr
```


```
[1] 0.8169588
```


We use this maximum value and the ACTUAL value of aptitude at pre-test to work out a maximum value for the interaction of interest, as follows:

“LV.PRE.aptitude” is the effect of aptitude at pre-test in LV “LV.POST.aptitude” is the effect of aptitude at post-test in LV “HV,PRE.aptitude” is the effect of aptitude at pre-test in HV “HV.POST.aptitude” is the effect of aptitude at post-test in HV

PRE.aptitude is the average value of aptitude at pre-test

max\_aptitude is maximum value of aptitude (as computed above)

Assumptions for plausible maximum: - both conditions have the same value of aptitude at pre-test (estimated as PRE.aptitude) - in LV condition, effect of aptitude is the same at pre and post test - in HV condition, effect of aptitude is increased to the maximum amount at post test (=max\_aptitude )

thus: session by aptitude by condition = (HV.POST.aptitude- HV.PRE.aptitude)-(LV.POST.aptitude- LV.PRE.aptitude) = (max\_aptitude- PRE.aptitude)-(PRE.aptitude- PRE.aptitude) = max\_aptitude- PRE.aptitude

since this is an estimate of the maximum value of condition, we halve it to get our estimate to use as the sd of the half normal:  
sdtheory = (max\_aptitude - PRE.aptidue)/2


```
contrast =   "Word Repetition, Tone score: aptitude by session by LV versus HV"
std.Error = round(summary(nw_glmer_pcpt)$coeff["condition2.ct:session.ct:aptitude.ct", "Std. Error"],3)
estimate = round(summary(nw_glmer_pcpt)$coeff["condition2.ct:session.ct:aptitude.ct","Estimate" ],3)
h1_sd =  round((max_aptitude_wr - summary(nw_glmer_pcpt_PRE)$coeff["aptitude.ct", "Estimate" ])/2,3)
              
tails = 1
WR_ap_table = data.frame(cbind(contrast, std.Error, estimate, h1_sd, tails))  
kable(WR_ap_table)
```


| contrast | std.Error | estimate | h1\_sd | tails |
| --- | --- | --- | --- | --- |
| Word Repetition, Tone score: aptitude by session by LV versus HV | 0.082 | 0.091 | 0.379 | 1 |

## 3 Interval oddity

Run factor condition2 levels LV versus HV+HVB


```
dis2$condition2= dis2$condition
dis2$condition2[dis2$condition=="HVB"]= "HV"
dis2 = lizCenter(dis2, list("session", "condition2"))
d_glmer_pcpt = glmer(score ~ session.ct * wordtype.ct * condition2.ct
                     + (Neutral_VERSUS_Easy + Neutral_VERSUS_Hard): session.ct 
                     + (Neutral_VERSUS_Easy + Neutral_VERSUS_Hard) 
                     + aptitude.ct
                     + aptitude.ct: session.ct
                     + aptitude.ct :condition2.ct : session.ct
                     + aptitude.ct : condition2.ct : session.ct : wordtype.ct
                     + (session.ct * wordtype.ct||subject)
                     , family = "binomial", 
                     control = glmerControl(optimizer = "bobyqa"), 
                     data = dis2)
kable(round(summary(d_glmer_pcpt)$coeff,3))
```


|  | Estimate | Std. Error | z value | Pr(>|z|) |
| --- | --- | --- | --- | --- |
| (Intercept) | 0.549 | 0.057 | 9.685 | 0.000 |
| session.ct | 0.302 | 0.046 | 6.590 | 0.000 |
| wordtype.ct | -0.246 | 0.046 | -5.375 | 0.000 |
| condition2.ct | 0.071 | 0.120 | 0.595 | 0.552 |
| Neutral\_VERSUS\_Easy | 0.266 | 0.056 | 4.715 | 0.000 |
| Neutral\_VERSUS\_Hard | -0.080 | 0.055 | -1.449 | 0.147 |
| aptitude.ct | 0.071 | 0.030 | 2.354 | 0.019 |
| session.ct:wordtype.ct | 0.141 | 0.091 | 1.546 | 0.122 |
| session.ct:condition2.ct | -0.005 | 0.097 | -0.050 | 0.960 |
| wordtype.ct:condition2.ct | -0.015 | 0.097 | -0.154 | 0.878 |
| session.ct:Neutral\_VERSUS\_Easy | -0.273 | 0.113 | -2.426 | 0.015 |
| session.ct:Neutral\_VERSUS\_Hard | 0.118 | 0.111 | 1.067 | 0.286 |
| session.ct:aptitude.ct | 0.007 | 0.025 | 0.292 | 0.770 |
| session.ct:wordtype.ct:condition2.ct | 0.009 | 0.193 | 0.045 | 0.964 |
| session.ct:condition2.ct:aptitude.ct | 0.048 | 0.050 | 0.969 | 0.333 |
| session.ct:wordtype.ct:condition2.ct:aptitude.ct | -0.003 | 0.099 | -0.034 | 0.973 |


```
dis3pre = subset(dis2, session == c("Pre-test"))
dis3pre = lizCenter(dis3pre, list("session", "condition2"))
d_glmer_pcpt_PRE = glmer(score ~wordtype.ct * condition2.ct
                     + (Neutral_VERSUS_Easy + Neutral_VERSUS_Hard) 
                     + aptitude.ct
                     + aptitude.ct : condition2.ct
                     + aptitude.ct : condition2.ct : wordtype.ct
                     + (wordtype.ct||subject)
                     , family = "binomial", 
                     control = glmerControl(optimizer = "bobyqa"), 
                     data = dis3pre)
kable(round(summary(d_glmer_pcpt_PRE)$coeff,3))
```


|  | Estimate | Std. Error | z value | Pr(>|z|) |
| --- | --- | --- | --- | --- |
| (Intercept) | 0.392 | 0.055 | 7.072 | 0.000 |
| wordtype.ct | -0.314 | 0.064 | -4.945 | 0.000 |
| condition2.ct | 0.069 | 0.117 | 0.591 | 0.554 |
| Neutral\_VERSUS\_Easy | 0.400 | 0.079 | 5.086 | 0.000 |
| Neutral\_VERSUS\_Hard | -0.138 | 0.077 | -1.807 | 0.071 |
| aptitude.ct | 0.066 | 0.030 | 2.213 | 0.027 |
| wordtype.ct:condition2.ct | -0.015 | 0.135 | -0.112 | 0.911 |
| condition2.ct:aptitude.ct | -0.054 | 0.060 | -0.898 | 0.369 |
| wordtype.ct:condition2.ct:aptitude.ct | 0.006 | 0.068 | 0.088 | 0.930 |


Model of the data is the estimate and st.error for session.ct:condition2.ct:aptitude.ct (session by aptitude by condition)

For our estimate of H1, need to work out a plausible maximum value of aptitude that we expect in any cell we get this from the scale.

We do this as follows: maximum possible value of aptitude (max\_aptitude), working from the scale: Assume participant with minimal value of aptitude is at chance in picking odd one out (1/3 in log odds) and participant with maximum value of aptitude is at ceiling in producing the correct tone - i.e. 72/72 (since 72 test items) - but since we compute this in logodds use 71/72. Then to work out the change in tone-score for a 1-step change in aptitude we need to divide it by the length of the aptitude predictor (max value of aptitude for any participant - minimal value of aptitude for any participant).


```
length = max(dis2$aptitude.ct) - min(dis2$aptitude.ct)
length
```


```
[1] 6.5625
```


```
max_aptitude_d = (logodds(71/72)- logodds(1/3))/length
max_aptitude_d
```


```
[1] 0.7551736
```


We use this maximum value and the ACTUAL value of aptitude at pre-test to work out a maximum value for the interaction of interest, as follows:

“LV.PRE.aptitude” is the effect of aptitude at pre-test in LV “LV.POST.aptitude” is the effect of aptitude at post-test in LV “HV,PRE.aptitude” is the effect of aptitude at pre-test in HV “HV.POST.aptitude” is the effect of aptitude at post-test in HV

PRE.aptitude is the average value of aptitude at pre-test

max\_aptitude is maximum value of aptitude (as computed above)

Assumptions for plausible maximum: - both conditions have the same value of aptitude at pre-test (estimated as PRE.aptitude) - in LV condition, effect of aptitude is the same at pre and post test - in HV condition, effect of aptitude is increased to the maximum amount at post test (=max\_aptitude )

thus: session by aptitude by condition = (HV.POST.aptitude- HV.PRE.aptitude)-(LV.POST.aptitude- LV.PRE.aptitude) = (max\_aptitude- PRE.aptitude)-(PRE.aptitude- PRE.aptitude) = max\_aptitude- PRE.aptitude

since this is an estimate of the maximum value of condition, we halve it to get our estimate to use as the sd of the half normal:  
sdtheory = (max\_aptitude - PRE.aptidue)/2


```
contrast =       "3 Int. Odd., Tone score: aptitude by session by LV versus HV"
std.Error = round(summary(d_glmer_pcpt)$coeff["session.ct:condition2.ct:aptitude.ct", "Std. Error" ],3)
estimate =  round(summary(d_glmer_pcpt)$coeff["session.ct:condition2.ct:aptitude.ct","Estimate" ],3)
h1_sd =  round((max_aptitude_d - summary(d_glmer_pcpt_PRE)$coeff["aptitude.ct", "Estimate" ])/2,3)
tails = 1
discrim_ap_table = data.frame(cbind(contrast, std.Error, estimate, h1_sd, tails))  
kable(discrim_ap_table)
```


| contrast | std.Error | estimate | h1\_sd | tails |
| --- | --- | --- | --- | --- |
| 3 Int. Odd., Tone score: aptitude by session by LV versus HV | 0.05 | 0.048 | 0.345 | 1 |

## training data


```
train2 = lizContrasts(train2, train2$condition, "LV")
train2 = lizCenter(train2, list("session","aptitude"))
train2.noHVB = lizCenter(subset(train2, condition != "HVB"), list("session", "condition", "aptitude"))
train2.noHV = lizCenter(subset(train2, condition != "HV"), list("session", "condition", "aptitude"))
t_glmer_pcpt = glmer(score ~ (LV_VERSUS_HV + LV_VERSUS_HVB) * aptitude.ct 
                + (1|subject)
              , family = "binomial", 
              control = glmerControl(optimizer = "bobyqa"), 
              data = train2)
kable(round(summary(t_glmer_pcpt )$coeff,3))
```


|  | Estimate | Std. Error | z value | Pr(>|z|) |
| --- | --- | --- | --- | --- |
| (Intercept) | 1.823 | 0.084 | 21.615 | 0.000 |
| LV\_VERSUS\_HV | -1.033 | 0.187 | -5.526 | 0.000 |
| LV\_VERSUS\_HVB | -0.506 | 0.196 | -2.582 | 0.010 |
| aptitude.ct | 0.129 | 0.048 | 2.686 | 0.007 |
| LV\_VERSUS\_HV:aptitude.ct | -0.037 | 0.119 | -0.309 | 0.757 |
| LV\_VERSUS\_HVB:aptitude.ct | 0.026 | 0.101 | 0.256 | 0.798 |


Model of the data is the estimate and st.error for condition2.ct:aptitude.ct (condition by aptitude)

Value to inform H1 (this is the same as for PI above):

Work out a plausible maximum - and use half this as the estimate - using the main effect of aptitude (= aptitude.ct - overall difference in performance on test for one step in aptitude (which is set to be 10% points on the pcpt test)).

if “LV.aptitude” is the effect of aptitude in the LV condition “HV.aptitude” is the effect of aptitude in the HV condition

Assumptions for plausible maximum: - positive value of aptitude in HV - NO effect of aptitude in LV (=0) [note: no reason to think would get NEGATIVE effect of aptitude in either condition, based on previous data]

thus: condition by aptitude = HV.aptitude-LV.aptitude = HV.aptitude-0

aptitude = (HV.aptitude+LV.aptitude)/2 = (HV.aptitude)/2

2 \* aptitude = HV.aptitude

condition by aptitude = 2 \* aptitude

since this is an estimate of the maximum value of condition, we halve it to get our estimate to use as the sd of the half normal:

sdtheory = aptitude  
(which is aptitude.ct in the above model)


```
contrast =   c("Training: aptitude by LV versus HV-unblocked",
               "Training: aptitude by LV versus HV-blocked")
std.Error = c(round(summary(t_glmer_pcpt)$coeff["LV_VERSUS_HV:aptitude.ct", "Std. Error"],3),
       round(summary(t_glmer_pcpt)$coeff["LV_VERSUS_HVB:aptitude.ct", "Std. Error"],3))      
              
estimate = c(round(summary(t_glmer_pcpt)$coeff["LV_VERSUS_HV:aptitude.ct", "Estimate"],3),
       round(summary(t_glmer_pcpt)$coeff["LV_VERSUS_HVB:aptitude.ct", "Estimate"],3))  
h1_sd =  round(rep(summary(t_glmer_pcpt)$coeff["aptitude.ct","Estimate" ],2),3)
              
tails = c(1,1)
train_ap_table = data.frame(cbind(contrast, std.Error, estimate, h1_sd, tails)) 
kable(train_ap_table)
```


| contrast | std.Error | estimate | h1\_sd | tails |
| --- | --- | --- | --- | --- |
| Training: aptitude by LV versus HV-unblocked | 0.119 | -0.037 | 0.129 | 1 |
| Training: aptitude by LV versus HV-blocked | 0.101 | 0.026 | 0.129 | 1 |

## compute bFs and ranges


| contrast | std.Error | estimate | sdtheory | h1 motivation | tail | Bf | BFranges |
| --- | --- | --- | --- | --- | --- | --- | --- |
| PI\_ap: LV versus HV by aptitude | 0.127 | 0.006 | 0.171 |  | 1 | 0.617 | ambiguous : From 0 To 0.3535 ; subst\_null : From 0.404 To 1.2626 ; strong\_null : From 1.3131 To 5 ; |
| PN\_ap: LV versus HV by aptitude | 0.083 | 0.042 | 0.099 |  | 1 | 0.904 | ambiguous : From 0 To 0.3535 ; subst\_null : From 0.404 To 1.2626 ; strong\_null : From 1.3131 To 5 ; |
| 3 Int. Odd., Tone score: aptitude by session by LV versus HV | 0.05 | 0.048 | 0.345 |  | 1 | 0.371 | ambiguous : From 0 To 0.3535 ; subst\_null : From 0.404 To 1.2626 ; strong\_null : From 1.3131 To 5 ; |
| Word Repetition, Tone score: aptitude by session by LV versus HV | 0.082 | 0.091 | 0.379 |  | 1 | 0.654 | ambiguous : From 0 To 0.7576 ; subst\_null : From 0.8081 To 2.5758 ; strong\_null : From 2.6263 To 5 ; |
| Training: aptitude by LV versus HV-unblocked | 0.119 | -0.037 | 0.129 |  | 1 | 0.572 | ambiguous : From 0 To 0.2525 ; subst\_null : From 0.303 To 0.9091 ; strong\_null : From 0.9596 To 5 ; |
| Training: aptitude by LV versus HV-blocked | 0.101 | 0.026 | 0.129 |  | 1 | 0.732 | ambiguous : From 0 To 0.3535 ; subst\_null : From 0.404 To 1.2626 ; strong\_null : From 1.3131 To 5 ; |

## estimate sample increase

Above we seen that we do not have substantial evidence for the null (or H1) with our current sample. We use a function to see how many participants we might need to see substanatial evidence for the null, assuming that the standard error decreases pro-portional to route N


```
# note lines5-6 of df are the training data where we are looking at contrasts not using the same sample, so the N is less (40 rather than 60)
addBf_powercalc(df[1:4,], 60, 500)%>%
  kable() %>%
  kable_styling()

addBf_powercalc(df[5:6,], 40, 500)%>%
  kable() %>%
  kable_styling()
```

LS0tDQp0aXRsZTogIkJheWVzaWFuIGFuYWx5c2lzIHN1cHBsZW1lbnQgc2NyaXB0Ig0Kb3V0cHV0Og0KICBodG1sX25vdGVib29rOg0KICAgIHRvYzogeWVzDQogICAgdG9jX2RlcHRoOiA0DQogIGh0bWxfZG9jdW1lbnQ6DQogICAgZGZfcHJpbnQ6IHBhZ2VkDQogICAgdG9jOiB5ZXMNCiAgICB0b2NfZGVwdGg6ICc0Jw0KLS0tDQoNCiMgUGFja2FnZXMgYW5kIEhlbHBlciBmdW5jdGlvbnMNCg0KIyMgUGFja2FnZXMNCg0KYGBge3J9DQpybShsaXN0PWxzKCkpDQoNCg0Kc3VwcHJlc3NQYWNrYWdlU3RhcnR1cE1lc3NhZ2VzKGxpYnJhcnkoZ2dwbG90MikpDQpzdXBwcmVzc1BhY2thZ2VTdGFydHVwTWVzc2FnZXMobGlicmFyeShsbWVyVGVzdCkpDQpzdXBwcmVzc1BhY2thZ2VTdGFydHVwTWVzc2FnZXMobGlicmFyeShsbWU0KSkNCnN1cHByZXNzUGFja2FnZVN0YXJ0dXBNZXNzYWdlcyhsaWJyYXJ5KE1hdHJpeCkpDQpzdXBwcmVzc1BhY2thZ2VTdGFydHVwTWVzc2FnZXMobGlicmFyeShncmlkRXh0cmEpKQ0Kc3VwcHJlc3NQYWNrYWdlU3RhcnR1cE1lc3NhZ2VzKGxpYnJhcnkocGx5cikpDQpzdXBwcmVzc1BhY2thZ2VTdGFydHVwTWVzc2FnZXMobGlicmFyeShrbml0cikpDQpzdXBwcmVzc1BhY2thZ2VTdGFydHVwTWVzc2FnZXMobGlicmFyeShpcnIpKQ0Kc3VwcHJlc3NQYWNrYWdlU3RhcnR1cE1lc3NhZ2VzKGxpYnJhcnkoY293cGxvdCkpDQpzdXBwcmVzc1BhY2thZ2VTdGFydHVwTWVzc2FnZXModGhlbWVfc2V0KHRoZW1lX2J3KCkpKQ0Kb3B0c19jaHVuayRzZXQoZmlnLndpZHRoPTgsIGZpZy5oZWlnaHQ9NSwgZWNobz1UUlVFLCB3YXJuaW5nPUZBTFNFLCBtZXNzYWdlPUZBTFNFLCBjYWNoZT1UUlVFKQ0KDQpzdXBwcmVzc1BhY2thZ2VTdGFydHVwTWVzc2FnZXMobGlicmFyeSgia2FibGVFeHRyYSIpKQ0KDQpgYGANCg0KIyMgSGVscGVyIEZ1bmN0aW9ucw0KIyMjIGxvZ29kZHMNCg0KVGhpcyBmdW5jdGlvbiB0YWtlcyBhIHByb3BvcnRpb24gYW5kIHJldHVybnMgdGhlIGxvZyBvZGRzLiANCg0KYGBge3J9DQpsb2dvZGRzIDwtIGZ1bmN0aW9uKHApe2xvZyhwLygxLXApKX0gIA0KYGBgDQoNCiMjIyBpbnZlcnNlX2xvZ29kZHMNCg0KVGhpcyBmdW5jdGlvbiB0YWtlcyBhIGxvZyBvZGRzIGFuZCByZXR1cm5zIHRoZSBlcXVpdmFsZW50IHByb3BvcnRpb24uDQpgYGB7cn0NCmludmVyc2VfbG9nb2RkcyA8LSBmdW5jdGlvbihsbykge2V4cChsbykvKDErZXhwKGxvKSkgfSAgDQpgYGANCg0KIyMjIG15Q2VudGVyDQoNClRoaXMgZnVuY3Rpb24gb3V0cHV0cyB0aGUgY2VudGVyZWQgdmFsdWVzIG9mIGEgdmFyaWFibGUsIHdoaWNoIGNhbiBiZSBhIG51bWVyaWMgdmFyaWFibGUsIGEgZmFjdG9yLCBvciBhIGRhdGEgZnJhbWUuIEl0IHdhcyB0YWtlbiBmcm9tIEZsb3JpYW4gSmFlZ2VyJ3MgYmxvZyAtIGh0dHBzOi8vaGxwbGFiLndvcmRwcmVzcy5jb20vMjAwOS8wNC8yNy9jZW50ZXJpbmctc2V2ZXJhbC12YXJpYWJsZXMvLg0KDQpGcm9tIGhpcyBibG9nOg0KDQotIElmIHRoZSBpbnB1dCBpcyBhIG51bWVyaWMgdmFyaWFibGUsIHRoZSBvdXRwdXQgaXMgdGhlIGNlbnRlcmVkIHZhcmlhYmxlDQoNCi0gSWYgdGhlIGlucHV0IGlzIGEgZmFjdG9yLCB0aGUgb3V0cHV0IGlzIGEgbnVtZXJpYyB2YXJpYWJsZSB3aXRoIGNlbnRlcmVkIGZhY3RvciBsZXZlbCB2YWx1ZXMuIFRoYXQgaXMsIHRoZSBmYWN0b3IncyBsZXZlbHMgYXJlIGNvbnZlcnRlZCBpbnRvIG51bWVyaWNhbCB2YWx1ZXMgaW4gdGhlaXIgaW5oZXJlbnQgb3JkZXIgKGlmIG5vdCBzcGVjaWZpZWQgb3RoZXJ3aXNlLCAgUiBkZWZhdWx0cyB0byBhbHBoYW51bWVyaWNhbCBvcmRlcikuIE1vcmUgc3BlY2lmaWNhbGx5LCB0aGlzIGNlbnRlcnMgYW55IGJpbmFyeSBmYWN0b3Igc28gdGhhdCB0aGUgdmFsdWUgYmVsb3cgMCB3aWxsIGJlIHRoZSAxc3QgbGV2ZWwgb2YgdGhlIG9yaWdpbmFsIGZhY3RvciwgYW5kIHRoZSB2YWx1ZSBhYm92ZSAwIHdpbGwgYmUgdGhlIDJuZCBsZXZlbC4NCg0KLSBJZiB0aGUgaW5wdXQgaXMgYSBkYXRhIGZyYW1lIG9yIG1hdHJpeCwgdGhlIG91dHB1dCBpcyBhIG5ldyBtYXRyaXggb2YgdGhlIHNhbWUgZGltZW5zaW9uIGFuZCB3aXRoIHRoZSBjZW50ZXJlZCB2YWx1ZXMgYW5kIGNvbHVtbiBuYW1lcyB0aGF0IGNvcnJlc3BvbmQgdG8gdGhlIGNvbG5hbWVzKCkgb2YgdGhlIGlucHV0IHByZWNlZGVkIGJ5ICJjIiAoZS5nLiAiVmFyaWFibGUxIiB3aWxsIGJlICJjVmFyaWFibGUxIikuDQoNCmBgYHtyfQ0KbXlDZW50ZXIgPSBmdW5jdGlvbih4KSB7DQogIGlmIChpcy5udW1lcmljKHgpKSB7IHJldHVybih4IC0gbWVhbih4LCBuYS5ybSA9IFQpKSB9DQoJaWYgKGlzLmZhY3Rvcih4KSkgew0KCQl4PSBhcy5udW1lcmljKHgpDQoJCXJldHVybih4IC0gbWVhbih4LCBuYS5ybSA9IFQpKQ0KCX0NCglpZiAoaXMuZGF0YS5mcmFtZSh4KSB8fCBpcy5tYXRyaXgoeCkpIHsNCgkJbT0gbWF0cml4KG5yb3cgPSBucm93KHgpLCBuY29sID0gbmNvbCh4KSkNCgkJY29sbmFtZXMobSk9IHBhc3RlKCJjIiwgY29sbmFtZXMoeCksIHNlcCA9ICIiKQ0KCQ0KCQlmb3IgKGkgaW4gMTpuY29sKHgpKSB7DQoJCQ0KCQkJbVssaV0gPSBteUNlbnRlcih4WyxpXSkNCgkJfQ0KCQlyZXR1cm4oYXMuZGF0YS5mcmFtZShtKSkNCgl9DQp9DQpgYGANCg0KIyMjIGxpekNlbnRlcg0KDQpUaGlzIGZ1bmN0aW9uIHByb3ZpZGVzIGEgd3JhcHBlciBhcm91bmQgbXlDZW50ZXIgYWxsb3dpbmcgeW91IHRvIGNlbnRlciBhIHNwZWNpZmljIGxpc3Qgb2YgdmFyaWFibGVzIGZyb20gYSBkYXRhIGZyYW1lLiANCg0KLSB4OiBkYXRhIGZyYW1lDQotIGxpc3RmbmFtZTogYSBsaXN0IG9mIHRoZSB2YXJpYWJsZXMgdG8gYmUgY2VudGVyZWQgKGUuZy4gbGlzdCh2YXJpYWJsZTEsIHZhcmlhYmxlMikpDQoNClRoZSBvdXRwdXQgaXMgYSBjb3B5IG9mIHRoZSBkYXRhIGZyYW1lIHdpdGggYSBjb2x1bW4gKGFsd2F5cyBhIG51bWVyaWMgdmFyaWFibGUpIGFkZGVkIGZvciBlYWNoIG9mIHRoZSBjZW50ZXJlZCB2YXJpYWJsZXMuIFRoZXNlIGNvbHVtbnMgYXJlIGxhYmVsbGVkIHdpdGggdGhlIGVhY2ggY29sdW1uJ3MgcHJldmlvdXMgbmFtZSwgYnV0IHdpdGggIi5jdCIgYXBwZW5kZWQgKGUuZy4sICJ2YXJpYWJsZTEiIHdpbGwgYmVjb21lICJ2YXJpYWJsZTEuY3QiKS4NCg0KDQpgYGB7cn0NCmxpekNlbnRlciA9IGZ1bmN0aW9uKHgsIGxpc3RmbmFtZSkgDQp7DQoJZm9yIChpIGluIDE6bGVuZ3RoKGxpc3RmbmFtZSkpIA0KCXsNCgkJZm5hbWUgPSBhcy5jaGFyYWN0ZXIobGlzdGZuYW1lW2ldKQ0KCQl4W3Bhc3RlKGZuYW1lLCIuY3QiLCBzZXA9IiIpXSA9IG15Q2VudGVyKHhbZm5hbWVdKQ0KCX0NCgkJDQoJcmV0dXJuKHgpDQp9DQpgYGAJDQoNCiMjIyBsaXpDb250cmFzdHMNCg0KVGhpcyBmdW5jdGlvbiBjYW4gYmUgdXNlZCB0byBjcmVhdGUgdHdvIGNlbnRlcmVkIGR1bW15IHZhcmlhYmxlcyB3aGljaCBzdGFuZCBpbiBwbGFjZSBvZiBhIHRocmVlLXdheSBmYWN0b3IgKGNvbmRpdGlvbikuIFRoaXMgYWxsb3dzIHVzIHRvIGluc3BlY3QgZWFjaCBjb250cmFzdCBzZXBhcmF0ZWx5LCBhcyB3ZWxsIGFzIHRoZWlyIGludGVyYWN0aW9ucyB3aXRoIG90aGVyIGZhY3RvcnMuIE90aGVyIGZpeGVkIGVmZmVjdHMgaW4gdGhlIG1vZGVsIGNhbiBiZSBldmFsdWF0ZWQgYXMgdGhlIGF2ZXJhZ2UgZWZmZWN0cyBhY3Jvc3MgYWxsIGxldmVscyBvZiB0aGUgZmFjdG9yLg0KDQpUaGUgZnVuY3Rpb24gdGFrZXMgYSBkYXRhIGZyYW1lIChkKSwgYSBmYWN0b3IgZnJvbSB0aGF0IGRhdGFiYXNlIChjb25kaXRpb24pLCB3aGljaCBtdXN0IGhhdmUgdGhyZWUgbGV2ZWxzLCBhbmQgdGhlIG5hbWUgb2YgdGhlIGxldmVsIG9mIHRoZSBmYWN0b3Igd2hpY2ggaXMgdG8gYmUgdXNlZCBhcyB0aGUgYmFzZWxpbmUgZm9yIHRoZSBjb250cmFzdHMgKGJhc2UgbGV2ZWwpLg0KDQpGb3IgZXhhbXBsZSwgaWYgZCBpcyBhIGRhdGEgZnJhbWUgd2l0aCBhIGZhY3RvciAiY29uZGl0aW9uIiB3aXRoIHRocmVlIGxldmVscyAiKCJsZXhfc2tldyIsICJsZXhfbm9za2V3IiwgYW5kICAibWl4ZWQiKSB0aGVuIGxpekNvbnRyYXN0cyhkLCBkJGNvbmRpdGlvbiwgImxleF9ub19za2V3IikgcmV0dXJucyBhIGRhdGEgZnJhbWUgd2l0aCB0d28gKG51bWVyaWMpIGNvbHVtbnMgYWRkZWQgbGFiZWxsZWQgImxleF9ub3NrZXdfVkVSU1VTX2xleF9taXhlZCIgYW5kICJsZXhfbm9za2V3X1ZFUlNVU19sZXhfc2tldyIuIA0KDQpXaGVyZXZlciB5b3Ugd291bGQgbm9ybWFsbHkgdXNlICJjb25kaXRpb24iIGluIGEgZm9ybXVsYSBpbiBhbiBMTUUsIGl0IGNhbiBiZSByZXBsYWNlZCBieSAobGV4X25vc2tld19WRVJTVVNfbGV4X21peGVkICsgImxleF9ub3NrZXdfVkVSU1VTX2xleF9za2V3KSBlLmcuIH4gKGEgKiBjb25kaXRpb24pIGJlY29tZXMgfiAoYSAqICAobGV4X25vc2tld19WRVJTVVNfbGV4X21peGVkICsgbGV4X25vc2tld19WRVJTVVNfbGV4X3NrZXcpKS4NCg0KYGBge3J9DQpsaXpDb250cmFzdHMgPSBmdW5jdGlvbihkLCBjb25kaXRpb24sIGJhc2VsZXZlbCkgDQp7DQogIA0KCWNvbmRpdGlvbiA9IGZhY3Rvcihjb25kaXRpb24pDQogIGNvbmRpdGlvbiA9IHJlbGV2ZWwoY29uZGl0aW9uLCBiYXNlbGV2ZWwpDQoNCglhID0gY29udHJhc3RzKGNvbmRpdGlvbiktYXBwbHkoY29udHJhc3RzKGNvbmRpdGlvbiksMixtZWFuKQ0KCWQkZHVtbXkxW2NvbmRpdGlvbiA9PSByb3duYW1lcyhhKVsxXV0gPC0gYVsxXSANCglkJGR1bW15MVtjb25kaXRpb24gPT0gcm93bmFtZXMoYSlbMl1dIDwtIGFbMl0gDQoJZCRkdW1teTFbY29uZGl0aW9uID09IHJvd25hbWVzKGEpWzNdXSA8LSBhWzNdIA0KCWQkZHVtbXkyW2NvbmRpdGlvbiA9PSByb3duYW1lcyhhKVsxXV0gPC0gYVs0XSANCglkJGR1bW15Mltjb25kaXRpb24gPT0gcm93bmFtZXMoYSlbMl1dIDwtIGFbNV0gDQoJZCRkdW1teTJbY29uZGl0aW9uID09IHJvd25hbWVzKGEpWzNdXSA8LSBhWzZdIA0KDQoJbmFtZTEgPSBwYXN0ZShiYXNlbGV2ZWwsIHJvd25hbWVzKGEpWzJdLHNlcCA9Il9WRVJTVVNfIikNCgluYW1lMiA9IHBhc3RlKGJhc2VsZXZlbCwgcm93bmFtZXMoYSlbM10sc2VwID0iX1ZFUlNVU18iKQ0KDQoJZFtuYW1lMV0gPSBkJGR1bW15MQkNCglkW25hbWUyXSA9IGQkZHVtbXkyCQ0KDQoJZCRkdW1teTEgPC1OVUxMCQ0KCWQkZHVtbXkyIDwtTlVMTAkNCgkNCglyZXR1cm4oZCkNCn0NCmBgYCAgDQoNCg0KIyMjIGdldF9jb2VmZnMNCg0KVGhpcyBmdW5jdGlvbiBhbGxvd3MgdXMgdG8gaW5zcGVjdCBwYXJ0aWN1bGFyIGNvZWZmaWNpZW50cyBmcm9tIHRoZSBvdXRwdXQgb2YgYW4gTE1FIG1vZGVsIGJ5IHB1dHRpbmcgdGhlbSBpbiBhIHRhYmxlLg0KDQotIHg6IHRoZSBvdXRwdXQgcmV0dXJuZWQgd2hlbiBydW5uaW5nIGxtZXIgb3IgZ2xtZXIgKGkuZS4gYW4gb2JqZWN0IG9mIHR5cGUgbG1lck1vZCBvciBnbG1lck1vZCkNCi0gbGlzdDogYSBsaXN0IG9mIHRoZSBuYW1lcyBvZiB0aGUgY29lZmZpY2llbnRzIHRvIGJlIGV4dHJhY3RlZCAoZS5nLiBjKCJ2YXJpYWJsZTEiLCAidmFyaWFibGUxOnZhcmlhYmxlMiIpKSANCg0KYGBge3J9DQpnZXRfY29lZmZzIDwtIGZ1bmN0aW9uKHgsbGlzdCl7KGFzLmRhdGEuZnJhbWUoc3VtbWFyeSh4KSRjb2VmZmljaWVudHMpW2xpc3QsXSl9DQpgYGAgDQoNCiMjIyBCZg0KDQpUaGlzIGZ1bmN0aW9uIGlzIGVxdWl2YWxlbnQgdG8gdGhlIERpZW5lcyAoMjAwOCkgY2FsY3VsYXRvciB3aGljaCBjYW4gYmUgZm91bmQgaGVyZTogaHR0cDovL3d3dy5saWZlc2NpLnN1c3NleC5hYy51ay9ob21lL1pvbHRhbl9EaWVuZXMvaW5mZXJlbmNlL0JheWVzLmh0bS4NCg0KVGhlIGNvZGUgd2FzIHByb3ZpZGVkIGJ5IEJhZ3VlbHkgYW5kIEtheW5lICgyMDEwKSBhbmQgY2FuIGJlIGZvdW5kIGhlcmU6IGh0dHA6Ly93d3cuYWNhZGVtaWEuZWR1LzQyNzI4OC9SZXZpZXdfb2ZfVW5kZXJzdGFuZGluZ19wc3ljaG9sb2d5X2FzX2Ffc2NpZW5jZV9Bbl9pbnRyb2R1Y3Rpb25fdG9fc2NpZW50aWZpY19hbmRfc3RhdGlzdGljYWxfaW5mZXJlbmNlDQoNCmBgYHtyfQ0KQmYgPC0gZnVuY3Rpb24oc2QsIG9idGFpbmVkLCB1bmlmb3JtLCBsb3dlciA9IDAsIHVwcGVyID0gMSwgbWVhbm9mdGhlb3J5ID0gMCwgc2R0aGVvcnkgPSAxLCB0YWlsID0gMSl7DQogIGFyZWEgPC0gMA0KICBpZihpZGVudGljYWwodW5pZm9ybSwgMSkpew0KICAgIHRoZXRhIDwtIGxvd2VyDQogICAgcmFuZ2UgPC0gdXBwZXIgLSBsb3dlcg0KICAgIGluY3IgPC0gcmFuZ2UgLyAyMDAwDQogICAgZm9yIChBIGluIC0xMDAwOjEwMDApew0KICAgICAgdGhldGEgPC0gdGhldGEgKyBpbmNyDQogICAgICBkaXN0X3RoZXRhIDwtIDEgLyByYW5nZQ0KICAgICAgaGVpZ2h0IDwtIGRpc3RfdGhldGEgKiBkbm9ybShvYnRhaW5lZCwgdGhldGEsIHNkKQ0KICAgICAgYXJlYSA8LSBhcmVhICsgaGVpZ2h0ICogaW5jcg0KICAgIH0NCiAgfWVsc2UNCiAge3RoZXRhIDwtIG1lYW5vZnRoZW9yeSAtIDUgKiBzZHRoZW9yeQ0KICBpbmNyIDwtIHNkdGhlb3J5IC8gMjAwDQogIGZvciAoQSBpbiAtMTAwMDoxMDAwKXsNCiAgICB0aGV0YSA8LSB0aGV0YSArIGluY3INCiAgICBkaXN0X3RoZXRhIDwtIGRub3JtKHRoZXRhLCBtZWFub2Z0aGVvcnksIHNkdGhlb3J5KQ0KICAgIGlmKGlkZW50aWNhbCh0YWlsLCAxKSl7DQogICAgICBpZiAodGhldGEgPD0gMCl7DQogICAgICAgIGRpc3RfdGhldGEgPC0gMA0KICAgICAgfSBlbHNlIHsNCiAgICAgICAgZGlzdF90aGV0YSA8LSBkaXN0X3RoZXRhICogMg0KICAgICAgfQ0KICAgIH0NCiAgICBoZWlnaHQgPC0gZGlzdF90aGV0YSAqIGRub3JtKG9idGFpbmVkLCB0aGV0YSwgc2QpDQogICAgYXJlYSA8LSBhcmVhICsgaGVpZ2h0ICogaW5jcg0KICB9DQogIH0NCiAgTGlrZWxpaG9vZFRoZW9yeSA8LSBhcmVhDQogIExpa2VsaWhvb2RudWxsIDwtIGRub3JtKG9idGFpbmVkLCAwLCBzZCkNCiAgQmF5ZXNGYWN0b3IgPC0gTGlrZWxpaG9vZFRoZW9yeSAvIExpa2VsaWhvb2RudWxsDQogIHJldCA8LSBsaXN0KCJMaWtlbGlob29kVGhlb3J5IiA9IExpa2VsaWhvb2RUaGVvcnksIkxpa2VsaWhvb2RudWxsIiA9IExpa2VsaWhvb2RudWxsLCAiQmF5ZXNGYWN0b3IiID0gQmF5ZXNGYWN0b3IpDQogIHJldA0KfSANCmBgYA0KDQoNCg0KIyMjIEJmX3RhYmxlDQoNClRoaXMgd29ya3MgYnkgY2FsbGluZyB0aGUgQmYgZnVuY3Rpb24gYWJvdmUgb24gYSBkYXRhZnJhbWUgd2hpY2ggaGFzIHRoZSBmb2xsb3dpbmcgY29sdW1uczoNCmNvbnRyYXN0DQpzdGQuRXJyb3INCmVzdGltYXRlDQpoMV9zZCANCmgxX21vdGl2YXRpb24gKHNvbWUgdGV4dCkNCg0KSXQgY29tcHV0ZXMgdGhlIEJGIGZvciBlYWNoIHJvdyB1c2luZyBzdGQuRXJyb3IgYW5kIGVzdGltYXRlIGFzIHRoZSBtb2RlbCBvZiB0aGUgZGF0YSwgYW5kIGgxIGFzIGEgaGFsZiBub3JtYWwgd2l0aCBtZWFuID0gMCBhbmQgc2QgPSBoMV9zZCBhbmQgdGFpbHMgPSB0YWlscy4NCg0KVGhlIGZ1bmN0aW9uIGFsbG93cyBmb3IgcG9zaXRpdmUgYW5kIG5lZ2F0aXZlIGgxIHZhbHVlcy4gSWYgc2RfdGhlb3J5IGlzIG5lZ2F0aXZlLCBzaW5jZSB0aGUgQkYgZnVuY3Rpb24gcmVxdWlyZXMgYSBwb3NpdGl2ZSB2YWx1ZSBmb3Igc2R0aGVvcnksIGJvdGggc2R0aGVvcnkgYW5kIG9idGFpbmVkIGFyZSBtdWx0aXBsaWVkIGJ5IC0xLg0KDQoNCmBgYHtyfQ0KI2RmDQpCZl90YWJsZTwtZnVuY3Rpb24oZGYpDQp7DQogIEJmcyA9IHZlY3RvcignZG91YmxlJykNCiAgZXN0aW1hdGVzID0gdmVjdG9yKCdkb3VibGUnKQ0KICBzdGVycm9ycyA9IHZlY3RvcignZG91YmxlJykNCiAgc2R0aGVvcnlzID0gdmVjdG9yKCdkb3VibGUnKQ0KICBjb250cmFzdHMgID0gYXMuY2hhcmFjdGVyKGRmJGNvbnRyYXN0KQ0KICBtb3RpdmF0aW9uID0gYXMuY2hhcmFjdGVyKGRmJGgxX21vdGl2YXRpb24pDQogIA0KICBkZiR0YWlscyA9IGFzLm51bWVyaWMoZGYkdGFpbHMpDQogIGZvciAoaSBpbiAxOmRpbShkZilbMV0pew0KICAgICNpPTUNCiAgICBzZF9lcnJvciA9IGRmJHN0ZC5FcnJvcltpXQ0KICAgIG9idGFpbmVkID0gZGYkZXN0aW1hdGVbaV0NCiAgICBzdGR0aGVvcnk9IGRmJGgxX3NkW2ldDQogICAgdGFpbCA9IGRmJHRhaWxzW2ldDQogICAgDQogICAgaWYoZGYkaDFfc2RbaV08MCkgew0KICAgICAgc3RkdGhlb3J5ID0gaDFfc2RbaV0qLTENCiAgICAgIG9idGFpbmVkID0gKGRmJGVzdGltYXRlW2ldKi0xKQ0KICAgIH0NCiAgICANCiAgICANCiAgICBCZnNbaV0gPSBCZiggc2RfZXJyb3IsIG9idGFpbmVkLCB1bmlmb3JtID0gMCwgbWVhbm9mdGhlb3J5ID0gMCwgc2R0aGVvcnk9c3RkdGhlb3J5ICwgdGFpbCA9IHRhaWwpJEJheWVzRmFjdG9yDQogICAgZXN0aW1hdGVzW2ldID0gb2J0YWluZWQNCiAgICBzZHRoZW9yeXNbaV0gPSBzdGR0aGVvcnkNCiAgICBzdGVycm9yc1tpXSA9IHNkX2Vycm9yDQogIH0NCiAgDQogIGRmMiA9IGRhdGEuZnJhbWUoY2JpbmQoY29udHJhc3RzLCAgcm91bmQoc3RlcnJvcnMsIDMpLCByb3VuZChlc3RpbWF0ZXMsMyksIHJvdW5kKHNkdGhlb3J5cywzKSwgICAgbW90aXZhdGlvbiwgZGYkdGFpbHMsIHJvdW5kKEJmcywzKSAgICApICAgKQ0KICBjb2xuYW1lcyhkZjIpID0gYygiY29udHJhc3QiLCAic3RkLkVycm9yIiwgImVzdGltYXRlIiwgInNkdGhlb3J5IiwgICAiaDEgbW90aXZhdGlvbiIsICJ0YWlsIiwgIkJmIiApDQogIA0KICByZXR1cm4oZGYyKQ0KICANCiAgZGYyW2ksXQ0KICANCn0NCmBgYA0KDQoNCg0KIyMjIEJmX3JhbmdlDQoNClRoaXMgd29ya3Mgd2l0aCB0aGUgQmYgZnVuY3Rpb24gYWJvdmUuIEl0IHJlcXVpcmVzIHRoZSBvYnRhaW5lZCBtZWFuIGFuZCBTRSBmb3IgdGhlIGN1cnJlbnQgc2FtcGxlIGFuZCB3b3JrcyBvdXQgd2hhdCB0aGUgQkYgd291bGQgYmUgZm9yIGEgcmFuZ2Ugb2YgcHJlZGljdGVkIG1lYW5zICh3aGljaCBhcmUgc2V0IHRvIGJlIHNkdGhlb3J5cmFuZ2UgKHdpdGggbWVhbm9mdGhlb3J5PTApLg0KYGBge3J9DQpCZl9yYW5nZTwtZnVuY3Rpb24oc2QsIG9idGFpbmVkLCBtZWFub2Z0aGVvcnk9MCwgc2R0aGVvcnlyYW5nZSwgdGFpbD0xKQ0Kew0KICANCiAgeCA9IGMoMCkNCiAgeSA9IGMoMCkNCiAgDQogIGZvcihzZGkgaW4gc2R0aGVvcnlyYW5nZSkNCiAgew0KICAgICNzZGkgPSBzZHRoZW9yeXJhbmdlWzFdDQogICAgQiA9IGFzLm51bWVyaWMoQmYoc2QsIG9idGFpbmVkLCBtZWFub2Z0aGVvcnk9MCwgdW5pZm9ybSA9IDAsIHNkdGhlb3J5PXNkaSwgdGFpbClbM10pDQogICAgDQogICAgI2ZvbGxvd2luZyBsaW5lIGNvcnJlY3RzIGZvciB0aGUgZmFjdCB0aGF0IHRoZSBjYWxjdWF0b3IgZG9lcyBub3QgY29ycmVjdGx5IGNvbXB1dGUgQkYgd2hlbiBzZHRoZW9yeT09MDsgdGhpcyBjb2RlIGVuc3VyZXMgdGhhdCBpZiBzZHRoZW9yeSA9PTAsIEJGPTENCiAgICANCiAgICBpZiAoc2RpID09MCApIHtCPTF9DQogICAgDQogICAgeD0gYXBwZW5kKHgsc2RpKSAgDQogICAgeT0gYXBwZW5kKHksQikNCiAgICBvdXRwdXQgPSBjYmluZCh4LHkpDQogICAgDQogIH0gDQogIG91dHB1dCA9IG91dHB1dFstMSxdIA0KICBjb2xuYW1lcyhvdXRwdXQpID0gYygic2R0aGVvcnkiLCAiQkYiKQ0KICByZXR1cm4ob3V0cHV0KSANCn0NCmBgYA0KDQoNCg0KIyMjIGFkZEJGX3Bvd2VyY2FsYw0KDQpUaGlzIGZ1bmN0aW9uIHRha2VzIGFzIGl0cyBpbnB1dCB0aGUgb3V0cHV0IG9mIEJGIHRhYmxlIGFib3ZlIChCRl9kZiksIHRoZSBudW1iZXIgb2YgcGFydGljaXBhbnRzIGluIHRoZSBjdXJyZW50IHNhbXBsZSAoTikgYW5kIHRoZSBtYXhpbXVtIG51bWJlciBvZiBwYXJ0aWNpcGFudHMgd2Ugd291bGQgY29uc2lkZXIgdGVzdGluZy4gSXQgdGhlbiB3b3JrcyBvdXQgd2hhdCB0aGUgbWluaW1hbCBOIHdvdWxkIGJlIHRvIGdldCBhIHN1YnN0YW50aWFsIEJGLCB1c2luZyB0aGUgcHJpbmNpcGxlIHRoYXQgdGhlIHN0YW5kYXJkIGVycm9yIGlzIHByb3BvcnRpb25hbCB0byB0aGUgc3F1YXJlIHJvb3Qgb2YgbmV3LXNhbXBsZS8gTiAoc2VlIERpZW5lcyB2aWRlbzogaHR0cHM6Ly93d3cueW91dHViZS5jb20vd2F0Y2g/dj0xMExzbV9vX0dSZyAiaG93IG1hbnkgcGFydGljaXBhbnRzIG1pZ2h0IEkgbmVlZCIpLiBJdCByZXR1cm5zIHRoZSB0YWJsZSB3aXRoIGFuIGFkZGl0aW9uYWwgY29sdW1uIHNheWluZyBob3cgbWFueSBwYXJ0aWNpcGFudHMgd291bGQgYmUgbmVlZGVkIHRvIGdldCBzdWJzdGFudGlhbCBldmlkZW5jZSBmb3IgdGhlIG51bGwgb3IgSDEsIG9yIHRoYXQgd2Ugc3RpbGwgd291bGRuJ3QgaGF2ZSBzdWJzdGFudGlhbCBldmlkZW5jZSBlaXRoZXIgd2F5IGV2ZW4gd2l0aCB0aGUgbWF4aW11bSBudW1iZXIgb2YgcGFydGljaXBhbnRzLg0KDQpgYGB7cn0NCmFkZEJmX3Bvd2VyY2FsYyA8LWZ1bmN0aW9uKEJmX2RmLCBOLCBtYXgpDQp7DQogIE51bWJlciA9IHZlY3RvcigpIA0KICBmb3IgKGIgaW4gMTpkaW0oQmZfZGYpWzFdKXsNCiAgICBmb3IobmV3TiBpbiBOIDogbWF4KXsNCiAgICAgIA0KICAgICAgQkY9ICBhcy5udW1lcmljKEJmKHNkPWFzLm51bWVyaWMoYXMuY2hhcmFjdGVyKEJmX2RmJHN0ZC5FcnJvcltiXSkpKnNxcnQoTi9uZXdOKSwgb2J0YWluZWQ9YXMubnVtZXJpYyhhcy5jaGFyYWN0ZXIoQmZfZGYkZXN0aW1hdGVbYl0pKSwgbWVhbm9mdGhlb3J5PTAsIHVuaWZvcm0gPSAwLCBzZHRoZW9yeT1hcy5udW1lcmljKGFzLmNoYXJhY3RlcihCZl9kZiRoMV9zZFtiXSkpLCB0YWlsPWFzLm51bWVyaWMoYXMuY2hhcmFjdGVyKEJmX2RmJHRhaWxbYl0pKSkpWzNdDQogICAgICANCiAgICAgIGlmKEJGPj0zKSB7DQogICAgICAgIE51bWJlcltiXSA9IHBhc3RlICgiZXZpZGVuY2UgZm9yIEgxIHdpdGgiLCBuZXdOLCAicGFydGljaXBhbnRzIikNCiAgICAgICAgYnJlYWsNCiAgICAgIH0gZWxzZSBpZiAoQkY8ICgxLzMpKXsNCiAgICAgICAgTnVtYmVyW2JdID0gcGFzdGUgKCJldmlkZW5jZSBmb3IgSDAgd2l0aCIsIG5ld04sICJwYXJ0aWNpcGFudHMiKQ0KICAgICAgICBicmVhaw0KICAgICAgfSBlbHNlIGlmKG1heD09IG5ld04pIHsgDQogICAgICAgIE51bWJlcltiXSA9IHBhc3RlICgiZXZpZGVuY2Ugc3RpbGwgYW1iaWd1b3VzIHdpdGgiLCBtYXgsICJwYXJ0aWNpcGFudHMiKQ0KICAgICAgfQ0KICAgIH0NCiAgICAgICAgDQogIH0NCiAgZGYyID0gY2JpbmQoQmZfZGYsIE51bWJlcikNCiAgY29sbmFtZXMoZGYyKVtuY29sKGRmMildPSAiTiBuZWVkZWQiDQogIHJldHVybiAoZGYyICkNCn0NCiAgICAgIA0KDQoNCmBgYA0KDQoNCg0KDQojIyMgYWRkQmZfcmFuZ2VzDQoNClRoaXMgZnVuY3Rpb24gdGFrZXMgYXMgaXRzIGlucHV0IGEgZGF0YWZyYW1lIHdoaWNoIGlzIHRoZSBvdXRwdXQgb2YgdGhlIEJGX3RhYmxlIGZ1bmN0aW9uLiBFYWNoIHJvdyBwcm92aWRlcyB0aGUgdmFsdWVzIHdoaWNoIGdpdmUgdGhlIG1vZGVsIG9mIHRoZSBkYXRhLiBJdCBhbHNvIHJlcXVpcmVzIGEgcmFuZ2Ugb2YgdmFsdWVzIHRvIHRlc3QgYXMgc2Qgb2YgaDEuIFRoZSBmdW5jdGlvbiBhZGRzIGFuIGFkZGl0aW9uYWwgY29sdW1uIHRvIHRoaXMgdGFibGUgd2hpY2ggd3JpdGVzIG91dCB0aGUgcmFuZ2Ugb2YgdmFsdWVzIHdpdGhpbiB0aGlzIHJhbmdlIHdoaWNoIGdpdmUgdmFsdWVzIHdoaWNoIG1lZXQgdGhlIGNyaXRlcmlhIGZvcjogKGkpIHN0cm9uZyBldmlkZW5jZSBmb3IgbnVsbCAoQkYgPCAxLzEwKTsgc3Vic3RhbnRpYWwgZXZpZGVuY2UgZm9yIG51bGwgKEJGIDwgMS8zKTsgYW1iaWd1b3VzICgzID4gQkYgPiAxLzMgKTsgc3Vic3RhbnRpYWwgZXZpZGVuY2UgZm9yIEgxIChCRiA+PSAxMCkuDQoNCg0KYGBge3J9DQphZGRCZl9yYW5nZXMgPC1mdW5jdGlvbihCZl9kZiwgc2R0aGVvcnlyYW5nZSkNCnsNCiAgDQogIEJGcmFuZ2VzID0gdmVjdG9yKCkgIA0KICBmb3IgKGIgaW4gMTpkaW0oQmZfZGYpWzFdKXsNCiAgICAjYj0xDQogIHJhbmdlID0gQmZfcmFuZ2Uoc2Q9YXMubnVtZXJpYyhhcy5jaGFyYWN0ZXIoQmZfZGYkc3RkLkVycm9yW2JdKSksIG9idGFpbmVkPWFzLm51bWVyaWMoYXMuY2hhcmFjdGVyKEJmX2RmJGVzdGltYXRlW2JdKSksIG1lYW5vZnRoZW9yeT0wLCBzZHRoZW9yeXJhbmdlPXNkdGhlb3J5cmFuZ2UsIHRhaWw9YXMubnVtZXJpYyhhcy5jaGFyYWN0ZXIoQmZfZGYkdGFpbFtiXSkpKQ0KICANCiAgZnJvbV90YWJsZSA9IHZlY3RvcigpDQogIHRvX3RhYmxlID0gdmVjdG9yKCkNCiAgY2F0ID0gdmVjdG9yKCkNCiAgY2F0ZWdvcnlfdGFibGUgPSB2ZWN0b3IoKQ0KICANCiAgZm9yKGkgaW4gMTpkaW0ocmFuZ2UpWzFdKSB7ICAgICAgICMgZ28gdGhyb3VnaCBlYWNoIHZhbHVlIGluIHRoZSByYW5nZSBhbmQgY2F0ZWdvcml6ZSBpdA0KICANCiAgI3JhbmdlID0gcmVhZC5jc3YoInJhbmdlLmNzdiIpDQogICNpPTENCiAgDQogICNjYXRlZ29yaXplIGN1cnJlbnQgQkYgDQogIGlmIChyYW5nZVtpLDJdIDw9ICgxLzEwKSkgeyANCiAgY2F0W2ldID0gInN0cm9uZ19udWxsIiAgICAgICMjIE5PVCBiZWxvdyBvciBlcXVhbCB0byAxLzEwDQogIH0gZWxzZSBpZiAocmFuZ2VbaSwyXSA8PSAoMS8zKSkgew0KICBjYXRbaV0gPSAic3Vic3RfbnVsbCIgIyMgTk9UIGJlbG93IG9yIGVxdWFsIHRvIDEvMTAsIElTIGJlbG93IG9yIGVxdWFsIHRvIDEvMyANCiAgfSBlbHNlIGlmIChyYW5nZVtpLDJdIDwgMykgeyAjIyBOT1QgYmVsb3cgb3IgZXF1YWwgdG8gMS8xMCwgTk9UIGJlbG93IG9yIGVxdWFsIHRvIDEvMywgSVMgYmVsb3cgMyANCiAgY2F0W2ldID0gImFtYmlndW91cyINCiAgfSBlbHNlIGlmIChyYW5nZVtpLDJdID49IDEwKSB7IyMgTk9UIGJlbG93IG9yIGVxdWFsIHRvIDEvMTAsIE5PVCBiZWxvdyBvciBlcXVhbCB0byAxLzMsIE5PVCBiZWxvdyAzLCBJUyBhYm92ZSBvciBlcXVhbCB0byAxMCANCiAgY2F0W2ldID0gInN0cm9uZ19oMSINCiAgfSBlbHNlIHsgICAgICAgICAgICAgICAgIyMgTk9UIGJlbG93IG9yIGVxdWFsIHRvIDEvMTAsIE5PVCBiZWxvdyBvciBlcXVhbCB0byAxLzMsIE5PVCBiZWxvdyAzLCBOT1QgYWJvdmUgb3IgZXF1YWwgdG8gMTAgDQogIGNhdFtpXSA9ICJzdWJzdF9oMSINCiAgfQ0KICANCiAgIyBhZGp1c3QgdGhlIHRhYmxlDQogIGogPSBsZW5ndGgoY2F0ZWdvcnlfdGFibGUpIA0KICANCiAgaWYgKGk9PTEpeyAgICAgICAgICAgICAgICAgICAgICAjIGZpcnN0IG9uZQ0KICBjYXRlZ29yeV90YWJsZVtqKzFdID0gY2F0W2ldICAgDQogIGZyb21fdGFibGVbaisxXSA9IHJhbmdlW2ksMV0NCiAgDQogIH0gZWxzZSBpZiAoY2F0W2ldICE9IGNhdFtpLTFdKSB7ICMgTk9UIHRoZSBmaXJzdCBvbmUsIElTIG9uZSB3aGVyZSBuZWVkIHRvIHN0YXJ0IG5ldyByYW5nZSANCiAgdG9fdGFibGVbal0gPSByYW5nZVtpLTEsMV0NCiAgY2F0ZWdvcnlfdGFibGVbaisxXSA9IGNhdFtpXQ0KICBmcm9tX3RhYmxlW2orMV0gPSByYW5nZVtpLDFdDQogIH0gDQogIA0KICBpZiAoaT09ZGltKHJhbmdlKVsxXSl7ICAgICAgICAjIGlmIGl0cyB0aGUgbGFzdCBvbmUsIGZpbmlzaCBvZmYgdGhlIHRhYmxlDQogIHRvX3RhYmxlW2pdID0gcmFuZ2VbaSwxXQ0KICB9DQogIH0NCiAgDQogIA0KICAjIGdvIHRocm91Z2ggdGhlIGxpdHRsZSB0YWJsZSBhbmQgdHVybiBpdCBpbnQgYSBzdHJpbmcgb2YgcmFuZ2VzICANCiAgc3RyaW5nID0gIiINCiAgZm9yKGkgaW4gMTogbGVuZ3RoKGNhdGVnb3J5X3RhYmxlKSl7DQogIHN0cmluZyA9IHBhc3RlKHN0cmluZywgY2F0ZWdvcnlfdGFibGVbaV0sICI6IEZyb20iICwgcm91bmQoZnJvbV90YWJsZVtpXSw0KSAsICJUbyIgLCByb3VuZCh0b190YWJsZVtpXSw0KSAsICI7IikNCiAgfQ0KICANCiAgQkZyYW5nZXNbYl0gPSBzdHJpbmcNCiAgfQ0KICBvdXQgPSBjYmluZChCZl9kZiwgQkZyYW5nZXMpDQogIHJldHVybihvdXQpDQp9DQoNCg0KYGBgDQoNCg0KDQoNCg0KDQoNCg0KIyBMb2FkIGFuZCBzZXQgdXAgZGF0YSBzZXRzDQoNCmBgYHtyfQ0KI3NldHdkKCJkYXRhZmlsZXMiKQ0KI2dldHdkKCkNCnBjcHQgPC0gcmVhZC5jc3YoIlBDUFQuY3N2IikNCmRpcyA8LSByZWFkLmNzdigiRGlzY3JpbWluYXRpb24uY3N2IikNCnRyYWluIDwtIHJlYWQuY3N2KCJUcmFpbmluZy5jc3YiKQ0KUEkgPC0gcmVhZC5jc3YoIlBpY3R1cmUgSWRlbnRpZmljYXRpb24uY3N2IikNClBSTyA8LSByZWFkLmNzdigiUHJvZHVjdGlvbl9hbGwuY3N2IikNCnNldHdkKCIuLiIpDQoNCiNTZXQgdXAgYXB0aXR1ZGUgbWVhc3VyZS0gaS5lLiBwY3B0IHByZS10ZXN0IHNjb3JlcyANCnBjcHQkY29uZGl0aW9uID0gZmFjdG9yKHBjcHQkY29uZGl0aW9uLCBsZXZlbHMgPSBjKCIwIiwgIjEiLCAiMiIpLCBsYWJlbHMgPSBjKCJMViIsICJIViIsICJIVkIiKSkNCnBjcHQkc2Vzc2lvbiA9IGZhY3RvcihwY3B0JHNlc3Npb24sIGxldmVscyA9IGMoIjEiLCAiMiIpLCBsYWJlbHMgPSBjKCJQcmUtdGVzdCIsICJQb3N0LXRlc3QiKSkNCklEX3ByZSA9IHdpdGgoc3Vic2V0KHBjcHQsIHNlc3Npb24gPT0gIlByZS10ZXN0IiksIGFnZ3JlZ2F0ZShhY2N1cmFjeSB+IHN1YmplY3QgKyBzZXNzaW9uICsgY29uZGl0aW9uLCBGVU4gPSBtZWFuKSkNCklEX3ByZSRzZXNzaW9uID0gTlVMTA0KSURfcHJlJGFwdGl0dWRlID0gSURfcHJlJGFjY3VyYWN5ICoxMA0KDQoNCg0KIyBTZXQgdXAgZGF0ZSBmb3IgUEkgDQpQSSRjb25kaXRpb24gPSBmYWN0b3IoUEkkY29uZGl0aW9uLCBsZXZlbHMgPSBjKCIwIiwgIjEiLCAiMiIpLCBsYWJlbHMgPSBjKCJMViIsICJIViIsICJIVkIiKSkNClBJJHZvaWNldHlwZSA9IGZhY3RvcihQSSR2b2ljZXR5cGUsIGxldmVscyA9IGMoIm52MSIsICJ0djEiKSwgbGFiZWxzID0gYygiTmV3IHZvaWNlIiwgIlRyYWluZWQgdm9pY2UiKSkNClBJMiA9IG1lcmdlKElEX3ByZSwgUEkpDQoNCiMgc2V0dGluZyB1cCBwcm9kdWN0aW9uIGRhdGENClBSTyRzZXNzaW9uID0gZmFjdG9yKFBSTyRzZXNzaW9uLCBsZXZlbHMgPSBjKCJwcmV0ZXN0IiwgInBvc3R0ZXN0IiwgIm5hdGl2ZXNwZWFrZXIiLCAicGljdHVyZW5hbWluZyIpLCBsYWJlbHMgPSBjKCJQcmUtdGVzdCIsICJQb3N0LXRlc3QiLCJOYXRpdmUtU3BlYWtlciIsICJQaWN0dXJlLU5hbWluZyIpKQ0KDQojR2V0IHJpZCBvZiBwYXJ0aWNpcGFudDQ4J3MgcHJvZHVjdGlvbiBkYXRhIGFuZCBvdGhlciB1bmlkZW50aWZpYWJsZSB0cmlhbHMgd2l0aCBxdWFsaXR5IGlzc3Vlcy4gDQpQUk8gPSBQUk9bIShQUk8kc3ViamVjdD09IjQ4IiksXQ0KUFJPID0gUFJPW1BSTyR0b25lICE9IDAsIF0NClBDID0gc3Vic2V0KFBSTywgc2Vzc2lvbiA9PSAiTmF0aXZlLVNwZWFrZXIiKQ0KQ1dSID0gc3Vic2V0KFBSTywgc2Vzc2lvbiAlaW4lIGMoIlByZS10ZXN0IiwgIlBvc3QtdGVzdCIpKQ0KQ1BOID0gc3Vic2V0KFBSTywgc2Vzc2lvbiA9PSAiUGljdHVyZS1OYW1pbmciKQ0KDQojIyBTZXQgdXAgZGF0YSBmb3IgV29yZCBSZXBldGl0aW9uDQpOV1IgPSBDV1INCk5XUiRjb25kaXRpb24gPSBmYWN0b3IoTldSJGNvbmRpdGlvbiwgbGV2ZWxzID0gYygiMCIsICIxIiwiMiIpLCBsYWJlbHMgPSBjKCJMViIsICJIViIsICJIVkIiKSkNCk5XUiR3b3JkdHlwZSA9IGZhY3RvcihOV1Ikd29yZHR5cGUsIGxldmVscyA9IGMoInRyYWluZWQiLCAidW50cmFpbmVkIiksIGxhYmVscyA9IGMoIlRyYWluZWQiLCAiVW50cmFpbmVkIikpDQpOV1IyID0gbWVyZ2UoTldSLCBJRF9wcmUpDQoNCiMjIFNldCB1cCBkYXRhIGZvciBQaWN0dXJlIE5hbWluZyANCk5QTiA9IENQTg0KTlBOJGNvbmRpdGlvbiA9IGZhY3RvcihOUE4kY29uZGl0aW9uLCBsZXZlbHMgPSBjKCIwIiwgIjEiLCAiMiIpLCBsYWJlbHMgPSBjKCJMViIsICJIViIsICJIVkIiKSkNCk5QTjIgPSBtZXJnZShJRF9wcmUsIE5QTikNCg0KDQojIFNldCB1cCBkYXRlIGZvciAzSU8NCmRpcyRjb25kaXRpb24gPSBmYWN0b3IoZGlzJGNvbmRpdGlvbiwgbGV2ZWxzID0gYygiMCIsICIxIiwgIjIiKSwgbGFiZWxzID0gYygiTFYiLCAiSFYiLCAiSFZCIikpDQpkaXMkc2Vzc2lvbiA9IGZhY3RvcihkaXMkc2Vzc2lvbiwgbGV2ZWxzID0gYygiMSIsICIyIiksIGxhYmVscyA9IGMoIlByZS10ZXN0IiwgIlBvc3QtdGVzdCIpKQ0KZGlzJHRyaWFsdHlwZSA9IGRpcyR2b2ljZXR5cGUgPSBmYWN0b3IoZGlzJHZvaWNldHlwZSwgbGV2ZWxzID0gYygiZmZmIiwgImZmbSIsICJmbWYiKSwgbGFiZWxzID0gYygiTmV1dHJhbCIsICJFYXN5IiwgIkhhcmQiKSkNCmRpcyR3b3JkdHlwZSA9IGZhY3RvcihkaXMkd29yZHR5cGUsIGxldmVscyA9IGMoIm5ld3dvcmQiLCAib2xkd29yZCIpLCBsYWJlbHMgPSBjKCJVbnRyYWluZWQgSXRlbSIsICJUcmFpbmVkIEl0ZW0iKSkNCmRpczIgPSBtZXJnZShJRF9wcmUsIGRpcykNCg0KI1NldCB1cCBkYXRlIGZvciBUcmFpbmluZw0KdHJhaW4kY29uZGl0aW9uID0gZmFjdG9yKHRyYWluJGNvbmRpdGlvbiwgbGV2ZWxzID0gYygiMCIsIjIiLCIxIiksIGxhYmVscyA9IGMoIkxWIiwgIkhWQiIsICJIViIpKQ0KdHJhaW4yID0gbWVyZ2UoSURfcHJlLCB0cmFpbikNCmBgYA0KDQoNCg0KDQoNCg0KDQojIEV2aWRlbmNlIGZvci8gYWdhaW5zdCBoeXBvdGhlc2lzIG9mIGdyZWF0ZXIgZ2VuZXJhbGl6YXRpb24gdG8gbm92ZWwgdm9pY2VzLyBpbiBwcm9kdWN0aW9uIGFmdGVyIG11bHRpcGxlIHZvaWNlIHRyYWluaW5nDQojIyBOb3Rlcw0KDQpLZXkgZ29hbCBpcyB0byBsb29rIGZvciBldmlkZW5jZSBmb3IgdGhlIGh5cG90aGVzaXMgdGhhdCBwYXJ0aWNpcGFudHMgc2hvdyBiZXR0ZXIgZ2VuZXJhbGl6YXRpb24gdG8gbm92ZWwgdm9pY2VzLCBvciBpbiBwcm9kdWN0aW9uLCBhZnRlciBIViBvciBIVkIgdHJhaW5pbmcgdGhhbiBhZnRlciBMViB0cmFpbmluZy4NCg0KUG9pbnRzOg0KMSkgVG8gaGF2ZSBtYXhpbXVtIGRhdGEgKG1heGltaXplIHRoZSBldmlkZW5jZSksIHdlIGxvb2sgZm9yIGV2aWRlbmNlIGZvciBncmVhdGVyIGxlYXJuaW5nIGluIGJvdGggSFYvSFZCIHRoYW4gaW4gTFYuIFRvIGdldCB0aGlzLCB3ZSBjb2RlIGEgYmluYXJ5IGNvbnRyYXN0IExWIHZlcnN1cyBIVitIVkIgKGNlbnRlcmVkKS4gRm9yIHRoZSBwb3N0LXRlc3RzIChQaWN0dXJlIElEL1BpY3R1cmUgTmFtaW5nKSB3ZSBhcmUgaW50ZXJlc3RlZCBpbiB0aGUgZXZpZGVuY2UgZm9yIGEgbWFpbiBlZmZlY3Qgb2YgdGhpcyBjb250cmFzdC4gRm9yIHRoZSBwcmUtcG9zdCB0ZXN0cywgd2UgYXJlIGludGVyZXN0ZWQgaW4gdGhlIGludGVyYWN0aW9uIGJldHdlZW4gdGhpcyBjb250cmFzdCBhbmQgc2Vzc2lvbi4NCjIpIEluIFBJIHRlc3Qgb25seSB1bnRyYWluZWQgdm9pY2UgdGVzdCBpdGVtcyBhcmUgcmVsZXZhbnQgc2luY2Ugd2UgZG9uJ3QgcHJlZGljdCBiZXR0ZXIgcGVyZm9ybWFuY2Ugd2l0aCBIViBmb3IgdHJhaW5lZCBpdGVtcy4NCjMpIEluIDNJTyB0ZXN0IGFuZCBXb3JkIFJlcGV0aXRpb246IHdlIGxvb2sgYXQgdHJhaW5lZCBhbmQgdW50cmFpbmVkIGl0ZW1zIGNvbWJpbmVkICh0byBtYXhpbWl6ZSBwb3dlciBhbmQgYmVjYXVzZSBpdCdzIGFsbCBnZW5lcmFsaXphdGlvbiBhd2F5IGZyb20gdHJhaW5lZCAqdm9pY2UqKQ0KNCkgSW4gdGhlIHByb2R1Y3Rpb24gbWVhc3VyZXMgLSBXb3JkIFJlcCBhbmQgUGljdHVyZSBOYW1pbmcgLSB3ZSBhcmUgaW50ZXJlc3RlZCBpbiBib3RoIHRvbmUgYW5kIHBpbnlpbi4gVGhlIGxhdHRlciBpcyByZWxldmFudCBnaXZlbiB0aGUgZmluZGluZ3Mgb2YgQmFyY3JvZnQgYW5kIFNvbW1lcnMgdGhhdCB0aGVyZSBpcyBtb3JlIGdlbmVyYWxpemF0aW9uIGluIHByb2R1Y3Rpb24gYWZ0ZXIgZXhwb3N1cmUgdG8gbXVsdGlwbGUgdm9pY2VzLiBIb3dldmVyIGZvciBXb3JkIFJlcCwgc2luY2Ugd2UgZGlkbid0IHNlZSBhbnkgb3ZlcmFsbCBpbXByb3ZlbWVudCBmcm9tIHByZS0gdG8gcG9zdC0sIHdlIGRvbid0IGxvb2sgYXQgd2hldGhlciB0aGVyZSBpcyBldmlkZW5jZSB0aGF0IHRoZXkgaW1wcm92ZSBtb3JlIGluIG9uZSBjb25kaXRpb24gdGhhdCB0aGUgb3RoZXIuDQo2KSBGb2xsb3dpbmcgRGllbm5lcyAyMDA4LCB3ZSBtb2RlbCBIMSB1c2luZyBhIGhhbGYgbm9ybWFsIHdpdGggYSBtZWFuIG9mIDAgYW5kIGFuIHNkIHNldCB0byBiZSBhbiBlc3RpbWF0ZSBvZiBIMS4gVGhpcyBiaWFzZXMgc21hbGxlciB2YWx1ZXMuIFdlIHRha2Ugb3VyIGVzdGltYXRlcyBvZiBIMSB1c2luZyBlc3RpbWF0ZXMgZnJvbSBlbHNld2hlcmUgd2l0aGluIHRoZSBkYXRhIHdoaWNoIGluZm9ybSB0aGUgbGlrZWx5IHNpemUgb2YgdGhlIGVmZmVjdC4gVGhlIG1ldGhvZCBmb3IgaW5mb3JtaW5nIGVhY2ggb25lIGlzIGV4cGxhaW5lZCBiZWxvdy4gV2UgdGFrZSBCRiA8IDEvMyB0byBiZSBzdWJzdGFudGlhbCBldmlkZW5jZSBmb3IgdGhlIG51bGwgYW5kIEJGID4gMyB0byBiZSBzdWJzdGFudGlhbCBldmlkZW5jZSBmb3IgSDE7IHZhbHVlcyBpbiBiZXR3ZWVuIGFyZSBhbWJpZ3VvdXMuIEluIGFkZGl0aW9uIHRvIGNvbXB1dGluZyB0aGUgQkYgZm9yIGEgcGFydGljdWxhciB2YWx1ZSBvZiBIMSBmb3IgZWFjaCBjb250cmFzdCwgd2UgYWxzbyBjb21wdXRlIGEgInJvYnVzdG5lc3MgcmVnaW9uIiBmb3IgZWFjaCBCYXllcyBmYWN0b3Igd2hpY2ggc2hvd3MgdGhlIHJhbmdlIG9mIHZhbHVlcyBvZiBIMSBvdmVyIHdoaWNoIHdlIGhhdmUgYW1iaWd1b3VzL3N1YnN0YW50aWFsLWV2aWRlbmNlSDEvIHN1YnN0YW50aWFsLWV2aWRlbmNlSDAuDQoNCiMjIFBpY3R1cmUgSWRlbnRpZmljYXRpb24gDQoNClJ1biBsbWVyIG1vZGVsIHdpdGgganVzdCBub3ZlbCB2b2ljZXMgZGF0YSBhbmQgZmFjdG9yIGNvbmRpdGlvbjIgbGV2ZWxzIExWIHZlcnN1cyBIVitIVkINCg0KYGBge3J9DQpQSTIkY29uZGl0aW9uMiA9IFBJMiRjb25kaXRpb24NClBJMiRjb25kaXRpb24yW1BJMiRjb25kaXRpb249PSJIVkIiXT0gIkhWIg0KUEkyLk5ld1ZvaWNlID0gc3Vic2V0IChQSTIsIHZvaWNldHlwZSA9PSAiTmV3IHZvaWNlIikNClBJMi5OZXdWb2ljZT0gbGl6Q2VudGVyKFBJMi5OZXdWb2ljZSwgbGlzdCgidm9pY2V0eXBlIiwgImFwdGl0dWRlIiwgImNvbmRpdGlvbjIiKSkNCg0KcF9nbG1lciA9IGdsbWVyKHNjb3JlIH4gIGNvbmRpdGlvbjIuY3QNCiAgICAgICAgICAgICAgICArICgxfHN1YmplY3QpDQogICAgICAgICAgICAgICAgLCBmYW1pbHkgPSAiYmlub21pYWwiLCANCiAgICAgICAgICAgICAgICBjb250cm9sID0gZ2xtZXJDb250cm9sKG9wdGltaXplciA9ICJib2J5cWEiKSwgDQogICAgICAgICAgICAgICAgZGF0YSA9IFBJMi5OZXdWb2ljZSkNCmthYmxlKHJvdW5kKHN1bW1hcnkocF9nbG1lcikkY29lZmYsMykpDQpgYGANCg0KTW9kZWwgb2YgdGhlIGRhdGEgaXMgdGhlIGVzdGltYXRlIGFuZCBzdC5lcnJvciBmb3IgY29uZGl0aW9uMi5jdA0KDQpWYWx1ZSB0byBpbmZvcm0gSDE6DQoNCldvcmsgb3V0IGEgcGxhdXNpYmxlIG1heGltdW0gLSBhbmQgdXNlIGhhbGYgdGhpcyBhcyB0aGUgZXN0aW1hdGUgLSB1c2luZyB0aGUgaW50ZXJjZXB0IChncmFuZCBtZWFuKS4NCg0KLSBpZiAiTFYiIGlzIHRoZSBsb2dvZGRzIG9mIHRoZSBwcm9wb3J0aW9uIGNvcnJlY3QgaW4gTFYgY29uZGl0aW9uICAgDQotICJIViIgaXMgdGhlIGxvZ29kZHMgb2YgdGhlIHByb3BvcnRpb24gY29ycmVjdCBpbiBIViBjb25kdGlvbg0KDQpBc3N1bXB0aW9ucyBmb3IgcGxhdXNpYmxlIG1heGltdW06ICAgDQotIG1pbmltYWwgcGVyZm9ybWFuY2UgaW4gTFYgLSBpLmUuIGF0IGNoYW5jZSAobm90ZSBjaGFuY2UgaGVyZSBpcyBsb2dvZGRzIG9mIDUwJSwgaS5lLiAwKQ0KLSBhYm92ZSBjaGFuY2UgaW4gSFYsIG1lYW5pbmcgdGhhdCBwb3N0LXRlc3QgZHJpdmVzIGFsbCBvZiB0aGUgZWZmZWN0DQoNCnRodXM6DQpjb25kaXRpb24NCj0gKEhWIC0gY2hhbmNlKSAtIChMViAtIGNoYW5jZSkNCj0gSFYgLSBjaGFuY2UgLSAwDQo9IEhWIC0gMA0KPSBIVg0KDQppbnRlcmNlcHQNCj0gKEhWK0xWKSkvMg0KPSAoSFYrMCkpLzINCj0gSFYvMg0KDQoyICogaW50ZXJjZXB0ID0gSFYNCmNvbmRpdGlvbiA9ICAyICogIGludGVyY2VwdA0KDQpzaW5jZSB0aGlzIGlzIGFuIGVzdGltYXRlIG9mIHRoZSBtYXhpbXVtIHZhbHVlIG9mIGNvbmRpdGlvbiwgd2UgaGFsdmUgaXQgdG8gZ2V0IG91ciBlc3RpbWF0ZSB0byB1c2UgYXMgdGhlIHNkIG9mIHRoZSBoYWxmIG5vcm1hbDogIA0KDQpzZHRoZW9yeSA9IGludGVyY2VwdCAgDQoNCnZhbHVlcyBmb3IgQmF5ZXMgZmFjdG9yIGNhbGN1bGF0aW9uIChjb21wdXRlZCBiZWxvdykNCg0KYGBge3J9DQpjb250cmFzdCA9ICJQSSwgTm92ZWxWb2ljZTogTFYgdmVyc3VzIEhWIg0Kc3RkLkVycm9yID0gcm91bmQoc3VtbWFyeShwX2dsbWVyKSRjb2VmZlsiY29uZGl0aW9uMi5jdCIsICJTdGQuIEVycm9yIiBdLDMpDQplc3RpbWF0ZSA9IHJvdW5kKHN1bW1hcnkocF9nbG1lcikkY29lZmZbImNvbmRpdGlvbjIuY3QiLCAiRXN0aW1hdGUiIF0sMykNCmgxX3NkID0gcm91bmQoc3VtbWFyeShwX2dsbWVyKSRjb2VmZlsiKEludGVyY2VwdCkiLCAiRXN0aW1hdGUiIF0sMykNCnRhaWxzID0gMQ0KUElfdGFibGUgPSBkYXRhLmZyYW1lKGNiaW5kKGNvbnRyYXN0LCBzdGQuRXJyb3IsIGVzdGltYXRlLCBoMV9zZCx0YWlscykpICANCmthYmxlKFBJX3RhYmxlKQ0KYGBgDQoNCiMjIFBpY3R1cmUgTmFtaW5nOiB0b25lIGFjY3VyYWN5DQoNClJ1biBsbWVyIG1vZGVsIHdpdGggZmFjdG9yIGNvbmRpdGlvbjIgbGV2ZWxzIExWIHZlcnN1cyBIVitIVkINCg0KYGBge3J9DQpOUE4yJGNvbmRpdGlvbjIgPSBOUE4yJGNvbmRpdGlvbg0KTlBOMiRjb25kaXRpb24yW05QTjIkY29uZGl0aW9uPT0iSFZCIl09ICJIViINCg0KTlBOMiA9IGxpekNlbnRlcihOUE4yLCBsaXN0KCJjb25kaXRpb24yIikpDQoNCk5QTl9nbG1lciA9IGdsbWVyKHRvbmVfc2NvcmUgfiBjb25kaXRpb24yLmN0DQogICAgICAgICAgICAgICAgICArICgxfHN1YmplY3QpDQogICAgICAgICAgICAgICAgICAsIGZhbWlseSA9ICJiaW5vbWlhbCIsIA0KICAgICAgICAgICAgICAgICAgY29udHJvbCA9IGdsbWVyQ29udHJvbChvcHRpbWl6ZXIgPSAiYm9ieXFhIiksIA0KICAgICAgICAgICAgICAgICAgZGF0YSA9IE5QTjIpDQprYWJsZShyb3VuZChzdW1tYXJ5KE5QTl9nbG1lcikkY29lZmYsMykpDQpgYGANCg0KTW9kZWwgb2YgdGhlIGRhdGEgaXMgdGhlIGVzdGltYXRlIGFuZCBzdC5lcnJvciBmb3IgY29uZGl0aW9uMi5jdA0KVmFsdWUgdG8gaW5mb3JtIEgxOg0KDQpXb3JrIG91dCBhIHBsYXVzaWJsZSBtYXhpbXVtIC0gYW5kIHVzZSBoYWxmIHRoaXMgYXMgdGhlIGVzdGltYXRlIC0gdXNpbmcgdGhlIGludGVyY2VwdCAoZ3JhbmQgbWVhbikuDQoNCmlmICJMViIgaXMgdGhlIGxvZ29kZHMgb2YgdGhlIHByb3BvcnRpb24gY29ycmVjdCBpbiBMViBjb25kaXRpb24gYW5kICJIViIgaXMgdGhlIGxvZ29kZHMgb2YgdGhlIHByb3BvcnRpb24gY29ycmVjdCBpbiBIViBjb25kaXRpb24NCg0KQXNzdW1wdGlvbnMgZm9yIHBsYXVzaWJsZSBtYXhpbXVtOiANCi0gY2hhbmNlIHByb2R1Y3Rpb24gb2YgdG9uZXMgaW4gTFYgY29uZGl0aW9uIC0gIDEvNCAoaS5lLiBpZiB0aGV5IHdlcmUgYXQgY2hhbmNlIGluIHByb2R1Y2luZyBlYWNoIG9mIGZvdXIgdG9uZXMpIGluIGxvZ29kZHMgc3BhY2UNCi0gYWJvdmUgbWluaW1hbCBwZXJmb3JtYW5jZSBpbiBIViwgbWVhbmluZyB0aGF0IEhWIGRyaXZlcyBhbGwgb2YgdGhlIGRpZmZlcmVuY2UgYmV0d2VlbiB0aGUgaW50ZXJjZXB0IGFuZCBjaGFuY2UNCg0KdGh1czoNCmNvbmRpdGlvbg0KPSAoSFYgLSBjaGFuY2UpIC0gKExWIC0gY2hhbmNlKQ0KPSBIViAtIGxvZ29kZHMoMS80KSAtIDANCj0gSFYgLSBsb2dvZGRzKDEvNCkNCg0KaW50ZXJjZXB0DQo9IChIVitMVikvMg0KMiAqaW50ZXJjZXB0ID0gSFYgKyBMVg0KPSBIViArIGxvZ29kZHMoMS80KQ0KSFYgPSAyICogaW50ZXJjZXB0IC0gbG9nb2RkcygxLzQpDQoNCnN1YmJpbmcgaW4gZm9yIEhWOg0KDQpjb25kaXRpb24gPSAoMiAqIGludGVyY2VwdCAtIGxvZ29kZHMoMS80KSkgLSBsb2dvZGRzKDEvNCkNCj0gMiooaW50ZXJjZXB0IC0gbG9nb2RkcygxLzQpKSAgDQoNCnNpbmNlIHRoaXMgaXMgYW4gZXN0aW1hdGUgb2YgdGhlIG1heGltdW0gdmFsdWUgb2YgY29uZGl0aW9uLCB3ZSBoYWx2ZSBpdCB0byBnZXQgb3VyIGVzdGltYXRlIHRvIHVzZSBhcyB0aGUgc2Qgb2YgdGhlIGhhbGYgbm9ybWFsOiAgDQoNCnNkdGhlb3J5ID0gaW50ZXJjZXB0IC0gbG9nb2RkcygxLzQpICANCg0KYGBge3J9DQpjb250cmFzdCA9ICJQTjogTFYgdmVyc3VzIEhWIg0Kc3RkLkVycm9yID0gcm91bmQoc3VtbWFyeShOUE5fZ2xtZXIpJGNvZWZmWyJjb25kaXRpb24yLmN0IiwgIlN0ZC4gRXJyb3IiIF0sMykNCmVzdGltYXRlID0gcm91bmQoc3VtbWFyeShOUE5fZ2xtZXIpJGNvZWZmWyJjb25kaXRpb24yLmN0IiwgIkVzdGltYXRlIiBdLDMpDQpoMV9zZCA9IHJvdW5kKHN1bW1hcnkoTlBOX2dsbWVyKSRjb2VmZlsiKEludGVyY2VwdCkiLCAiRXN0aW1hdGUiIF0tIGxvZ29kZHMoMS80KSwzKQ0KdGFpbHMgPSAxDQpQTl90YWJsZSA9IGRhdGEuZnJhbWUoY2JpbmQoY29udHJhc3QsIHN0ZC5FcnJvciwgZXN0aW1hdGUsIGgxX3NkLHRhaWxzKSkgIA0Ka2FibGUoUE5fdGFibGUpDQpgYGANCg0KDQojIyBQaWN0dXJlIE5hbWluZzogcGlueWluIGFjY3VyYWN5DQpSdW4gbG1lciBtb2RlbCB3aXRoIGZhY3RvciBjb25kaXRpb24yIGxldmVscyBMViB2ZXJzdXMgSFYrSFZCDQoNCmBgYHtyfQ0KTlBOMiRjb25kaXRpb24yID0gTlBOMiRjb25kaXRpb24NCk5QTjIkY29uZGl0aW9uMltOUE4yJGNvbmRpdGlvbj09IkhWQiJdPSAiSFYiDQojdGFibGUoTlBOMiRjb25kaXRpb24yLCBOUE4yJGNvbmRpdGlvbikNCk5QTjIgPSBsaXpDZW50ZXIoTlBOMiwgbGlzdCgiY29uZGl0aW9uMiIpKQ0KDQpOUE5fcGlueWluX2dsbWVyID0gZ2xtZXIocGlueWluX3Njb3JlIH4gY29uZGl0aW9uMi5jdA0KICAgICAgICAgICAgICAgICAgKyAoMXxzdWJqZWN0KQ0KICAgICAgICAgICAgICAgICAgLCBmYW1pbHkgPSAiYmlub21pYWwiLCANCiAgICAgICAgICAgICAgICAgIGNvbnRyb2wgPSBnbG1lckNvbnRyb2wob3B0aW1pemVyID0gImJvYnlxYSIpLCANCiAgICAgICAgICAgICAgICAgIGRhdGEgPSBOUE4yKQ0Ka2FibGUocm91bmQoc3VtbWFyeShOUE5fcGlueWluX2dsbWVyKSRjb2VmZiwzKSkNCmBgYA0KDQoNCk1vZGVsIG9mIHRoZSBkYXRhIGlzIHRoZSBlc3RpbWF0ZSBhbmQgc3QuZXJyb3IgZm9yIGNvbmRpdGlvbjIuY3QNClZhbHVlIHRvIGluZm9ybSBIMToNCg0KV29yayBvdXQgYSBwbGF1c2libGUgbWF4aW11bSAtIGFuZCB1c2UgaGFsZiB0aGlzIGFzIHRoZSBlc3RpbWF0ZSAtIHVzaW5nIHRoZSBpbnRlcmNlcHQgKGdyYW5kIG1lYW4pLg0KDQppZiAiTFYiIGlzIHRoZSBsb2dvZGRzIG9mIHRoZSBwcm9wb3J0aW9uIGNvcnJlY3QgaW4gTFYgY29uZGl0aW9uIGFuZCAiSFYiIGlzIHRoZSBsb2dvZGRzIG9mIHRoZSBwcm9wb3J0aW9uIGNvcnJlY3QgaW4gSFYgY29uZGl0aW9uDQoNCkFzc3VtcHRpb25zIGZvciBwbGF1c2libGUgbWF4aW11bTogDQotIG1pbmltYWwgcGVyZm9ybWFuY2UgaW4gTFYgLSB0aGlzIGlzIGFjdHVhbGx5IDAlIGJ1dCB3ZSBjYW4ndCBjb21wdXRlIHRoaXMgaW4gbG9nb2RkcyBzcGFjZS4gV2UgdGhlcmVmb3JlIHNldCB0aGlzIHRvIGJlIGVxdWl2YWxlbnQgdG8gb25lIGNvcnJlY3QgKGkuZS4gMS83MikgaW4gbG9nb2RkcyBzcGFjZQ0KLSBhYm92ZSBtaW5pbWFsIHBlcmZvcm1hbmNlIGluIEhWLCBtZWFuaW5nIHRoYXQgSFYgZHJpdmVzIGFsbCBvZiB0aGUgZGlmZmVyZW5jZSBiZXR3ZWVuIHRoZSBpbnRlcmNlcHQgYW5kIHRoZSBtaW5pbXVtDQoNCnRodXM6DQpjb25kaXRpb24NCj0gKEhWIC0gbWluaW11bSkgLSAoTFYgLSBtaW5pbXVtKQ0KPSBIViAtIGxvZ29kZHMoMS83MikgLSAwDQo9IEhWIC0gbG9nb2RkcygxLzcyKQ0KDQppbnRlcmNlcHQNCj0gKEhWK0xWKS8yIA0KMiAqIGludGVyY2VwdCA9IEhWK0xWDQo9IEhWICsgbG9nb2RkcygxLzcyKQ0KSFYgPSAyICogaW50ZXJjZXB0IC0gbG9nb2RkcygxLzcyKQ0KDQpzdWJiaW5nIGluIGZvciBIVjoNCg0KY29uZGl0aW9uID0gKDIgKiBpbnRlcmNlcHQgLSBsb2dvZGRzKDEvNzIpKSAtIGxvZ29kZHMoMS83MikNCj0gMiooaW50ZXJjZXB0IC0gbG9nb2RkcygxLzcyKSkgIA0KDQpzaW5jZSB0aGlzIGlzIGFuIGVzdGltYXRlIG9mIHRoZSBtYXhpbXVtIHZhbHVlIG9mIGNvbmRpdGlvbiwgd2UgaGFsdmUgaXQgdG8gZ2V0IG91ciBlc3RpbWF0ZSB0byB1c2UgYXMgdGhlIHNkIG9mIHRoZSBoYWxmIG5vcm1hbDogIA0KDQpzZHRoZW9yeSA9IGludGVyY2VwdCAtIGxvZ29kZHMoMS83MikgIA0KDQpgYGB7cn0NCmNvbnRyYXN0ID0gIlBOLCBwaW55aW46IExWIHZlcnN1cyBIViINCnN0ZC5FcnJvciA9IHJvdW5kKHN1bW1hcnkoTlBOX3Bpbnlpbl9nbG1lcikkY29lZmZbImNvbmRpdGlvbjIuY3QiLCAiU3RkLiBFcnJvciIgXSwzKQ0KZXN0aW1hdGUgPSByb3VuZChzdW1tYXJ5KE5QTl9waW55aW5fZ2xtZXIpJGNvZWZmWyJjb25kaXRpb24yLmN0IiwgIkVzdGltYXRlIiBdLDMpDQpoMV9zZCA9IHJvdW5kKHN1bW1hcnkoTlBOX3Bpbnlpbl9nbG1lcikkY29lZmZbIihJbnRlcmNlcHQpIiwgIkVzdGltYXRlIiBdLSBsb2dvZGRzKDEvNzIpLDMpDQp0YWlscyA9IDENClBOX3Bpbnlpbl90YWJsZSA9IGRhdGEuZnJhbWUoY2JpbmQoY29udHJhc3QsIHN0ZC5FcnJvciwgZXN0aW1hdGUsIGgxX3NkLHRhaWxzKSkgIA0Ka2FibGUoUE5fcGlueWluX3RhYmxlKQ0KYGBgDQoNCg0KDQojIyBXb3JkIFJlcGV0aXRpb246IHRvbmUgYWNjdXJhY3kNCg0KUnVuIGxtZXIgbW9kZWwgd2l0aCBmYWN0b3IgY29uZGl0aW9uMiBsZXZlbHMgTFYgdmVyc3VzIEhWK0hWQg0KDQoNCmBgYHtyfQ0KTldSMiRjb25kaXRpb24yID0gTldSMiRjb25kaXRpb24NCk5XUjIkY29uZGl0aW9uMltOV1IyJGNvbmRpdGlvbiA9PSAiSFZCIl09ICJIViINCk5XUjIgPSBsaXpDZW50ZXIoTldSMiwgbGlzdCgid29yZHR5cGUiLCJjb25kaXRpb24yIikpDQoNCm53X2dsbWVyID0gZ2xtZXIodG9uZV9zY29yZSB+IHdvcmR0eXBlLmN0ICogY29uZGl0aW9uMi5jdCAqIHNlc3Npb24NCiAgICAgICAgICAgICAgKyAod29yZHR5cGUuY3QgKiBzZXNzaW9ufHxzdWJqZWN0KQ0KICAgICAgICAgICAgICAsIGZhbWlseT0iYmlub21pYWwiLCANCiAgICAgICAgICAgICAgY29udHJvbCA9IGdsbWVyQ29udHJvbChvcHRpbWl6ZXIgPSAiYm9ieXFhIiksIA0KICAgICAgICAgICAgICBkYXRhID0gTldSMikNCg0Ka2FibGUocm91bmQoc3VtbWFyeShud19nbG1lcikkY29lZmYsMykpDQoNCg0KDQpgYGANCg0KDQpNb2RlbCBvZiB0aGUgZGF0YSBpcyB0aGUgZXN0aW1hdGUgYW5kIHN0LmVycm9yIGZvciBjb25kaXRpb24yLmN0OnNlc3Npb25Qb3N0LXRlc3QgKGNvbmRpdGlvbiBieSBzZXNzaW9uKQ0KDQpWYWx1ZSB0byBpbmZvcm0gSDE6DQoNCldvcmsgb3V0IGEgcGxhdXNpYmxlIG1heGltdW0gLSBhbmQgdXNlIGhhbGYgdGhpcyBhcyB0aGUgZXN0aW1hdGUgLSB1c2luZyB0aGUgbWFpbiBlZmZlY3Qgb2Ygc2Vzc2lvbiAob3ZlcmFsbCBkaWZmZXJlbmNlIGJldHdlZW4gcHJlIGFuZCBwb3N0KSAuDQoNCmlmIA0KIkxWLlBPU1QiIGlzIHRoZSBsb2dvZGRzIG9mIHRoZSBwcm9wb3J0aW9uIGNvcnJlY3QgaW4gTFYgY29uZGl0aW9uIGF0IHBvc3R0ZXN0DQoiTFYuUFJFIiBpcyB0aGUgbG9nb2RkcyBvZiB0aGUgcHJvcG9ydGlvbiBjb3JyZWN0IGluIExWIGNvbmRpdGlvbiBhdCBwcmV0ZXN0DQoiSFYuUE9TVCIgaXMgdGhlIGxvZ29kZHMgb2YgdGhlIHByb3BvcnRpb24gY29ycmVjdCBpbiBIViBjb25kaXRpb24gYXQgcG9zdHRlc3QNCiJIVi5QUkUiIGlzIHRoZSBsb2dvZGRzIG9mIHRoZSBwcm9wb3J0aW9uIGNvcnJlY3QgaW4gSFYgY29uZGl0aW9uIGF0IHByZXRlc3QNCg0KQXNzdW1wdGlvbnMgZm9yIHBsYXVzaWJsZSBtYXhpbXVtOiANCi0gaW1wcm92ZW1lbnQgZnJvbSBwcmUgdG8gcG9zdCBpbiBIViANCi0gTk8gaW1wcm92ZW1lbnQgZnJvbSBwcmUgdG8gcG9zdCBpbiBMViBpLmUuIExWLlBPU1Q9TFYuUFJFLCBtZWFuaW5nIGFsbCBvZiB0aGUgZWZmZWN0IG9mIHNlc3Npb24gY29tZXMgZnJvbSB0aGUgSFYgY29uZGl0aW9uIA0KDQp0aHVzOg0KY29uZGl0aW9uIGJ5IHNlc3Npb24NCj0gKEhWLlBPU1QgLSBIVi5QUkUpIC0gKExWLlBPU1QtIExWLlBSRSkNCj0gKEhWLlBPU1QgLSBIVi5QUkUpDQoNCnNlc3Npb24gIA0KPSAoKEhWLlBPU1QgLSBIVi5QUkUpICsgKExWLlBPU1QtIExWLlBSRSkpLzINCj0gKEhWLlBPU1QgLSBIVi5QUkUpLzINCiAgDQoyICogc2Vzc2lvbg0KPSAoSFYuUE9TVCAtIEhWLlBSRSkgIA0KDQpjb25kaXRpb24gYnkgc2Vzc2lvbiA9ICAyICogc2Vzc2lvbiAgDQoNCnNpbmNlIHRoaXMgaXMgYW4gZXN0aW1hdGUgb2YgdGhlIG1heGltdW0gdmFsdWUgb2YgY29uZGl0aW9uLCB3ZSBoYWx2ZSBpdCB0byBnZXQgb3VyIGVzdGltYXRlIHRvIHVzZSBhcyB0aGUgc2Qgb2YgdGhlIGhhbGYgbm9ybWFsOiAgDQoNCnNkdGhlb3J5ID0gc2Vzc2lvbiAgICANCih3aGljaCBpcyBzZXNzaW9uUG9zdC10ZXN0IGluIHRoZSBhYm92ZSBtb2RlbCkNCg0KVG9uZV9hY2N1cmFjeQ0KYGBge3J9DQpjb250cmFzdCA9ICAiV29yZCBSZXBldGl0aW9uLCBUb25lIHNjb3JlOiBMViB2ZXJzdXMgSFYgYnkgc2Vzc2lvbiINCnN0ZC5FcnJvciA9IHJvdW5kKHN1bW1hcnkobndfZ2xtZXIpJGNvZWZmWyJjb25kaXRpb24yLmN0OnNlc3Npb25Qb3N0LXRlc3QiLCAiU3RkLiBFcnJvciIgXSwzKQ0KZXN0aW1hdGUgPSByb3VuZChzdW1tYXJ5KG53X2dsbWVyKSRjb2VmZlsiY29uZGl0aW9uMi5jdDpzZXNzaW9uUG9zdC10ZXN0IiwgIkVzdGltYXRlIiBdLDMpDQoNCmgxX3NkID0gcm91bmQoc3VtbWFyeShud19nbG1lcikkY29lZmZbInNlc3Npb25Qb3N0LXRlc3QiLCAiRXN0aW1hdGUiIF0sMykNCnRhaWxzID0gMQ0Kd29yZHJlcF90YWJsZSA9IGRhdGEuZnJhbWUoY2JpbmQoY29udHJhc3QsIHN0ZC5FcnJvciwgZXN0aW1hdGUsIGgxX3NkLHRhaWxzKSkgIA0Ka2FibGUod29yZHJlcF90YWJsZSkNCmBgYA0KDQojIyBXb3JkIFJlcGV0aXRpb246IHBpbnlpbiBhY2N1cmFjeQ0KDQpSdW4gbG1lciBtb2RlbCB3aXRoIGZhY3RvciBjb25kaXRpb24yIGxldmVscyBMViB2ZXJzdXMgSFYrSFZCDQoNCg0KYGBge3J9DQoNCm53X2dsbWVyX3BpbnlpbiA9IGdsbWVyKHBpbnlpbl9zY29yZSB+IHdvcmR0eXBlLmN0ICogY29uZGl0aW9uMi5jdCAqIHNlc3Npb24NCiAgICAgICAgICAgICAgKyAod29yZHR5cGUuY3QgKiBzZXNzaW9ufHxzdWJqZWN0KQ0KICAgICAgICAgICAgICAsIGZhbWlseT0iYmlub21pYWwiLCANCiAgICAgICAgICAgICAgY29udHJvbCA9IGdsbWVyQ29udHJvbChvcHRpbWl6ZXIgPSAiYm9ieXFhIiksIA0KICAgICAgICAgICAgICBkYXRhID0gTldSMikNCg0Ka2FibGUocm91bmQoc3VtbWFyeShud19nbG1lcl9waW55aW4pJGNvZWZmLDMpKQ0KDQoNCg0KYGBgDQoNCg0KTW9kZWwgb2YgdGhlIGRhdGEgaXMgdGhlIGVzdGltYXRlIGFuZCBzdC5lcnJvciBmb3IgY29uZGl0aW9uMi5jdDpzZXNzaW9uUG9zdC10ZXN0IChjb25kaXRpb24gYnkgc2Vzc2lvbikNCg0KVmFsdWUgdG8gaW5mb3JtIEgxOg0KDQpXb3JrIG91dCBhIHBsYXVzaWJsZSBtYXhpbXVtIC0gYW5kIHVzZSBoYWxmIHRoaXMgYXMgdGhlIGVzdGltYXRlIC0gdXNpbmcgdGhlIG1haW4gZWZmZWN0IG9mIHNlc3Npb24gKG92ZXJhbGwgZGlmZmVyZW5jZSBiZXR3ZWVuIHByZSBhbmQgcG9zdCkgLg0KDQppZiANCiJMVi5QT1NUIiBpcyB0aGUgbG9nb2RkcyBvZiB0aGUgcHJvcG9ydGlvbiBjb3JyZWN0IGluIExWIGNvbmRpdGlvbiBhdCBwb3N0dGVzdA0KIkxWLlBSRSIgaXMgdGhlIGxvZ29kZHMgb2YgdGhlIHByb3BvcnRpb24gY29ycmVjdCBpbiBMViBjb25kaXRpb24gYXQgcHJldGVzdA0KIkhWLlBPU1QiIGlzIHRoZSBsb2dvZGRzIG9mIHRoZSBwcm9wb3J0aW9uIGNvcnJlY3QgaW4gSFYgY29uZGl0aW9uIGF0IHBvc3R0ZXN0DQoiSFYuUFJFIiBpcyB0aGUgbG9nb2RkcyBvZiB0aGUgcHJvcG9ydGlvbiBjb3JyZWN0IGluIEhWIGNvbmRpdGlvbiBhdCBwcmV0ZXN0DQoNCkFzc3VtcHRpb25zIGZvciBwbGF1c2libGUgbWF4aW11bTogDQotIGltcHJvdmVtZW50IGZyb20gcHJlIHRvIHBvc3QgaW4gSFYgDQotIE5PIGltcHJvdmVtZW50IGZyb20gcHJlIHRvIHBvc3QgaW4gTFYgaS5lLiBMVi5QT1NUPUxWLlBSRSwgbWVhbmluZyBhbGwgb2YgdGhlIGVmZmVjdCBvZiBzZXNzaW9uIGNvbWVzIGZyb20gdGhlIEhWIGNvbmRpdGlvbiANCg0KdGh1czoNCmNvbmRpdGlvbiBieSBzZXNzaW9uDQo9IChIVi5QT1NUIC0gSFYuUFJFKSAtIChMVi5QT1NULSBMVi5QUkUpDQo9IChIVi5QT1NUIC0gSFYuUFJFKQ0KDQpzZXNzaW9uICANCj0gKChIVi5QT1NUIC0gSFYuUFJFKSArIChMVi5QT1NULSBMVi5QUkUpKS8yDQo9IChIVi5QT1NUIC0gSFYuUFJFKS8yDQogIA0KMiAqIHNlc3Npb24NCj0gKEhWLlBPU1QgLSBIVi5QUkUpICANCg0KY29uZGl0aW9uIGJ5IHNlc3Npb24gPSAgMiAqIHNlc3Npb24gIA0KDQpzaW5jZSB0aGlzIGlzIGFuIGVzdGltYXRlIG9mIHRoZSBtYXhpbXVtIHZhbHVlIG9mIGNvbmRpdGlvbiwgd2UgaGFsdmUgaXQgdG8gZ2V0IG91ciBlc3RpbWF0ZSB0byB1c2UgYXMgdGhlIHNkIG9mIHRoZSBoYWxmIG5vcm1hbDogIA0KDQpzZHRoZW9yeSA9IHNlc3Npb24gICAgDQood2hpY2ggaXMgc2Vzc2lvblBvc3QtdGVzdCBpbiB0aGUgYWJvdmUgbW9kZWwpDQoNCg0KYGBge3J9DQpjb250cmFzdCA9ICAiV29yZCBSZXBldGl0aW9uLCBQaW55aW4gc2NvcmU6IExWIHZlcnN1cyBIViBieSBzZXNzaW9uIg0Kc3RkLkVycm9yID0gcm91bmQoc3VtbWFyeShud19nbG1lcl9waW55aW4pJGNvZWZmWyJjb25kaXRpb24yLmN0OnNlc3Npb25Qb3N0LXRlc3QiLCAiU3RkLiBFcnJvciIgXSwzKQ0KZXN0aW1hdGUgPSByb3VuZChzdW1tYXJ5KG53X2dsbWVyX3BpbnlpbikkY29lZmZbImNvbmRpdGlvbjIuY3Q6c2Vzc2lvblBvc3QtdGVzdCIsICJFc3RpbWF0ZSIgXSwzKQ0KDQpoMV9zZCA9IHJvdW5kKHN1bW1hcnkobndfZ2xtZXJfcGlueWluKSRjb2VmZlsic2Vzc2lvblBvc3QtdGVzdCIsICJFc3RpbWF0ZSIgXSwzKQ0KdGFpbHMgPSAxDQp3b3JkcmVwX3RhYmxlX3BpbnlpbiA9IGRhdGEuZnJhbWUoY2JpbmQoY29udHJhc3QsIHN0ZC5FcnJvciwgZXN0aW1hdGUsIGgxX3NkLHRhaWxzKSkgIA0Ka2FibGUod29yZHJlcF90YWJsZV9waW55aW4pDQpgYGANCiMjIFRocmVlLWludGVydmFsIE9kZGl0eSBEaXNjcmltaW5hdGlvbiBUYXNrIA0KUnVuIGxtZXIgbW9kZWwgd2l0aCBmYWN0b3IgY29uZGl0aW9uMiBsZXZlbHMgTFYgdmVyc3VzIEhWK0hWQg0KDQpgYGB7cn0NCmRpczIkY29uZGl0aW9uMiA9IGRpczIkY29uZGl0aW9uDQpkaXMyJGNvbmRpdGlvbjJbZGlzMiRjb25kaXRpb24gPT0gIkhWQiJdPSAiSFYiDQpkaXMyID0gbGl6Q2VudGVyKGRpczIsIGxpc3QoICJ3b3JkdHlwZSIsICJhcHRpdHVkZSIsImNvbmRpdGlvbjIiKSkNCmRpczIgPSBsaXpDb250cmFzdHMoZGlzMiwgZGlzMiRjb25kaXRpb24sICJMViIpDQpkaXMyID0gbGl6Q29udHJhc3RzKGRpczIsIGRpczIkdHJpYWx0eXBlLCAiTmV1dHJhbCIpDQoNCmRfZ2xtZXIgPSBnbG1lcihzY29yZSB+IHNlc3Npb24gKiB3b3JkdHlwZS5jdCAqICBjb25kaXRpb24yLmN0DQogICAgICAgICAgICAgICAgKyAoTmV1dHJhbF9WRVJTVVNfRWFzeSArIE5ldXRyYWxfVkVSU1VTX0hhcmQpOiBzZXNzaW9uDQogICAgICAgICAgICAgICAgKyAoTmV1dHJhbF9WRVJTVVNfRWFzeSArIE5ldXRyYWxfVkVSU1VTX0hhcmQpIA0KICAgICAgICAgICAgICAgICsgKHNlc3Npb24gKiB3b3JkdHlwZS5jdHx8c3ViamVjdCkNCiAgICAgICAgICAgICAgICAsIGZhbWlseSA9ICJiaW5vbWlhbCIsIA0KICAgICAgICAgICAgICAgIGNvbnRyb2wgPSBnbG1lckNvbnRyb2wob3B0aW1pemVyID0gImJvYnlxYSIpLCANCiAgICAgICAgICAgICAgICBkYXRhID0gZGlzMikNCg0KDQprYWJsZShyb3VuZChzdW1tYXJ5KGRfZ2xtZXIpJGNvZWZmLDMpKQ0KDQpgYGANCg0KTW9kZWwgb2YgdGhlIGRhdGEgaXMgdGhlIGVzdGltYXRlIGFuZCBzdC5lcnJvciBmb3Igc2Vzc2lvblBvc3QtdGVzdDpjb25kaXRpb24yLmN0IChjb25kaXRpb24gYnkgc2Vzc2lvbikNCg0KVmFsdWUgdG8gaW5mb3JtIEgxIChub3RlOiB0aGlzIGlzIGlkZW50aWNhbCB0byB3b3JkcmVwIGFib3ZlKToNCg0KV29yayBvdXQgYSBwbGF1c2libGUgbWF4aW11bSAtIGFuZCB1c2UgaGFsZiB0aGlzIGFzIHRoZSBlc3RpbWF0ZSAtIHVzaW5nIHRoZSBtYWluIGVmZmVjdCBvZiBzZXNzaW9uIChvdmVyYWxsIGRpZmZlcmVuY2UgYmV0d2VlbiBwcmUgYW5kIHBvc3QpIC4NCg0KaWYgDQoiTFYuUE9TVCIgaXMgdGhlIGxvZ29kZHMgb2YgdGhlIHByb3BvcnRpb24gY29ycmVjdCBpbiBMViBjb25kaXRpb24gYXQgcG9zdHRlc3QNCiJMVi5QUkUiIGlzIHRoZSBsb2dvZGRzIG9mIHRoZSBwcm9wb3J0aW9uIGNvcnJlY3QgaW4gTFYgY29uZGl0aW9uIGF0IHByZXRlc3QNCiJIVi5QT1NUIiBpcyB0aGUgbG9nb2RkcyBvZiB0aGUgcHJvcG9ydGlvbiBjb3JyZWN0IGluIEhWIGNvbmRpdGlvbiBhdCBwb3N0dGVzdA0KIkhWLlBSRSIgaXMgdGhlIGxvZ29kZHMgb2YgdGhlIHByb3BvcnRpb24gY29ycmVjdCBpbiBIViBjb25kaXRpb24gYXQgcHJldGVzdA0KDQpBc3N1bXB0aW9ucyBmb3IgcGxhdXNpYmxlIG1heGltdW06IA0KLSBpbXByb3ZlbWVudCBmcm9tIHByZSB0byBwb3N0IGluIEhWIA0KLSBOTyBpbXByb3ZlbWVudCBmcm9tIHByZSB0byBwb3N0IGluIExWIGkuZS4gTFYuUE9TVD1MVi5QUkUsIG1lYW5pbmcgYWxsIG9mIHRoZSBlZmZlY3Qgb2Ygc2Vzc2lvbiBjb21lcyBmcm9tIHRoZSBIViBjb25kaXRpb24gDQoNCnRodXM6DQpjb25kaXRpb24gYnkgc2Vzc2lvbg0KPSAoSFYuUE9TVCAtIEhWLlBSRSkgLSAoTFYuUE9TVC0gTFYuUFJFKQ0KPSAoSFYuUE9TVCAtIEhWLlBSRSkNCg0Kc2Vzc2lvbiAgDQo9ICgoSFYuUE9TVCAtIEhWLlBSRSkgKyAoTFYuUE9TVC0gTFYuUFJFKSkvMg0KPSAoSFYuUE9TVCAtIEhWLlBSRSkvMg0KICANCjIgKiBzZXNzaW9uDQo9IChIVi5QT1NUIC0gSFYuUFJFKSAgDQoNCmNvbmRpdGlvbiBieSBzZXNzaW9uID0gIDIgKiBzZXNzaW9uICANCg0Kc2luY2UgdGhpcyBpcyBhbiBlc3RpbWF0ZSBvZiB0aGUgbWF4aW11bSB2YWx1ZSBvZiBjb25kaXRpb24sIHdlIGhhbHZlIGl0IHRvIGdldCBvdXIgZXN0aW1hdGUgdG8gdXNlIGFzIHRoZSBzZCBvZiB0aGUgaGFsZiBub3JtYWw6ICANCg0Kc2R0aGVvcnkgPSBzZXNzaW9uICAgIA0KKHdoaWNoIGlzIHNlc3Npb25Qb3N0LXRlc3QgaW4gdGhlIGFib3ZlIG1vZGVsKQ0KDQoNCmBgYHtyfQ0KY29udHJhc3QgPSAiMyBJbnQuIE9kZCwgVG9uZSBzY29yZTogTFYgYnkgSFYgYnkgc2Vzc2lvbiINCnN0ZC5FcnJvciA9IHJvdW5kKHN1bW1hcnkoZF9nbG1lcikkY29lZmZbInNlc3Npb25Qb3N0LXRlc3Q6Y29uZGl0aW9uMi5jdCIsICJTdGQuIEVycm9yIiBdLDMpDQplc3RpbWF0ZSA9IHJvdW5kKHN1bW1hcnkoZF9nbG1lcikkY29lZmZbInNlc3Npb25Qb3N0LXRlc3Q6Y29uZGl0aW9uMi5jdCIsICJFc3RpbWF0ZSIgXSwzKQ0KaDFfc2QgPSByb3VuZChzdW1tYXJ5KGRfZ2xtZXIpJGNvZWZmWyJzZXNzaW9uUG9zdC10ZXN0IiwgIkVzdGltYXRlIiBdLDMpDQp0YWlscyA9IDENCmRpc2NyaW1fdGFibGUgPSBkYXRhLmZyYW1lKGNiaW5kKGNvbnRyYXN0LCBzdGQuRXJyb3IsIGVzdGltYXRlLCBoMV9zZCx0YWlscykpICANCmthYmxlKGRpc2NyaW1fdGFibGUpDQpgYGANCg0KDQoNCiMjIGNvbXB1dGUgYkZzIGFuZCByYW5nZXMNCg0KYGBge3J9DQojZGYgPSByYmluZCh3b3JkcmVwX3RhYmxlLGRpc2NyaW1fdGFibGUsIHdvcmRyZXBfYXBfdGFibGUsIGRpc2NyaW1fYXBfdGFibGUpDQpkZj0gcmJpbmQoUElfdGFibGUsIFBOX3RhYmxlLCBQTl9waW55aW5fdGFibGUsIHdvcmRyZXBfdGFibGUsIHdvcmRyZXBfdGFibGVfcGlueWluLCBkaXNjcmltX3RhYmxlKQ0KDQpkZiRzdGQuRXJyb3IgPSBhcy5udW1lcmljKGFzLmNoYXJhY3RlcihkZiRzdGQuRXJyb3IpKQ0KZGYkZXN0aW1hdGUgPSBhcy5udW1lcmljKGFzLmNoYXJhY3RlcihkZiRlc3RpbWF0ZSkpDQpkZiRoMV9zZCA9IGFzLm51bWVyaWMoYXMuY2hhcmFjdGVyKGRmJGgxX3NkKSkNCmRmJHRhaWxzID0gYXMubnVtZXJpYyhhcy5jaGFyYWN0ZXIoZGYkdGFpbHMpKQ0KZGYkaDFfbW90aXZhdGlvbiA9ICIiDQoNCmRmMiA9IEJmX3RhYmxlKGRmKQ0KDQoja2FibGUoZGYyKQ0KDQoja2FibGUoYWRkQmZfcmFuZ2VzMihkZjIpKQ0KDQpzZHRoZW9yeXJhbmdlID0gc2VxKCBmcm9tID0gMCwgdG8gPSAxMCAsIGxlbmd0aC5vdXQgPSAxMDApDQoNCmFkZEJmX3JhbmdlcyhkZjIsc2R0aGVvcnlyYW5nZSApICU+JQ0KICBrYWJsZSgpICU+JQ0KICBrYWJsZV9zdHlsaW5nKCkNCg0KYGBgDQoNCldlIGhhdmUgc3Vic3RhbnRpYWwgZXZpZGVuY2UgZm9yIHRoZSBudWxsIGluIGVhY2ggdGVzdCBhcGFydCBmcm9tIFdvcmQgUmVwIHBpbnlpbi4gIE5vdGUgdGhhdCByb2J1c3RuZXNzIHJlZ2lvbnMgaW5kaWNhdGUgdGhhdCB3ZSB3b3VsZCBoYXZlIGFsc28gaGF2ZSBvYnRhaW5lZCBzdWJzdGFudGlhbCBCRnMgdXNpbmcgc21hbGxlciB2YWx1ZXMgdGhhbiB0aG9zZSB3ZSB1c2VkLiBOb3RlIHRoYXQgZm9yIGV2aWRlbmNlIGZvciBIMCwgdGhlIG1heGltdW0gc2NhbGUgZmFjdG9yIGlzIGFsd2F5cyBpbmZpbml0eS4NCg0KDQojIEV2aWRlbmNlIGZvci9hZ2FpbnN0IGh5cG90aGVzaXMgdGhhdCBwYXJ0aWNpcGFudHMgd2l0aCBoaWdoZXIgYXB0aXR1ZGUgYmVuZWZpdCBtb3JlIGZyb20gSFYgdHJhaW5pbmcgDQoNCiMjIE5vdGVzOg0KDQpQb2ludHM6DQoxKSBVbmxpa2UgYWJvdmUsIHdlIGFsc28gbG9vayBhdCB0cmFpbmluZyBkYXRhIGhlcmUuIFRoaXMgaXMgYmVjYXVzZSBpbiBTYWRha2F0YSAmIE1jUXVlZW4gdGhlIGludGVyYWN0aW9uIHdhcyBmb3VuZCB3aXRoIHRyYWluZWQgaXRlbXMsIHNvIGl0IGlzbid0IGp1c3QgZ2VuZXJhbGl6YXRpb24gdHJpYWxzIHRoYXQgYXJlIHJlbGV2YW50Lg0KMikgVG8gaGF2ZSBtYXhpbXVtIGRhdGEsIGZvciBhbGwgb2YgdGhlIHRhc2tzIGV4Y2VwdCB0cmFpbmluZywgd2UgZG8gd2hhdCB3ZSBkaWQgYWJvdmUgYW5kIGxvb2sgZm9yIGV2aWRlbmNlIGZvciBncmVhdGVyIGxlYXJuaW5nIGluIGJvdGggSFYvSFZCIHRoYW4gaW4gTFYsIGkuZS4gd2UgY29kZSBhIGJpbmFyeSBjb250cmFzdCBMViB2ZXJzdXMgSFYrSFZCIChjZW50ZXJlZCkuIEZvciB0cmFpbmluZywgd2Ugc2F3IGluIHRoZSBwcmV2aW91cyBhbmFseXNpcyB0aGF0IEhWIGFuZCBIVkIgYXJlIHF1aXRlIGRpZmZlcmVudCwgc28gaGVyZSB3ZSBsb29rIGF0IExWIHYgSFYgYW5kIExWIHYgSFZCIHNlcGFyYXRlbHkgKGFsc28gbm90ZSB3ZSBoYXZlIGEgbG90IG1vcmUgZGF0YSBwb2ludHMgcGVyIHBhcnRpY2lwYW50IGZvciB0cmFpbmluZykuIA0KMykgRm9yIHRoZSBwb3N0LXRlc3RzIChQSS9QTikgd2UgYXJlIGludGVyZXN0ZWQgaW4gdGhlIGV2aWRlbmNlIGZvciBhbiBpbnRlcmFjdGlvbiBiZXR3ZWVuIHRoZSBjb25kaXRpb24gY29udHJhc3QgYW5kIGFwdGl0dWRlLiBGb3IgdGhlIHByZS1wb3N0IHRlc3RzLCB3ZSBhcmUgaW50ZXJlc3RlZCBpbiB0aGUgaW50ZXJhY3Rpb24gYmV0d2VlbiB0aGVjb250cmFzdCwgYXB0aXR1ZGUgYW5kIHNlc3Npb24uIEZvciB0cmFpbmluZyB3ZSBsb29rIGF0IHRoZSBldmlkZW5jZSBmb3IgYW4gaW50ZXJhY3Rpb24gYmV0d2VlbiBlYWNoIGNvbmRpdGlvbiBjb250cmFzdCBhbmQgYXB0aXR1ZGUuIChOT1RFOiBpbnRlcmFjdGlvbiB3aXRoIHNlc3Npb24gd291bGQgYWxzbyBiZSByZWxldmFudCBidXQgdGhlIG1vZGVsIHdvbid0IGNvbnZlcmdlIHdpdGggdGhhdCBpbiAtIGV2ZW4gaWYgd2UgcmVtb3ZlIGFsbCBvZiB0aGUgc2xvcGVzLikNCjQpIEZvciBQSSB0ZXN0LCB0byBoYXZlIG1heGltdW0gcG93ZXIsIHdlIGxvb2sgYXQgY29tYmluYXRpb24gb2YgdHJhaW5lZCBhbmQgdW50cmFpbmVkIHZvaWNlcyAobm90ZTogdGhpcyBpcyB1bmxpa2Ugd2hhdCB3ZSBkaWQgYWJvdmUsIHdoZXJlIHdlIG9ubHkgbG9va2VkIGF0IHVudHJhaW5lZCB2b2ljZXMuIEhvd2V2ZXIsIGZvciB0aGUgaW50ZXJhY3Rpb24gd2l0aCBhcHRpdHVkZSwgb25lIHByZXZpb3VzIHN0dWR5IGZvdW5kIGV2aWRlbmNlIHdpdGggdHJhaW5lZCB2b2ljZXMsIGFuZCBvbmUgd2l0aCB1bnRyYWluZWQsIHNvIHdlIGxvb2sgYWNyb3NzIGJvdGgpLg0KNSkgSW4gM0lPIGFuZCBXb3JkIFJlcGV0aXRpb24gdGVzdDogd2UgbG9vayBhdCB0cmFpbmVkIGFuZCB1bnRyYWluZWQgaXRlbXMgY29tYmluZWQuIA0KNikgSW4gdGhlIHByb2R1Y3Rpb24gbWVhc3VyZXMgLSBXUiBhbmQgUE4gLSB3ZSBhcmUgb25seSBpbnRlcmVzdGVkIGluIHRvbmUgc2luY2Ugb3VyIGFwdGl0dWRlIG1lYXN1cmUgaXMgcmVsZXZhbnQgZm9yIHRvbmUuIA0KDQo3KSBBcyBhYm92ZSwgZm9sbG93aW5nIERpZW5lcyAyMDA4LCB3ZSBtb2RlbCBIMSB1c2luZyBhIGhhbGYgbm9ybWFsIHdpdGggYSBtZWFuIG9mIDAgYW5kIGFuIHNkIHNldCB0byBiZSBhbiBlc3RpbWF0ZSBvZiBIMS4gVGhpcyBiaWFzZXMgc21hbGxlciB2YWx1ZXMuIFdlIHRha2Ugb3VyIGVzdGltYXRlcyBvZiBIMSB1c2luZyBlc3RpbWF0ZXMgZnJvbSBlbHNld2hlcmUgd2l0aGluIHRoZSBkYXRhIHdoaWNoIGluZm9ybSB0aGUgbGlrZWx5IHNpemUgb2YgdGhlIGVmZmVjdC4gVGhlIG1ldGhvZCBmb3IgaW5mb3JtaW5nIGVhY2ggb25lIGlzIGV4cGxhaW5lZCBiZWxvdy4gV2UgdGFrZSBCRiA8IDEvMyB0byBiZSBzdWJzdG5hdGlhbCBldmlkZW5jZSBmb3IgIHRoZSBudWxsIGFuZCBCRiA+IDMgdG8gYmUgc3Vic3RhbnRpYWwgZXZpZGVuY2UgZm9yIEgxOyB2YWx1ZXMgaW4gYmV0d2VlbiBhcmUgYW1iaWd1b3VzLiBJbiBhZGRpdGlvbiB0byBjb21wdXRpbmcgdGhlIEJGIGZvciBhIHBhcnRpY3VsYXIgdmFsdWUgb2YgSDEgZm9yIGVhY2ggY29udHJhc3QsIHdlIGFsc28gY29tcHV0ZSBhICJyb2J1c3RuZXNzIHJlZ2lvbiIgZm9yIGVhY2ggQmF5ZXMgZmFjdG9yIHdoaWNoIHNob3dzIHRoZSByYW5nZSBvZiB2YWx1ZXMgb2YgSDEgb3ZlciB3aGljaCB3ZSBoYXZlIGFtYmlndW91cy9zdWJzdGFudGlhbC1ldmlkZW5jZUgxLyBzdWJzdGFudGlhbC1ldmlkZW5jZUgwKQ0KDQojIyBQaWN0dXJlIElkZW50aWZpY2F0aW9uDQpSdW4gbG1lciBtb2RlbCBmYWN0b3IgY29uZGl0aW9uMiBsZXZlbHMgTFYgdmVyc3VzIEhWK0hWQg0KDQoNCmBgYHtyfQ0KUEkyJGNvbmRpdGlvbjIgPSBQSTIkY29uZGl0aW9uDQpQSTIkY29uZGl0aW9uMltQSTIkY29uZGl0aW9uPT0iSFZCIl09ICJIViINClBJMiA9IGxpekNlbnRlcihQSTIsIGxpc3QoInZvaWNldHlwZSIsICJhcHRpdHVkZSIsICJjb25kaXRpb24yIikpDQoNCnBfYXBfZ2xtZXIgPSBnbG1lcihzY29yZSB+ICBjb25kaXRpb24yLmN0ICphcHRpdHVkZS5jdA0KICAgICAgICAgICAgICAgICsgKDF8c3ViamVjdCkNCiAgICAgICAgICAgICAgICAsIGZhbWlseSA9ICJiaW5vbWlhbCIsIA0KICAgICAgICAgICAgICAgIGNvbnRyb2wgPSBnbG1lckNvbnRyb2wob3B0aW1pemVyID0gImJvYnlxYSIpLCANCiAgICAgICAgICAgICAgICBkYXRhID0gUEkyKQ0Ka2FibGUocm91bmQoc3VtbWFyeShwX2FwX2dsbWVyKSRjb2VmZiwzKSkNCmBgYA0KDQpNb2RlbCBvZiB0aGUgZGF0YSBpcyB0aGUgZXN0aW1hdGUgYW5kIHN0LmVycm9yIGZvciBjb25kaXRpb24yLmN0OmFwdGl0dWRlLmN0ICAoY29uZGl0aW9uIGJ5IGFwdGl0dWRlKQ0KDQpWYWx1ZSB0byBpbmZvcm0gSDE6DQoNCldvcmsgb3V0IGEgcGxhdXNpYmxlIG1heGltdW0gLSBhbmQgdXNlIGhhbGYgdGhpcyBhcyB0aGUgZXN0aW1hdGUgLSB1c2luZyB0aGUgbWFpbiBlZmZlY3Qgb2YgYXB0aXR1ZGUgICg9IGFwdGl0dWRlLmN0IC0gb3ZlcmFsbCBkaWZmZXJlbmNlIGluIHBlcmZvcm1hbmNlIG9uIHRlc3QgZm9yIG9uZSBzdGVwIGluIGFwdGl0dWRlICh3aGljaCBpcyBzZXQgdG8gYmUgMTAlIHBvaW50cyBvbiB0aGUgcGNwdCB0ZXN0KSkuDQoNCmlmIA0KIkxWLmFwdGl0dWRlIiBpcyB0aGUgZWZmZWN0IG9mIGFwdGl0dWRlIGluIHRoZSBMViBjb25kaXRpb24gDQoiSFYuYXB0aXR1ZGUiIGlzIHRoZSBlZmZlY3Qgb2YgYXB0aXR1ZGUgaW4gdGhlIEhWIGNvbmRpdGlvbiANCg0KQXNzdW1wdGlvbnMgZm9yIHBsYXVzaWJsZSBtYXhpbXVtOiANCi0gcG9zaXRpdmUgdmFsdWUgb2YgYXB0aXR1ZGVpbiBIViANCi0gTk8gZWZmZWN0IG9mIGFwdGl0dWRlIGluIExWICg9MCkgW25vdGU6IG5vIHJlYXNvbiB0byB0aGluayB3ZSB3b3VsZCBnZXQgTkVHQVRJVkUgZWZmZWN0IG9mIGFwdGl0dWRlIGluIGVpdGhlciBjb25kaXRpb24sIGJhc2VkIG9uIHByZXZpb3VzIGRhdGFdDQoNCnRodXM6DQpjb25kaXRpb24gYnkgYXB0aXR1ZGUNCj0gSFYuYXB0aXR1ZGUtTFYuYXB0aXR1ZGUNCj0gSFYuYXB0aXR1ZGUtMA0KDQphcHRpdHVkZQ0KPSAoSFYuYXB0aXR1ZGUrTFYuYXB0aXR1ZGUpLzINCj0gKEhWLmFwdGl0dWRlKS8yDQoNCjIgKiBhcHRpdHVkZQ0KPSBIVi5hcHRpdHVkZS0wDQoNCmNvbmRpdGlvbiBieSBhcHRpdHVkZSA9ICAyICogYXB0aXR1ZGUgIA0KDQpzaW5jZSB0aGlzIGlzIGFuIGVzdGltYXRlIG9mIHRoZSBtYXhpbXVtIHZhbHVlIG9mIGNvbmRpdGlvbiwgd2UgaGFsdmUgaXQgdG8gZ2V0IG91ciBlc3RpbWF0ZSB0byB1c2UgYXMgdGhlIHNkIG9mIHRoZSBoYWxmIG5vcm1hbDogIA0KDQpzZHRoZW9yeSA9IGFwdGl0dWRlICANCih3aGljaCBpcyBhcHRpdHVkZS5jdCBpbiB0aGUgYWJvdmUgbW9kZWwpDQoNCmBgYHtyfQ0KY29udHJhc3QgPSAiUElfYXA6IExWIHZlcnN1cyBIViBieSBhcHRpdHVkZSINCnN0ZC5FcnJvciA9IHJvdW5kKHN1bW1hcnkocF9hcF9nbG1lcikkY29lZmZbImNvbmRpdGlvbjIuY3Q6YXB0aXR1ZGUuY3QiLCAiU3RkLiBFcnJvciIgXSwzKQ0KZXN0aW1hdGUgPSByb3VuZChzdW1tYXJ5KHBfYXBfZ2xtZXIpJGNvZWZmWyJjb25kaXRpb24yLmN0OmFwdGl0dWRlLmN0IiwgIkVzdGltYXRlIiBdLDMpDQpoMV9zZCA9IHJvdW5kKHN1bW1hcnkocF9hcF9nbG1lcikkY29lZmZbImFwdGl0dWRlLmN0IiwgIkVzdGltYXRlIiBdLDMpDQp0YWlscyA9IDENClBJX2FwX3RhYmxlID0gZGF0YS5mcmFtZShjYmluZChjb250cmFzdCwgc3RkLkVycm9yLCBlc3RpbWF0ZSwgaDFfc2QsdGFpbHMpKSAgDQprYWJsZShQSV9hcF90YWJsZSkNCmBgYA0KDQojIyBQaWN0dXJlIE5hbWluZw0KDQpSdW4gbG1lciBtb2RlbCB3aXRoIGZhY3RvciBjb25kaXRpb24yIGxldmVscyBMViB2ZXJzdXMgSFYrSFZCDQoNCmBgYHtyfQ0KTlBOMiRjb25kaXRpb24yID0gTlBOMiRjb25kaXRpb24NCk5QTjIkY29uZGl0aW9uMltOUE4yJGNvbmRpdGlvbj09IkhWQiJdPSAiSFYiDQpOUE4yID0gbGl6Q2VudGVyKE5QTjIsIGxpc3QoImNvbmRpdGlvbjIiLCJhcHRpdHVkZSIpKQ0KDQpOUE5fYXBfZ2xtZXIgPSBnbG1lcih0b25lX3Njb3JlIH4gY29uZGl0aW9uMi5jdCphcHRpdHVkZS5jdA0KICAgICAgICAgICAgICAgICAgKyAoMXxzdWJqZWN0KQ0KICAgICAgICAgICAgICAgICAgLCBmYW1pbHkgPSAiYmlub21pYWwiLCANCiAgICAgICAgICAgICAgICAgIGNvbnRyb2wgPSBnbG1lckNvbnRyb2wob3B0aW1pemVyID0gImJvYnlxYSIpLCANCiAgICAgICAgICAgICAgICAgIGRhdGEgPSBOUE4yKQ0Ka2FibGUocm91bmQoc3VtbWFyeShOUE5fYXBfZ2xtZXIpJGNvZWZmLDMpKQ0KYGBgDQoNCg0KTW9kZWwgb2YgdGhlIGRhdGEgaXMgdGhlIGVzdGltYXRlIGFuZCBzdC5lcnJvciBmb3IgY29uZGl0aW9uMi5jdDphcHRpdHVkZS5jdCAgKGNvbmRpdGlvbiBieSBhcHRpdHVkZSkNCg0KVmFsdWUgdG8gaW5mb3JtIEgxOg0KDQpXb3JrIG91dCBhIHBsYXVzaWJsZSBtYXhpbXVtIC0gYW5kIHVzZSBoYWxmIHRoaXMgYXMgdGhlIGVzdGltYXRlIC0gdXNpbmcgdGhlIG1haW4gZWZmZWN0IG9mIGFwdGl0dWRlICAoPSBhcHRpdHVkZS5jdCAtIG92ZXJhbGwgZGlmZmVyZW5jZSBpbiBwZXJmb3JtYW5jZSBvbiB0ZXN0IGZvciBvbmUgc3RlcCBpbiBhcHRpdHVkZSAod2hpY2ggaXMgc2V0IHRvIGJlIDEwJSBwb2ludHMgb24gdGhlIHBjcHQgdGVzdCkpLg0KDQppZiANCiJMVi5hcHRpdHVkZSIgaXMgdGhlIGVmZmVjdCBvZiBhcHRpdHVkZSBpbiB0aGUgTFYgY29uZGl0aW9uIA0KIkhWLmFwdGl0dWRlIiBpcyB0aGUgZWZmZWN0IG9mIGFwdGl0dWRlIGluIHRoZSBIViBjb25kaXRpb24gDQoNCkFzc3VtcHRpb25zIGZvciBwbGF1c2libGUgbWF4aW11bTogDQotIHBvc2l0aXZlIHZhbHVlIG9mIGFwdGl0dWRlIGluIEhWIA0KLSBOTyBlZmZlY3Qgb2YgYXB0aXR1ZGUgaW4gTFYgKD0wKSBbbm90ZTogbm8gcmVhc29uIHRvIHRoaW5rIHdvdWxkIGdldCBORUdBVElWRSBlZmZlY3Qgb2YgYXB0aXR1ZGUgaW4gZWl0aGVyIGNvbmRpdGlvbiwgYmFzZWQgb24gcHJldmlvdXMgZGF0YV0NCg0KdGh1czoNCmNvbmRpdGlvbiBieSBhcHRpdHVkZQ0KPSBIVi5hcHRpdHVkZS1MVi5hcHRpdHVkZQ0KPSBIVi5hcHRpdHVkZS0wDQoNCmFwdGl0dWRlDQo9IChIVi5hcHRpdHVkZStMVi5hcHRpdHVkZSkvMg0KPSAoSFYuYXB0aXR1ZGUpLzINCg0KMiAqIGFwdGl0dWRlDQo9IEhWLmFwdGl0dWRlLTANCg0KY29uZGl0aW9uIGJ5IGFwdGl0dWRlID0gIDIgKiBhcHRpdHVkZSAgDQoNCnNpbmNlIHRoaXMgaXMgYW4gZXN0aW1hdGUgb2YgdGhlIG1heGltdW0gdmFsdWUgb2YgY29uZGl0aW9uLCB3ZSBoYWx2ZSBpdCB0byBnZXQgb3VyIGVzdGltYXRlIHRvIHVzZSBhcyB0aGUgc2Qgb2YgdGhlIGhhbGYgbm9ybWFsOiAgDQoNCnNkdGhlb3J5ID0gYXB0aXR1ZGUgIA0KKHdoaWNoIGlzIGFwdGl0dWRlLmN0IGluIHRoZSBhYm92ZSBtb2RlbCkNCg0KDQpgYGB7cn0NCmNvbnRyYXN0ID0gIlBOX2FwOiBMViB2ZXJzdXMgSFYgYnkgYXB0aXR1ZGUiDQpzdGQuRXJyb3IgPSByb3VuZChzdW1tYXJ5KE5QTl9hcF9nbG1lcikkY29lZmZbImNvbmRpdGlvbjIuY3Q6YXB0aXR1ZGUuY3QiLCAiU3RkLiBFcnJvciIgXSwzKQ0KZXN0aW1hdGUgPSByb3VuZChzdW1tYXJ5KE5QTl9hcF9nbG1lcikkY29lZmZbImNvbmRpdGlvbjIuY3Q6YXB0aXR1ZGUuY3QiLCAiRXN0aW1hdGUiIF0sMykNCmgxX3NkID0gcm91bmQoc3VtbWFyeShOUE5fYXBfZ2xtZXIpJGNvZWZmWyJhcHRpdHVkZS5jdCIsICJFc3RpbWF0ZSIgXSwzKQ0KdGFpbHMgPSAxDQpQTl9hcF90YWJsZSA9IGRhdGEuZnJhbWUoY2JpbmQoY29udHJhc3QsIHN0ZC5FcnJvciwgZXN0aW1hdGUsIGgxX3NkLHRhaWxzKSkgIA0Ka2FibGUoUE5fYXBfdGFibGUpDQpgYGANCg0KDQoNCg0KIyMgV29yZCBSZXBldGl0aW9uIA0KDQpSdW4gbG1lciBtb2RlbCB3aXRoIGZhY3RvciBjb25kaXRpb24yIGxldmVscyBMViB2ZXJzdXMgSFYrSFZCLg0KQWxzbyBydW4gc2Vjb25kIG1vZGVsIHdoaWNoIGlzIHVzZWQganVzdCB0byBnZXQgb3ZlcmFsbCB2YWx1ZSBvZiBhcHRpdHVkZSBhdCBwcmUtdGVzdA0KDQpgYGB7cn0NCg0KTldSMiRjb25kaXRpb24yID0gTldSMiRjb25kaXRpb24NCk5XUjIkY29uZGl0aW9uMltOV1IyJGNvbmRpdGlvbiA9PSAiSFZCIl09ICJIViINCg0KTldSMiA9IGxpekNlbnRlcihOV1IyLCBsaXN0KCJzZXNzaW9uIiwgImFwdGl0dWRlIiwgICJ3b3JkdHlwZSIsImNvbmRpdGlvbjIiKSkNCk5XUjIgPSBsaXpDb250cmFzdHMoTldSMiwgTldSMiRjb25kaXRpb24sICJMViIpDQoNCm53X2dsbWVyX3BjcHQgPSBnbG1lcih0b25lX3Njb3JlIH4gd29yZHR5cGUuY3QgKiBjb25kaXRpb24yLmN0ICogc2Vzc2lvbi5jdCANCiAgICAgICAgICAgICAgKyBhcHRpdHVkZS5jdA0KICAgICAgICAgICAgICArIGFwdGl0dWRlLmN0IDogc2Vzc2lvbi5jdA0KICAgICAgICAgICAgICArIGFwdGl0dWRlLmN0IDogY29uZGl0aW9uMi5jdDogc2Vzc2lvbi5jdCANCiAgICAgICAgICAgICAgKyBhcHRpdHVkZS5jdCA6IGNvbmRpdGlvbjIuY3Q6IHNlc3Npb24uY3QgOiB3b3JkdHlwZS5jdA0KICAgICAgICAgICAgICArICh3b3JkdHlwZS5jdCAqIHNlc3Npb24uY3R8fHN1YmplY3QpDQogICAgICAgICAgICAgICwgZmFtaWx5PSJiaW5vbWlhbCIgLCANCiAgICAgICAgICAgICAgY29udHJvbCA9IGdsbWVyQ29udHJvbChvcHRpbWl6ZXIgPSAiYm9ieXFhIiksIA0KICAgICAgICAgICAgICBkYXRhID0gTldSMikNCmthYmxlKHJvdW5kKHN1bW1hcnkobndfZ2xtZXJfcGNwdCkkY29lZmYsMykpDQpgYGANCg0KYGBge3J9DQpOV1IzcHJlID0gc3Vic2V0KE5XUjIsIHNlc3Npb249PSAiUHJlLXRlc3QiKQ0KTldSM3ByZSA9IGxpekNlbnRlcihOV1IzcHJlLCBsaXN0KCJzZXNzaW9uIiwgImFwdGl0dWRlIiwgICJ3b3JkdHlwZSIsImNvbmRpdGlvbiIpKQ0KTldSM3ByZSA9IGxpekNvbnRyYXN0cyhOV1IzcHJlLCBOV1IzcHJlJGNvbmRpdGlvbiwgIkxWIikNCg0KbndfZ2xtZXJfcGNwdF9QUkUgPSBnbG1lcih0b25lX3Njb3JlIH4gd29yZHR5cGUuY3QgKiBjb25kaXRpb24yLmN0DQogICAgICAgICAgICAgICsgYXB0aXR1ZGUuY3QNCiAgICAgICAgICAgICAgICAgICAgICAgICAgKyBhcHRpdHVkZS5jdCA6Y29uZGl0aW9uMi5jdA0KICAgICAgICAgICAgICArIGFwdGl0dWRlLmN0IDogY29uZGl0aW9uMi5jdCA6IHdvcmR0eXBlLmN0DQogICAgICAgICAgICAgICsgKHdvcmR0eXBlLmN0IHx8c3ViamVjdCkNCiAgICAgICAgICAgICAgLCBmYW1pbHk9ImJpbm9taWFsIiAsIA0KICAgICAgICAgICAgICBjb250cm9sID0gZ2xtZXJDb250cm9sKG9wdGltaXplciA9ICJib2J5cWEiKSwgDQogICAgICAgICAgICAgIGRhdGEgPSBOV1IzcHJlKQ0Ka2FibGUocm91bmQoc3VtbWFyeShud19nbG1lcl9wY3B0X1BSRSkkY29lZmYsMykpDQoNCmBgYA0KDQoNCk1vZGVsIG9mIHRoZSBkYXRhIGlzIHRoZSBlc3RpbWF0ZSBhbmQgc3QuZXJyb3IgZm9yIHNlc3Npb24uY3Q6Y29uZGl0aW9uMi5jdDphcHRpdHVkZS5jdCAoc2Vzc2lvbiBieSBhcHRpdHVkZSBieSBjb25kaXRpb24pDQoNCkZvciBvdXIgZXN0aW1hdGUgb2YgSDEsIG5lZWQgdG8gd29yayBvdXQgYSBwbGF1c2libGUgbWF4aW11bSB2YWx1ZSBvZiBhcHRpdHVkZSB0aGF0IHdlIGV4cGVjdCBpbiBhbnkgY2VsbCAtIHdlIGdldCB0aGlzIGZyb20gdGhlIHNjYWxlLiANCg0KV2UgZG8gdGhpcyBhcyBmb2xsb3dzOg0KbWF4aW11bSBwb3NzaWJsZSB2YWx1ZSBvZiBhcHRpdHVkZSAobWF4X2FwdGl0dWRlKSwgd29ya2luZyBmcm9tIHRoZSBzY2FsZTogQXNzdW1lIHBhcnRpY2lwYW50IHdpdGggbWluaW1hbCB2YWx1ZSBvZiBhcHRpdHVkZSBpcyBhdCBjaGFuY2UgaW4gcHJvZHVjaW5nIG9uZSBvZiB0aGUgZm91ciB0b25lcyAoMS80IGluIGxvZyBvZGRzKSBhbmQgcGFydGljaXBhbnQgd2l0aCBtYXhpbXVtIHZhbHVlIG9mIGFwdGl0dWRlIGlzIGF0IGNlaWxpbmcgaW4gcHJvZHVjaW5nIHRoZSBjb3JyZWN0IHRvbmUgLSBpLmUuIDcyLzcyIChzaW5jZSA3MiB0ZXN0IGl0ZW1zKSAtIGJ1dCBzaW5jZSB3ZSBjb21wdXRlIHRoaXMgaW4gbG9nb2RkcyB1c2UgNzEvNzIuIFRoZW4gdG8gd29yayBvdXQgdGhlIGNoYW5nZSBpbiB0b25lLXNjb3JlIGZvciBhIDEtc3RlcCBjaGFuZ2UgaW4gYXB0aXR1ZGUgd2UgbmVlZCB0byBkaXZpZGUgaXQgYnkgdGhlIGxlbmd0aCBvZiB0aGUgYXB0aXR1ZGUgcHJlZGljdG9yIChtYXggdmFsdWUgb2YgYXB0aXR1ZGUgZm9yIGFueSBwYXJ0aWNpcGFudCAtIG1pbmltYWwgdmFsdWUgb2YgYXB0aXR1ZGUgZm9yIGFueSBwYXJ0aWNpcGFudCkuDQoNCg0KYGBge3J9DQpsZW5ndGggPSBtYXgoTldSMiRhcHRpdHVkZS5jdCkgLSBtaW4oTldSMiRhcHRpdHVkZS5jdCkNCmxlbmd0aA0KbWF4X2FwdGl0dWRlX3dyID0gKGxvZ29kZHMoNzEvNzIpLSBsb2dvZGRzKDEvNCkpL2xlbmd0aA0KbWF4X2FwdGl0dWRlX3dyDQpgYGANCg0KDQoNCldlIHVzZSB0aGlzIG1heGltdW0gdmFsdWUgYW5kIHRoZSBBQ1RVQUwgdmFsdWUgb2YgYXB0aXR1ZGUgYXQgcHJlLXRlc3QgdG8gd29yayBvdXQgYSBtYXhpbXVtIHZhbHVlIGZvciB0aGUgaW50ZXJhY3Rpb24gb2YgaW50ZXJlc3QsIGFzIGZvbGxvd3M6ICANCg0KIkxWLlBSRS5hcHRpdHVkZSIgaXMgdGhlIGVmZmVjdCBvZiBhcHRpdHVkZSBhdCBwcmUtdGVzdCBpbiBMVg0KIkxWLlBPU1QuYXB0aXR1ZGUiIGlzIHRoZSBlZmZlY3Qgb2YgYXB0aXR1ZGUgYXQgcG9zdC10ZXN0IGluIExWDQoiSFYsUFJFLmFwdGl0dWRlIiBpcyB0aGUgZWZmZWN0IG9mIGFwdGl0dWRlIGF0IHByZS10ZXN0IGluIEhWDQoiSFYuUE9TVC5hcHRpdHVkZSIgaXMgdGhlIGVmZmVjdCBvZiBhcHRpdHVkZSBhdCBwb3N0LXRlc3QgaW4gSFYNCg0KUFJFLmFwdGl0dWRlIGlzIHRoZSBhdmVyYWdlIHZhbHVlIG9mIGFwdGl0dWRlIGF0IHByZS10ZXN0DQoNCm1heF9hcHRpdHVkZSBpcyBtYXhpbXVtIHZhbHVlIG9mIGFwdGl0dWRlIChhcyBjb21wdXRlZCBhYm92ZSkNCg0KQXNzdW1wdGlvbnMgZm9yIHBsYXVzaWJsZSBtYXhpbXVtOiANCi0gYm90aCBjb25kaXRpb25zIGhhdmUgdGhlIHNhbWUgdmFsdWUgb2YgYXB0aXR1ZGUgYXQgcHJlLXRlc3QgKGVzdGltYXRlZCBhcyBQUkUuYXB0aXR1ZGUpDQotIGluIExWIGNvbmRpdGlvbiwgZWZmZWN0IG9mIGFwdGl0dWRlIGlzIHRoZSBzYW1lIGF0IHByZSBhbmQgcG9zdCB0ZXN0DQotIGluIEhWIGNvbmRpdGlvbiwgZWZmZWN0IG9mIGFwdGl0dWRlIGlzIGluY3JlYXNlZCB0byB0aGUgbWF4aW11bSBhbW91bnQgYXQgcG9zdCB0ZXN0ICg9bWF4X2FwdGl0dWRlICkNCg0KdGh1czoNCnNlc3Npb24gYnkgYXB0aXR1ZGUgYnkgY29uZGl0aW9uDQo9IChIVi5QT1NULmFwdGl0dWRlLSBIVi5QUkUuYXB0aXR1ZGUpLShMVi5QT1NULmFwdGl0dWRlLSBMVi5QUkUuYXB0aXR1ZGUpDQo9IChtYXhfYXB0aXR1ZGUtIFBSRS5hcHRpdHVkZSktKFBSRS5hcHRpdHVkZS0gUFJFLmFwdGl0dWRlKQ0KPSBtYXhfYXB0aXR1ZGUtIFBSRS5hcHRpdHVkZQ0KDQpzaW5jZSB0aGlzIGlzIGFuIGVzdGltYXRlIG9mIHRoZSBtYXhpbXVtIHZhbHVlIG9mIGNvbmRpdGlvbiwgd2UgaGFsdmUgaXQgdG8gZ2V0IG91ciBlc3RpbWF0ZSB0byB1c2UgYXMgdGhlIHNkIG9mIHRoZSBoYWxmIG5vcm1hbDogIA0Kc2R0aGVvcnkgPSAobWF4X2FwdGl0dWRlIC0gUFJFLmFwdGlkdWUpLzINCg0KDQpgYGB7cn0NCmNvbnRyYXN0ID0gICAiV29yZCBSZXBldGl0aW9uLCBUb25lIHNjb3JlOiBhcHRpdHVkZSBieSBzZXNzaW9uIGJ5IExWIHZlcnN1cyBIViINCnN0ZC5FcnJvciA9IHJvdW5kKHN1bW1hcnkobndfZ2xtZXJfcGNwdCkkY29lZmZbImNvbmRpdGlvbjIuY3Q6c2Vzc2lvbi5jdDphcHRpdHVkZS5jdCIsICJTdGQuIEVycm9yIl0sMykNCg0KZXN0aW1hdGUgPSByb3VuZChzdW1tYXJ5KG53X2dsbWVyX3BjcHQpJGNvZWZmWyJjb25kaXRpb24yLmN0OnNlc3Npb24uY3Q6YXB0aXR1ZGUuY3QiLCJFc3RpbWF0ZSIgXSwzKQ0KDQpoMV9zZCA9ICByb3VuZCgobWF4X2FwdGl0dWRlX3dyIC0gc3VtbWFyeShud19nbG1lcl9wY3B0X1BSRSkkY29lZmZbImFwdGl0dWRlLmN0IiwgIkVzdGltYXRlIiBdKS8yLDMpDQogICAgICAgICAgICAgIA0KdGFpbHMgPSAxDQpXUl9hcF90YWJsZSA9IGRhdGEuZnJhbWUoY2JpbmQoY29udHJhc3QsIHN0ZC5FcnJvciwgZXN0aW1hdGUsIGgxX3NkLCB0YWlscykpICANCmthYmxlKFdSX2FwX3RhYmxlKQ0KYGBgDQoNCg0KDQoNCg0KDQoNCg0KDQoNCg0KDQojIyAzIEludGVydmFsIG9kZGl0eQ0KUnVuIGZhY3RvciBjb25kaXRpb24yIGxldmVscyBMViB2ZXJzdXMgSFYrSFZCDQpgYGB7cn0NCmRpczIkY29uZGl0aW9uMj0gZGlzMiRjb25kaXRpb24NCmRpczIkY29uZGl0aW9uMltkaXMyJGNvbmRpdGlvbj09IkhWQiJdPSAiSFYiDQpkaXMyID0gbGl6Q2VudGVyKGRpczIsIGxpc3QoInNlc3Npb24iLCAiY29uZGl0aW9uMiIpKQ0KDQpkX2dsbWVyX3BjcHQgPSBnbG1lcihzY29yZSB+IHNlc3Npb24uY3QgKiB3b3JkdHlwZS5jdCAqIGNvbmRpdGlvbjIuY3QNCiAgICAgICAgICAgICAgICAgICAgICsgKE5ldXRyYWxfVkVSU1VTX0Vhc3kgKyBOZXV0cmFsX1ZFUlNVU19IYXJkKTogc2Vzc2lvbi5jdCANCiAgICAgICAgICAgICAgICAgICAgICsgKE5ldXRyYWxfVkVSU1VTX0Vhc3kgKyBOZXV0cmFsX1ZFUlNVU19IYXJkKSANCiAgICAgICAgICAgICAgICAgICAgICsgYXB0aXR1ZGUuY3QNCiAgICAgICAgICAgICAgICAgICAgICsgYXB0aXR1ZGUuY3Q6IHNlc3Npb24uY3QNCiAgICAgICAgICAgICAgICAgICAgICsgYXB0aXR1ZGUuY3QgOmNvbmRpdGlvbjIuY3QgOiBzZXNzaW9uLmN0DQogICAgICAgICAgICAgICAgICAgICArIGFwdGl0dWRlLmN0IDogY29uZGl0aW9uMi5jdCA6IHNlc3Npb24uY3QgOiB3b3JkdHlwZS5jdA0KICAgICAgICAgICAgICAgICAgICAgKyAoc2Vzc2lvbi5jdCAqIHdvcmR0eXBlLmN0fHxzdWJqZWN0KQ0KICAgICAgICAgICAgICAgICAgICAgLCBmYW1pbHkgPSAiYmlub21pYWwiLCANCiAgICAgICAgICAgICAgICAgICAgIGNvbnRyb2wgPSBnbG1lckNvbnRyb2wob3B0aW1pemVyID0gImJvYnlxYSIpLCANCiAgICAgICAgICAgICAgICAgICAgIGRhdGEgPSBkaXMyKQ0Ka2FibGUocm91bmQoc3VtbWFyeShkX2dsbWVyX3BjcHQpJGNvZWZmLDMpKQ0KYGBgDQoNCmBgYHtyfQ0KZGlzM3ByZSA9IHN1YnNldChkaXMyLCBzZXNzaW9uID09IGMoIlByZS10ZXN0IikpDQpkaXMzcHJlID0gbGl6Q2VudGVyKGRpczNwcmUsIGxpc3QoInNlc3Npb24iLCAiY29uZGl0aW9uMiIpKQ0KDQpkX2dsbWVyX3BjcHRfUFJFID0gZ2xtZXIoc2NvcmUgfndvcmR0eXBlLmN0ICogY29uZGl0aW9uMi5jdA0KICAgICAgICAgICAgICAgICAgICAgKyAoTmV1dHJhbF9WRVJTVVNfRWFzeSArIE5ldXRyYWxfVkVSU1VTX0hhcmQpIA0KICAgICAgICAgICAgICAgICAgICAgKyBhcHRpdHVkZS5jdA0KICAgICAgICAgICAgICAgICAgICAgKyBhcHRpdHVkZS5jdCA6IGNvbmRpdGlvbjIuY3QNCiAgICAgICAgICAgICAgICAgICAgICsgYXB0aXR1ZGUuY3QgOiBjb25kaXRpb24yLmN0IDogd29yZHR5cGUuY3QNCiAgICAgICAgICAgICAgICAgICAgICsgKHdvcmR0eXBlLmN0fHxzdWJqZWN0KQ0KICAgICAgICAgICAgICAgICAgICAgLCBmYW1pbHkgPSAiYmlub21pYWwiLCANCiAgICAgICAgICAgICAgICAgICAgIGNvbnRyb2wgPSBnbG1lckNvbnRyb2wob3B0aW1pemVyID0gImJvYnlxYSIpLCANCiAgICAgICAgICAgICAgICAgICAgIGRhdGEgPSBkaXMzcHJlKQ0KDQoNCmthYmxlKHJvdW5kKHN1bW1hcnkoZF9nbG1lcl9wY3B0X1BSRSkkY29lZmYsMykpDQoNCg0KYGBgDQoNCk1vZGVsIG9mIHRoZSBkYXRhIGlzIHRoZSBlc3RpbWF0ZSBhbmQgc3QuZXJyb3IgZm9yIHNlc3Npb24uY3Q6Y29uZGl0aW9uMi5jdDphcHRpdHVkZS5jdCAoc2Vzc2lvbiBieSBhcHRpdHVkZSBieSBjb25kaXRpb24pDQoNCkZvciBvdXIgZXN0aW1hdGUgb2YgSDEsIG5lZWQgdG8gd29yayBvdXQgYSBwbGF1c2libGUgbWF4aW11bSB2YWx1ZSBvZiBhcHRpdHVkZSB0aGF0IHdlIGV4cGVjdCBpbiBhbnkgY2VsbCB3ZSBnZXQgdGhpcyBmcm9tIHRoZSBzY2FsZS4gDQoNCldlIGRvIHRoaXMgYXMgZm9sbG93czoNCm1heGltdW0gcG9zc2libGUgdmFsdWUgb2YgYXB0aXR1ZGUgKG1heF9hcHRpdHVkZSksIHdvcmtpbmcgZnJvbSB0aGUgc2NhbGU6IEFzc3VtZSBwYXJ0aWNpcGFudCB3aXRoIG1pbmltYWwgdmFsdWUgb2YgYXB0aXR1ZGUgaXMgYXQgY2hhbmNlIGluIHBpY2tpbmcgb2RkIG9uZSBvdXQgKDEvMyBpbiBsb2cgb2RkcykgYW5kIHBhcnRpY2lwYW50IHdpdGggbWF4aW11bSB2YWx1ZSBvZiBhcHRpdHVkZSBpcyBhdCBjZWlsaW5nIGluIHByb2R1Y2luZyB0aGUgY29ycmVjdCB0b25lIC0gaS5lLiA3Mi83MiAoc2luY2UgNzIgdGVzdCBpdGVtcykgLSBidXQgc2luY2Ugd2UgY29tcHV0ZSB0aGlzIGluIGxvZ29kZHMgdXNlIDcxLzcyLiBUaGVuIHRvIHdvcmsgb3V0IHRoZSBjaGFuZ2UgaW4gdG9uZS1zY29yZSBmb3IgYSAxLXN0ZXAgY2hhbmdlIGluIGFwdGl0dWRlIHdlIG5lZWQgdG8gZGl2aWRlIGl0IGJ5IHRoZSBsZW5ndGggb2YgdGhlIGFwdGl0dWRlIHByZWRpY3RvciAobWF4IHZhbHVlIG9mIGFwdGl0dWRlIGZvciBhbnkgcGFydGljaXBhbnQgLSBtaW5pbWFsIHZhbHVlIG9mIGFwdGl0dWRlIGZvciBhbnkgcGFydGljaXBhbnQpLg0KDQoNCg0KYGBge3J9DQpsZW5ndGggPSBtYXgoZGlzMiRhcHRpdHVkZS5jdCkgLSBtaW4oZGlzMiRhcHRpdHVkZS5jdCkNCmxlbmd0aA0KbWF4X2FwdGl0dWRlX2QgPSAobG9nb2Rkcyg3MS83MiktIGxvZ29kZHMoMS8zKSkvbGVuZ3RoDQptYXhfYXB0aXR1ZGVfZA0KYGBgDQoNCg0KV2UgdXNlIHRoaXMgbWF4aW11bSB2YWx1ZSBhbmQgdGhlIEFDVFVBTCB2YWx1ZSBvZiBhcHRpdHVkZSBhdCBwcmUtdGVzdCB0byB3b3JrIG91dCBhIG1heGltdW0gdmFsdWUgZm9yIHRoZSBpbnRlcmFjdGlvbiBvZiBpbnRlcmVzdCwgYXMgZm9sbG93czogIA0KDQoiTFYuUFJFLmFwdGl0dWRlIiBpcyB0aGUgZWZmZWN0IG9mIGFwdGl0dWRlIGF0IHByZS10ZXN0IGluIExWDQoiTFYuUE9TVC5hcHRpdHVkZSIgaXMgdGhlIGVmZmVjdCBvZiBhcHRpdHVkZSBhdCBwb3N0LXRlc3QgaW4gTFYNCiJIVixQUkUuYXB0aXR1ZGUiIGlzIHRoZSBlZmZlY3Qgb2YgYXB0aXR1ZGUgYXQgcHJlLXRlc3QgaW4gSFYNCiJIVi5QT1NULmFwdGl0dWRlIiBpcyB0aGUgZWZmZWN0IG9mIGFwdGl0dWRlIGF0IHBvc3QtdGVzdCBpbiBIVg0KDQpQUkUuYXB0aXR1ZGUgaXMgdGhlIGF2ZXJhZ2UgdmFsdWUgb2YgYXB0aXR1ZGUgYXQgcHJlLXRlc3QNCg0KbWF4X2FwdGl0dWRlIGlzIG1heGltdW0gdmFsdWUgb2YgYXB0aXR1ZGUgKGFzIGNvbXB1dGVkIGFib3ZlKQ0KDQpBc3N1bXB0aW9ucyBmb3IgcGxhdXNpYmxlIG1heGltdW06IA0KLSBib3RoIGNvbmRpdGlvbnMgaGF2ZSB0aGUgc2FtZSB2YWx1ZSBvZiBhcHRpdHVkZSBhdCBwcmUtdGVzdCAoZXN0aW1hdGVkIGFzIFBSRS5hcHRpdHVkZSkNCi0gaW4gTFYgY29uZGl0aW9uLCBlZmZlY3Qgb2YgYXB0aXR1ZGUgaXMgdGhlIHNhbWUgYXQgcHJlIGFuZCBwb3N0IHRlc3QNCi0gaW4gSFYgY29uZGl0aW9uLCBlZmZlY3Qgb2YgYXB0aXR1ZGUgaXMgaW5jcmVhc2VkIHRvIHRoZSBtYXhpbXVtIGFtb3VudCBhdCBwb3N0IHRlc3QgKD1tYXhfYXB0aXR1ZGUgKQ0KDQp0aHVzOg0Kc2Vzc2lvbiBieSBhcHRpdHVkZSBieSBjb25kaXRpb24NCj0gKEhWLlBPU1QuYXB0aXR1ZGUtIEhWLlBSRS5hcHRpdHVkZSktKExWLlBPU1QuYXB0aXR1ZGUtIExWLlBSRS5hcHRpdHVkZSkNCj0gKG1heF9hcHRpdHVkZS0gUFJFLmFwdGl0dWRlKS0oUFJFLmFwdGl0dWRlLSBQUkUuYXB0aXR1ZGUpDQo9IG1heF9hcHRpdHVkZS0gUFJFLmFwdGl0dWRlDQoNCnNpbmNlIHRoaXMgaXMgYW4gZXN0aW1hdGUgb2YgdGhlIG1heGltdW0gdmFsdWUgb2YgY29uZGl0aW9uLCB3ZSBoYWx2ZSBpdCB0byBnZXQgb3VyIGVzdGltYXRlIHRvIHVzZSBhcyB0aGUgc2Qgb2YgdGhlIGhhbGYgbm9ybWFsOiAgDQpzZHRoZW9yeSA9IChtYXhfYXB0aXR1ZGUgLSBQUkUuYXB0aWR1ZSkvMg0KDQoNCg0KYGBge3J9DQpjb250cmFzdCA9ICAgICAgICIzIEludC4gT2RkLiwgVG9uZSBzY29yZTogYXB0aXR1ZGUgYnkgc2Vzc2lvbiBieSBMViB2ZXJzdXMgSFYiDQoNCnN0ZC5FcnJvciA9IHJvdW5kKHN1bW1hcnkoZF9nbG1lcl9wY3B0KSRjb2VmZlsic2Vzc2lvbi5jdDpjb25kaXRpb24yLmN0OmFwdGl0dWRlLmN0IiwgIlN0ZC4gRXJyb3IiIF0sMykNCg0KZXN0aW1hdGUgPSAgcm91bmQoc3VtbWFyeShkX2dsbWVyX3BjcHQpJGNvZWZmWyJzZXNzaW9uLmN0OmNvbmRpdGlvbjIuY3Q6YXB0aXR1ZGUuY3QiLCJFc3RpbWF0ZSIgXSwzKQ0KaDFfc2QgPSAgcm91bmQoKG1heF9hcHRpdHVkZV9kIC0gc3VtbWFyeShkX2dsbWVyX3BjcHRfUFJFKSRjb2VmZlsiYXB0aXR1ZGUuY3QiLCAiRXN0aW1hdGUiIF0pLzIsMykNCnRhaWxzID0gMQ0KZGlzY3JpbV9hcF90YWJsZSA9IGRhdGEuZnJhbWUoY2JpbmQoY29udHJhc3QsIHN0ZC5FcnJvciwgZXN0aW1hdGUsIGgxX3NkLCB0YWlscykpICANCmthYmxlKGRpc2NyaW1fYXBfdGFibGUpDQpgYGANCg0KIyMgdHJhaW5pbmcgZGF0YQ0KDQpgYGB7cn0NCg0KdHJhaW4yID0gbGl6Q29udHJhc3RzKHRyYWluMiwgdHJhaW4yJGNvbmRpdGlvbiwgIkxWIikNCnRyYWluMiA9IGxpekNlbnRlcih0cmFpbjIsIGxpc3QoInNlc3Npb24iLCJhcHRpdHVkZSIpKQ0KdHJhaW4yLm5vSFZCID0gbGl6Q2VudGVyKHN1YnNldCh0cmFpbjIsIGNvbmRpdGlvbiAhPSAiSFZCIiksIGxpc3QoInNlc3Npb24iLCAiY29uZGl0aW9uIiwgImFwdGl0dWRlIikpDQp0cmFpbjIubm9IViA9IGxpekNlbnRlcihzdWJzZXQodHJhaW4yLCBjb25kaXRpb24gIT0gIkhWIiksIGxpc3QoInNlc3Npb24iLCAiY29uZGl0aW9uIiwgImFwdGl0dWRlIikpDQoNCnRfZ2xtZXJfcGNwdCA9IGdsbWVyKHNjb3JlIH4gKExWX1ZFUlNVU19IViArIExWX1ZFUlNVU19IVkIpICogYXB0aXR1ZGUuY3QgDQogICAgICAgICAgICAgICAgKyAoMXxzdWJqZWN0KQ0KICAgICAgICAgICAgICAsIGZhbWlseSA9ICJiaW5vbWlhbCIsIA0KICAgICAgICAgICAgICBjb250cm9sID0gZ2xtZXJDb250cm9sKG9wdGltaXplciA9ICJib2J5cWEiKSwgDQogICAgICAgICAgICAgIGRhdGEgPSB0cmFpbjIpDQoNCmthYmxlKHJvdW5kKHN1bW1hcnkodF9nbG1lcl9wY3B0ICkkY29lZmYsMykpDQoNCmBgYA0KDQoNCk1vZGVsIG9mIHRoZSBkYXRhIGlzIHRoZSBlc3RpbWF0ZSBhbmQgc3QuZXJyb3IgZm9yIGNvbmRpdGlvbjIuY3Q6YXB0aXR1ZGUuY3QgKGNvbmRpdGlvbiBieSBhcHRpdHVkZSkNCg0KVmFsdWUgdG8gaW5mb3JtIEgxICh0aGlzIGlzIHRoZSBzYW1lIGFzIGZvciBQSSBhYm92ZSk6DQoNCldvcmsgb3V0IGEgcGxhdXNpYmxlIG1heGltdW0gLSBhbmQgdXNlIGhhbGYgdGhpcyBhcyB0aGUgZXN0aW1hdGUgLSB1c2luZyB0aGUgbWFpbiBlZmZlY3Qgb2YgYXB0aXR1ZGUgICg9IGFwdGl0dWRlLmN0IC0gb3ZlcmFsbCBkaWZmZXJlbmNlIGluIHBlcmZvcm1hbmNlIG9uIHRlc3QgZm9yIG9uZSBzdGVwIGluIGFwdGl0dWRlICh3aGljaCBpcyBzZXQgdG8gYmUgMTAlIHBvaW50cyBvbiB0aGUgcGNwdCB0ZXN0KSkuDQoNCmlmIA0KIkxWLmFwdGl0dWRlIiBpcyB0aGUgZWZmZWN0IG9mIGFwdGl0dWRlIGluIHRoZSBMViBjb25kaXRpb24gDQoiSFYuYXB0aXR1ZGUiIGlzIHRoZSBlZmZlY3Qgb2YgYXB0aXR1ZGUgaW4gdGhlIEhWIGNvbmRpdGlvbiANCg0KQXNzdW1wdGlvbnMgZm9yIHBsYXVzaWJsZSBtYXhpbXVtOiANCi0gcG9zaXRpdmUgdmFsdWUgb2YgYXB0aXR1ZGUgaW4gSFYgDQotIE5PIGVmZmVjdCBvZiBhcHRpdHVkZSBpbiBMViAoPTApIFtub3RlOiBubyByZWFzb24gdG8gdGhpbmsgd291bGQgZ2V0IE5FR0FUSVZFIGVmZmVjdCBvZiBhcHRpdHVkZSBpbiBlaXRoZXIgY29uZGl0aW9uLCBiYXNlZCBvbiBwcmV2aW91cyBkYXRhXQ0KDQp0aHVzOg0KY29uZGl0aW9uIGJ5IGFwdGl0dWRlDQo9IEhWLmFwdGl0dWRlLUxWLmFwdGl0dWRlDQo9IEhWLmFwdGl0dWRlLTANCg0KYXB0aXR1ZGUNCj0gKEhWLmFwdGl0dWRlK0xWLmFwdGl0dWRlKS8yDQo9IChIVi5hcHRpdHVkZSkvMg0KDQoyICogYXB0aXR1ZGUNCj0gSFYuYXB0aXR1ZGUNCg0KY29uZGl0aW9uIGJ5IGFwdGl0dWRlID0gIDIgKiBhcHRpdHVkZSAgDQoNCnNpbmNlIHRoaXMgaXMgYW4gZXN0aW1hdGUgb2YgdGhlIG1heGltdW0gdmFsdWUgb2YgY29uZGl0aW9uLCB3ZSBoYWx2ZSBpdCB0byBnZXQgb3VyIGVzdGltYXRlIHRvIHVzZSBhcyB0aGUgc2Qgb2YgdGhlIGhhbGYgbm9ybWFsOiAgDQoNCnNkdGhlb3J5ID0gYXB0aXR1ZGUgIA0KKHdoaWNoIGlzIGFwdGl0dWRlLmN0IGluIHRoZSBhYm92ZSBtb2RlbCkNCg0KDQpgYGB7cn0NCmNvbnRyYXN0ID0gICBjKCJUcmFpbmluZzogYXB0aXR1ZGUgYnkgTFYgdmVyc3VzIEhWLXVuYmxvY2tlZCIsDQogICAgICAgICAgICAgICAiVHJhaW5pbmc6IGFwdGl0dWRlIGJ5IExWIHZlcnN1cyBIVi1ibG9ja2VkIikNCg0Kc3RkLkVycm9yID0gYyhyb3VuZChzdW1tYXJ5KHRfZ2xtZXJfcGNwdCkkY29lZmZbIkxWX1ZFUlNVU19IVjphcHRpdHVkZS5jdCIsICJTdGQuIEVycm9yIl0sMyksDQogICAgICAgcm91bmQoc3VtbWFyeSh0X2dsbWVyX3BjcHQpJGNvZWZmWyJMVl9WRVJTVVNfSFZCOmFwdGl0dWRlLmN0IiwgIlN0ZC4gRXJyb3IiXSwzKSkgICAgICANCiAgICAgICAgICAgICAgDQplc3RpbWF0ZSA9IGMocm91bmQoc3VtbWFyeSh0X2dsbWVyX3BjcHQpJGNvZWZmWyJMVl9WRVJTVVNfSFY6YXB0aXR1ZGUuY3QiLCAiRXN0aW1hdGUiXSwzKSwNCiAgICAgICByb3VuZChzdW1tYXJ5KHRfZ2xtZXJfcGNwdCkkY29lZmZbIkxWX1ZFUlNVU19IVkI6YXB0aXR1ZGUuY3QiLCAiRXN0aW1hdGUiXSwzKSkgIA0KDQpoMV9zZCA9ICByb3VuZChyZXAoc3VtbWFyeSh0X2dsbWVyX3BjcHQpJGNvZWZmWyJhcHRpdHVkZS5jdCIsIkVzdGltYXRlIiBdLDIpLDMpDQogICAgICAgICAgICAgIA0KdGFpbHMgPSBjKDEsMSkNCnRyYWluX2FwX3RhYmxlID0gZGF0YS5mcmFtZShjYmluZChjb250cmFzdCwgc3RkLkVycm9yLCBlc3RpbWF0ZSwgaDFfc2QsIHRhaWxzKSkgDQprYWJsZSh0cmFpbl9hcF90YWJsZSkNCmBgYA0KDQoNCiMjIGNvbXB1dGUgYkZzIGFuZCByYW5nZXMNCg0KDQpgYGB7cn0NCmRmPSByYmluZChQSV9hcF90YWJsZSwgUE5fYXBfdGFibGUsIGRpc2NyaW1fYXBfdGFibGUsIFdSX2FwX3RhYmxlLCB0cmFpbl9hcF90YWJsZSkNCmRmJHN0ZC5FcnJvciA9IGFzLm51bWVyaWMoYXMuY2hhcmFjdGVyKGRmJHN0ZC5FcnJvcikpDQpkZiRlc3RpbWF0ZSA9IGFzLm51bWVyaWMoYXMuY2hhcmFjdGVyKGRmJGVzdGltYXRlKSkNCmRmJGgxX3NkID0gYXMubnVtZXJpYyhhcy5jaGFyYWN0ZXIoZGYkaDFfc2QpKQ0KZGYkdGFpbHMgPSBhcy5udW1lcmljKGFzLmNoYXJhY3RlcihkZiR0YWlscykpDQpkZiRoMV9tb3RpdmF0aW9uID0gIiINCg0KZGYyID0gQmZfdGFibGUoZGYpDQoNCiNrYWJsZShkZjIpDQoNCiNrYWJsZShhZGRCZl9yYW5nZXMyKGRmMikpDQoNCiMgbm90ZSBvdXIgcmFuZ2Ugb2YgdmFsdWVzIGZvciBCZiANCnNkdGhlb3J5cmFuZ2UgPSBzZXEoIGZyb20gPSAwLCB0byA9IDUgLCBsZW5ndGgub3V0ID0gMTAwKQ0KDQphZGRCZl9yYW5nZXMoZGYyLHNkdGhlb3J5cmFuZ2UpICU+JQ0KICBrYWJsZSgpICU+JQ0KICBrYWJsZV9zdHlsaW5nKCkNCg0KYGBgDQoNCg0KIyMgZXN0aW1hdGUgc2FtcGxlIGluY3JlYXNlDQoNCg0KQWJvdmUgd2Ugc2VlbiB0aGF0IHdlIGRvIG5vdCBoYXZlIHN1YnN0YW50aWFsIGV2aWRlbmNlIGZvciB0aGUgbnVsbCAob3IgSDEpIHdpdGggb3VyIGN1cnJlbnQgc2FtcGxlLiBXZSB1c2UgYSBmdW5jdGlvbiB0byBzZWUgaG93IG1hbnkgcGFydGljaXBhbnRzIHdlIG1pZ2h0IG5lZWQgdG8gc2VlIHN1YnN0YW5hdGlhbCBldmlkZW5jZSBmb3IgdGhlIG51bGwsIGFzc3VtaW5nIHRoYXQgdGhlIHN0YW5kYXJkIGVycm9yIGRlY3JlYXNlcyBwcm8tcG9ydGlvbmFsIHRvIHJvdXRlIE4NCg0KYGBge3J9DQoNCiMgbm90ZSBsaW5lczUtNiBvZiBkZiBhcmUgdGhlIHRyYWluaW5nIGRhdGEgd2hlcmUgd2UgYXJlIGxvb2tpbmcgYXQgY29udHJhc3RzIG5vdCB1c2luZyB0aGUgc2FtZSBzYW1wbGUsIHNvIHRoZSBOIGlzIGxlc3MgKDQwIHJhdGhlciB0aGFuIDYwKQ0KYWRkQmZfcG93ZXJjYWxjKGRmWzE6NCxdLCA2MCwgNTAwKSU+JQ0KICBrYWJsZSgpICU+JQ0KICBrYWJsZV9zdHlsaW5nKCkNCg0KYWRkQmZfcG93ZXJjYWxjKGRmWzU6NixdLCA0MCwgNTAwKSU+JQ0KICBrYWJsZSgpICU+JQ0KICBrYWJsZV9zdHlsaW5nKCkNCg0KDQpgYGA=
