# Supplementary material for: The effects of high versus low talker variability and individual aptitude on phonetic training of Mandarin lexical tones
Source: PeerJ. 2019 Aug 9;7:e7191. doi: 10.7717/peerj.7191 (PMC6690337; doi:10.7717/peerj.7191)
Supplement: Supplemental Information 6 [file peerj-07-7191-s006.docx]

This is the document explaining the data structure in each csv file.

## PCPT.csv

This is the Pitch-Contour-Perception-Test.

Version: This variable represents which counterbalancing version was employed in that trial, details can be seen from Table 3.

Subject: The assigned participant number

Condition: The variability condition the participant is assigned to. 0 = Low variability; 1 = High variability; 2 = High variability blocked

Session: Whether this trial is in Pre-training test (0) or Post-training test (1)

Vowel: The vowel used in the current trial.

Tone: The tone pronounced in this trial

Speaker: The speaker produced this trial. (e.g. f1 = female speaker 1)

Choice: The tone selected by participants.

Accuracy: Whether the participant answers this trial correctly (1 = correct, 0 = incorrect).

RT: Participants’ response time.

## CSTC-curve.csv

This is the Categorisation of Synthesized Tonal Continua task.

Subject: The assigned participant number

Condition: The variability condition the participant is assigned to. 0 = Low variability; 1 = High variability; 2 = High variability blocked

Session: Whether this trial is in Pre-training test (0) or Post-training test (1)

Step: The specific sound used in this trial. Step 1-3 are synthesized from tone 2 and step 4-6 are synthesized from tone 3 (see section 2.3.1.1 for details)

Category: The choice made by participants (0.000001 = tone 2 is selected; 0.999999 = tone 3 is selected).

## Discrimination.csv

This is the Three Interval Oddity task.

Version: This variable represents which counterbalancing version was employed in that trial, details can be seen from Table 3.

Subject: The assigned participant number

Condition: The variability condition the participant is assigned to. 0 = Low variability; 1 = High variability; 2 = High variability blocked

Session: Whether this trial is in Pre-training test (0) or Post-training test (1)

Same 1: The first same sound in this trial. Pinyin, tone, and speaker are provided in the file name (e.g. xiang_t4_fn1).

Same 2: The second same sound in this trial.

Diff: The different sound in this trial (the odd one out/target sound). Note also it is also arranged in the csv file in this order, the actual order is scrambled in the task.

Wordtype: whether this word is used in training (oldword) or not (newwrod).

Voicetype: The speaker combination in this trial. (i)fff: “Neutral” - all three words were spoken by female speakers (ii) ffm: “Easy” - the “different” word was spoken by the one male speaker (iii) fmf: “Hard” - the “different” word was spoken by one of the two female speakers.

Tones: The tone contrast used in this trial. The second tone represents the target tone (e.g. t4t1, t1 is the odd one out tone).

Result: The choice made by participants. chosediff: participant chose the odd one out word. chosesame: participant chose one of the same words.

Score: Whether the participant answers this trial correctly (1 = correct, 0 = incorrect).

RT: Participants’ response time.

## Training.csv

This is the Training task.

Version: This variable represents which counterbalancing version was employed in that trial, details can be seen from Table 3.

Subject: The assigned participant number

Condition: The variability condition the participant is assigned to. 0 = Low variability; 1 = High variability; 2 = High variability blocked

Session: Training sessions 1-6.

Sound: The sound stimuli pronounced in this trial.

Pic1: The first picture presented in this trial.

Pic2: The second picture presented in this trial.

CorrectAnswer: The correct picture that should be selected.

Contrast: The tone contrast and the speaker involved in the trial, the first tone is always the target tone (e.g. T2-3_tv3 means this trial uses a tone 2 versus tone 3 contrast produced by speaker 2, the target tone is tone 2). For counterbalancing of speakers in training, see Table 3.

Choice: Which picture participants clicked on the screen.

Result: Whether participants answered this trial correctly.

Score: Whether the participant answers this trial correctly (1 = correct, 0 = incorrect).

RT: Participants’ response time.

## Picture Identification.csv

This is the Picture Identification task.

Version: This variable represents which counterbalancing version was employed in that trial, details can be seen from Table 3.

Subject: The assigned participant number

Condition: The variability condition the participant is assigned to. 0 = Low variability; 1 = High variability; 2 = High variability blocked

Sound: The sound stimuli pronounced in this trial.

Pic1: The first picture presented in this trial.

Pic2: The second picture presented in this trial.

correct answer: The correct picture that should be selected.

Contrast: The tone contrast and the speaker involved in the trial, the first tone is always the target tone (e.g. T2-3_tv3 means this trial uses a tone 2 versus tone 3 contrast produced by speaker 2, the target tone is tone 2). For counterbalancing of speakers in training, see Table 3. \

Voicetype: Speaker used in this trial. tv = Trained speaker; nv = New speaker.

Choice: Which picture participants clicked on the screen.

Result: Whether participants answered this trial correctly.

Score: Whether the participant answers this trial correctly (1 = correct, 0 = incorrect).

RT: Participants’ response time.

## Production_all.csv

This files contains all the data of Word Repetition and Picture Naming.

Assigned number: The number assigned to this trial. This is used to match trials when creating this combined file.

Subject: The assigned participant number.

Session: Which task and which session the trial is in. pretest: Word Repetition, pre-training test; posttest: Word Repetition, post-training test; picturenaming: Picture Naming; nativespeaker: the trial produced by native speaker These are included to examine the reliability of raters.

Pinyin_correct: The pinyin used in this trial.

Tone_correct: The tone used in this trial.

Word type: Whether this word is used in training (trained) or not (untrained). This factor is only used for Word Repetition.

Pinyin: The pinyin produced by the participant, transcribed by the first rater.

Tone: The tone produced by the participant, transcribed by the first rater.

Rating: The rating of tone given by the first rater.

Tone_score: Whether participant answered the tone of this trial correct (1 = correct, 0 = incorrect) according to the first rater.

Pinyin_score: Whether participant answered the pinyin of this trial correct (1 = correct, 0 = incorrect) according to the first rater.

Reference: The assigned number used to create this combined file.

Pinyin_rater1: The pinyin produced by the participant, transcribed by the second rater.

Tone_rater1: The tone produced by the participant, transcribed by the second rater.

Rating_rater1: The rating of tone given by the second rater.

Tone_score_rater1: Whether participant answered the tone of this trial correct (1 = correct, 0 = incorrect) according to the second rater.

Pinyin_score_rater1: Whether participant answered the pinyin of this trial correct (1 = correct, 0 = incorrect) according to the second rater.

Condition: The variability condition the participant is assigned to. 0 = Low variability; 1 = High variability; 2 = High variability blocked
